# Mapping persistence and change in psychological problems during the transition to adolescence: Adding, subtracting, shifting, and persisting

**DOI:** 10.1002/jcv2.70043

**Published:** 2025-09-03

**Authors:** Brooks Applegate, Benjamin B. Lahey

**Affiliations:** ^1^ Educational Leadership Research and Technology Western Michigan University Kalamazoo Michigan USA; ^2^ Public Health Sciences University of Chicago Chicago Illinois USA

**Keywords:** causal models, developmental psychopathology

## Abstract

**Background:**

Recent developmental models posited that general tendencies to exhibit psychological problems are relatively stable, but specific problems change frequently. We need comprehensive descriptions of persistence and change in psychological problems before advancing such theories, however.

**Methods:**

Data from four annual assessments of 9806 children (ages 9–10 years at baseline) in the Adolescent Behavior Cognitive Development Study™ were used to quantify persistence and change in each of 10 parent‐rated specific psychological problems.

**Results:**

Novel pairwise analyses revealed that the persistence of psychological problems over 1–3 years was common, but behavior change in the sense that problem *x*_1_ at baseline desisted and was replaced by a new problem *y*_2_ at follow‐up was uncommon. The only relatively common changes in behavior over time involved adding a new problem (i.e., *x*_1_ at baseline followed by *x*_2_ + *y*_2_ at follow‐up) or subtracting a problem (i.e., *x*_1_ + *y*_1_ at baseline followed by only *y*_2_ at follow‐up).

**Conclusions:**

If confirmed across other measures and developmental epochs, these findings challenge a key postulate of current theories that the developmental course of psychological problems involves frequent replacement of one problem by another.

## Key Points

- Recent models posit that general tendencies to exhibit psychological problems are relatively stable, but specific problems often change into other problems.
- Novel analyses of data during adolescence partitioned longitudinal paths of specific psychological problems.
- The persistence of psychological problems over 1–3 years was common, but behavior change in the sense that problem *x*_1_ at baseline desisted and was replaced by a new instance of problem *y*_2_ at follow‐up was uncommon. The only relatively common changes observed involved *adding* a new problem or *subtracting* an existing problem from a pair of problems at baseline.
- These findings suggest the need for better descriptions of change and stability in psychological problems to revise and improve developmental theories.

## Graphical abstract

Pathways from a psychological problem at baseline to a different problem at follow‐up that are conflated in traditional 2 × 2 calculations of heterotypic change (left panel), but parsed in 4 × 4 analyses (right panel). Note that the same children who are in the shaded cells in the 2 × 2 tables in the left panel are in the shaded cells in the pairwise 4 × 4 analyses in the right panel. The bottom rows in each panel provide an empirical example from ABCD Study data for two problems (dichotomized at the high rating cut of 0 or 1 vs. 2) from baseline to the first annual follow‐up.

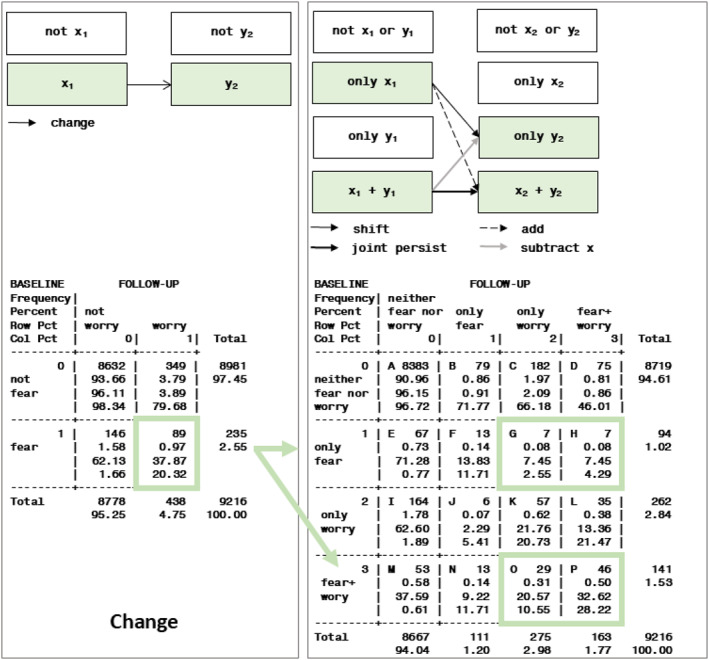

## INTRODUCTION

Several theorists have posited that general tendencies to exhibit psychological problems^1^ are relatively stable, but specific psychological problems often change over time (Beauchaine & McNulty, 2013; Caspi & Moffitt, 2018; Lahey et al., 2017; Oldehinkel & Ormel, 2023). Three quotes exemplify this postulate:

1. “Mental disorders … do not simply go away, instead often morphing with time into other, different conditions.” (Caspi et al., 2014) (Page 840).
2. “…the underlying pleiotropic liabilities are relatively unchanging but often give rise to changing symptomatic manifestations over time…” (Lahey et al., 2014) (Pages 993–994).
3. “…phenotypic plasticity, wherein the pattern of symptoms morphs across the life course” (Nolen‐Hoeksema & Watkins, 2011) (Page 591).

If this view is correct, developmental theories of psychological problems must explain changing phenotypes. The discovery of the causal pathways to “problem x” is relatively straightforward if some persons stably exhibit problem *x* and others do not. It is more challenging, however, if we are studying a moving target—if problem *x* often changes into problem *y* over time (Petersen et al., 2020). We assert, however, that these theorists—including ourselves—prematurely proposed theoretical explanations of change in psychological problems before achieving an accurate description of persistence and change. Therefore, we begin the process of moving the empirical horse in front of the theoretical cart in this paper. This effort cannot be accomplished in a single study and will require multiple large studies across informants and spans of life, but the present analyses provide a beginning.

Our understanding of stability and change in psychological problems has been both advanced and obscured by previous longitudinal studies of categorical diagnoses (Oldehinkel & Ormel, 2023; Shevlin et al., 2017). These studies suggest that essentially every categorical mental disorder predicts every other categorical mental disorder to varying degrees over time, suggesting considerable change (Caspi et al., 2020; Costello et al., 2003; Lahey et al., 2014; Plana‐Ripoll et al., 2019). Nonetheless, describing persistence and change using categorical diagnoses can be misleading. Desisting from categorical diagnosis A present at baseline and then meeting criteria for diagnoses B for the first time at follow‐up could reflect the cessation of up to all symptoms of diagnosis A and the emergence of up to all new symptoms of diagnosis B at follow‐up, but it could equally reflect the cessation of a single symptom of A and the emergence of one new symptom of B. To avoid this ambiguity, we conducted fine‐grain longitudinal analyses of persistence and change in specific psychological problems rather than diagnoses. This approach has the virtue of being consistent with the movement in the field toward dimensional models defined by counts of problems (Kotov et al., 2017; Lahey, 2021).

Furthermore, we suggest that the statistical methods used in previous studies to quantify change were misleading. Heretofore, change in psychological problems has been quantified using 2 × 2 tables of the presence or absence of problem *x*_1_ at baseline by the presence or absence of problem *y*_2_ in a follow‐up assessment. This is insufficient because it allows many different transitions to “count” as change simply because *x*_1_ at baseline is followed by *y*_2_ at follow‐up. Oldehinkel and Ormel (2023) distinguished between “pure” change in which *x*_1_ desists and is followed by a new instance of *y*_2_ and “mixed” change in which *x*_1_ persists and is followed by *x*_2_ + *y*_2_. We formalized their thinking to develop a new *pairwise strategy* that distinguishes all pathways of persistence and change. This strategy uses 4 × 4 tables in which each predictor problem either occurs by itself at baseline (only *x*_1_) or occurs simultaneously with the outcome problem at baseline (*x*_1_ + *y*_1_). Similarly, each outcome problem can either occur by itself (*y*_2_) or occur simultaneously with the potentially persisting predictor problem (*x*_2_ + *y*_2_). Note that each analyzed psychological problem serves as both the “*x*” and “*y*” problem in the analyses of all pairs of problems.

### Parsed paths of persistence

**FIGURE 1:**
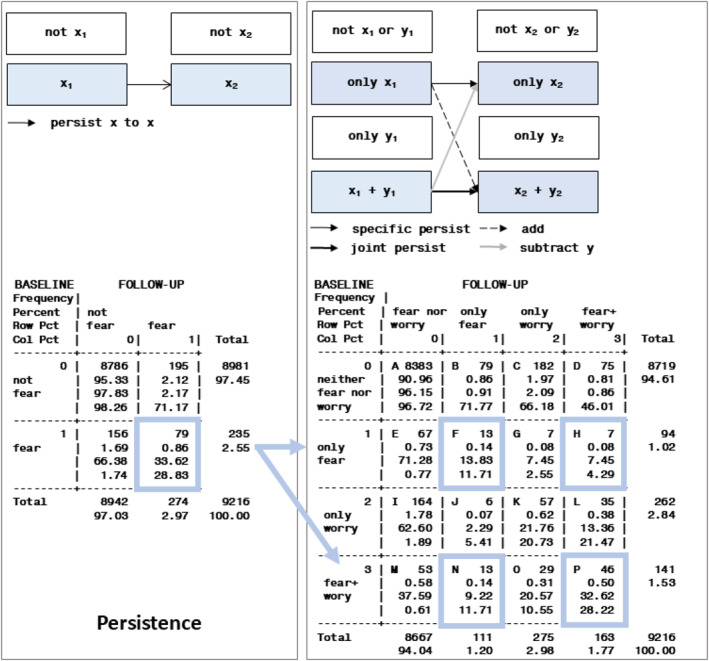
Conceptual illustrations in the top rows of the two panels describe the pathways from a psychological problem at baseline to the same problem at follow‐up that are conflated in traditional 2 × 2 table estimates of continuity (left panel) but are parsed in 4 × 4 analyses (right panel). Note that the *same children* who are in the shaded cells in the 2 × 2 tables in the left panel are in the shaded cells in the pairwise 4 × 4 analyses in the right panel. The bottom rows in each panel provide an empirical example from Adolescent Brain Cognitive Development Study data for two problems (dichotomized at the high rating cut of 0 or 1 vs. 2) from baseline to the first annual follow‐up. Note that the 79 children said to exhibit persistent fear in the boxed cell in the 2 × 2 analyses in the left panel are parsed into the four boxed cells representing distinct paths of persistence in the right panel (13 + 7 + 13 + 46 = 79) representing four different paths of persistence.

Traditional analyses based on 2 × 2 tables provide accurate quantitative estimates of total persistence, but Figure 1 shows that they combine four different paths of persistence that are distinguished in 4 × 4 models:

1. *Specific persist path*. Children with only *x*_1_ at baseline continue to exhibit only *x*_2_ at follow‐up.
2. *Add path*. Children with only *x*_1_ at baseline continue to exhibit *x*_2_ at follow‐up but also exhibit *y*_2_.
3. *Subtract y path*. Children with both *x*_1_ and *y*_1_ at baseline cease to exhibit problem *y*_1_ but continue to exhibit *x*_2_ at follow‐up.
4. *Joint persist path*. Children with both *x*_1_ + *y*_1_ at baseline exhibit *x*_2_ + *y*_2_ at follow‐up.

**FIGURE 2:**
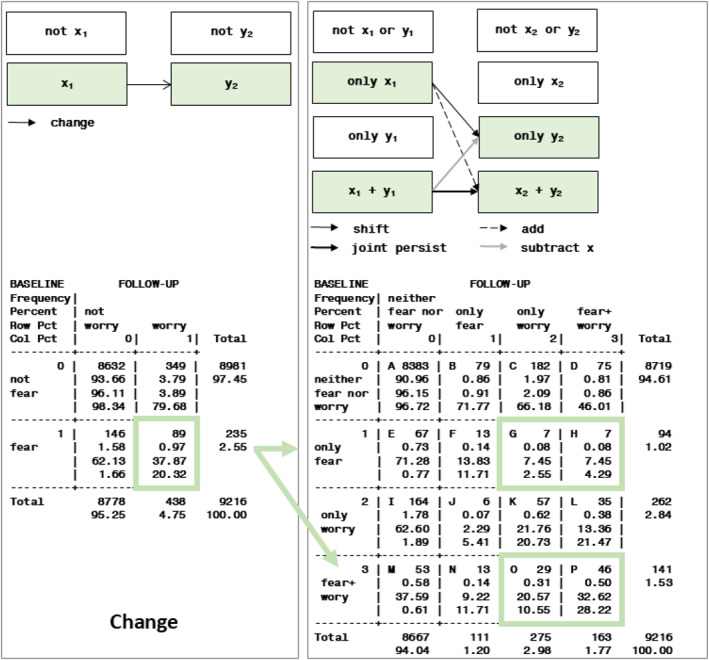
Conceptual illustrations in the top rows of the two panels describing the pathways from a psychological problem at baseline to different problems at follow‐up that are conflated in traditional 2 × 2 estimates of change (left panel) but parsed in 4 × 4 analyses (right panel). Note that the *same children* who are in the shaded cells in the 2 × 2 tables in the left panel are in the shaded cells in the pairwise 4 × 4 analyses in the right panel. The bottom rows in each panel provide an empirical example from Adolescent Brain Cognitive Development Study data for two problems (dichotomized at the high rating cut of 0 or 1 vs. 2) from baseline to the first annual follow‐up. Note that the 89 children said to exhibit change from fear to worry in the boxed cell in the 2 × 2 analyses in the left panel are parsed into the four boxed cells in the right panel (7 + 7 + 29 + 46 = 89), representing four different paths of change.

Our pairwise analyses are illustrated in Figures 1 and 2 using a concrete example from the actual data analyzed in this paper. As detailed later, parent ratings of these problem behaviors were dichotomized at the “high rating cut” (parent ratings of 0 or 1 of each item vs. parent ratings of 2). The illustration shows that traditional 2 × 2 tables accurately indicate that 79 of 235 children (33.62%) exhibited persistent fear because they exhibited fear at baseline and exhibited fear at follow‐up. The 4 × 4 table on the lower‐right side of Figure 1 parse this persistence into four paths. Thirteen of the 235 children followed a *specific persist* path from only fear at baseline to only fear at follow‐up (5.5%). Seven children with only fear at baseline followed an *add* path, in which they continued to exhibit fear at follow‐up but also added worry at follow‐up (3.0%). Many other children were on a *joint persist* path in which they exhibited both fear and worry at baseline and continued to exhibit both fear and worry at follow‐up (46/235 = 19.6%). Others were on a *subtract y* path in which they exhibited both fear and worry at baseline but only fear at follow‐up (13/235 = 5.5%). Note that the sum of the prevalences of these four different paths (13 + 7 + 46 + 13) equals the total of 79 overall instances of “persistent fear” in the 2 × 2 analyses shown on the left side of Figure 1.

### Parsed paths of change

Unlike estimates of persistence, however, traditional 2 × 2 analyses do not provide sensible estimates of *change* in psychological problems. As illustrated in Figure 2, four paths are conflated in 2 × 2 models that have been interpreted as evidence of change in problems. Surprisingly, some of these paths do not actually involve behavior change and three of the four parsed paths of change are the same as three of the parsed paths of persistence:

1. *Shift path*. Children with only problem *x*_1_ at baseline cease to exhibit that problem at follow‐up and exhibit only problem *y*_2_ at follow‐up. This represents “pure heterotypic” change in the terms used by Oldehinkel and Ormel (2023).
2. *Add path*. Children with only *x*_1_ at baseline subsequently exhibit both *x*_2_ and *y*_2_ at follow‐up. Note that such transitions “count” as change in traditional 2 × 2 tables because *x*_1_ precedes *y*_2_ and because *y*_1_ was not present at baseline. Nonetheless, the *add path* also involves persistence because *x*_1_ at baseline is followed by the same problem *x*_2_ at follow‐up (Oldehinkel & Ormel, 2023).
3. *Subtract x path*. Children with both *x*_1_ and *y*_1_ at baseline cease to exhibit problem *x*_1_ and exhibit only *y*_2_ at follow‐up. This path is said to reflect change in 2 × 2 analyses because *x*_1_ precedes *y*_2_, but there is no change from *x*_1_ to *y*_2_ because problem *y*_1_ was already present at baseline.
4. *Joint persist path*. Nonsensically, children with both *x*_1_ + *y*_1_ at baseline who continue to exhibit both *x*_2_ + *y*_2_ at follow‐up also contribute to quantitative estimates of change in 2 × 2 models simply because *x*_1_ precedes *y*_2_.

As illustrated in Figure 2, analysis of the 2 × 2 table on the lower‐left side shows that 89 of the 235 children who exhibited fear at baseline later exhibited worry at follow‐up (37.9%). These results are interpreted in traditional 2 × 2 analyses as reflecting substantial change from fear to worry. In contrast, analyses of the same data using pairwise 4 × 4 tables shown in the lower‐right of Figure 2 parse the 89 children into distinctly different pathways of change, some of which do not involve change from fear to worry at all. Only 7 of the 235 children with fear at baseline (3.0%) desisted from fear and exhibited a new instance of worry at follow‐up (i.e., the *shift path)*. An additional 7 of the 235 children with fear at baseline were on the *add path*, exhibiting only fear at baseline and retaining fear but adding worry at follow‐up (3.0%). Twenty‐nine of the 235 children (12.34%) were on the *subtract x path*, exhibiting both fear and worry at baseline but only worry at follow‐up. These children are said to exhibit change from fear to worry in the 2 × 2 analyses because fear precedes worry, even though worry was already present at baseline. Most illogically, the sizable number of children on the *joint persist* path (46/235 = 19.57%) contributed to traditional 2 × 2 estimates of change because fear at baseline preceded worry at follow‐up, even though these children exhibited the persistence of both problems over time.

Thus, previous estimates of change based on traditional 2 × 2 analyses overestimated the prevalence of change, perhaps to a substantial degree. Notably, some authors recognized this issue and attempted estimate change in mental disorder diagnoses more accurately by controlling the outcome diagnosis. These analyses generally revealed that estimates of change were substantially reduced, but often remained statistically significant when confounding on the outcome was controlled (Copeland et al., 2009; Keenan et al., 2009; Lahey et al., 2014; Oldehinkel & Ormel, 2023). Unfortunately, controlling confounding on the outcome only adjusts the estimate of change to the extent that the outcome was persistent from baseline and to the extent of the correlation between the two problems. Moreover, controlling on the outcome is an opaque process that does not reveal the specific predictor‐to‐outcome sequences underlying persistence and change as clearly as pairwise analyses based on 4 × 4 tables.

## METHOD

### Sample

Analyses were based on Curated Release 5.1 from the NIMH Data Archive (nda.nih.gov) from the baseline and three annual follow‐up assessments in the Adolescent Brain Cognitive Development (ABCD) Study®. The sample was recruited at 21 sites across the United States at 9–10 years of age (Garavan et al., 2018). Parent ratings of psychological problems were collected on 11,869 children. Most participants were one child from each family, but 2019 participants had a twin or non‐twin sibling in the sample. To avoid miss‐estimation of standard errors due to clustering within families, one child was randomly selected from each family. The numbers of children with non‐missing data and the demographics of the analyzed sample are in Table S1.

### Measure

Adult caregivers (85.12% biological mothers in the baseline assessment) rated their children's psychological problems using the Child Behavior Checklist (CBCL) (Achenbach, 2009). CBCL items describing child behaviors and emotions were rated on a scale of 0 = not true (as far as you know), 1 = somewhat or sometimes true, or 2 = very true or often true. The present analyses are based on selected psychological problems during the four assessments across late childhood and early adolescence. These analyses were limited to 10 problems to reduce the number of computations and facilitate understanding of the method and findings. We selected cardinal psychological problems that consistently exhibit high loadings on factors defining dimensions of fearfulness and anxiety (fearful, worry), depression (dysphoria, feelings of worthlessness), attention‐deficit hyperactivity disorder (inattention, hyperactivity), oppositional defiant disorder (arguing with adults, losing temper), and conduct disorder (fighting and stealing) (Lahey et al., 2004, 2015) in a proof of principle spirit. Because analyses of persistence and change require binary measures of the presence or absence of problems, the problem ratings were dichotomized for separate analyses at 0 versus 1 or 2 (low cut), and at 0 or 1 versus 2 (high cut).

### Statistical analyses

Four paths of persistence and change from baseline to each of three annual follow‐up assessments were quantified in separate sets of analyses. Because the pairwise analyses based on the 4 × 4 tables illustrated in Figures 1 and 2 were conducted individually for each pair of the 10 problems, 90 pairwise analyses of the *shift*, *add*, and *subtract* paths were conducted. Note that the *subtract x* and *subtract y* paths were combined in all analyses because the designation of *x* and *y* in each pair is arbitrary when both problems are present at baseline. In addition, 45 tests were conducted of the *joint persistence* path (*x*_1_ + *y*_1_ at baseline and *x*_2_ + *y*_2_ at follow‐up) for each baseline to follow‐up interval—half as many as the other paths because two problems jointly serve as the predictor and the outcome in each test.

These analyses determined if the observed rates of the wave‐to‐wave within‐person transitions that define each path occurred significantly more often than chance, defined as the empirical base‐rate for that path (i.e., the relative frequency of each outcome among children with neither the predictor nor the outcome at baseline). To illustrate this approach using the concrete example in Figure 2, when only fear at baseline was the predictor and only worry was the outcome at follow‐up, the *shift path* was defined by the 7 children exhibiting only worry at outcome among the 94 children exhibiting only fear at baseline (7.45%). The statistical significance of this path was evaluated against the empirical base‐rate of 182 children who exhibited only worry at follow‐up among the 8719 children with neither fear nor worry at baseline (2.09%).

Each path of persistence or change was tested separately in contingency tables by extracting four relevant frequencies from the 4 × 4 tables. For example, based on the illustration at the lower‐right of Figure 2, the *shift path* from only fear was defined by cell G (i.e., only worry) relative to the sum of cells E + F + H (i.e., any outcome except only worry) among children with only fear at baseline. The base‐rate for this path was defined by cell C (i.e., only worry) relative to the sum of cells A + B + D (i.e., any outcome except only worry) among children with neither fear nor worry at baseline. Tetrachoric correlations were calculated to quantify associations between predictors and outcomes because they are not influenced by differences in base‐rates.

The prevalence of each path of persistence and change was compared quantitatively. Separate sets of comparisons of paths for each pair of psychological problems determined if (a) the *add* path (only *x*_1_ to *x*_2_ + *y*_2_) was more common than the *shift path* (only *x*_1_ to only *y*_2_), (b) the *specific persist* path (only *x*_1_ to only *x*_2_) was more common than *shift* (only *x*_1_ to only *y*_2_), and (c) the *joint persist* path (*x*_1_ + *y*_1_ to *x*_2_ + *y*_2_) was more common than the *subtract* path (*x*_1_ + *y*_1_ to only *y*_2_). Control of false discovery rates for all analyses is described in the Supplement.

## RESULTS

### Persistence quantified in 2 × 2 analyses

To frame the results of the 4 × 4 analyses, it is useful to quantify the overall persistence of psychological problems using 2 × 2 analyses. Across all paths, whether beginning with either only *x*_1_ or both *x*_1_ + *y*_1_, the 10 problems that were rated by parents as present at the low rating cut at baseline were rated as present again in the first annual follow‐up an average of 59.92% of the time (range: 43.59%–76.93%). The corresponding overall persistence estimates were 52.83% from baseline to the second follow‐up and 49.35% over the 3 years to the third follow‐up. The averages for the overall persistence of the 10 problems at the more stringent high rating cut from baseline were 36.94%, 30.23%, and 27.22% over 1, 2, and 3 years.

### Paths of persistence revealed in pairwise analyses

**FIGURE 3:**
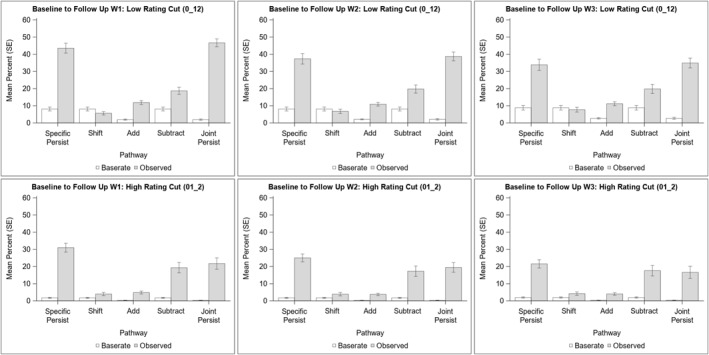
Mean percent of children following each path from the baseline assessment to each of the three annual follow‐ups based each rating cut based on the results of the 4 × 4 pairwise analyses of the specific persist path illustrated on the right‐hand side of Figure 1 and the four paths of change illustrated on the right‐hand side of Figure 2.

Rates of persistence of psychological problems in the four parsed paths are shown in Table 1 and illustrated in Figure 3. At the low rating cut, when one of the 10 psychological problems in the 90 pairs occurred by itself at baseline, persistence on the *specific persist* path (only *x*_1_ to only *x*_2_) was observed 43.53% of the time on average. In addition, the persistence of only *x*_1_ at baseline was observed on the *add* path (only *x*_1_ to both *x*_2_ + *y*_2_) 11.85% of time on average. Each of these rates of persistence significantly exceeded their corresponding base‐rates for all 90 pairs of problems (Table 1).

**TABLE 1:** Summary of the results of all pairwise analyses detailed in Tables S3A—S3L for the prevalence of each path of persistence and change illustrated in Figures 1 and 2 its corresponding base‐rate.

Reference path: Specific persist								
FU yr	Observed % (only *x* to only *x*)		Base‐rate % (neither *x* nor *y* to only *x*)		Tetrachoric correlation (*r*_*t*_) observed versus base‐rate		N of tetrachoric correlations that were FDR‐adj significant, indicating that the prevalence of shift was > or < its base‐rate	
	Range	Mn	Range	Mn	Range	Mn	>	<
Dichotomized at low rating cut (0 vs. 1 or 2)								
1	13.33–72.80	43.53	0.55–18.66	8.09	0.48–0.80	0.66	90	0
2	5.15–69.34	37.33	0.34–19.30	8.10	0.46–0.74	0.60	90	0
3	3.57–67.92	33.89	0.39–23.60	8.84	0.28–0.69	0.53	90	0
Dichotomized at high rating cut (0 or 1 vs. 2)								
1	0.00–54.78	30.99	0.07–4.88	1.77	0.51–0.81	0.71	88	0
2	4.62–47.00	25.02	0.70–4.94	1.66	0.33–0.80	0.64	90	0
3	0.00–44.81	21.55	0.08–5.76	1.95	0.28–0.74	0.58	87	0

Parsed heterotypic paths: Shift								
FU yr	Observed % (only *x* to only *y*)		Base‐rate % (neither *x* nor *y* to only *y*)		Observed versus base‐rate (*r*_*t*_)			
Dichotomized at low rating cut (0 vs. 1 or 2)								
1	0.23–23.23	5.64	0.55–18.66	8.09	−0.40 to 0.08	−0.12	0	50
2	0.17–27.12	6.74	0.34–19.30	8.10	−0.32 to 0.13	−0.08	1	38
3	0.10–30.95	7.41	0.39–23.60	8.84	−0.31 to 0.16	−0.06	3	36
Dichotomized at high rating cut (0 or 1 vs. 2)								
1	0.00–18.75	3.80	0.06–4.90	1.77	−0.17 to 0.34	0.12	22	0
2	0.00–26.67	3.91	0.07–4.94	1.76	−0.14 to 0.39	0.13	20	0
3	0.00–27.27	4.59	0.09–5.64	1.95	−0.12 to 0.44	0.13	22	1

Parsed heterotypic paths: Add								
FU yr	Observed % (only *x* to *x* + y)		Base‐rate % (neither *x* nor *y* to *x* + y)		Observed versus base‐rate (*r*_*t*_)			
Dichotomized at low rating cut (0 vs. 1 or 2)								
1	1.94–26.03	11.85	0.38–5.03	1.94	0.30–0.68	0.48	90	0
2	1.88–24.23	10.92	0.36–5.31	2.10	0.25–0.64	0.44	90	0
3	2.23–25.10	11.19	0.39–6.97	2.70	0.22–0.60	0.40	90	0
Dichotomized at high rating cut (0 or 1 vs. 2)								
1	0.00–19.05	4.66	0.01–1.23	0.25	0.26–0.79	0.52	75	0
2	0.00–16.05	3.76	0.00–1.10	0.24	0.28–0.80	0.50	76	0
3	0.00–13.04	3.84	0.00–1.33	0.32	0.12–0.68	0.45	70	0

Parsed heterotypic paths: Subtract^a^								
FU yr	Observed % (*x* + *y* to only *y*)		Base‐rate % (neither *x* nor *y* to only *y*)		Observed versus base‐rate (*r*_*t*_)			
Dichotomized at low rating cut (0 vs. 1 or 2)								
1	2.82–44.83	18.70	0.55–18.66	8.09	−0.07 to 0.60	0.28	85	1
2	2.79–51.14	19.74	0.34–19.80	8.10	−0.05 to 0.56	0.30	85	0
3	1.51–53.83	19.81	0.39–23.60	8.84	−0.11 to 0.51	0.27	83	1
Dichotomized at high rating cut (0 or 1 vs. 2)								
1	0.00–50.00	19.31	0.70–4.88	1.77	0.29–0.69	0.49	76	0
2	0.00–52.38	17.27	0.07–4.94	1.76	0.22–0.77	0.44	69	0
3	0.00–57.89	17.60	0.08–5.76	1.93	0.14–0.69	0.44	70	0

Parsed heterotypic paths: Joint persist^b^								
FU yr	Observed % (*x* + *y* to *x* + *y*)		Base‐rate % (neither *x* nor *y* to *x* + *y*)		Observed versus base‐rate (*r*_*t*_)			
Dichotomized at low rating cut (0 vs. 1 or 2)								
1	32.41–64.80	46.64	0.38–5.03	1.94	0.80–0.93	0.86	45	0
2	24.11–61.47	38.73	0.36–5.31	2.10	0.71–0.88	0.81	45	0
3	17.70–61.00	34.90	0.39–6.97	2.70	0.63–0.83	0.75	45	0
Dichotomized at high rating cut (0 or 1 vs. 2)								
1	0.00–50.00	23.19	0.01–1.23	0.25	0.60–0.91	0.79	42	0
2	0.00–38.89	19.46	0.00–1.10	0.24	0.62–0.87	0.76	40	0
3	0.00–50.00	15.58	0.00–1.33	0.32	0.40–0.93	0.67	38	0

^a^ Subtract *x* and subtract *y* paths were combined because the designation of *x* and *y* in each pair is arbitrary when both are present at baseline.

^b^ The possible number of tetrachoric correlations calculated for the joint persist path is 45 instead of 90. As shown in Tables S3A‐S3L, tetrachoric correlations and associated *p* levels could not be estimated in all cases because some cells had 0 observations.

When a problem was present at baseline along with another problem from each of the 90 pairs, two paths of persistence were observed. Table 1 shows that, on average, both members of pairs of problems (*x*_1_ + *y*_1_) at baseline persisted to *x*_2_ + *y*_2_ in the first annual follow‐up on the *joint persist* path 46.64% of the time at the low rating cut and 23.19% of the time at the high rating cut. In addition, on the *subtract* path one of the two baseline problems persisted over 12 months an average of 18.70% of the time at the low rating cut and 19.31% of the time at the high rating cut. Persistence on the joint persist and subtract paths from the presence of two problems at baseline both significantly exceeded their corresponding base‐rates for nearly all problems. Together, persistence was observed on these two paths 65.34% of the time at the low rating cut and 42.5% of the time at the high rating cut over 12 months. The levels of persistence on these paths were somewhat lower at over longer follow‐up intervals for both rating cuts but were significantly above their base‐rates.

### Paths of change revealed in pairwise analyses

The results in Table 1 and Figure 3 also provide a detailed description of change in individual psychological problems over time. Change in the strong sense of “pure heterotypic” change defined by Oldehinkel and Ormel (2023) on the *shift* path (only *x*_1_ to only *y*_2_) occurred infrequently at rates that were at or below the corresponding base‐rates for *y*_2_ at the low rating cut in all three follow‐ups. Change on the *shift* path was also uncommon at the high rating cut, occurring 3.80%–4.59% of the time in the three follow‐ups. Unlike the low rating cut, these rates significantly exceeded the corresponding base‐rates (1.76%–1.95%), but only for about one‐quarter of the 90 problem pairs.

Table 1 and Figure 3 show that change in the sense of adding a new problem (from only *x*_1_ at baseline to both *x*_2_ + *y*_2_ on the *add* path) occurred an average of 10.92%–11.85% of the time for the 10 problems across the three follow‐up intervals. These rates were significantly greater than the base‐rates for every pair of items in each of the annual follow‐ups. The same pattern was found at the high rating cut, but at lower absolute numbers of significant *add* path transitions.

### Comparisons of the prevalence of the parsed paths

Table 2 shows that, across all 90 pairs of problems, when only *x*_1_ was present at baseline, the *add* path was significantly more common than the *shift* path at the low rating cut across all waves. Results were similar at the high rating cut across but at lower absolute rates. Table 2 also shows that when only *x*_1_ was present at baseline, children were markedly more likely to continue to exhibit the same problem—on the *specific persist* path (only *x*_1_ to only *x*_2_)—than to change to a different problem on the *shift* path at both the low and high rating cuts. When both problems in each of the 90 pairs were present at baseline (*x*_1_+ *y*_1_), Table 2 shows that both problems jointly persisted to *x*_2_ + *y*_2_ far more often than one of the two problems desisted on the *subtract* path at the low and high rating cuts.

**TABLE 2:** Summary of results presented in Tables S3M—S3R for comparisons of the prevalence of the five paths of continuity and change illustrated in Figures 1 and 2 based on every pair of each psychological problem treated as predictors at baseline and outcomes in each of the three annual follow‐up assessments.

Comparison: Is *adding* (to *x*_2_ + *y*_2_) more common than *shifting* (to only *y*_2_) following only *x*_1_?						
FU YR	Tetrachoric correlation (*r*_*t*_) (Mn, range)	Mean absolute number of shifts to y	Mean absolute number of adds *y*	Mean % (adding/adding or shifting) following only x	*p* (unadj) range	Number (%) of FDR‐adj significant *r*_*t*_ indicating that add was more common than shift for each problem pair/number of calculated *r*_*t*_
Dichotomized at low rating cut (0 vs. 1 or 2)						
1	0.69 (0.45–0.86)	42.88	141.11	71.52	0.0000–0.0000	90/90 = 100%
2	0.61 (0.36–0.80)	48.15	131.73	66.16	0.0000–0.0007	90/90 = 100%
3	0.55 (0.26–0.76)	52.10	128.16	63.86	0.0000–0.0002	90/90 = 100%
Dichotomized at high rating cut (0 or 1 vs. 2)						
1	0.60 (0.18–0.88)	5.62	15.94	61.00	0.0000–0.5735	60/76 = 78.95%]
2	0.58 (0.10–0.88)	5.47	10.10	59.59	0.0000–0.7404	58/72 = 80.56%
3	0.51(0.02–0.95)	5.44	9.23	54.08	0.0000–0.9298	52/73 = 71.23%

Comparison: Is *specific persist* (to only *x*_2_) more common than *shifting* (to only *y*_2_) following only *x*_1_?						
FU YR	Tetrachoric correlation (Mn, range)	Mean absolute number of shifts	Mean absolute number of specific persists	Mean % specific persist versus shifting following the predictor (only x)	*p* (unadj) range	Number (%) of FDR‐adj significant *r*_*t*_ indicating that specific persist was more common than shift for each problem pair/number of calculated *r*_*t*_
Dichotomized at low rating cut (0 vs. 1 or 2)						
1	0.65 (0.33–0.90)	42.88	641.50	84.27	0.0000–0.0000	90/90 = 100%
2	0.58 (0.27–0.83)	48.16	642.81	82.17	0.0000–0.0007	90/90 = 100%
3	0.52 (0.28–0.76)	52.10	559.61	77.99	0.0000–0.0002	90/90 = 100%
Dichotomized at high rating cut (0 or 1 vs. 2)						
1	0.60 (0.21–0.89)	5.62	129.29	85.87	0.0000–0.1711	75/79 = 94.94%
2	0.54 (0.17–0.89)	5.47	104.07	84.91	0.0000–0.3722	74/82 = 87.80%
3	0.48 (−0.00 to 0.77)	5.54	87.66	79.97	0.0000–0.9935	61/76 = 80.26%

Comparison: Is *joint persist* (to *x*_2_+ *y*_2_) more common than *subtracting* ^a^ (to only one problem at follow‐up) following *x*_1_+ *y*_1_?						
FU YR	Tetrachoric correlation (Mn, range)	Mean absolute number of subtracts	Mean absolute number of joint persists	Mean % joint persist versus subtracting following the predictor (*x*_1_+ *y*_1_)	*p* (unadj) range	Number (%) of FDR‐adj significant *r*_*t*_ indicating that joint persist was more common than subtract for each problem pair/number of calculated *r*_*t*_
Dichotomized at low rating cut (0 vs. 1 or 2)						
1	0.72 (0.53–0.88)	149.21	445.62	72.42	0.0000–0.0000	90/90 = 100%
2	0.65 (0.34–0.82)	153.92	368.33	67.76	0.0000–0.0086	90/90 = 100%
3	0.60 (0.32–0.81)	143.61	312.20	65.86	0.0000–0.0002	90/90 = 100%
Dichotomized at high rating cut (0 or 1 vs. 2)						
1	0.55 (0.06–0.83)	13.76	22.56	55.95	0.0000–0.8923	58/74 = 78.38%
2	0.60 (0.14–0.89)	11.60	17.33	57.80	0.0000–0.6600	65/73 = 89.04%
3	0.49 (0.03–0.81)	10.50	13.71	51.48	0.0000–0.8795	58/66 = 87.88%

Abbreviation: FU YR, Year of annual follow‐up evaluation.

^a^ Subtract *x* and subtract *y* paths were combined because the designation of *x* and *y* in each pair is arbitrary when both are present at baseline.

### Follow‐up analyses of the shift path

The present analyses focus on estimates of the extent of “pure heterotypic” change from one problem to another (Oldehinkel & Ormel, 2023) on the *shift* path to determine if explanations of change from one psychological problem to another are needed in developmental theories. To cast a wider net to capture all instances of “pure heterotypic” shifting, we examined rates of the *shift* path from only problem *x*_1_ at baseline to the occurrence of only problem *y*_2_ in *any* of the three follow‐up waves. The extent of such shifting was evaluated against the base‐rate of only problem *y*_2_ in any of the three follow‐ups among children who did not exhibit either *x*_1_ or *y*_1_ at baseline. The results closely mirrored the results for the analyses of the *shift* path to each separate follow‐up. As detailed in Table S4A, at the low cut, shifting from only *x*_1_ to only *y*_2_ in at least one of the three follow‐up waves occurred an average of 13.53% of the time, which was below the average 16.14% base‐rate. Of the 90 pairwise tests of observed rates of shifting from only *x*_1_ to only *y*_2_ in any of the three follow‐up waves at the high rating cut, shifting (8.29% on average) was slightly but significantly more common than the corresponding base‐rates (3.84% on average) in 38 of the 90 pairs at the high rating cut.

An additional follow‐up analysis examined the extent to which changing from one problem to another on the *shift* path might be more common between pairs of problems within the same broad internalizing or externalizing domains, as has observed for categorical mental disorders in adults (Lahey et al., 2014). Table S2 shows that all psychological problems were positively and significantly correlated concurrently within each assessment wave after FDR correction, consistent with the large literature on the robustly correlated nature of child and adolescent psychological problems (Lahey, 2021). Table S2 also shows that concurrent tetrachoric correlations in the baseline assessment across low and high cuts were significantly greater between pairs of problems within broad internalizing or externalizing domains (range 0.42–0.84, MN = 0.55; SE = 0.02) than across domains (range 0.19–0.62, MN = 0.38; SE = 0.02), *t* (87) = −7.57, *p* < 0.0001. Although rates of the *shift* path were modest, problem pairs that were more strongly correlated cross‐sectionally at baseline were more likely to exhibit shifting from only *x*_1_ to only *y*_1_ over 12 months, Spearman's *r* (90) = 0.58, *p* < 0.0001, at the low rating cut, and Spearman's *r* (81) = 0.22, *p* < 0.0502, at the high rating cut.

## DISCUSSION

The present findings show that individual psychological problems during the transition to adolescence are far more persistent than changeable. When defined at the low rating cut, parent‐rated psychological problems were found to persist over one to 3 years about half the time. At the more stringent high rating cut, parent‐rated problems persisted over the same time periods one‐quarter to one‐third of the time. Specifically, when one problem from each of the 90 pairs was present by itself at baseline, children were 10–15 times more likely to continue to exhibit only the same problem than to cease to display that problem and instead exhibit a different problem on the *shift* path. Furthermore, when change from a single problem at baseline did occur, it was 2–3 times more likely for a second problem to be added than for the problem to shift to a different problem.

When both members of each problem pair were present at baseline, joint persistence from *x*_1_ + *y*_1_ at baseline to *x*_2_ + *y*_2_ occurred 34%–46% of the time from baseline to each of the three follow‐ups at the low rating cut and 15%–23% of the time at the high rating cut. These levels of persistence significantly exceeded the corresponding base‐rates for nearly all pairs of problems. Furthermore, children exhibiting *x*_1_ + *y*_1_ at baseline were more than twice as likely to follow the *joint persist* path than for one problem in the pair to cease to be present at follow‐up on the *subtract path* at the low rating cut, and 30%–60% more likely at the high rating cut.

The present findings are consistent with a recent narrative review of studies of broad domains of internalizing and externalizing problems. Oldehinkel and Ormel (2023) concluded that within‐person change from one broad domain to another appears to be less common than most developmental theories suppose. Consistent with their conclusion, the present follow‐up analyses showed that problem pairs within the same broad domains were significantly more likely to exhibit shifting from only *x*_1_ to only *y*_1_ over time. The review by Oldehinkel and Ormel (2023) was couched in terms of the theoretical constructs of “homotypic continuity” and “heterotypic continuity”. We did not use those terms because different theorists define them in different ways (Cicchetti & Rogosch, 2002; Rutter et al., 2006). The present study provides descriptive findings on operationally defined patterns of change that could be used by theorists to address those constructs, however.

The present findings argue against the prevailing view that psychological problems frequently change across development. Theorists may have proposed explanation for a level of change that does not occur partly because statistical methods based on 2 × 2 tables accurately quantify of persistence but substantially overestimate change (Figure 2). Previous attempts to deal with this issue by controlling confounding on the outcome were useful, but analyses of 4 × 4 tables parse paths of persistence and change in more comprehensive and straightforward ways.

Additionally, it is likely that previous assertions that mental health problems change frequently (Caspi et al., 2020; Lahey et al., 2014, 2017) were based on misunderstandings of the limits of categorical diagnoses and a lack of full appreciation for the robustly correlated nature of individual psychological problems. To this point, changes over time from one categorical diagnosis to another can occur even when there is little change in the specific psychological problems on which the categorical diagnoses were based. Because cross‐sectional correlations among psychological problems are substantial and ubiquitous (Lahey, 2021), persons with enough relevant problems to meet diagnostic criteria for major depression, for example, are very likely to also exhibit problems associated with generalized anxiety and other problems. Therefore, a decline in one depression problem and the emergence of a single new anxiety problem over time could lead to the apparent desistance of depression and the incidence of a new case of generalized anxiety. To be useful, future causal theories will need to rest on a more detailed and nuanced description of persistence and change at the level of specific psychological problems.

## LIMITATIONS AND FUTURE DIRECTIONS

The present data provides only a small part of the evidence needed to fully understand the course of psychological problems over the life span. The current analyses benefit from a large amount of data collected over 4 years on children of similar ages in the baseline assessment, but the results could have differed if change had been studied using other informants and measures and/or across other segments of the life span.

In addition, future studies should address developmental transitions from psychological problems that are common in childhood to important problems that begin for the first time in adolescence. For example, the misuse of psychoactive substances increases in prevalence during adolescence from a complete absence at earlier ages. In such cases, there could be pure heterotypic shifts from earlier psychological problems that are declining in prevalence with age and are replaced by substance misuse. Nonetheless, it is also possible that substance misuse represents an addition to persisting psychological problems. In data from future waves of the ABCD Study when substance misuse is common, it will be important to use pairwise analyses to examine this issue. Larger samples will be needed in the future to examine potential sex/gender and other demographic differences in stability and change.

## AUTHOR CONTRIBUTIONS

**Brooks Applegate**: Conceptualization; formal analysis; writing—review and editing. **Benjamin B. Lahey**: Conceptualization; formal analysis; writing—original draft.

## CONFLICT OF INTEREST STATEMENT

The authors declare no conflicts of interest.

## ETHICAL CONSIDERATTIONS

Data were collected at each site with informed consent for sharing de‐identified data under institutional review board approval from each university participating in the ABCD Study. Waivers of consent were obtained in 2024 from the Institutional Review Boards of the University of Chicago and Western Michigan University for analyses of the public use de‐identified data obtained with permission from the National Institute of Mental Health Data Archive.

## Supporting information

Supporting Information S1

## Data Availability

The Adolescent Brain Cognitive Development (ABCD) Study data used in the present analyses are openly available from the NIMH Data Archive (NDA) (https://abcdstudy.org). SAS code is available from the authors.

## ENDNOTE

^1^
The term “psychological problems” is used here as a denotative synonym for “psychopathology,” but is preferred because psychopathology literally means “sick mind,” which fosters stigma (Lahey, 2021). The terms “specific psychological problem” is a denotative synonym of “symptom of psychopathology.”
