# Supplementary material for: Mapping persistence and change in psychological problems during the transition to adolescence: Adding, subtracting, shifting, and persisting
Source: JCPP Adv. 2025 Sep 3;6(2):e70043. doi: 10.1002/jcv2.70043 (PMC13260686; doi:10.1002/jcv2.70043)
Supplement: Supplementary file 1 — Supporting Information S1 [file JCV2-6-e70043-s001.docx]

Mapping Persistence and Change in Psychological Problems during the

Transition to Adolescence: Adding, Subtracting, Shifting, and Persisting

Brooks Applegate and Benjamin B. Lahey

Supplemental Figures and Tables

Contents

1. Control of False Discovery.

2. Supplemental Table S1. Baseline demographic characteristics of the subsample used in analyses (N = 9,807).

3. Supplemental Table S2. Cross-sectional tetrachoric correlations among each of the 10 problems in the baseline and three annual follow-up assessments when problems were dichotomized at the low severity cut (parent ratings of 0 vs 1 or 2, above the diagonal) and at the high severity cut (parent ratings of 0 or 1 vs 2, below the diagonal).

Supplemental Tables S3A – S3F. Tetrachoric correlations from 4 x 4 pairwise analyses of all 90 combinations of the 10 selected psychological problems with every other problem to quantify the associations of each baseline problem with the same problem assessed in three annual follow-ups in one of the two homotypic paths (the **specific persist path** defined by only x_1_ at baseline and only x_2_ at follow-up) for problems dichotomized at the low and high rating cuts.

Supplemental Tables S3G – S3L. Tetrachoric correlations from 4 x 4 pairwise analyses of all 90 combinations of the 10 selected psychological problems with every other problem to quantify the associations of each baseline problem with outcomes in each of the three annual follow-ups that define four parsed paths traditionally conflated in estimates of change: ***add path*** (x_1_ at baseline and x_2_ + y_2_ at follow-up); ***joint persist path*** (x_1_ + y_1_ at baseline and x_2_ + y_2_ at follow-up); ***shift path*** (only x_1_ at baseline and only y_2_ at follow-up); ***subtract x path*** (x_1_ + y_1_ at baseline and only y_2_ at follow-up); and ***subtract y path*** (x_1_ + y_1_ at baseline and only x_2_ at follow-up). Results are presented for problems dichotomized at each of the rating cuts.

Supplemental Tables S3M – S3R. Tetrachoric correlations from 4 x 4 pairwise analyses of all 90 combinations of the 10 selected psychological problems with every other problem to compare the prevalence of three pairs of paths to each of the three annual follow-ups at the two rating cuts.

Supplemental Tables S4A – S4B. Results of sensitivity tests for only the ***shift path*** from only problem x at baseline to only problem y in **any one or more of the three annual follow-ups**. Results are for problems separately dichotomized at the low or the high rating cuts.

1. Control of False Discovery

Alpha was adjusted separately for eight families of analyses:

A. Alpha was adjusted separately for 6 sets of analyses conducted across each of the 12-, 24-, or 36- month periods from baseline to each of the three follow-ups. Within each time period, alpha was adjusted separately for analyses based on the low and high rating threshold cuts, for a total of three sets of analyses. The following tests were included in each family of analyses:

a. All tests of the *specific persist* path against its base-rates.

b. All tests of the *shift*, *add*, *subtract,* and *joint persist* paths against their base-rates.

c. All tests of the comparisons of the rates of the different paths.

B. In addition, alpha was adjusted for the sensitivity tests for *shifting* in any of the three annual outcome assessments, separately for the low and high rating threshold cuts.

The linear step-up method (Benjamini & Hochberg, 1995), the false discovery rate was controlled at 5% in each family of analyses. This study was not preregistered, but we report how we determined our sample size, all data exclusions, all manipulations, and all measures in the study.

Supplemental Table S1. Baseline demographic characteristics of the children with non-missing data parent rating of child psychological problems at baseline included in the analyses, and the ages and number of children with non-missing data in each wave, after the random selection of one child from each household with more than one participating child.

| Female (%) | 47.61 |
| --- | --- |
| Age in months at baseline (N) | MN = 118.87(18.76; range = 107-133 (N = 9806) |
| Age in months at follow-up 1 (N) | MN = 130.84; range = 117-149 (N = 9234) |
| Age in months at follow-up 2 (N) | MN = 144.14; range = 127-168 (N = 9037) |
| Age in months at follow-up 3 (N) | MN = 154.77; range = 137-177 (N = 8499) |
| Total family income | % |
| < $5,000 | 3.99 |
| $5,000-$11,999 | 4.06 |
| $12,000-$15,999 | 2.63 |
| $16,000-$24,999 | 4.93 |
| $25,000-$34,999 | 6.30 |
| $35,000-$49,999 | 8.65 |
| $50,000-$74,999 | 13.61 |
| $75,000-$99,999 | 14.50 |
| $100,000-$199,999 | 29.99 |
| ≥ $200,000 | 11.34 |
| Race-Ethnicity | % |
| Non-Hispanic white | 50.67 |
| Black | 15.22 |
| Hispanic | 21.36 |
| Asian | 2.34 |
| Other | 10.41 |

| Table S2. Cross-sectional tetrachoric correlations among each of the 10 problems in the baseline and three annual follow-up assessments when problems were dichotomized at the low severity cut (parent ratings of 0 vs 1 or 2, above the diagonal) and at the high severity cut (parent ratings of 0 or 1 vs 2, below the diagonal). | | | | | | | | | | |
| --- | --- | --- | --- | --- | --- | --- | --- | --- | --- | --- |
| Baseline Assessment (N = 9,801) | | | | | | | | | | |
|  | argues | attend | dysphoria | fearful | fights | hyper | steals | temper | worry | worthless |
| argues |  | **0.45** | 0.46 | 0.33 | **0.58** | **0.46** | **0.46** | **0.67** | 0.32 | 0.40 |
| attend | **0.55** |  | 0.40 | 0.35 | **0.42** | **0.78** | **0.47** | **0.42** | 0.28 | 0.44 |
| dysphoria | 0.54 | 0.45 |  | **0.50** | 0.44 | 0.33 | 0.37 | 0.48 | **0.55** | **0.67** |
| fearful | 0.43 | 0.48 | **0.61** |  | 0.27 | 0.33 | 0.21 | 0.38 | **0.67** | **0.52** |
| fights | **0.60** | **0.53** | 0.44 | 0.39 |  | **0.43** | **0.52** | **0.63** | 0.23 | 0.35 |
| hyper | **0.54** | **0.84** | 0.34 | 0.48 | **0.45** |  | **0.43** | **0.42** | 0.24 | 0.36 |
| steals | **0.44** | **0.52** | 0.38 | *0.19* | **0.45** | **0.47** |  | **0.45** | 0.23 | 0.36 |
| temper | **0.73** | **0.54** | 0.62 | 0.51 | **0.66** | **0.53** | **0.42** |  | 0.34 | 0.44 |
| worry | 0.41 | 0.44 | **0.63** | **0.81** | 0.40 | 0.39 | 0.19 | 0.46 |  | **0.53** |
| worthless | 0.49 | 0.48 | **0.67** | **0.62** | 0.44 | 0.33 | 0.29 | 0.56 | **0.64** |  |
| First Annual Follow-up Assessment (N = 9,220) | | | | | | | | | | |
|  | argues | attend | dysphoria | fearful | fights | hyper | steals | temper | worry | Worthless |
| argues |  | **0.44** | 0.43 | 0.31 | **0.56** | **0.44** | **0.48** | **0.66** | 0.30 | 0.41 |
| attend | **0.54** |  | 0.38 | 0.36 | **0.41** | **0.78** | **0.45** | **0.42** | 0.28 | 0.40 |
| dysphoria | 0.57 | 0.46 |  | **0.53** | 0.45 | 0.32 | 0.37 | 0.48 | **0.55** | **0.68** |
| fearful | 0.41 | 0.47 | **0.60** |  | 0.30 | 0.32 | 0.20 | 0.38 | **0.70** | **0.52** |
| fights | **0.64** | **0.51** | 0.55 | 0.30 |  | **0.44** | **0.52** | **0.64** | 0.25 | 0.37 |
| hyper | **0.57** | **0.85** | 0.43 | 0.40 | **0.49** |  | **0.43** | **0.44** | 0.22 | 0.33 |
| steals | **0.61** | **0.42** | 0.44 | 0.30 | **0.51** | **0.42** |  | **0.45** | 0.18 | 0.33 |
| temper | **0.78** | **0.52** | 0.58 | 0.46 | **0.67** | **0.55** | **0.60** |  | 0.33 | 0.46 |
| worry | 0.42 | 0.45 | **0.64** | **0.81** | *0.11* | 0.37 | 0.30 | 0.45 |  | **0.52** |
| worthless | 0.47 | 0.39 | **0.78** | **0.58** | 0.43 | 0.40 | 0.41 | 0.52 | **0.65** |  |
| Second Annual Follow-up Assessment (N = 8,974) | | | | | | | | | | |
|  | argues | attend | dysphoria | fearful | fights | hyper | steals | temper | Worry | Worthless |
| argues |  | **0.46** | 0.43 | 0.31 | **0.60** | **0.48** | **0.51** | **0.70** | 0.27 | 0.40 |
| attend | **0.58** |  | 0.39 | 0.36 | **0.41** | **0.75** | **0.48** | **0.43** | 0.31 | 0.40 |
| dysphoria | 0.43 | 0.41 |  | **0.56** | 0.39 | 0.32 | 0.41 | 0.47 | **0.56** | **0.68** |
| fearful | 0.33 | 0.38 | **0.67** |  | 0.30 | 0.35 | 0.26 | 0.36 | **0.71** | **0.57** |
| fights | **0.58** | **0.53** | 0.45 | *0.21* |  | **0.43** | **0.55** | **0.65** | 0.24 | 0.36 |
| hyper | **0.55** | **0.87** | 0.38 | 0.38 | **0.59** |  | **0.43** | **0.46** | 0.27 | 0.36 |
| steals | **0.48** | **0.55** | 0.33 | *0.20* | **0.60** | **0.42** |  | **0.47** | 0.22 | 0.36 |
| temper | **0.77** | **0.54** | 0.54 | 0.37 | **0.66** | **0.52** | **0.52** |  | 0.29 | 0.46 |
| worry | 0.36 | 0.40 | **0.64** | **0.83** | *0.14* | 0.37 | *0.16* | 0.48 |  | **0.56** |
| worthless | 0.44 | 0.46 | **0.78** | **0.68** | *0.28* | 0.35 | 0.35 | 0.60 | **0.64** |  |
| Third Annual Follow-up Assessment (N = 8,309) | | | | | | | | | | |
|  | argues | attend | dysphoria | fearful | fights | hyper | steals | temper | Worry | Worthless |
| argues |  | **0.46** | 0.39 | 0.31 | **0.59** | **0.47** | **0.50** | **0.67** | 0.30 | 0.42 |
| attend | **0.58** |  | 0.38 | 0.34 | **0.40** | **0.78** | **0.50** | **0.48** | 0.30 | 0.45 |
| dysphoria | 0.52 | 0.46 |  | **0.56** | 0.35 | 0.28 | 0.39 | 0.44 | **0.60** | **0.72** |
| fearful | 0.41 | 0.51 | **0.68** |  | 0.24 | 0.32 | 0.22 | 0.37 | **0.72** | **0.57** |
| fights | **0.63** | **0.50** | *0.25* | 0.30 |  | **0.42** | **0.58** | **0.63** | 0.26 | 0.40 |
| hyper | **0.55** | **0.84** | 0.32 | 0.35 | **0.40** |  | **0.47** | **0.47** | 0.26 | 0.37 |
| steals | **0.58** | **0.47** | 0.37 | 0.32 | **0.51** | **0.54** |  | **0.50** | 0.28 | 0.40 |
| temper | **0.76** | **0.52** | 0.44 | 0.45 | **0.66** | **0.51** | **0.58** |  | 0.37 | 0.48 |
| worry | 0.38 | 0.47 | **0.71** | **0.84** | 0.30 | 0.34 | 0.25 | 0.38 |  | **0.57** |
| worthless | 0.45 | 0.50 | **0.80** | **0.71** | 0.38 | 0.38 | 0.31 | 0.43 | **0.71** |  |

Tetrachoric correlations in italics in follow-up waves 1 and 2 were not significant at < 0.05 after FDR adjustment for each family of 90 tests for both rating thresholds within each wave of assessments. Tetrachoric correlations in bold are for pairs of problems within the same broad domains of internalizing or externalizing.

argues = Argues a lot; hyper = Can't sit still, restless, or hyperactive; fears = Too fearful or anxious;

worthless = Feels worthless or inferior; fights = Gets in many fights or Physically attacks people after dichotomization; steals = Steals at home or Steals outside the home after dichotomization; temper = Temper tantrums or hot temper; dysphoria = Unhappy, sad, or depressed; worries = Worries;

attend = Mean of can't concentrate, can't pay attention for long and inattentive or easily distracted, rounded before dichotomization.

| Supplemental Table S3A. Tetrachoric correlations from 4 x 4 pairwise analyses of all 90 combinations of the 10 selected psychological problems with every other problem to quantify the associations of each baseline problem with the same problem assessed in **the first annual follow-up** in the **specific persist path** defined by only x1 at baseline and only x2 at follow-up) for problems dichotomized at the **low rating cut (0 vs 1 or 2)**. rt = tetrachoric correlation. –CL and +CL = lower and upper 95% confidence intervals for the tetrachoric correlation. c00 = number without the predictor or the outcome; c10 = number with predictor without the outcome; c01 = number without the predictor with the outcome; c11 = number with the predictor and the outcome. Base% = percent of the outcome only x2 among children with neither x1 or the other member of the pair, y1, at baseline. Obs% = Observed percent of specific persistence (i.e., only x2 among children with only x1 at baseline). Raw P values significant after FDR adjustment in **bold**. | | | | | | | | | | | | | |
| --- | --- | --- | --- | --- | --- | --- | --- | --- | --- | --- | --- | --- | --- |
| prefix | Path | Predictor | Outcome | r_t_ | -CL | +CL | P | c00 | c01 | c10 | c11 | Base% | Obs% |
| bf | Specific Persist | fear | fear | 0.50 | 0.44 | 0.57 | **0.0000** | 5336 | 206 | 439 | 118 | 3.72 | 21.18 |
| ck | Specific Persist | worry | worry | 0.48 | 0.44 | 0.52 | **0.0000** | 4855 | 687 | 1019 | 631 | 12.40 | 38.24 |
| bf | Specific Persist | fear | fear | 0.64 | 0.61 | 0.67 | **0.0000** | 6092 | 575 | 797 | 646 | 8.62 | 44.77 |
| ck | Specific Persist | dysph | dysph | 0.56 | 0.51 | 0.61 | **0.0000** | 6365 | 302 | 371 | 154 | 4.53 | 29.33 |
| bf | Specific Persist | fear | fear | 0.63 | 0.60 | 0.66 | **0.0000** | 6067 | 591 | 774 | 633 | 8.88 | 44.99 |
| ck | Specific Persist | worth | worth | 0.62 | 0.57 | 0.67 | **0.0000** | 6372 | 286 | 356 | 178 | 4.30 | 33.33 |
| bf | Specific Persist | fear | fear | 0.62 | 0.58 | 0.67 | **0.0000** | 3765 | 294 | 381 | 264 | 7.24 | 40.93 |
| ck | Specific Persist | attend | attend | 0.71 | 0.69 | 0.73 | **0.0000** | 3501 | 558 | 1239 | 1894 | 13.75 | 60.45 |
| bf | Specific Persist | fear | fear | 0.66 | 0.62 | 0.69 | **0.0000** | 4747 | 468 | 531 | 493 | 8.97 | 48.14 |
| ck | Specific Persist | hyper | hyper | 0.72 | 0.70 | 0.75 | **0.0000** | 4794 | 421 | 981 | 996 | 8.07 | 50.38 |
| bf | Specific Persist | fear | fear | 0.62 | 0.58 | 0.66 | **0.0000** | 4098 | 310 | 464 | 303 | 7.03 | 39.50 |
| ck | Specific Persist | argues | argues | 0.66 | 0.63 | 0.68 | **0.0000** | 3713 | 695 | 1155 | 1629 | 15.77 | 58.51 |
| bf | Specific Persist | fear | fear | 0.64 | 0.61 | 0.68 | **0.0000** | 5225 | 487 | 621 | 519 | 8.53 | 45.53 |
| ck | Specific Persist | temper | temper | 0.63 | 0.60 | 0.66 | **0.0000** | 5256 | 456 | 863 | 617 | 7.98 | 41.69 |
| bf | Specific Persist | fear | fear | 0.70 | 0.67 | 0.72 | **0.0000** | 6012 | 752 | 764 | 986 | 11.12 | 56.34 |
| ck | Specific Persist | fights | fights | 0.70 | 0.65 | 0.75 | **0.0000** | 6619 | 145 | 298 | 130 | 2.14 | 30.37 |
| bf | Specific Persist | fear | fear | 0.70 | 0.68 | 0.72 | **0.0000** | 6121 | 799 | 792 | 1072 | 11.55 | 57.51 |
| ck | Specific Persist | steals | steals | 0.76 | 0.71 | 0.81 | **0.0000** | 6796 | 124 | 169 | 103 | 1.79 | 37.87 |
| bf | Specific Persist | worry | worry | 0.58 | 0.55 | 0.61 | **0.0000** | 4898 | 887 | 1130 | 1195 | 15.33 | 51.40 |
| ck | Specific Persist | dysph | dysph | 0.52 | 0.45 | 0.60 | **0.0000** | 5624 | 161 | 249 | 65 | 2.78 | 20.70 |
| bf | Specific Persist | worry | worry | 0.59 | 0.56 | 0.62 | **0.0000** | 4848 | 898 | 1099 | 1220 | 15.63 | 52.61 |
| ck | Specific Persist | worth | worth | 0.55 | 0.48 | 0.62 | **0.0000** | 5578 | 168 | 273 | 80 | 2.92 | 22.66 |
| bf | Specific Persist | worry | worry | 0.60 | 0.56 | 0.63 | **0.0000** | 3022 | 458 | 622 | 602 | 13.16 | 49.18 |
| ck | Specific Persist | attend | attend | 0.70 | 0.68 | 0.73 | **0.0000** | 3071 | 409 | 1148 | 1471 | 11.75 | 56.17 |
| bf | Specific Persist | worry | worry | 0.61 | 0.57 | 0.64 | **0.0000** | 3721 | 709 | 818 | 991 | 16.00 | 54.78 |
| ck | Specific Persist | hyper | hyper | 0.71 | 0.68 | 0.73 | **0.0000** | 4098 | 332 | 885 | 784 | 7.49 | 46.97 |
| bf | Specific Persist | worry | worry | 0.57 | 0.53 | 0.60 | **0.0000** | 3373 | 493 | 710 | 599 | 12.75 | 45.76 |
| ck | Specific Persist | argues | argues | 0.62 | 0.59 | 0.65 | **0.0000** | 3337 | 529 | 1087 | 1146 | 13.68 | 51.32 |
| bf | Specific Persist | worry | worry | 0.60 | 0.57 | 0.63 | **0.0000** | 4173 | 749 | 903 | 1027 | 15.22 | 53.21 |
| ck | Specific Persist | temper | temper | 0.61 | 0.58 | 0.65 | **0.0000** | 4581 | 341 | 737 | 440 | 6.93 | 37.38 |
| bf | Specific Persist | worry | worry | 0.66 | 0.63 | 0.68 | **0.0000** | 4697 | 1052 | 1029 | 1736 | 18.30 | 62.78 |
| ck | Specific Persist | fights | fights | 0.71 | 0.65 | 0.76 | **0.0000** | 5642 | 107 | 247 | 103 | 1.86 | 29.43 |
| bf | Specific Persist | worry | worry | 0.67 | 0.64 | 0.69 | **0.0000** | 4795 | 1100 | 1028 | 1861 | 18.66 | 64.42 |
| ck | Specific Persist | steals | steals | 0.75 | 0.69 | 0.80 | **0.0000** | 5800 | 95 | 132 | 72 | 1.61 | 35.29 |
| bf | Specific Persist | dysph | dysph | 0.54 | 0.49 | 0.59 | **0.0000** | 7133 | 358 | 410 | 164 | 4.78 | 28.57 |
| ck | Specific Persist | worth | worth | 0.58 | 0.54 | 0.63 | **0.0000** | 7155 | 336 | 427 | 192 | 4.49 | 31.02 |
| bf | Specific Persist | dysph | dysph | 0.58 | 0.51 | 0.65 | **0.0000** | 4248 | 184 | 188 | 84 | 4.15 | 30.88 |
| ck | Specific Persist | attend | attend | 0.74 | 0.72 | 0.76 | **0.0000** | 3763 | 669 | 1267 | 2411 | 15.09 | 65.55 |
| bf | Specific Persist | dysph | dysph | 0.64 | 0.60 | 0.69 | **0.0000** | 5410 | 309 | 314 | 206 | 5.40 | 39.62 |
| ck | Specific Persist | hyper | hyper | 0.75 | 0.72 | 0.77 | **0.0000** | 5219 | 500 | 1079 | 1312 | 8.74 | 54.87 |
| bf | Specific Persist | dysph | dysph | 0.57 | 0.49 | 0.64 | **0.0000** | 4718 | 177 | 203 | 77 | 3.62 | 27.50 |
| ck | Specific Persist | argues | argues | 0.66 | 0.63 | 0.68 | **0.0000** | 4068 | 827 | 1276 | 1939 | 16.89 | 60.31 |
| bf | Specific Persist | dysph | dysph | 0.57 | 0.52 | 0.62 | **0.0000** | 6058 | 317 | 323 | 154 | 4.97 | 32.29 |
| ck | Specific Persist | temper | temper | 0.66 | 0.63 | 0.69 | **0.0000** | 5842 | 533 | 949 | 786 | 8.36 | 45.30 |
| bf | Specific Persist | dysph | dysph | 0.63 | 0.59 | 0.66 | **0.0000** | 7095 | 562 | 486 | 371 | 7.34 | 43.29 |
| ck | Specific Persist | fights | fights | 0.68 | 0.63 | 0.73 | **0.0000** | 7496 | 161 | 323 | 130 | 2.10 | 28.70 |
| bf | Specific Persist | dysph | dysph | 0.66 | 0.63 | 0.69 | **0.0000** | 7234 | 589 | 507 | 454 | 7.53 | 47.24 |
| ck | Specific Persist | steals | steals | 0.80 | 0.75 | 0.84 | **0.0000** | 7708 | 115 | 171 | 116 | 1.47 | 40.42 |
| bf | Specific Persist | worth | worth | 0.65 | 0.58 | 0.71 | **0.0000** | 4285 | 162 | 167 | 90 | 3.64 | 35.02 |
| ck | Specific Persist | attend | attend | 0.73 | 0.71 | 0.75 | **0.0000** | 3768 | 679 | 1248 | 2370 | 15.27 | 65.51 |
| bf | Specific Persist | worth | worth | 0.66 | 0.62 | 0.71 | **0.0000** | 5421 | 304 | 301 | 213 | 5.31 | 41.44 |
| ck | Specific Persist | hyper | hyper | 0.74 | 0.71 | 0.76 | **0.0000** | 5209 | 516 | 1063 | 1277 | 9.01 | 54.57 |
| bf | Specific Persist | worth | worth | 0.61 | 0.55 | 0.67 | **0.0000** | 4665 | 165 | 244 | 101 | 3.42 | 29.28 |
| ck | Specific Persist | argues | argues | 0.65 | 0.63 | 0.68 | **0.0000** | 4013 | 817 | 1293 | 1942 | 16.92 | 60.03 |
| bf | Specific Persist | worth | worth | 0.63 | 0.58 | 0.67 | **0.0000** | 6013 | 295 | 351 | 193 | 4.68 | 35.48 |
| ck | Specific Persist | temper | temper | 0.65 | 0.62 | 0.68 | **0.0000** | 5767 | 541 | 970 | 787 | 8.58 | 44.79 |
| bf | Specific Persist | worth | worth | 0.68 | 0.65 | 0.72 | **0.0000** | 7053 | 519 | 494 | 448 | 6.85 | 47.56 |
| ck | Specific Persist | fights | fights | 0.72 | 0.67 | 0.76 | **0.0000** | 7405 | 167 | 332 | 161 | 2.21 | 32.66 |
| bf | Specific Persist | worth | worth | 0.70 | 0.67 | 0.73 | **0.0000** | 7220 | 562 | 495 | 507 | 7.22 | 50.60 |
| ck | Specific Persist | steals | steals | 0.78 | 0.74 | 0.82 | **0.0000** | 7658 | 124 | 171 | 112 | 1.59 | 39.58 |
| bf | Specific Persist | attend | attend | 0.63 | 0.60 | 0.66 | **0.0000** | 3816 | 513 | 974 | 936 | 11.85 | 49.01 |
| ck | Specific Persist | hyper | hyper | 0.58 | 0.51 | 0.65 | **0.0000** | 4211 | 118 | 292 | 83 | 2.73 | 22.13 |
| bf | Specific Persist | attend | attend | 0.67 | 0.64 | 0.70 | **0.0000** | 2956 | 364 | 907 | 948 | 10.96 | 51.11 |
| ck | Specific Persist | argues | argues | 0.63 | 0.60 | 0.67 | **0.0000** | 2934 | 386 | 704 | 680 | 11.63 | 49.13 |
| bf | Specific Persist | attend | attend | 0.72 | 0.70 | 0.74 | **0.0000** | 3449 | 551 | 1092 | 1760 | 13.78 | 61.71 |
| ck | Specific Persist | temper | temper | 0.63 | 0.58 | 0.67 | **0.0000** | 3775 | 225 | 455 | 249 | 5.63 | 35.37 |
| bf | Specific Persist | attend | attend | 0.77 | 0.76 | 0.79 | **0.0000** | 3818 | 735 | 1126 | 2835 | 16.14 | 71.57 |
| ck | Specific Persist | fights | fights | 0.67 | 0.58 | 0.76 | **0.0000** | 4502 | 51 | 118 | 33 | 1.12 | 21.85 |
| bf | Specific Persist | attend | attend | 0.78 | 0.76 | 0.79 | **0.0000** | 3859 | 782 | 1127 | 3016 | 16.85 | 72.80 |
| ck | Specific Persist | steals | steals | 0.74 | 0.64 | 0.85 | **0.0000** | 4615 | 26 | 47 | 16 | 0.56 | 25.40 |
| bf | Specific Persist | hyper | hyper | 0.69 | 0.65 | 0.73 | **0.0000** | 3903 | 219 | 641 | 412 | 5.31 | 39.13 |
| ck | Specific Persist | argues | argues | 0.65 | 0.62 | 0.68 | **0.0000** | 3519 | 603 | 931 | 1186 | 14.63 | 56.02 |
| bf | Specific Persist | hyper | hyper | 0.71 | 0.68 | 0.74 | **0.0000** | 4732 | 381 | 912 | 827 | 7.45 | 47.56 |
| ck | Specific Persist | temper | temper | 0.65 | 0.62 | 0.69 | **0.0000** | 4764 | 349 | 666 | 460 | 6.83 | 40.85 |
| bf | Specific Persist | hyper | hyper | 0.76 | 0.74 | 0.78 | **0.0000** | 5405 | 581 | 1048 | 1480 | 9.71 | 58.54 |
| ck | Specific Persist | fights | fights | 0.69 | 0.63 | 0.76 | **0.0000** | 5891 | 95 | 183 | 70 | 1.59 | 27.67 |
| bf | Specific Persist | hyper | hyper | 0.77 | 0.75 | 0.79 | **0.0000** | 5494 | 609 | 1063 | 1618 | 9.98 | 60.35 |
| ck | Specific Persist | steals | steals | 0.79 | 0.73 | 0.85 | **0.0000** | 6055 | 48 | 91 | 45 | 0.79 | 33.09 |
| bf | Specific Persist | argues | argues | 0.58 | 0.55 | 0.61 | **0.0000** | 4040 | 661 | 1101 | 1050 | 14.06 | 48.81 |
| ck | Specific Persist | temper | temper | 0.56 | 0.49 | 0.62 | **0.0000** | 4558 | 143 | 371 | 103 | 3.04 | 21.73 |
| bf | Specific Persist | argues | argues | 0.69 | 0.67 | 0.71 | **0.0000** | 4149 | 927 | 1196 | 2242 | 18.26 | 65.21 |
| ck | Specific Persist | fights | fights | 0.55 | 0.42 | 0.69 | **0.0000** | 5034 | 42 | 86 | 13 | 0.83 | 13.13 |
| bf | Specific Persist | argues | argues | 0.71 | 0.69 | 0.73 | **0.0000** | 4154 | 942 | 1173 | 2515 | 18.49 | 68.19 |
| ck | Specific Persist | steals | steals | 0.75 | 0.66 | 0.85 | **0.0000** | 5068 | 28 | 59 | 20 | 0.55 | 25.32 |
| bf | Specific Persist | temper | temper | 0.65 | 0.63 | 0.68 | **0.0000** | 6044 | 631 | 964 | 875 | 9.45 | 47.58 |
| ck | Specific Persist | fights | fights | 0.57 | 0.47 | 0.67 | **0.0000** | 6610 | 65 | 151 | 26 | 0.97 | 14.69 |
| bf | Specific Persist | temper | temper | 0.71 | 0.69 | 0.74 | **0.0000** | 6042 | 651 | 953 | 1138 | 9.73 | 54.42 |
| ck | Specific Persist | steals | steals | 0.76 | 0.70 | 0.82 | **0.0000** | 6624 | 69 | 107 | 52 | 1.03 | 32.70 |
| bf | Specific Persist | fights | fights | 0.74 | 0.70 | 0.77 | **0.0000** | 8021 | 211 | 345 | 207 | 2.56 | 37.50 |
| ck | Specific Persist | steals | steals | 0.78 | 0.73 | 0.82 | **0.0000** | 8098 | 134 | 170 | 112 | 1.63 | 39.72 |

| Supplemental Table S3B. Tetrachoric correlations from 4 x 4 pairwise analyses of all 90 combinations of the 10 selected psychological problems with every other problem to quantify the associations of each baseline problem with the same problem assessed in the **first annual follow-up** in the **specific persist path** defined by only x_1_ at baseline and only x_2_ at follow-up for problems dichotomized at the **high rating cut (0 or 1 vs 2).** r_t_ = tetrachoric correlation. –CL and +CL = lower and upper 95% confidence intervals for the tetrachoric correlation. c00 = number without the predictor or the outcome; c01 = number without predictor with the outcome; c10 = number with the predictor without the outcome; c11 = number with the predictor and the outcome. Base% = percent of the outcome only x_2_ among children with neither x_1_ nor the other member of the pair, y_1_, at baseline. Obs% = Observed percent of specific persistence (i.e., only x_2_ among children with only x_1_ at baseline). Raw P values significant after FDR adjustment in **bold**. | | | | | | | | | | | | | |
| --- | --- | --- | --- | --- | --- | --- | --- | --- | --- | --- | --- | --- | --- |
| prefix | Path | Predictor | Outcome | r_t_ | -CL | +CL | P | c00 | c01 | c10 | c11 | Base% | Obs% |
| bf | Specific Persist | Fear | fear | 0.51 | 0.39 | 0.64 | **0.0000** | 8640 | 79 | 81 | 13 | 0.91 | 13.83 |
| ck | Specific Persist | worry | worry | 0.56 | 0.48 | 0.63 | **0.0000** | 8537 | 182 | 205 | 57 | 2.09 | 21.76 |
| bf | Specific Persist | fear | fear | 0.64 | 0.57 | 0.71 | **0.0000** | 8767 | 169 | 149 | 60 | 1.89 | 28.71 |
| ck | Specific Persist | dysph | dysph | 0.78 | 0.68 | 0.87 | **0.0000** | 8899 | 37 | 30 | 15 | 0.41 | 33.33 |
| bf | Specific Persist | fear | fear | 0.65 | 0.58 | 0.71 | **0.0000** | 8752 | 172 | 140 | 61 | 1.93 | 30.35 |
| ck | Specific Persist | worth | worth | 0.58 | 0.43 | 0.73 | **0.0000** | 8882 | 42 | 49 | 8 | 0.47 | 14.04 |
| bf | Specific Persist | fear | fear | 0.70 | 0.62 | 0.77 | **0.0000** | 7879 | 93 | 85 | 37 | 1.17 | 30.33 |
| ck | Specific Persist | attend | attend | 0.77 | 0.74 | 0.80 | **0.0000** | 7618 | 354 | 518 | 491 | 4.44 | 48.66 |
| bf | Specific Persist | Fear | fear | 0.67 | 0.59 | 0.74 | **0.0000** | 8267 | 138 | 104 | 47 | 1.64 | 31.13 |
| ck | Specific Persist | Hyper | hyper | 0.77 | 0.74 | 0.81 | **0.0000** | 8201 | 204 | 338 | 238 | 2.43 | 41.32 |
| bf | Specific Persist | Fear | fear | 0.69 | 0.62 | 0.76 | **0.0000** | 8118 | 126 | 103 | 49 | 1.53 | 32.24 |
| ck | Specific Persist | argues | argues | 0.73 | 0.70 | 0.76 | **0.0000** | 7909 | 335 | 414 | 323 | 4.06 | 43.83 |
| bf | Specific Persist | fear | fear | 0.64 | 0.57 | 0.71 | **0.0000** | 8533 | 142 | 125 | 48 | 1.64 | 27.75 |
| ck | Specific Persist | temper | temper | 0.72 | 0.67 | 0.77 | **0.0000** | 8520 | 155 | 201 | 105 | 1.79 | 34.31 |
| bf | Specific Persist | fear | fear | 0.67 | 0.61 | 0.73 | **0.0000** | 8756 | 190 | 151 | 76 | 2.12 | 33.48 |
| ck | Specific Persist | fights | fights | 0.59 | 0.40 | 0.79 | **0.0000** | 8923 | 23 | 31 | 4 | 0.26 | 11.43 |
| bf | Specific Persist | fear | fear | 0.66 | 0.60 | 0.72 | **0.0000** | 8748 | 192 | 156 | 75 | 2.15 | 32.47 |
| ck | Specific Persist | steals | steals | 0.75 | 0.64 | 0.87 | **0.0000** | 8912 | 28 | 30 | 11 | 0.31 | 26.83 |
| bf | Specific Persist | worry | worry | 0.67 | 0.63 | 0.72 | **0.0000** | 8532 | 246 | 237 | 130 | 2.80 | 35.42 |
| ck | Specific Persist | dysph | dysph | 0.72 | 0.59 | 0.85 | **0.0000** | 8747 | 31 | 26 | 9 | 0.35 | 25.71 |
| bf | Specific Persist | worry | worry | 0.68 | 0.63 | 0.73 | **0.0000** | 8525 | 245 | 228 | 127 | 2.79 | 35.77 |
| ck | Specific Persist | worth | worth | 0.57 | 0.39 | 0.75 | **0.0000** | 8740 | 30 | 38 | 5 | 0.34 | 11.63 |
| bf | Specific Persist | worry | worry | 0.66 | 0.60 | 0.72 | **0.0000** | 7692 | 160 | 169 | 73 | 2.04 | 30.17 |
| ck | Specific Persist | attend | attend | 0.78 | 0.75 | 0.80 | **0.0000** | 7522 | 330 | 493 | 468 | 4.20 | 48.70 |
| bf | Specific Persist | worry | worry | 0.68 | 0.63 | 0.73 | **0.0000** | 8045 | 209 | 196 | 106 | 2.53 | 35.10 |
| ck | Specific Persist | hyper | hyper | 0.78 | 0.75 | 0.81 | **0.0000** | 8065 | 189 | 328 | 231 | 2.29 | 41.32 |
| bf | Specific Persist | worry | worry | 0.68 | 0.63 | 0.74 | **0.0000** | 7935 | 181 | 186 | 94 | 2.23 | 33.57 |
| ck | Specific Persist | argues | argues | 0.73 | 0.69 | 0.76 | **0.0000** | 7799 | 317 | 398 | 299 | 3.91 | 42.90 |
| bf | Specific Persist | worry | worry | 0.68 | 0.63 | 0.73 | **0.0000** | 8309 | 216 | 209 | 114 | 2.53 | 35.29 |
| ck | Specific Persist | temper | temper | 0.69 | 0.63 | 0.74 | **0.0000** | 8367 | 158 | 198 | 90 | 1.85 | 31.25 |
| bf | Specific Persist | worry | worry | 0.71 | 0.67 | 0.75 | **0.0000** | 8514 | 267 | 232 | 160 | 3.04 | 40.82 |
| ck | Specific Persist | fights | fights | 0.65 | 0.47 | 0.82 | **0.0000** | 8758 | 23 | 27 | 5 | 0.26 | 15.63 |
| bf | Specific Persist | worry | worry | 0.71 | 0.67 | 0.75 | **0.0000** | 8509 | 264 | 237 | 161 | 3.01 | 40.45 |
| ck | Specific Persist | steals | steals | 0.77 | 0.66 | 0.88 | **0.0000** | 8748 | 25 | 29 | 11 | 0.28 | 27.50 |
| bf | Specific Persist | dysph | dysph | 0.71 | 0.59 | 0.82 | **0.0000** | 9030 | 44 | 38 | 13 | 0.48 | 25.49 |
| ck | Specific Persist | worth | worth | 0.62 | 0.49 | 0.75 | **0.0000** | 9031 | 43 | 60 | 11 | 0.47 | 15.49 |
| bf | Specific Persist | dysph | dysph | 0.81 | 0.71 | 0.91 | **0.0000** | 8036 | 25 | 21 | 12 | 0.31 | 36.36 |
| ck | Specific Persist | attend | attend | 0.79 | 0.76 | 0.81 | **0.0000** | 7679 | 382 | 518 | 566 | 4.74 | 52.21 |
| bf | Specific Persist | dysph | dysph | 0.76 | 0.66 | 0.85 | **0.0000** | 8464 | 42 | 34 | 16 | 0.49 | 32.00 |
| ck | Specific Persist | hyper | hyper | 0.79 | 0.76 | 0.82 | **0.0000** | 8291 | 215 | 357 | 282 | 2.53 | 44.13 |
| bf | Specific Persist | dysph | dysph | 0.76 | 0.64 | 0.89 | **0.0000** | 8340 | 26 | 21 | 9 | 0.31 | 30.00 |
| ck | Specific Persist | argues | argues | 0.74 | 0.71 | 0.77 | **0.0000** | 8010 | 356 | 421 | 358 | 4.26 | 45.96 |
| bf | Specific Persist | dysph | dysph | 0.74 | 0.62 | 0.85 | **0.0000** | 8774 | 37 | 26 | 11 | 0.42 | 29.73 |
| ck | Specific Persist | temper | temper | 0.71 | 0.67 | 0.76 | **0.0000** | 8633 | 178 | 218 | 116 | 2.02 | 34.73 |
| bf | Specific Persist | dysph | dysph | 0.73 | 0.64 | 0.82 | **0.0000** | 9046 | 60 | 46 | 21 | 0.66 | 31.34 |
| ck | Specific Persist | fights | fights | 0.68 | 0.52 | 0.83 | **0.0000** | 9086 | 20 | 33 | 6 | 0.22 | 15.38 |
| bf | Specific Persist | dysph | dysph | 0.76 | 0.68 | 0.84 | **0.0000** | 9042 | 62 | 43 | 24 | 0.68 | 35.82 |
| ck | Specific Persist | steals | steals | 0.74 | 0.63 | 0.86 | **0.0000** | 9073 | 31 | 30 | 11 | 0.34 | 26.83 |
| bf | Specific Persist | worth | worth | 0.74 | 0.62 | 0.86 | **0.0000** | 8027 | 25 | 32 | 10 | 0.31 | 23.81 |
| ck | Specific Persist | attend | attend | 0.79 | 0.77 | 0.82 | **0.0000** | 7668 | 384 | 503 | 570 | 4.77 | 53.12 |
| bf | Specific Persist | worth | worth | 0.66 | 0.54 | 0.77 | **0.0000** | 8445 | 45 | 53 | 13 | 0.53 | 19.70 |
| ck | Specific Persist | hyper | hyper | 0.79 | 0.76 | 0.82 | **0.0000** | 8274 | 216 | 350 | 285 | 2.54 | 44.88 |
| bf | Specific Persist | worth | worth | 0.68 | 0.55 | 0.82 | **0.0000** | 8320 | 29 | 38 | 9 | 0.35 | 19.15 |
| ck | Specific Persist | argues | argues | 0.74 | 0.71 | 0.77 | **0.0000** | 7989 | 360 | 417 | 359 | 4.31 | 46.26 |
| bf | Specific Persist | worth | worth | 0.64 | 0.51 | 0.77 | **0.0000** | 8752 | 39 | 47 | 10 | 0.44 | 17.54 |
| ck | Specific Persist | temper | temper | 0.71 | 0.66 | 0.76 | **0.0000** | 8608 | 183 | 218 | 116 | 2.08 | 34.73 |
| bf | Specific Persist | worth | worth | 0.67 | 0.58 | 0.77 | **0.0000** | 9025 | 61 | 67 | 20 | 0.67 | 22.99 |
| ck | Specific Persist | fights | fights | 0.66 | 0.50 | 0.82 | **0.0000** | 9063 | 23 | 33 | 6 | 0.25 | 15.38 |
| bf | Specific Persist | worth | worth | 0.67 | 0.57 | 0.77 | **0.0000** | 9020 | 63 | 68 | 20 | 0.69 | 22.73 |
| ck | Specific Persist | steals | steals | 0.78 | 0.68 | 0.88 | **0.0000** | 9053 | 30 | 29 | 13 | 0.33 | 30.95 |
| bf | Specific Persist | attend | attend | 0.67 | 0.63 | 0.71 | **0.0000** | 7665 | 268 | 408 | 215 | 3.38 | 34.51 |
| ck | Specific Persist | hyper | hyper | 0.66 | 0.57 | 0.75 | **0.0000** | 7884 | 49 | 134 | 27 | 0.62 | 16.77 |
| bf | Specific Persist | attend | attend | 0.76 | 0.73 | 0.79 | **0.0000** | 7343 | 275 | 433 | 345 | 3.61 | 44.34 |
| ck | Specific Persist | argues | argues | 0.66 | 0.62 | 0.71 | **0.0000** | 7385 | 233 | 318 | 158 | 3.06 | 33.19 |
| bf | Specific Persist | attend | attend | 0.77 | 0.74 | 0.80 | **0.0000** | 7572 | 334 | 488 | 454 | 4.22 | 48.20 |
| ck | Specific Persist | temper | temper | 0.67 | 0.60 | 0.74 | **0.0000** | 7802 | 104 | 138 | 50 | 1.32 | 26.60 |
| bf | Specific Persist | attend | attend | 0.80 | 0.78 | 0.82 | **0.0000** | 7691 | 388 | 498 | 596 | 4.80 | 54.48 |
| ck | Specific Persist | fights | fights | 0.55 | 0.20 | 0.90 | **0.0019** | 8070 | 9 | 14 | 1 | 0.11 | 6.67 |
| bf | Specific Persist | attend | attend | 0.79 | 0.77 | 0.81 | **0.0000** | 7683 | 394 | 509 | 585 | 4.88 | 53.47 |
| ck | Specific Persist | steals | steals | 0.78 | 0.63 | 0.93 | **0.0000** | 8062 | 15 | 12 | 5 | 0.19 | 29.41 |
| bf | Specific Persist | hyper | hyper | 0.75 | 0.71 | 0.80 | **0.0000** | 7840 | 134 | 279 | 143 | 1.68 | 33.89 |
| ck | Specific Persist | argues | argues | 0.71 | 0.68 | 0.75 | **0.0000** | 7701 | 273 | 350 | 232 | 3.42 | 39.86 |
| bf | Specific Persist | hyper | hyper | 0.78 | 0.75 | 0.82 | **0.0000** | 8147 | 170 | 320 | 211 | 2.04 | 39.74 |
| ck | Specific Persist | temper | temper | 0.69 | 0.63 | 0.75 | **0.0000** | 8191 | 126 | 170 | 69 | 1.51 | 28.87 |
| bf | Specific Persist | hyper | hyper | 0.80 | 0.77 | 0.83 | **0.0000** | 8315 | 217 | 345 | 296 | 2.54 | 46.18 |
| ck | Specific Persist | fights | fights | 0.67 | 0.46 | 0.88 | **0.0000** | 8521 | 11 | 21 | 3 | 0.13 | 12.50 |
| bf | Specific Persist | hyper | hyper | 0.80 | 0.78 | 0.83 | **0.0000** | 8311 | 219 | 343 | 298 | 2.57 | 46.49 |
| ck | Specific Persist | steals | steals | 0.77 | 0.64 | 0.90 | **0.0000** | 8512 | 18 | 19 | 7 | 0.21 | 26.92 |
| bf | Specific Persist | argues | argues | 0.68 | 0.64 | 0.72 | **0.0000** | 7986 | 271 | 384 | 207 | 3.28 | 35.03 |
| ck | Specific Persist | temper | temper | 0.57 | 0.47 | 0.68 | **0.0000** | 8192 | 65 | 119 | 20 | 0.79 | 14.39 |
| bf | Specific Persist | argues | argues | 0.75 | 0.72 | 0.78 | **0.0000** | 8019 | 364 | 416 | 374 | 4.34 | 47.34 |
| ck | Specific Persist | fights | fights | . | . | . | . | 8377 | 6 | 13 | 0 | 0.07 | 0.00 |
| bf | Specific Persist | argues | argues | 0.75 | 0.72 | 0.78 | **0.0000** | 8013 | 360 | 421 | 377 | 4.30 | 47.24 |
| ck | Specific Persist | steals | steals | 0.81 | 0.67 | 0.96 | **0.0000** | 8366 | 7 | 18 | 5 | 0.08 | 21.74 |
| bf | Specific Persist | temper | temper | 0.71 | 0.67 | 0.76 | **0.0000** | 8643 | 187 | 222 | 121 | 2.12 | 35.28 |
| ck | Specific Persist | fights | fights | . | . | . | . | 8820 | 10 | 18 | 0 | 0.11 | 0.00 |
| bf | Specific Persist | temper | temper | 0.72 | 0.68 | 0.77 | **0.0000** | 8633 | 183 | 228 | 127 | 2.08 | 35.77 |
| ck | Specific Persist | steals | steals | 0.70 | 0.53 | 0.87 | **0.0000** | 8801 | 15 | 27 | 5 | 0.17 | 15.63 |
| bf | Specific Persist | fights | fights | 0.61 | 0.44 | 0.79 | **0.0000** | 9107 | 24 | 35 | 5 | 0.26 | 12.50 |
| ck | Specific Persist | steals | steals | 0.76 | 0.65 | 0.87 | **0.0000** | 9100 | 31 | 30 | 12 | 0.34 | 28.57 |

| Supplemental Table S3C. Tetrachoric correlations from 4 x 4 pairwise analyses of all 90 combinations of the 10 selected psychological problems with every other problem to quantify the associations of each baseline problem with the same problem assessed in the **second annual follow-up** in the **specific persist path** defined by only x1 at baseline and only x2 at follow-up for problems dichotomized at the **low rating cut (0 vs 1 or 2)**. rt = tetrachoric correlation. –CL and +CL = lower and upper 95% confidence intervals for the tetrachoric correlation. c00 = number without the predictor or the outcome; c01 = number without predictor with the outcome; c10 = number with the predictor without the outcome; c11 = number with the predictor and the outcome. Base% = percent of the outcome only x2 among children with neither x1 nor the other member of the pair, y1, at baseline. Obs% = Observed percent of specific persistence (i.e., only x2 among children with only x1 at baseline). Raw P values significant after FDR adjustment in **bold**. | | | | | | | | | | | | | |
| --- | --- | --- | --- | --- | --- | --- | --- | --- | --- | --- | --- | --- | --- |
| prefix | Path | Predictor | Outcome | r_t_ | -CL | +CL | P | c00 | c01 | c10 | c11 | Base% | Obs% |
| bf | Specific Persist | Fear | fear | 0.44 | 0.37 | 0.51 | **0.0000** | 5212 | 180 | 454 | 87 | 3.34 | 16.08 |
| ck | Specific Persist | Worry | worry | 0.44 | 0.40 | 0.48 | **0.0000** | 4786 | 606 | 1088 | 532 | 11.24 | 32.84 |
| bf | Specific Persist | Fear | fear | 0.58 | 0.55 | 0.62 | **0.0000** | 6020 | 482 | 893 | 511 | 7.41 | 36.40 |
| ck | Specific Persist | Dysph | dysph | 0.44 | 0.38 | 0.50 | **0.0000** | 6126 | 376 | 385 | 125 | 5.78 | 24.51 |
| bf | Specific Persist | Fear | fear | 0.56 | 0.52 | 0.60 | **0.0000** | 5963 | 526 | 870 | 495 | 8.11 | 36.26 |
| ck | Specific Persist | worth | worth | 0.55 | 0.50 | 0.61 | **0.0000** | 6217 | 272 | 380 | 143 | 4.19 | 27.34 |
| bf | Specific Persist | fear | fear | 0.60 | 0.55 | 0.65 | **0.0000** | 3719 | 239 | 409 | 217 | 6.04 | 34.66 |
| ck | Specific Persist | attend | attend | 0.67 | 0.64 | 0.69 | **0.0000** | 3361 | 597 | 1274 | 1780 | 15.08 | 58.28 |
| bf | Specific Persist | fear | fear | 0.61 | 0.57 | 0.65 | **0.0000** | 4641 | 435 | 575 | 419 | 8.57 | 42.15 |
| ck | Specific Persist | hyper | hyper | 0.69 | 0.66 | 0.72 | **0.0000** | 4730 | 346 | 1106 | 830 | 6.82 | 42.87 |
| bf | Specific Persist | fear | fear | 0.57 | 0.53 | 0.62 | **0.0000** | 4017 | 272 | 505 | 250 | 6.34 | 33.11 |
| ck | Specific Persist | argues | argues | 0.61 | 0.58 | 0.64 | **0.0000** | 3577 | 712 | 1211 | 1512 | 16.60 | 55.53 |
| bf | Specific Persist | fear | fear | 0.60 | 0.57 | 0.64 | **0.0000** | 5120 | 466 | 654 | 454 | 8.34 | 40.97 |
| ck | Specific Persist | temper | temper | 0.62 | 0.59 | 0.66 | **0.0000** | 5199 | 387 | 883 | 543 | 6.93 | 38.08 |
| bf | Specific Persist | fear | fear | 0.64 | 0.61 | 0.67 | **0.0000** | 5895 | 702 | 859 | 845 | 10.64 | 49.59 |
| ck | Specific Persist | fights | fights | 0.60 | 0.54 | 0.66 | **0.0000** | 6469 | 128 | 329 | 86 | 1.94 | 20.72 |
| bf | Specific Persist | fear | fear | 0.65 | 0.62 | 0.67 | **0.0000** | 6021 | 735 | 900 | 907 | 10.88 | 50.19 |
| ck | Specific Persist | steals | steals | 0.70 | 0.64 | 0.77 | **0.0000** | 6656 | 100 | 183 | 73 | 1.48 | 28.52 |
| bf | Specific Persist | worry | worry | 0.52 | 0.48 | 0.55 | **0.0000** | 4868 | 758 | 1313 | 967 | 13.47 | 42.41 |
| ck | Specific Persist | dysph | dysph | 0.41 | 0.33 | 0.49 | **0.0000** | 5402 | 224 | 250 | 57 | 3.98 | 18.57 |
| bf | Specific Persist | worry | worry | 0.50 | 0.47 | 0.53 | **0.0000** | 4785 | 803 | 1294 | 972 | 14.37 | 42.89 |
| ck | Specific Persist | worth | worth | 0.49 | 0.41 | 0.56 | **0.0000** | 5416 | 172 | 279 | 66 | 3.08 | 19.13 |
| bf | Specific Persist | worry | worry | 0.55 | 0.51 | 0.59 | **0.0000** | 3005 | 381 | 702 | 496 | 11.25 | 41.40 |
| ck | Specific Persist | attend | attend | 0.63 | 0.60 | 0.66 | **0.0000** | 2923 | 463 | 1211 | 1336 | 13.67 | 52.45 |
| bf | Specific Persist | worry | worry | 0.56 | 0.53 | 0.60 | **0.0000** | 3668 | 639 | 902 | 861 | 14.84 | 48.84 |
| ck | Specific Persist | hyper | hyper | 0.68 | 0.65 | 0.72 | **0.0000** | 4047 | 260 | 972 | 654 | 6.04 | 40.22 |
| bf | Specific Persist | worry | worry | 0.53 | 0.49 | 0.57 | **0.0000** | 3312 | 447 | 760 | 525 | 11.89 | 40.86 |
| ck | Specific Persist | argues | argues | 0.58 | 0.55 | 0.61 | **0.0000** | 3203 | 556 | 1094 | 1080 | 14.79 | 49.68 |
| bf | Specific Persist | worry | worry | 0.56 | 0.53 | 0.60 | **0.0000** | 4084 | 711 | 970 | 929 | 14.83 | 48.92 |
| ck | Specific Persist | temper | temper | 0.60 | 0.56 | 0.64 | **0.0000** | 4495 | 300 | 754 | 384 | 6.26 | 33.74 |
| bf | Specific Persist | worry | worry | 0.60 | 0.57 | 0.63 | **0.0000** | 4627 | 977 | 1183 | 1514 | 17.43 | 56.14 |
| ck | Specific Persist | fights | fights | 0.57 | 0.49 | 0.65 | **0.0000** | 5504 | 100 | 270 | 59 | 1.78 | 17.93 |
| bf | Specific Persist | worry | worry | 0.60 | 0.58 | 0.63 | **0.0000** | 4731 | 1016 | 1215 | 1601 | 17.68 | 56.85 |
| ck | Specific Persist | steals | steals | 0.70 | 0.62 | 0.77 | **0.0000** | 5668 | 79 | 135 | 51 | 1.37 | 27.42 |
| bf | Specific Persist | dysph | dysph | 0.41 | 0.35 | 0.47 | **0.0000** | 6842 | 457 | 422 | 133 | 6.26 | 23.96 |
| ck | Specific Persist | worth | worth | 0.51 | 0.45 | 0.56 | **0.0000** | 6975 | 324 | 457 | 150 | 4.44 | 24.71 |
| bf | Specific Persist | dysph | dysph | 0.49 | 0.41 | 0.57 | **0.0000** | 4117 | 201 | 198 | 68 | 4.65 | 25.56 |
| ck | Specific Persist | attend | attend | 0.66 | 0.64 | 0.69 | **0.0000** | 3628 | 690 | 1454 | 2134 | 15.98 | 59.48 |
| bf | Specific Persist | dysph | dysph | 0.49 | 0.43 | 0.55 | **0.0000** | 5142 | 428 | 335 | 165 | 7.68 | 33.00 |
| ck | Specific Persist | hyper | hyper | 0.69 | 0.66 | 0.71 | **0.0000** | 5151 | 419 | 1293 | 1043 | 7.52 | 44.65 |
| bf | Specific Persist | dysph | dysph | 0.43 | 0.35 | 0.51 | **0.0000** | 4551 | 220 | 214 | 59 | 4.61 | 21.61 |
| ck | Specific Persist | argues | argues | 0.59 | 0.57 | 0.62 | **0.0000** | 3964 | 807 | 1430 | 1705 | 16.91 | 54.39 |
| bf | Specific Persist | dysph | dysph | 0.46 | 0.40 | 0.52 | **0.0000** | 5800 | 427 | 330 | 137 | 6.86 | 29.34 |
| ck | Specific Persist | temper | temper | 0.61 | 0.57 | 0.64 | **0.0000** | 5791 | 436 | 1068 | 611 | 7.00 | 36.39 |
| bf | Specific Persist | dysph | dysph | 0.54 | 0.50 | 0.58 | **0.0000** | 6763 | 713 | 488 | 337 | 9.54 | 40.85 |
| ck | Specific Persist | fights | fights | 0.61 | 0.55 | 0.67 | **0.0000** | 7333 | 143 | 338 | 92 | 1.91 | 21.40 |
| bf | Specific Persist | dysph | dysph | 0.56 | 0.52 | 0.60 | **0.0000** | 6913 | 721 | 536 | 393 | 9.44 | 42.30 |
| ck | Specific Persist | steals | steals | 0.70 | 0.64 | 0.76 | **0.0000** | 7541 | 93 | 202 | 70 | 1.22 | 25.74 |
| bf | Specific Persist | worth | worth | 0.60 | 0.53 | 0.67 | **0.0000** | 4165 | 170 | 170 | 79 | 3.92 | 31.73 |
| ck | Specific Persist | attend | attend | 0.67 | 0.65 | 0.69 | **0.0000** | 3614 | 721 | 1368 | 2151 | 16.63 | 61.13 |
| bf | Specific Persist | worth | worth | 0.60 | 0.55 | 0.65 | **0.0000** | 5246 | 326 | 313 | 185 | 5.85 | 37.15 |
| ck | Specific Persist | hyper | hyper | 0.69 | 0.66 | 0.71 | **0.0000** | 5148 | 424 | 1250 | 1032 | 7.61 | 45.22 |
| bf | Specific Persist | worth | worth | 0.51 | 0.44 | 0.58 | **0.0000** | 4500 | 201 | 258 | 85 | 4.28 | 24.78 |
| ck | Specific Persist | argues | argues | 0.61 | 0.59 | 0.64 | **0.0000** | 3879 | 822 | 1357 | 1796 | 17.49 | 56.96 |
| bf | Specific Persist | worth | worth | 0.55 | 0.50 | 0.61 | **0.0000** | 5818 | 342 | 363 | 171 | 5.55 | 32.02 |
| ck | Specific Persist | temper | temper | 0.63 | 0.60 | 0.66 | **0.0000** | 5731 | 429 | 1037 | 657 | 6.96 | 38.78 |
| bf | Specific Persist | worth | worth | 0.60 | 0.56 | 0.64 | **0.0000** | 6810 | 573 | 539 | 379 | 7.76 | 41.29 |
| ck | Specific Persist | fights | fights | 0.64 | 0.58 | 0.69 | **0.0000** | 7235 | 148 | 359 | 112 | 2.00 | 23.78 |
| bf | Specific Persist | worth | worth | 0.63 | 0.59 | 0.66 | **0.0000** | 6997 | 590 | 547 | 429 | 7.78 | 43.95 |
| ck | Specific Persist | steals | steals | 0.68 | 0.62 | 0.74 | **0.0000** | 7471 | 116 | 194 | 73 | 1.53 | 27.34 |
| bf | Specific Persist | attend | attend | 0.57 | 0.54 | 0.60 | **0.0000** | 3622 | 597 | 961 | 891 | 14.15 | 48.11 |
| ck | Specific Persist | hyper | hyper | 0.50 | 0.41 | 0.58 | **0.0000** | 4120 | 99 | 309 | 57 | 2.35 | 15.57 |
| bf | Specific Persist | attend | attend | 0.60 | 0.57 | 0.64 | **0.0000** | 2838 | 399 | 957 | 850 | 12.33 | 47.04 |
| ck | Specific Persist | argues | argues | 0.55 | 0.51 | 0.59 | **0.0000** | 2843 | 394 | 773 | 575 | 12.17 | 42.66 |
| bf | Specific Persist | attend | attend | 0.67 | 0.64 | 0.69 | **0.0000** | 3296 | 607 | 1136 | 1655 | 15.55 | 59.30 |
| ck | Specific Persist | temper | temper | 0.60 | 0.55 | 0.65 | **0.0000** | 3733 | 170 | 487 | 194 | 4.36 | 28.49 |
| bf | Specific Persist | attend | attend | 0.72 | 0.70 | 0.74 | **0.0000** | 3634 | 806 | 1228 | 2633 | 18.15 | 68.19 |
| ck | Specific Persist | fights | fights | 0.60 | 0.50 | 0.71 | **0.0000** | 4393 | 47 | 120 | 24 | 1.06 | 16.67 |
| bf | Specific Persist | attend | attend | 0.73 | 0.71 | 0.75 | **0.0000** | 3698 | 827 | 1238 | 2800 | 18.28 | 69.34 |
| ck | Specific Persist | steals | steals | 0.65 | 0.51 | 0.80 | **0.0000** | 4501 | 24 | 49 | 10 | 0.53 | 16.95 |
| bf | Specific Persist | hyper | hyper | 0.63 | 0.59 | 0.67 | **0.0000** | 3839 | 181 | 711 | 313 | 4.50 | 30.57 |
| ck | Specific Persist | argues | argues | 0.60 | 0.56 | 0.63 | **0.0000** | 3383 | 637 | 962 | 1089 | 15.85 | 53.10 |
| bf | Specific Persist | hyper | hyper | 0.66 | 0.63 | 0.69 | **0.0000** | 4651 | 341 | 1017 | 685 | 6.83 | 40.25 |
| ck | Specific Persist | temper | temper | 0.62 | 0.58 | 0.66 | **0.0000** | 4696 | 296 | 698 | 380 | 5.93 | 35.25 |
| bf | Specific Persist | hyper | hyper | 0.71 | 0.68 | 0.73 | **0.0000** | 5312 | 512 | 1234 | 1243 | 8.79 | 50.18 |
| ck | Specific Persist | fights | fights | 0.62 | 0.54 | 0.70 | **0.0000** | 5739 | 85 | 196 | 50 | 1.46 | 20.33 |
| bf | Specific Persist | hyper | hyper | 0.72 | 0.70 | 0.74 | **0.0000** | 5421 | 521 | 1265 | 1356 | 8.77 | 51.74 |
| ck | Specific Persist | steals | steals | 0.70 | 0.61 | 0.78 | **0.0000** | 5888 | 54 | 97 | 31 | 0.91 | 24.22 |
| bf | Specific Persist | argues | argues | 0.53 | 0.50 | 0.57 | **0.0000** | 3890 | 700 | 1123 | 981 | 15.25 | 46.63 |
| ck | Specific Persist | temper | temper | 0.46 | 0.38 | 0.54 | **0.0000** | 4489 | 101 | 396 | 58 | 2.20 | 12.78 |
| bf | Specific Persist | argues | argues | 0.64 | 0.61 | 0.66 | **0.0000** | 3999 | 948 | 1281 | 2073 | 19.16 | 61.81 |
| ck | Specific Persist | fights | fights | 0.44 | 0.24 | 0.64 | **0.0000** | 4925 | 22 | 92 | 5 | 0.44 | 5.15 |
| bf | Specific Persist | argues | argues | 0.67 | 0.64 | 0.69 | **0.0000** | 4010 | 959 | 1275 | 2319 | 19.30 | 64.52 |
| ck | Specific Persist | steals | steals | 0.74 | 0.63 | 0.85 | **0.0000** | 4952 | 17 | 61 | 14 | 0.34 | 18.67 |
| bf | Specific Persist | temper | temper | 0.63 | 0.60 | 0.66 | **0.0000** | 6000 | 520 | 1052 | 729 | 7.98 | 40.93 |
| ck | Specific Persist | fights | fights | 0.58 | 0.48 | 0.68 | **0.0000** | 6465 | 55 | 150 | 24 | 0.84 | 13.79 |
| bf | Specific Persist | temper | temper | 0.69 | 0.66 | 0.71 | **0.0000** | 5999 | 537 | 1067 | 960 | 8.22 | 47.36 |
| ck | Specific Persist | steals | steals | 0.66 | 0.57 | 0.74 | **0.0000** | 6458 | 78 | 121 | 37 | 1.19 | 23.42 |
| bf | Specific Persist | fights | fights | 0.62 | 0.57 | 0.67 | **0.0000** | 7838 | 194 | 398 | 133 | 2.42 | 25.05 |
| ck | Specific Persist | steals | steals | 0.68 | 0.62 | 0.74 | **0.0000** | 7898 | 134 | 192 | 77 | 1.67 | 28.62 |

| Supplemental Table S3D. Tetrachoric correlations from 4 x 4 pairwise analyses of all 90 combinations of the 10 selected psychological problems with every other problem to quantify the associations of each baseline problem with the same problem assessed in the **second annual follow-up** in one of the two homotypic paths (the **specific persist path** defined by only x_1_ at baseline and only x_2_ at follow-up) for problems dichotomized at the **high rating cut (0 or 1 vs 2).** r_t_ = tetrachoric correlation. –CL and +CL = lower and upper 95% confidence intervals for the tetrachoric correlation. c00 = number without the predictor or the outcome; c01 = number without predictor with the outcome; c10 = number with the predictor without the outcome; c11 = number with the predictor and the outcome. Base% = percent of the outcome only x_2_ among children with neither x_1_ nor the other member of the pair, y_1_, at baseline. Obs% = Observed percent of specific persistence (i.e., only x_2_ among children with only x_1_ at baseline). Raw P values significant after FDR adjustment in **bold**. | | | | | | | | | | | | | |
| --- | --- | --- | --- | --- | --- | --- | --- | --- | --- | --- | --- | --- | --- |
| Prefix | Path | Predictor | Outcome | r_t_ | -CL | +CL | P | c00 | c01 | c10 | c11 | Base% | Obs% |
| bf | Specific Persist | Fear | fear | 0.47 | 0.32 | 0.61 | **0.0000** | 8423 | 67 | 78 | 9 | 0.79 | 10.34 |
| ck | Specific Persist | Worry | worry | 0.50 | 0.42 | 0.58 | **0.0000** | 8324 | 166 | 215 | 44 | 1.96 | 16.99 |
| bf | Specific Persist | Fear | fear | 0.63 | 0.55 | 0.70 | **0.0000** | 8573 | 135 | 148 | 49 | 1.55 | 24.87 |
| ck | Specific Persist | Dysph | dysph | 0.58 | 0.42 | 0.74 | **0.0000** | 8657 | 51 | 34 | 7 | 0.59 | 17.07 |
| bf | Specific Persist | Fear | fear | 0.63 | 0.55 | 0.70 | **0.0000** | 8560 | 138 | 142 | 48 | 1.59 | 25.26 |
| ck | Specific Persist | worth | worth | 0.49 | 0.31 | 0.67 | **0.0000** | 8654 | 44 | 46 | 5 | 0.51 | 9.80 |
| bf | Specific Persist | fear | fear | 0.66 | 0.58 | 0.75 | **0.0000** | 7661 | 101 | 84 | 33 | 1.30 | 28.21 |
| ck | Specific Persist | attend | attend | 0.73 | 0.71 | 0.76 | **0.0000** | 7421 | 341 | 554 | 433 | 4.39 | 43.87 |
| bf | Specific Persist | fear | fear | 0.63 | 0.55 | 0.71 | **0.0000** | 8054 | 133 | 106 | 40 | 1.62 | 27.40 |
| ck | Specific Persist | hyper | hyper | 0.74 | 0.70 | 0.78 | **0.0000** | 8025 | 162 | 375 | 187 | 1.98 | 33.27 |
| bf | Specific Persist | fear | fear | 0.66 | 0.58 | 0.73 | **0.0000** | 7933 | 113 | 102 | 39 | 1.40 | 27.66 |
| ck | Specific Persist | argues | argues | 0.68 | 0.64 | 0.72 | **0.0000** | 7663 | 383 | 413 | 290 | 4.76 | 41.25 |
| bf | Specific Persist | fear | fear | 0.61 | 0.53 | 0.69 | **0.0000** | 8321 | 139 | 121 | 40 | 1.64 | 24.84 |
| ck | Specific Persist | temper | temper | 0.59 | 0.51 | 0.66 | **0.0000** | 8322 | 138 | 231 | 58 | 1.63 | 20.07 |
| bf | Specific Persist | fear | fear | 0.66 | 0.59 | 0.72 | **0.0000** | 8552 | 164 | 150 | 65 | 1.88 | 30.23 |
| ck | Specific Persist | fights | fights | 0.71 | 0.57 | 0.86 | **0.0000** | 8693 | 23 | 26 | 7 | 0.26 | 21.21 |
| bf | Specific Persist | fear | fear | 0.67 | 0.60 | 0.73 | **0.0000** | 8550 | 160 | 151 | 67 | 1.84 | 30.73 |
| ck | Specific Persist | steals | steals | 0.75 | 0.63 | 0.86 | **0.0000** | 8678 | 32 | 28 | 11 | 0.37 | 28.21 |
| bf | Specific Persist | worry | worry | 0.58 | 0.52 | 0.64 | **0.0000** | 8323 | 222 | 271 | 89 | 2.60 | 24.72 |
| ck | Specific Persist | dysph | dysph | 0.52 | 0.33 | 0.72 | **0.0000** | 8504 | 41 | 28 | 4 | 0.48 | 12.50 |
| bf | Specific Persist | worry | worry | 0.59 | 0.53 | 0.65 | **0.0000** | 8317 | 221 | 258 | 92 | 2.59 | 26.29 |
| ck | Specific Persist | worth | worth | 0.36 | 0.10 | 0.62 | **0.0062** | 8498 | 40 | 37 | 2 | 0.47 | 5.13 |
| bf | Specific Persist | worry | worry | 0.57 | 0.50 | 0.64 | **0.0000** | 7469 | 169 | 185 | 56 | 2.21 | 23.24 |
| ck | Specific Persist | attend | attend | 0.72 | 0.69 | 0.76 | **0.0000** | 7311 | 327 | 542 | 397 | 4.28 | 42.28 |
| bf | Specific Persist | worry | worry | 0.56 | 0.49 | 0.63 | **0.0000** | 7828 | 205 | 229 | 71 | 2.55 | 23.67 |
| ck | Specific Persist | hyper | hyper | 0.74 | 0.70 | 0.78 | **0.0000** | 7881 | 152 | 367 | 177 | 1.89 | 32.54 |
| bf | Specific Persist | worry | worry | 0.63 | 0.57 | 0.69 | **0.0000** | 7738 | 172 | 200 | 77 | 2.17 | 27.80 |
| ck | Specific Persist | argues | argues | 0.66 | 0.62 | 0.70 | **0.0000** | 7545 | 365 | 410 | 257 | 4.61 | 38.53 |
| bf | Specific Persist | worry | worry | 0.57 | 0.50 | 0.63 | **0.0000** | 8101 | 202 | 243 | 75 | 2.43 | 23.58 |
| ck | Specific Persist | temper | temper | 0.52 | 0.44 | 0.61 | **0.0000** | 8179 | 124 | 232 | 42 | 1.49 | 15.33 |
| bf | Specific Persist | worry | worry | 0.61 | 0.56 | 0.67 | **0.0000** | 8296 | 251 | 271 | 113 | 2.94 | 29.43 |
| ck | Specific Persist | fights | fights | 0.62 | 0.42 | 0.81 | **0.0000** | 8525 | 22 | 26 | 4 | 0.26 | 13.33 |
| bf | Specific Persist | worry | worry | 0.62 | 0.57 | 0.67 | **0.0000** | 8295 | 245 | 273 | 115 | 2.87 | 29.64 |
| ck | Specific Persist | steals | steals | 0.66 | 0.51 | 0.81 | **0.0000** | 8509 | 31 | 30 | 7 | 0.36 | 18.92 |
| bf | Specific Persist | dysph | dysph | 0.53 | 0.37 | 0.69 | **0.0000** | 8781 | 59 | 41 | 7 | 0.67 | 14.58 |
| ck | Specific Persist | worth | worth | 0.33 | 0.11 | 0.55 | **0.0031** | 8789 | 51 | 62 | 3 | 0.58 | 4.62 |
| bf | Specific Persist | dysph | dysph | 0.70 | 0.56 | 0.83 | **0.0000** | 7803 | 46 | 21 | 9 | 0.59 | 30.00 |
| ck | Specific Persist | attend | attend | 0.74 | 0.72 | 0.77 | **0.0000** | 7489 | 360 | 575 | 481 | 4.59 | 45.55 |
| bf | Specific Persist | dysph | dysph | 0.61 | 0.47 | 0.74 | **0.0000** | 8224 | 62 | 37 | 10 | 0.75 | 21.28 |
| ck | Specific Persist | hyper | hyper | 0.76 | 0.72 | 0.79 | **0.0000** | 8114 | 172 | 399 | 220 | 2.08 | 35.54 |
| bf | Specific Persist | dysph | dysph | 0.61 | 0.44 | 0.77 | **0.0000** | 8109 | 50 | 22 | 6 | 0.61 | 21.43 |
| ck | Specific Persist | argues | argues | 0.69 | 0.65 | 0.72 | **0.0000** | 7769 | 390 | 431 | 315 | 4.78 | 42.23 |
| bf | Specific Persist | dysph | dysph | 0.55 | 0.38 | 0.72 | **0.0000** | 8524 | 63 | 28 | 6 | 0.73 | 17.65 |
| ck | Specific Persist | temper | temper | 0.56 | 0.48 | 0.63 | **0.0000** | 8441 | 146 | 260 | 58 | 1.70 | 18.24 |
| bf | Specific Persist | dysph | dysph | 0.60 | 0.48 | 0.72 | **0.0000** | 8786 | 83 | 48 | 14 | 0.94 | 22.58 |
| ck | Specific Persist | fights | fights | 0.64 | 0.46 | 0.81 | **0.0000** | 8847 | 22 | 31 | 5 | 0.25 | 13.89 |
| bf | Specific Persist | dysph | dysph | 0.62 | 0.50 | 0.73 | **0.0000** | 8782 | 84 | 47 | 15 | 0.95 | 24.19 |
| ck | Specific Persist | steals | steals | 0.72 | 0.59 | 0.84 | **0.0000** | 8831 | 35 | 29 | 10 | 0.39 | 25.64 |
| bf | Specific Persist | worth | worth | 0.59 | 0.42 | 0.76 | **0.0000** | 7804 | 37 | 32 | 6 | 0.47 | 15.79 |
| ck | Specific Persist | attend | attend | 0.74 | 0.71 | 0.77 | **0.0000** | 7480 | 361 | 577 | 470 | 4.60 | 44.89 |
| bf | Specific Persist | worth | worth | 0.46 | 0.28 | 0.63 | **0.0000** | 8214 | 58 | 55 | 6 | 0.70 | 9.84 |
| ck | Specific Persist | hyper | hyper | 0.77 | 0.74 | 0.81 | **0.0000** | 8107 | 165 | 389 | 227 | 1.99 | 36.85 |
| bf | Specific Persist | worth | worth | 0.54 | 0.37 | 0.71 | **0.0000** | 8098 | 45 | 38 | 6 | 0.55 | 13.64 |
| ck | Specific Persist | argues | argues | 0.70 | 0.66 | 0.73 | **0.0000** | 7753 | 390 | 425 | 320 | 4.79 | 42.95 |
| bf | Specific Persist | worth | worth | 0.42 | 0.22 | 0.62 | **0.0000** | 8519 | 50 | 48 | 4 | 0.58 | 7.69 |
| ck | Specific Persist | temper | temper | 0.58 | 0.51 | 0.65 | **0.0000** | 8427 | 142 | 257 | 62 | 1.66 | 19.44 |
| bf | Specific Persist | worth | worth | 0.48 | 0.34 | 0.62 | **0.0000** | 8772 | 79 | 70 | 10 | 0.89 | 12.50 |
| ck | Specific Persist | fights | fights | 0.70 | 0.55 | 0.84 | **0.0000** | 8828 | 23 | 30 | 7 | 0.26 | 18.92 |
| bf | Specific Persist | worth | worth | 0.52 | 0.38 | 0.65 | **0.0000** | 8774 | 74 | 69 | 11 | 0.84 | 13.75 |
| ck | Specific Persist | steals | steals | 0.74 | 0.62 | 0.86 | **0.0000** | 8815 | 33 | 29 | 11 | 0.37 | 27.50 |
| bf | Specific Persist | attend | attend | 0.65 | 0.60 | 0.69 | **0.0000** | 7453 | 265 | 418 | 198 | 3.43 | 32.14 |
| ck | Specific Persist | hyper | hyper | 0.60 | 0.48 | 0.71 | **0.0000** | 7683 | 35 | 145 | 17 | 0.45 | 10.49 |
| bf | Specific Persist | attend | attend | 0.73 | 0.69 | 0.76 | **0.0000** | 7188 | 232 | 482 | 286 | 3.13 | 37.24 |
| ck | Specific Persist | argues | argues | 0.62 | 0.57 | 0.67 | **0.0000** | 7177 | 243 | 320 | 140 | 3.27 | 30.43 |
| bf | Specific Persist | attend | attend | 0.73 | 0.70 | 0.76 | **0.0000** | 7385 | 314 | 533 | 389 | 4.08 | 42.19 |
| ck | Specific Persist | temper | temper | 0.62 | 0.53 | 0.71 | **0.0000** | 7629 | 70 | 149 | 31 | 0.91 | 17.22 |
| bf | Specific Persist | attend | attend | 0.75 | 0.72 | 0.78 | **0.0000** | 7495 | 370 | 565 | 501 | 4.70 | 47.00 |
| ck | Specific Persist | fights | fights | 0.72 | 0.48 | 0.97 | **0.0000** | 7859 | 6 | 12 | 2 | 0.08 | 14.29 |
| bf | Specific Persist | attend | attend | 0.75 | 0.72 | 0.78 | **0.0000** | 7501 | 363 | 573 | 491 | 4.62 | 46.15 |
| ck | Specific Persist | steals | steals | 0.74 | 0.54 | 0.94 | **0.0000** | 7854 | 10 | 12 | 3 | 0.13 | 20.00 |
| bf | Specific Persist | hyper | hyper | 0.70 | 0.65 | 0.75 | **0.0000** | 7662 | 107 | 314 | 105 | 1.38 | 25.06 |
| ck | Specific Persist | argues | argues | 0.66 | 0.62 | 0.71 | **0.0000** | 7457 | 312 | 355 | 210 | 4.02 | 37.17 |
| bf | Specific Persist | hyper | hyper | 0.74 | 0.70 | 0.78 | **0.0000** | 7967 | 138 | 354 | 162 | 1.70 | 31.40 |
| ck | Specific Persist | temper | temper | 0.61 | 0.53 | 0.68 | **0.0000** | 7999 | 106 | 183 | 45 | 1.31 | 19.74 |
| bf | Specific Persist | hyper | hyper | 0.76 | 0.72 | 0.79 | **0.0000** | 8135 | 174 | 397 | 225 | 2.09 | 36.17 |
| ck | Specific Persist | fights | fights | 0.69 | 0.48 | 0.90 | **0.0000** | 8300 | 9 | 21 | 3 | 0.11 | 12.50 |
| bf | Specific Persist | hyper | hyper | 0.76 | 0.72 | 0.80 | **0.0000** | 8135 | 174 | 394 | 225 | 2.09 | 36.35 |
| ck | Specific Persist | steals | steals | 0.72 | 0.57 | 0.88 | **0.0000** | 8286 | 23 | 18 | 6 | 0.28 | 25.00 |
| bf | Specific Persist | argues | argues | 0.62 | 0.57 | 0.66 | **0.0000** | 7733 | 318 | 385 | 185 | 3.95 | 32.46 |
| ck | Specific Persist | temper | temper | 0.51 | 0.37 | 0.64 | **0.0000** | 8004 | 47 | 124 | 12 | 0.58 | 8.82 |
| bf | Specific Persist | argues | argues | 0.69 | 0.65 | 0.73 | **0.0000** | 7772 | 404 | 431 | 324 | 4.94 | 42.91 |
| ck | Specific Persist | fights | fights | 0.82 | 0.65 | 1.00 | **0.0000** | 8170 | 6 | 8 | 3 | 0.07 | 27.27 |
| bf | Specific Persist | argues | argues | 0.70 | 0.66 | 0.73 | **0.0000** | 7770 | 395 | 434 | 329 | 4.84 | 43.12 |
| ck | Specific Persist | steals | steals | 0.74 | 0.58 | 0.90 | **0.0000** | 8149 | 16 | 17 | 5 | 0.20 | 22.73 |
| bf | Specific Persist | temper | temper | 0.58 | 0.51 | 0.65 | **0.0000** | 8454 | 150 | 261 | 66 | 1.74 | 20.18 |
| ck | Specific Persist | fights | fights | 0.51 | 0.17 | 0.86 | **0.0036** | 8593 | 11 | 16 | 1 | 0.13 | 5.88 |
| bf | Specific Persist | temper | temper | 0.60 | 0.54 | 0.66 | **0.0000** | 8440 | 149 | 267 | 72 | 1.73 | 21.24 |
| ck | Specific Persist | steals | steals | 0.68 | 0.53 | 0.84 | **0.0000** | 8566 | 23 | 26 | 6 | 0.27 | 18.75 |
| bf | Specific Persist | fights | fights | 0.68 | 0.52 | 0.84 | **0.0000** | 8871 | 20 | 31 | 6 | 0.22 | 16.22 |
| ck | Specific Persist | steals | steals | 0.69 | 0.56 | 0.82 | **0.0000** | 8856 | 35 | 31 | 9 | 0.39 | 22.50 |

4e

| Supplemental Table S3E. Tetrachoric correlations from 4 x 4 pairwise analyses of all 90 combinations of the 10 selected psychological problems with every other problem to quantify the associations of each baseline problem with the same problem assessed in the **third annual follow-up** in one of the two homotypic paths (the **specific persist path** defined by only x_1_ at baseline and only x_2_ at follow-up) for problems dichotomized at the **low rating cut (0 vs 1 or 2).** r_t_ = tetrachoric correlation. –CL and +CL = lower and upper 95% confidence intervals for the tetrachoric correlation. c00 = number without the predictor or the outcome; c01 = number without predictor with the outcome; c10 = number with the predictor without the outcome; c11 = number with the predictor and the outcome. Base% = percent of the outcome only x_2_ among children with neither x_1_ nor the other member of the pair, y_1_, at baseline. Obs% = Observed percent of specific persistence (i.e., only x_2_ among children with only x_1_ at baseline). Raw P values significant after FDR adjustment in **bold**. | | | | | | | | | | | | | |
| --- | --- | --- | --- | --- | --- | --- | --- | --- | --- | --- | --- | --- | --- |
| prefix | Path | Predictor | Outcome | r_t_ | -CL | +CL | P | c00 | c01 | c10 | c11 | Base% | Obs% |
| bf | Specific Persist | fear | fear | 0.41 | 0.34 | 0.49 | **0.0000** | 4795 | 181 | 423 | 80 | 3.64 | 15.90 |
| ck | Specific Persist | worry | worry | 0.38 | 0.34 | 0.43 | **0.0000** | 4402 | 574 | 1057 | 449 | 11.54 | 29.81 |
| bf | Specific Persist | fear | fear | 0.53 | 0.49 | 0.57 | **0.0000** | 5581 | 442 | 892 | 419 | 7.34 | 31.96 |
| ck | Specific Persist | dysph | dysph | 0.36 | 0.30 | 0.43 | **0.0000** | 5549 | 474 | 345 | 114 | 7.87 | 24.84 |
| bf | Specific Persist | fear | fear | 0.52 | 0.48 | 0.56 | **0.0000** | 5472 | 525 | 836 | 443 | 8.75 | 34.64 |
| ck | Specific Persist | worth | worth | 0.45 | 0.39 | 0.52 | **0.0000** | 5713 | 284 | 377 | 107 | 4.74 | 22.11 |
| bf | Specific Persist | fear | fear | 0.55 | 0.50 | 0.61 | **0.0000** | 3422 | 256 | 395 | 200 | 6.96 | 33.61 |
| ck | Specific Persist | attend | attend | 0.63 | 0.60 | 0.65 | **0.0000** | 3076 | 602 | 1225 | 1579 | 16.37 | 56.31 |
| bf | Specific Persist | fear | fear | 0.57 | 0.52 | 0.61 | **0.0000** | 4290 | 429 | 568 | 371 | 9.09 | 39.51 |
| ck | Specific Persist | hyper | hyper | 0.64 | 0.61 | 0.67 | **0.0000** | 4371 | 348 | 1060 | 703 | 7.37 | 39.88 |
| bf | Specific Persist | fear | fear | 0.53 | 0.48 | 0.58 | **0.0000** | 3699 | 259 | 494 | 213 | 6.54 | 30.13 |
| ck | Specific Persist | argues | argues | 0.57 | 0.54 | 0.60 | **0.0000** | 3205 | 753 | 1129 | 1395 | 19.02 | 55.27 |
| bf | Specific Persist | fear | fear | 0.60 | 0.57 | 0.64 | **0.0000** | 4720 | 452 | 601 | 437 | 8.74 | 42.10 |
| ck | Specific Persist | temper | temper | 0.56 | 0.52 | 0.60 | **0.0000** | 4841 | 331 | 905 | 405 | 6.40 | 30.92 |
| bf | Specific Persist | fear | fear | 0.60 | 0.57 | 0.63 | **0.0000** | 5387 | 724 | 827 | 755 | 11.85 | 47.72 |
| ck | Specific Persist | fights | fights | 0.59 | 0.52 | 0.66 | **0.0000** | 6007 | 104 | 302 | 69 | 1.70 | 18.60 |
| bf | Specific Persist | fear | fear | 0.58 | 0.55 | 0.61 | **0.0000** | 5496 | 768 | 899 | 797 | 12.26 | 46.99 |
| ck | Specific Persist | steals | steals | 0.61 | 0.54 | 0.69 | **0.0000** | 6148 | 116 | 166 | 52 | 1.85 | 23.85 |
| bf | Specific Persist | worry | worry | 0.45 | 0.41 | 0.48 | **0.0000** | 4536 | 664 | 1374 | 760 | 12.77 | 35.61 |
| ck | Specific Persist | dysph | dysph | 0.28 | 0.19 | 0.37 | **0.0000** | 4927 | 273 | 237 | 42 | 5.25 | 15.05 |
| bf | Specific Persist | worry | worry | 0.48 | 0.44 | 0.51 | **0.0000** | 4411 | 753 | 1240 | 872 | 14.58 | 41.29 |
| ck | Specific Persist | worth | worth | 0.40 | 0.32 | 0.49 | **0.0000** | 4988 | 176 | 265 | 50 | 3.41 | 15.87 |
| bf | Specific Persist | worry | worry | 0.49 | 0.44 | 0.53 | **0.0000** | 2764 | 385 | 697 | 427 | 12.23 | 37.99 |
| ck | Specific Persist | attend | attend | 0.60 | 0.57 | 0.63 | **0.0000** | 2694 | 455 | 1151 | 1179 | 14.45 | 50.60 |
| bf | Specific Persist | worry | worry | 0.49 | 0.45 | 0.53 | **0.0000** | 3364 | 639 | 917 | 738 | 15.96 | 44.59 |
| ck | Specific Persist | hyper | hyper | 0.63 | 0.59 | 0.66 | **0.0000** | 3726 | 277 | 925 | 551 | 6.92 | 37.33 |
| bf | Specific Persist | worry | worry | 0.41 | 0.36 | 0.46 | **0.0000** | 3068 | 395 | 827 | 375 | 11.41 | 31.20 |
| ck | Specific Persist | argues | argues | 0.54 | 0.50 | 0.57 | **0.0000** | 2880 | 583 | 1024 | 992 | 16.84 | 49.21 |
| bf | Specific Persist | worry | worry | 0.49 | 0.46 | 0.53 | **0.0000** | 3760 | 671 | 1003 | 776 | 15.14 | 43.62 |
| ck | Specific Persist | temper | temper | 0.54 | 0.50 | 0.59 | **0.0000** | 4187 | 244 | 762 | 286 | 5.51 | 27.29 |
| bf | Specific Persist | worry | worry | 0.54 | 0.52 | 0.57 | **0.0000** | 4225 | 958 | 1184 | 1326 | 18.48 | 52.83 |
| ck | Specific Persist | fights | fights | 0.58 | 0.49 | 0.66 | **0.0000** | 5109 | 74 | 249 | 47 | 1.43 | 15.88 |
| bf | Specific Persist | worry | worry | 0.54 | 0.51 | 0.57 | **0.0000** | 4335 | 995 | 1247 | 1383 | 18.67 | 52.59 |
| ck | Specific Persist | steals | steals | 0.54 | 0.44 | 0.64 | **0.0000** | 5242 | 88 | 122 | 27 | 1.65 | 18.12 |
| bf | Specific Persist | dysph | dysph | 0.35 | 0.29 | 0.41 | **0.0000** | 6231 | 544 | 379 | 122 | 8.03 | 24.35 |
| ck | Specific Persist | worth | worth | 0.43 | 0.36 | 0.49 | **0.0000** | 6513 | 262 | 459 | 99 | 3.87 | 17.74 |
| bf | Specific Persist | dysph | dysph | 0.40 | 0.32 | 0.48 | **0.0000** | 3744 | 283 | 182 | 64 | 7.03 | 26.02 |
| ck | Specific Persist | attend | attend | 0.65 | 0.62 | 0.67 | **0.0000** | 3412 | 615 | 1441 | 1866 | 15.27 | 56.43 |
| bf | Specific Persist | dysph | dysph | 0.41 | 0.35 | 0.47 | **0.0000** | 4640 | 554 | 308 | 156 | 10.67 | 33.62 |
| ck | Specific Persist | hyper | hyper | 0.64 | 0.62 | 0.67 | **0.0000** | 4801 | 393 | 1271 | 869 | 7.57 | 40.61 |
| bf | Specific Persist | dysph | dysph | 0.28 | 0.19 | 0.37 | **0.0000** | 4137 | 272 | 213 | 43 | 6.17 | 16.80 |
| ck | Specific Persist | argues | argues | 0.55 | 0.52 | 0.58 | **0.0000** | 3575 | 834 | 1363 | 1562 | 18.92 | 53.40 |
| bf | Specific Persist | dysph | dysph | 0.42 | 0.36 | 0.48 | **0.0000** | 5220 | 564 | 284 | 142 | 9.75 | 33.33 |
| ck | Specific Persist | temper | temper | 0.55 | 0.51 | 0.59 | **0.0000** | 5417 | 367 | 1089 | 461 | 6.35 | 29.74 |
| bf | Specific Persist | dysph | dysph | 0.45 | 0.40 | 0.49 | **0.0000** | 6026 | 909 | 451 | 307 | 13.11 | 40.50 |
| ck | Specific Persist | fights | fights | 0.59 | 0.52 | 0.66 | **0.0000** | 6833 | 102 | 332 | 67 | 1.47 | 16.79 |
| bf | Specific Persist | dysph | dysph | 0.46 | 0.42 | 0.50 | **0.0000** | 6183 | 922 | 505 | 350 | 12.98 | 40.94 |
| ck | Specific Persist | steals | steals | 0.57 | 0.49 | 0.65 | **0.0000** | 6998 | 107 | 187 | 42 | 1.51 | 18.34 |
| bf | Specific Persist | worth | worth | 0.52 | 0.44 | 0.60 | **0.0000** | 3894 | 146 | 177 | 56 | 3.61 | 24.03 |
| ck | Specific Persist | attend | attend | 0.63 | 0.61 | 0.66 | **0.0000** | 3335 | 705 | 1336 | 1900 | 17.45 | 58.71 |
| bf | Specific Persist | worth | worth | 0.52 | 0.46 | 0.57 | **0.0000** | 4870 | 314 | 329 | 144 | 6.06 | 30.44 |
| ck | Specific Persist | hyper | hyper | 0.63 | 0.60 | 0.66 | **0.0000** | 4748 | 436 | 1226 | 866 | 8.41 | 41.40 |
| bf | Specific Persist | worth | worth | 0.42 | 0.34 | 0.51 | **0.0000** | 4200 | 150 | 263 | 52 | 3.45 | 16.51 |
| ck | Specific Persist | argues | argues | 0.55 | 0.52 | 0.57 | **0.0000** | 3442 | 908 | 1290 | 1636 | 20.87 | 55.91 |
| bf | Specific Persist | worth | worth | 0.49 | 0.43 | 0.55 | **0.0000** | 5385 | 326 | 362 | 136 | 5.71 | 27.31 |
| ck | Specific Persist | temper | temper | 0.55 | 0.51 | 0.59 | **0.0000** | 5316 | 395 | 1071 | 494 | 6.92 | 31.57 |
| bf | Specific Persist | worth | worth | 0.56 | 0.51 | 0.60 | **0.0000** | 6275 | 567 | 521 | 329 | 8.29 | 38.71 |
| ck | Specific Persist | fights | fights | 0.63 | 0.57 | 0.69 | **0.0000** | 6738 | 104 | 349 | 85 | 1.52 | 19.59 |
| bf | Specific Persist | worth | worth | 0.57 | 0.53 | 0.61 | **0.0000** | 6470 | 583 | 549 | 357 | 8.27 | 39.40 |
| ck | Specific Persist | steals | steals | 0.55 | 0.47 | 0.64 | **0.0000** | 6937 | 116 | 182 | 41 | 1.64 | 18.39 |
| bf | Specific Persist | attend | attend | 0.49 | 0.46 | 0.53 | **0.0000** | 3336 | 596 | 977 | 749 | 15.16 | 43.40 |
| ck | Specific Persist | hyper | hyper | 0.47 | 0.37 | 0.56 | **0.0000** | 3858 | 74 | 300 | 41 | 1.88 | 12.02 |
| bf | Specific Persist | attend | attend | 0.54 | 0.50 | 0.58 | **0.0000** | 2630 | 370 | 966 | 699 | 12.33 | 41.98 |
| ck | Specific Persist | argues | argues | 0.51 | 0.46 | 0.55 | **0.0000** | 2572 | 428 | 726 | 547 | 14.27 | 42.97 |
| bf | Specific Persist | attend | attend | 0.63 | 0.60 | 0.66 | **0.0000** | 3041 | 601 | 1100 | 1468 | 16.50 | 57.17 |
| ck | Specific Persist | temper | temper | 0.53 | 0.47 | 0.59 | **0.0000** | 3497 | 145 | 492 | 139 | 3.98 | 22.03 |
| bf | Specific Persist | attend | attend | 0.69 | 0.67 | 0.72 | **0.0000** | 3317 | 825 | 1139 | 2412 | 19.92 | 67.92 |
| ck | Specific Persist | fights | fights | 0.55 | 0.42 | 0.68 | **0.0000** | 4104 | 38 | 115 | 16 | 0.92 | 12.21 |
| bf | Specific Persist | attend | attend | 0.69 | 0.67 | 0.71 | **0.0000** | 3386 | 838 | 1215 | 2521 | 19.84 | 67.48 |
| ck | Specific Persist | steals | steals | 0.63 | 0.48 | 0.79 | **0.0000** | 4201 | 23 | 41 | 8 | 0.54 | 16.33 |
| bf | Specific Persist | hyper | hyper | 0.57 | 0.52 | 0.62 | **0.0000** | 3559 | 168 | 697 | 241 | 4.51 | 25.69 |
| ck | Specific Persist | argues | argues | 0.53 | 0.49 | 0.56 | **0.0000** | 3029 | 698 | 932 | 999 | 18.73 | 51.73 |
| bf | Specific Persist | hyper | hyper | 0.61 | 0.58 | 0.65 | **0.0000** | 4308 | 334 | 985 | 583 | 7.20 | 37.18 |
| ck | Specific Persist | temper | temper | 0.55 | 0.50 | 0.60 | **0.0000** | 4372 | 270 | 722 | 294 | 5.82 | 28.94 |
| bf | Specific Persist | hyper | hyper | 0.67 | 0.64 | 0.69 | **0.0000** | 4908 | 516 | 1185 | 1084 | 9.51 | 47.77 |
| ck | Specific Persist | fights | fights | 0.57 | 0.48 | 0.67 | **0.0000** | 5365 | 59 | 201 | 33 | 1.09 | 14.10 |
| bf | Specific Persist | hyper | hyper | 0.66 | 0.64 | 0.69 | **0.0000** | 5007 | 536 | 1262 | 1155 | 9.67 | 47.79 |
| ck | Specific Persist | steals | steals | 0.67 | 0.58 | 0.77 | **0.0000** | 5494 | 49 | 90 | 25 | 0.88 | 21.74 |
| bf | Specific Persist | argues | argues | 0.48 | 0.45 | 0.52 | **0.0000** | 3453 | 795 | 1018 | 944 | 18.71 | 48.11 |
| ck | Specific Persist | temper | temper | 0.49 | 0.40 | 0.58 | **0.0000** | 3988 | 75 | 289 | 43 | 1.85 | 12.95 |
| bf | Specific Persist | argues | argues | 0.59 | 0.57 | 0.62 | **0.0000** | 3500 | 1081 | 1133 | 1979 | 23.60 | 63.59 |
| ck | Specific Persist | fights | fights | 0.38 | 0.13 | 0.62 | **0.0023** | 4563 | 18 | 81 | 3 | 0.39 | 3.57 |
| bf | Specific Persist | argues | argues | 0.60 | 0.58 | 0.63 | **0.0000** | 3523 | 1084 | 1198 | 2155 | 23.53 | 64.27 |
| ck | Specific Persist | steals | steals | 0.46 | 0.24 | 0.67 | **0.0000** | 4584 | 23 | 54 | 4 | 0.50 | 6.90 |
| bf | Specific Persist | temper | temper | 0.58 | 0.55 | 0.61 | **0.0000** | 5556 | 501 | 1020 | 616 | 8.27 | 37.65 |
| ck | Specific Persist | fights | fights | 0.49 | 0.36 | 0.62 | **0.0000** | 6010 | 47 | 139 | 14 | 0.78 | 9.15 |
| bf | Specific Persist | temper | temper | 0.61 | 0.58 | 0.64 | **0.0000** | 5573 | 507 | 1120 | 760 | 8.34 | 40.43 |
| ck | Specific Persist | steals | steals | 0.57 | 0.47 | 0.68 | **0.0000** | 6012 | 68 | 108 | 22 | 1.12 | 16.92 |
| bf | Specific Persist | fights | fights | 0.59 | 0.53 | 0.65 | **0.0000** | 7322 | 144 | 397 | 97 | 1.93 | 19.64 |
| ck | Specific Persist | steals | steals | 0.57 | 0.49 | 0.65 | **0.0000** | 7324 | 142 | 178 | 49 | 1.90 | 21.59 |

| Supplemental Table S3F. Tetrachoric correlations from 4 x 4 pairwise analyses of all 90 combinations of the 10 selected psychological problems with every other problem to quantify the associations of each baseline problem with the same problem assessed in the **third annual follow-up** in one of the two homotypic paths (the **specific persist path** defined by only x_1_ at baseline and only x_2_ at follow-up) for problems dichotomized at the **high rating cut (0 or 1 vs 2).** r_t_ = tetrachoric correlation. –CL and +CL = lower and upper 95% confidence intervals for the tetrachoric correlation. c00 = number without the predictor or the outcome; c01 = number without predictor with the outcome; c10 = number with the predictor without the outcome; c11 = number with the predictor and the outcome. Base% = percent of the outcome only x_2_ among children with neither x_1_ nor the other member of the pair, y_1_, at baseline. Obs% = Observed percent of specific persistence (i.e., only x_2_ among children with only x_1_ at baseline). Raw P values significant after FDR adjustment in **bold**. | | | | | | | | | | | | | |
| --- | --- | --- | --- | --- | --- | --- | --- | --- | --- | --- | --- | --- | --- |
| prefix | Path | Predictor | Outcome | r_t_ | -CL | +CL | P | c00 | c01 | c10 | c11 | Base% | Obs% |
| bf | Specific Persist | fear | fear | 0.28 | 0.08 | 0.47 | **0.0054** | 7799 | 74 | 78 | 4 | 0.94 | 4.88 |
| ck | Specific Persist | worry | worry | 0.50 | 0.41 | 0.59 | **0.0000** | 7734 | 139 | 193 | 37 | 1.77 | 16.09 |
| bf | Specific Persist | fear | fear | 0.56 | 0.47 | 0.64 | **0.0000** | 7926 | 139 | 146 | 38 | 1.72 | 20.65 |
| ck | Specific Persist | dysph | dysph | 0.28 | 0.02 | 0.53 | **0.0316** | 7995 | 70 | 36 | 2 | 0.87 | 5.26 |
| bf | Specific Persist | fear | fear | 0.54 | 0.45 | 0.63 | **0.0000** | 7909 | 145 | 139 | 35 | 1.80 | 20.11 |
| ck | Specific Persist | worth | worth | 0.50 | 0.32 | 0.69 | **0.0000** | 8013 | 41 | 43 | 5 | 0.51 | 10.42 |
| bf | Specific Persist | fear | fear | 0.49 | 0.38 | 0.61 | **0.0000** | 7112 | 101 | 92 | 17 | 1.40 | 15.60 |
| ck | Specific Persist | attend | attend | 0.70 | 0.66 | 0.73 | **0.0000** | 6880 | 333 | 528 | 362 | 4.62 | 40.67 |
| bf | Specific Persist | fear | fear | 0.59 | 0.51 | 0.68 | **0.0000** | 7471 | 139 | 98 | 34 | 1.83 | 25.76 |
| ck | Specific Persist | hyper | hyper | 0.67 | 0.62 | 0.71 | **0.0000** | 7432 | 178 | 352 | 141 | 2.34 | 28.60 |
| bf | Specific Persist | fear | fear | 0.59 | 0.50 | 0.68 | **0.0000** | 7343 | 111 | 100 | 29 | 1.49 | 22.48 |
| ck | Specific Persist | argues | argues | 0.63 | 0.59 | 0.68 | **0.0000** | 7058 | 396 | 398 | 251 | 5.31 | 38.67 |
| bf | Specific Persist | fear | fear | 0.56 | 0.47 | 0.65 | **0.0000** | 7694 | 145 | 116 | 34 | 1.85 | 22.67 |
| ck | Specific Persist | temper | temper | 0.61 | 0.54 | 0.68 | **0.0000** | 7723 | 116 | 209 | 55 | 1.48 | 20.83 |
| bf | Specific Persist | fear | fear | 0.57 | 0.50 | 0.65 | **0.0000** | 7892 | 181 | 150 | 50 | 2.24 | 25.00 |
| ck | Specific Persist | fights | fights | 0.64 | 0.45 | 0.84 | **0.0000** | 8056 | 17 | 26 | 4 | 0.21 | 13.33 |
| bf | Specific Persist | fear | fear | 0.57 | 0.49 | 0.64 | **0.0000** | 7894 | 180 | 153 | 49 | 2.23 | 24.26 |
| ck | Specific Persist | steals | steals | 0.59 | 0.39 | 0.79 | **0.0000** | 8047 | 27 | 25 | 4 | 0.33 | 13.79 |
| bf | Specific Persist | worry | worry | 0.57 | 0.50 | 0.64 | **0.0000** | 7732 | 195 | 246 | 76 | 2.46 | 23.60 |
| ck | Specific Persist | dysph | dysph | 0.36 | 0.10 | 0.62 | **0.0071** | 7872 | 55 | 26 | 2 | 0.69 | 7.14 |
| bf | Specific Persist | worry | worry | 0.55 | 0.48 | 0.62 | **0.0000** | 7711 | 206 | 239 | 72 | 2.60 | 23.15 |
| ck | Specific Persist | worth | worth | 0.37 | 0.11 | 0.63 | **0.0048** | 7882 | 35 | 36 | 2 | 0.44 | 5.26 |
| bf | Specific Persist | worry | worry | 0.53 | 0.45 | 0.61 | **0.0000** | 6951 | 150 | 177 | 44 | 2.11 | 19.91 |
| ck | Specific Persist | attend | attend | 0.71 | 0.68 | 0.74 | **0.0000** | 6792 | 309 | 505 | 349 | 4.35 | 40.87 |
| bf | Specific Persist | worry | worry | 0.57 | 0.50 | 0.64 | **0.0000** | 7276 | 195 | 204 | 67 | 2.61 | 24.72 |
| ck | Specific Persist | hyper | hyper | 0.67 | 0.62 | 0.72 | **0.0000** | 7300 | 171 | 346 | 138 | 2.29 | 28.51 |
| bf | Specific Persist | worry | worry | 0.58 | 0.50 | 0.65 | **0.0000** | 7175 | 160 | 190 | 58 | 2.18 | 23.39 |
| ck | Specific Persist | argues | argues | 0.63 | 0.59 | 0.67 | **0.0000** | 6948 | 387 | 383 | 237 | 5.28 | 38.23 |
| bf | Specific Persist | worry | worry | 0.58 | 0.52 | 0.65 | **0.0000** | 7505 | 198 | 212 | 74 | 2.57 | 25.87 |
| ck | Specific Persist | temper | temper | 0.62 | 0.55 | 0.69 | **0.0000** | 7587 | 116 | 197 | 55 | 1.51 | 21.83 |
| bf | Specific Persist | worry | worry | 0.61 | 0.55 | 0.67 | **0.0000** | 7693 | 236 | 242 | 102 | 2.98 | 29.65 |
| ck | Specific Persist | fights | fights | 0.53 | 0.26 | 0.79 | **0.0001** | 7912 | 17 | 24 | 2 | 0.21 | 7.69 |
| bf | Specific Persist | worry | worry | 0.61 | 0.55 | 0.67 | **0.0000** | 7691 | 237 | 245 | 103 | 2.99 | 29.60 |
| ck | Specific Persist | steals | steals | 0.55 | 0.32 | 0.77 | **0.0000** | 7902 | 26 | 24 | 3 | 0.33 | 11.11 |
| bf | Specific Persist | dysph | dysph | 0.39 | 0.19 | 0.59 | **0.0001** | 8109 | 78 | 37 | 4 | 0.95 | 9.76 |
| ck | Specific Persist | worth | worth | 0.46 | 0.27 | 0.64 | **0.0000** | 8143 | 44 | 56 | 5 | 0.54 | 8.20 |
| bf | Specific Persist | dysph | dysph | 0.50 | 0.29 | 0.70 | **0.0000** | 7236 | 61 | 21 | 4 | 0.84 | 16.00 |
| ck | Specific Persist | attend | attend | 0.71 | 0.68 | 0.75 | **0.0000** | 6945 | 352 | 541 | 411 | 4.82 | 43.17 |
| bf | Specific Persist | dysph | dysph | 0.49 | 0.33 | 0.65 | **0.0000** | 7611 | 91 | 33 | 7 | 1.18 | 17.50 |
| ck | Specific Persist | hyper | hyper | 0.69 | 0.65 | 0.73 | **0.0000** | 7519 | 183 | 379 | 168 | 2.38 | 30.71 |
| bf | Specific Persist | dysph | dysph | 0.36 | 0.10 | 0.63 | **0.0066** | 7499 | 60 | 22 | 2 | 0.79 | 8.33 |
| ck | Specific Persist | argues | argues | 0.64 | 0.60 | 0.68 | **0.0000** | 7152 | 407 | 418 | 272 | 5.38 | 39.42 |
| bf | Specific Persist | dysph | dysph | 0.42 | 0.22 | 0.62 | **0.0001** | 7867 | 93 | 25 | 4 | 1.17 | 13.79 |
| ck | Specific Persist | temper | temper | 0.64 | 0.58 | 0.71 | **0.0000** | 7837 | 123 | 221 | 68 | 1.55 | 23.53 |
| bf | Specific Persist | dysph | dysph | 0.51 | 0.37 | 0.65 | **0.0000** | 8102 | 117 | 43 | 11 | 1.42 | 20.37 |
| ck | Specific Persist | fights | fights | 0.48 | 0.22 | 0.74 | **0.0003** | 8198 | 21 | 28 | 2 | 0.26 | 6.67 |
| bf | Specific Persist | dysph | dysph | 0.49 | 0.34 | 0.63 | **0.0000** | 8105 | 116 | 45 | 10 | 1.41 | 18.18 |
| ck | Specific Persist | steals | steals | 0.68 | 0.52 | 0.84 | **0.0000** | 8193 | 28 | 22 | 6 | 0.34 | 21.43 |
| bf | Specific Persist | worth | worth | 0.57 | 0.38 | 0.75 | **0.0000** | 7251 | 36 | 29 | 5 | 0.49 | 14.71 |
| ck | Specific Persist | attend | attend | 0.71 | 0.68 | 0.74 | **0.0000** | 6929 | 358 | 536 | 405 | 4.91 | 43.04 |
| bf | Specific Persist | worth | worth | 0.53 | 0.37 | 0.68 | **0.0000** | 7627 | 58 | 48 | 8 | 0.75 | 14.29 |
| ck | Specific Persist | hyper | hyper | 0.69 | 0.64 | 0.73 | **0.0000** | 7501 | 184 | 378 | 165 | 2.39 | 30.39 |
| bf | Specific Persist | worth | worth | 0.56 | 0.39 | 0.73 | **0.0000** | 7500 | 42 | 34 | 6 | 0.56 | 15.00 |
| ck | Specific Persist | argues | argues | 0.64 | 0.60 | 0.68 | **0.0000** | 7123 | 419 | 409 | 277 | 5.56 | 40.38 |
| bf | Specific Persist | worth | worth | 0.52 | 0.36 | 0.69 | **0.0000** | 7881 | 59 | 41 | 7 | 0.74 | 14.58 |
| ck | Specific Persist | temper | temper | 0.61 | 0.55 | 0.68 | **0.0000** | 7808 | 132 | 224 | 64 | 1.66 | 22.22 |
| bf | Specific Persist | worth | worth | 0.55 | 0.43 | 0.68 | **0.0000** | 8119 | 78 | 62 | 13 | 0.95 | 17.33 |
| ck | Specific Persist | fights | fights | 0.63 | 0.43 | 0.82 | **0.0000** | 8178 | 19 | 27 | 4 | 0.23 | 12.90 |
| bf | Specific Persist | worth | worth | 0.55 | 0.42 | 0.67 | **0.0000** | 8120 | 80 | 62 | 13 | 0.98 | 17.33 |
| ck | Specific Persist | steals | steals | 0.68 | 0.52 | 0.84 | **0.0000** | 8173 | 27 | 22 | 6 | 0.33 | 21.43 |
| bf | Specific Persist | attend | attend | 0.60 | 0.55 | 0.65 | **0.0000** | 6917 | 264 | 396 | 165 | 3.68 | 29.41 |
| ck | Specific Persist | hyper | hyper | 0.51 | 0.38 | 0.64 | **0.0000** | 7130 | 51 | 127 | 14 | 0.71 | 9.93 |
| bf | Specific Persist | attend | attend | 0.66 | 0.62 | 0.70 | **0.0000** | 6650 | 241 | 469 | 223 | 3.50 | 32.23 |
| ck | Specific Persist | argues | argues | 0.58 | 0.53 | 0.64 | **0.0000** | 6621 | 270 | 303 | 128 | 3.92 | 29.70 |
| bf | Specific Persist | attend | attend | 0.70 | 0.66 | 0.73 | **0.0000** | 6832 | 318 | 502 | 337 | 4.45 | 40.17 |
| ck | Specific Persist | temper | temper | 0.61 | 0.52 | 0.70 | **0.0000** | 7079 | 71 | 142 | 30 | 0.99 | 17.44 |
| bf | Specific Persist | attend | attend | 0.72 | 0.69 | 0.75 | **0.0000** | 6931 | 378 | 532 | 432 | 5.17 | 44.81 |
| ck | Specific Persist | fights | fights | . | . | . | . | 7300 | 9 | 13 | 0 | 0.12 | 0.00 |
| bf | Specific Persist | attend | attend | 0.71 | 0.67 | 0.74 | **0.0000** | 6929 | 381 | 545 | 421 | 5.21 | 43.58 |
| ck | Specific Persist | steals | steals | 0.52 | 0.17 | 0.87 | **0.0038** | 7296 | 14 | 11 | 1 | 0.19 | 8.33 |
| bf | Specific Persist | hyper | hyper | 0.61 | 0.54 | 0.68 | **0.0000** | 7115 | 113 | 285 | 70 | 1.56 | 19.72 |
| ck | Specific Persist | argues | argues | 0.61 | 0.56 | 0.66 | **0.0000** | 6890 | 338 | 335 | 179 | 4.68 | 34.82 |
| bf | Specific Persist | hyper | hyper | 0.65 | 0.60 | 0.71 | **0.0000** | 7372 | 162 | 334 | 121 | 2.15 | 26.59 |
| ck | Specific Persist | temper | temper | 0.60 | 0.52 | 0.68 | **0.0000** | 7433 | 101 | 167 | 41 | 1.34 | 19.71 |
| bf | Specific Persist | hyper | hyper | 0.68 | 0.63 | 0.72 | **0.0000** | 7521 | 200 | 383 | 169 | 2.59 | 30.62 |
| ck | Specific Persist | fights | fights | 0.59 | 0.32 | 0.85 | **0.0000** | 7708 | 13 | 19 | 2 | 0.17 | 9.52 |
| bf | Specific Persist | hyper | hyper | 0.67 | 0.63 | 0.72 | **0.0000** | 7529 | 194 | 388 | 165 | 2.51 | 29.84 |
| ck | Specific Persist | steals | steals | 0.65 | 0.43 | 0.86 | **0.0000** | 7706 | 17 | 16 | 3 | 0.22 | 15.79 |
| bf | Specific Persist | argues | argues | 0.57 | 0.52 | 0.62 | **0.0000** | 7111 | 350 | 362 | 166 | 4.69 | 31.44 |
| ck | Specific Persist | temper | temper | 0.55 | 0.41 | 0.69 | **0.0000** | 7358 | 38 | 97 | 11 | 0.51 | 10.19 |
| bf | Specific Persist | argues | argues | 0.65 | 0.61 | 0.69 | **0.0000** | 7138 | 436 | 409 | 290 | 5.76 | 41.49 |
| ck | Specific Persist | fights | fights | . | . | . | . | 7568 | 6 | 9 | 0 | 0.08 | 0.00 |
| bf | Specific Persist | argues | argues | 0.65 | 0.61 | 0.68 | **0.0000** | 7138 | 432 | 417 | 289 | 5.71 | 40.93 |
| ck | Specific Persist | steals | steals | 0.58 | 0.23 | 0.92 | **0.0012** | 7562 | 8 | 12 | 1 | 0.11 | 7.69 |
| bf | Specific Persist | temper | temper | 0.61 | 0.54 | 0.67 | **0.0000** | 7839 | 136 | 233 | 65 | 1.71 | 21.81 |
| ck | Specific Persist | fights | fights | . | . | . | . | 7965 | 10 | 14 | 0 | 0.13 | 0.00 |
| bf | Specific Persist | temper | temper | 0.63 | 0.56 | 0.69 | **0.0000** | 7833 | 134 | 238 | 71 | 1.68 | 22.98 |
| ck | Specific Persist | steals | steals | 0.74 | 0.58 | 0.90 | **0.0000** | 7952 | 15 | 17 | 5 | 0.19 | 22.73 |
| bf | Specific Persist | fights | fights | 0.49 | 0.23 | 0.75 | **0.0003** | 8226 | 19 | 29 | 2 | 0.23 | 6.45 |
| ck | Specific Persist | steals | steals | 0.67 | 0.51 | 0.83 | **0.0000** | 8215 | 30 | 22 | 6 | 0.36 | 21.43 |

| Supplemental Table S3G. Tetrachoric correlations from 4 x 4 pairwise analyses of all 90 combinations of the 10 selected psychological problems with every other problem to quantify the associations of each baseline problem with outcomes in the **first annual follow-up** that define four parsed paths traditionally conflated in estimates of change: ***add path*** (x_1_ at baseline and x_2_ + y_2_ at follow-up); ***joint persist path*** (x_1_ + y_1_ at baseline and x_2_ + y_2_ at follow-up); ***shift path*** (only x_1_ at baseline and only y_2_ at follow-up ***subtract x path*** (x_1_ + y_1_ at baseline and only y_2_ at follow-up); and ***subtract y path*** (x_1_ + y_1_ at baseline and only x_2_ at follow-up). Results are for problems dichotomized at the **low rating cut (0 vs 1 or 2).** r_t_ = tetrachoric correlation. –CL and +CL = lower and upper 95% confidence intervals for the tetrachoric correlation. c00 = number without the predictor or the outcome; c01 = number without predictor with the outcome; c10 = number with the predictor without the outcome; c11 = number with the predictor and the outcome. Base% = percent of the outcome only x_2_ among children with neither x_1_ nor the other member of the pair, y_1_, at baseline. Obs% = Observed percent of outcomes on the indicated path. Raw P values significant after FDR adjustment in **bold**. | | | | | | | | | | | | | |
| --- | --- | --- | --- | --- | --- | --- | --- | --- | --- | --- | --- | --- | --- |
| prefix | Parsed paths | Predictor | Outcome | r_t_ | -CL | +CL | P | c00 | c01 | c10 | c11 | Base% | Obs% |
| dh | Add | fear | fear+worry | 0.52 | 0.46 | 0.57 | **0.0000** | 5272 | 270 | 412 | 145 | 4.87 | 26.03 |
| dl | Add | worry | worry+fear | 0.46 | 0.42 | 0.51 | **0.0000** | 5272 | 270 | 1323 | 327 | 4.87 | 19.82 |
| dh | Add | fear | fear+dysph | 0.42 | 0.36 | 0.47 | **0.0000** | 6497 | 170 | 1281 | 162 | 2.55 | 11.23 |
| dl | Add | dysph | dysph+fear | 0.48 | 0.41 | 0.55 | **0.0000** | 6497 | 170 | 442 | 83 | 2.55 | 15.81 |
| dh | Add | fear | fear+worth | 0.42 | 0.36 | 0.48 | **0.0000** | 6501 | 157 | 1258 | 149 | 2.36 | 10.59 |
| dl | Add | worth | worth+fear | 0.48 | 0.41 | 0.55 | **0.0000** | 6501 | 157 | 455 | 79 | 2.36 | 14.79 |
| dh | Add | fear | fear+attend | 0.47 | 0.40 | 0.54 | **0.0000** | 3950 | 109 | 553 | 92 | 2.69 | 14.26 |
| dl | Add | attend | attend+fear | 0.47 | 0.42 | 0.51 | **0.0000** | 3950 | 109 | 2724 | 409 | 2.69 | 13.05 |
| dh | Add | fear | fear+hyper | 0.49 | 0.43 | 0.56 | **0.0000** | 5127 | 88 | 914 | 110 | 1.69 | 10.74 |
| dl | Add | hyper | hyper+fear | 0.53 | 0.48 | 0.58 | **0.0000** | 5127 | 88 | 1744 | 233 | 1.69 | 11.79 |
| dh | Add | fear | fear+argues | 0.47 | 0.40 | 0.53 | **0.0000** | 4271 | 137 | 648 | 119 | 3.11 | 15.51 |
| dl | Add | argues | argues+fear | 0.42 | 0.37 | 0.47 | **0.0000** | 4271 | 137 | 2438 | 346 | 3.11 | 12.43 |
| dh | Add | fear | fear+temper | 0.44 | 0.37 | 0.50 | **0.0000** | 5601 | 111 | 1028 | 112 | 1.94 | 9.82 |
| dl | Add | temper | temper+fear | 0.53 | 0.47 | 0.58 | **0.0000** | 5601 | 111 | 1287 | 193 | 1.94 | 13.04 |
| dh | Add | fear | fear+fights | 0.34 | 0.26 | 0.43 | **0.0000** | 6711 | 53 | 1692 | 58 | 0.78 | 3.31 |
| dl | Add | fights | fights+fear | 0.52 | 0.43 | 0.61 | **0.0000** | 6711 | 53 | 392 | 36 | 0.78 | 8.41 |
| dh | Add | fear | fear+steals | 0.39 | 0.29 | 0.49 | **0.0000** | 6894 | 26 | 1822 | 42 | 0.38 | 2.25 |
| dl | Add | steals | steals+fear | 0.65 | 0.56 | 0.74 | **0.0000** | 6894 | 26 | 245 | 27 | 0.38 | 9.93 |
| dh | Add | worry | worry+dysph | 0.42 | 0.37 | 0.46 | **0.0000** | 5608 | 177 | 2042 | 283 | 3.06 | 12.17 |
| dl | Add | dysph | dysph+worry | 0.48 | 0.41 | 0.56 | **0.0000** | 5608 | 177 | 253 | 61 | 3.06 | 19.43 |
| dh | Add | worry | worry+worth | 0.41 | 0.36 | 0.46 | **0.0000** | 5590 | 156 | 2063 | 256 | 2.71 | 11.04 |
| dl | Add | worth | worth+worry | 0.52 | 0.44 | 0.59 | **0.0000** | 5590 | 156 | 284 | 69 | 2.71 | 19.55 |
| dh | Add | worry | worry+attend | 0.36 | 0.30 | 0.42 | **0.0000** | 3311 | 169 | 1045 | 179 | 4.86 | 14.62 |
| dl | Add | attend | attend+worry | 0.43 | 0.38 | 0.48 | **0.0000** | 3311 | 169 | 2159 | 460 | 4.86 | 17.56 |
| dh | Add | worry | worry+hyper | 0.43 | 0.37 | 0.49 | **0.0000** | 4329 | 101 | 1626 | 183 | 2.28 | 10.12 |
| dl | Add | hyper | hyper+worry | 0.55 | 0.50 | 0.60 | **0.0000** | 4329 | 101 | 1413 | 256 | 2.28 | 15.34 |
| dh | Add | worry | worry+argues | 0.44 | 0.39 | 0.50 | **0.0000** | 3693 | 173 | 1079 | 230 | 4.47 | 17.57 |
| dl | Add | argues | argues+worry | 0.46 | 0.42 | 0.51 | **0.0000** | 3693 | 173 | 1827 | 406 | 4.47 | 18.18 |
| dh | Add | worry | worry+temper | 0.41 | 0.35 | 0.47 | **0.0000** | 4799 | 123 | 1732 | 198 | 2.50 | 10.26 |
| dl | Add | temper | temper+worry | 0.54 | 0.49 | 0.60 | **0.0000** | 4799 | 123 | 983 | 194 | 2.50 | 16.48 |
| dh | Add | worry | worry+fights | 0.33 | 0.26 | 0.41 | **0.0000** | 5698 | 51 | 2670 | 95 | 0.89 | 3.44 |
| dl | Add | fights | fights+worry | 0.52 | 0.42 | 0.62 | **0.0000** | 5698 | 51 | 318 | 32 | 0.89 | 9.14 |
| dh | Add | worry | worry+steals | 0.30 | 0.21 | 0.39 | **0.0000** | 5864 | 31 | 2833 | 56 | 0.53 | 1.94 |
| dl | Add | steals | steals+worry | 0.68 | 0.59 | 0.77 | **0.0000** | 5864 | 31 | 174 | 30 | 0.53 | 14.71 |
| dh | Add | dysph | dysph+worth | 0.47 | 0.40 | 0.53 | **0.0000** | 7298 | 193 | 486 | 88 | 2.58 | 15.33 |
| dl | Add | worth | worth+dysph | 0.47 | 0.41 | 0.54 | **0.0000** | 7298 | 193 | 523 | 96 | 2.58 | 15.51 |
| dh | Add | dysph | dysph+attend | 0.48 | 0.38 | 0.57 | **0.0000** | 4345 | 87 | 235 | 37 | 1.96 | 13.60 |
| dl | Add | attend | attend+dysph | 0.46 | 0.41 | 0.51 | **0.0000** | 4345 | 87 | 3305 | 373 | 1.96 | 10.14 |
| dh | Add | dysph | dysph+hyper | 0.36 | 0.27 | 0.45 | **0.0000** | 5624 | 95 | 481 | 39 | 1.66 | 7.50 |
| dl | Add | hyper | hyper+dysph | 0.45 | 0.40 | 0.51 | **0.0000** | 5624 | 95 | 2181 | 210 | 1.66 | 8.78 |
| dh | Add | dysph | dysph+argues | 0.54 | 0.46 | 0.62 | **0.0000** | 4783 | 112 | 226 | 54 | 2.29 | 19.29 |
| dl | Add | argues | argues+dysph | 0.43 | 0.38 | 0.48 | **0.0000** | 4783 | 112 | 2882 | 333 | 2.29 | 10.36 |
| dh | Add | dysph | dysph+temper | 0.35 | 0.26 | 0.44 | **0.0000** | 6250 | 125 | 437 | 40 | 1.96 | 8.39 |
| dl | Add | temper | temper+dysph | 0.47 | 0.41 | 0.52 | **0.0000** | 6250 | 125 | 1551 | 184 | 1.96 | 10.61 |
| dh | Add | dysph | dysph+fights | 0.46 | 0.38 | 0.55 | **0.0000** | 7604 | 53 | 810 | 47 | 0.69 | 5.48 |
| dl | Add | fights | fights+dysph | 0.52 | 0.43 | 0.62 | **0.0000** | 7604 | 53 | 417 | 36 | 0.69 | 7.95 |
| dh | Add | dysph | dysph+steals | 0.38 | 0.28 | 0.48 | **0.0000** | 7778 | 45 | 929 | 32 | 0.58 | 3.33 |
| dl | Add | steals | steals+dysph | 0.59 | 0.49 | 0.68 | **0.0000** | 7778 | 45 | 258 | 29 | 0.58 | 10.10 |
| dh | Add | worth | worth+attend | 0.49 | 0.40 | 0.59 | **0.0000** | 4362 | 85 | 220 | 37 | 1.91 | 14.40 |
| dl | Add | attend | attend+worth | 0.44 | 0.39 | 0.49 | **0.0000** | 4362 | 85 | 3274 | 344 | 1.91 | 9.51 |
| dh | Add | worth | worth+hyper | 0.48 | 0.40 | 0.57 | **0.0000** | 5653 | 72 | 465 | 49 | 1.26 | 9.53 |
| dl | Add | hyper | hyper+worth | 0.50 | 0.44 | 0.55 | **0.0000** | 5653 | 72 | 2141 | 199 | 1.26 | 8.50 |
| dh | Add | worth | worth+argues | 0.56 | 0.48 | 0.64 | **0.0000** | 4738 | 92 | 285 | 60 | 1.90 | 17.39 |
| dl | Add | argues | argues+worth | 0.46 | 0.41 | 0.51 | **0.0000** | 4738 | 92 | 2907 | 328 | 1.90 | 10.14 |
| dh | Add | worth | worth+temper | 0.52 | 0.45 | 0.60 | **0.0000** | 6222 | 86 | 480 | 64 | 1.36 | 11.76 |
| dl | Add | temper | temper+worth | 0.56 | 0.51 | 0.61 | **0.0000** | 6222 | 86 | 1556 | 201 | 1.36 | 11.44 |
| dh | Add | worth | worth+fights | 0.48 | 0.39 | 0.57 | **0.0000** | 7531 | 41 | 897 | 45 | 0.54 | 4.78 |
| dl | Add | fights | fights+worth | 0.59 | 0.51 | 0.68 | **0.0000** | 7531 | 41 | 450 | 43 | 0.54 | 8.72 |
| dh | Add | worth | worth+steals | 0.42 | 0.31 | 0.52 | **0.0000** | 7748 | 34 | 971 | 31 | 0.44 | 3.09 |
| dl | Add | steals | steals+worth | 0.61 | 0.51 | 0.70 | **0.0000** | 7748 | 34 | 257 | 26 | 0.44 | 9.19 |
| dh | Add | attend | attend+hyper | 0.45 | 0.40 | 0.50 | **0.0000** | 4143 | 186 | 1584 | 326 | 4.30 | 17.07 |
| dl | Add | hyper | hyper+attend | 0.47 | 0.40 | 0.55 | **0.0000** | 4143 | 186 | 293 | 82 | 4.30 | 21.87 |
| dh | Add | attend | attend+argues | 0.49 | 0.44 | 0.53 | **0.0000** | 3153 | 167 | 1465 | 390 | 5.03 | 21.02 |
| dl | Add | argues | argues+attend | 0.40 | 0.34 | 0.46 | **0.0000** | 3153 | 167 | 1153 | 231 | 5.03 | 16.69 |
| dh | Add | attend | attend+temper | 0.44 | 0.38 | 0.49 | **0.0000** | 3892 | 108 | 2515 | 337 | 2.70 | 11.82 |
| dl | Add | temper | temper+attend | 0.49 | 0.42 | 0.56 | **0.0000** | 3892 | 108 | 597 | 107 | 2.70 | 15.20 |
| dh | Add | attend | attend+fights | 0.36 | 0.30 | 0.43 | **0.0000** | 4512 | 41 | 3802 | 159 | 0.90 | 4.01 |
| dl | Add | fights | fights+attend | 0.60 | 0.49 | 0.71 | **0.0000** | 4512 | 41 | 129 | 22 | 0.90 | 14.57 |
| dh | Add | attend | attend+steals | 0.44 | 0.36 | 0.51 | **0.0000** | 4619 | 22 | 4003 | 140 | 0.47 | 3.38 |
| dl | Add | steals | steals+attend | 0.66 | 0.52 | 0.80 | **0.0000** | 4619 | 22 | 53 | 10 | 0.47 | 15.87 |
| dh | Add | hyper | hyper+argues | 0.57 | 0.52 | 0.63 | **0.0000** | 3998 | 124 | 840 | 213 | 3.01 | 20.23 |
| dl | Add | argues | argues+hyper | 0.42 | 0.37 | 0.48 | **0.0000** | 3998 | 124 | 1857 | 260 | 3.01 | 12.28 |
| dh | Add | hyper | hyper+temper | 0.53 | 0.47 | 0.58 | **0.0000** | 5020 | 93 | 1527 | 212 | 1.82 | 12.19 |
| dl | Add | temper | temper+hyper | 0.49 | 0.43 | 0.56 | **0.0000** | 5020 | 93 | 999 | 127 | 1.82 | 11.28 |
| dh | Add | hyper | hyper+fights | 0.50 | 0.43 | 0.57 | **0.0000** | 5952 | 34 | 2404 | 124 | 0.57 | 4.91 |
| dl | Add | fights | fights+hyper | 0.56 | 0.45 | 0.67 | **0.0000** | 5952 | 34 | 231 | 22 | 0.57 | 8.70 |
| dh | Add | hyper | hyper+steals | 0.45 | 0.38 | 0.53 | **0.0000** | 6073 | 30 | 2586 | 95 | 0.49 | 3.54 |
| dl | Add | steals | steals+hyper | 0.64 | 0.53 | 0.75 | **0.0000** | 6073 | 30 | 118 | 18 | 0.49 | 13.24 |
| dh | Add | argues | argues+temper | 0.46 | 0.42 | 0.51 | **0.0000** | 4523 | 178 | 1801 | 350 | 3.79 | 16.27 |
| dl | Add | temper | temper+argues | 0.52 | 0.45 | 0.58 | **0.0000** | 4523 | 178 | 368 | 106 | 3.79 | 22.36 |
| dh | Add | argues | argues+fights | 0.44 | 0.37 | 0.50 | **0.0000** | 5032 | 44 | 3260 | 178 | 0.87 | 5.18 |
| dl | Add | fights | fights+argues | 0.65 | 0.55 | 0.76 | **0.0000** | 5032 | 44 | 79 | 20 | 0.87 | 20.20 |
| dh | Add | argues | argues+steals | 0.36 | 0.28 | 0.43 | **0.0000** | 5057 | 39 | 3564 | 124 | 0.77 | 3.36 |
| dl | Add | steals | steals+argues | 0.66 | 0.54 | 0.77 | **0.0000** | 5057 | 39 | 63 | 16 | 0.77 | 20.25 |
| dh | Add | temper | temper+fights | 0.52 | 0.46 | 0.58 | **0.0000** | 6621 | 54 | 1711 | 128 | 0.81 | 6.96 |
| dl | Add | fights | fights+temper | 0.60 | 0.51 | 0.70 | **0.0000** | 6621 | 54 | 151 | 26 | 0.81 | 14.69 |
| dh | Add | temper | temper+steals | 0.42 | 0.34 | 0.50 | **0.0000** | 6655 | 38 | 2018 | 73 | 0.57 | 3.49 |
| dl | Add | steals | steals+temper | 0.67 | 0.58 | 0.77 | **0.0000** | 6655 | 38 | 133 | 26 | 0.57 | 16.35 |
| dh | Add | fights | fights+steals | 0.50 | 0.40 | 0.60 | **0.0000** | 8199 | 33 | 526 | 26 | 0.40 | 4.71 |
| dl | Add | steals | steals+fights | 0.52 | 0.40 | 0.64 | **0.0000** | 8199 | 33 | 265 | 17 | 0.40 | 6.03 |
| dp | Joint Persist | fear+worry | fear+worry | 0.83 | 0.81 | 0.85 | **0.0000** | 5272 | 270 | 631 | 836 | 4.87 | 56.99 |
| dp | Joint Persist | fear+dysph | fear+dysph | 0.81 | 0.78 | 0.84 | **0.0000** | 6497 | 170 | 313 | 268 | 2.55 | 46.13 |
| dp | Joint Persist | fear+worth | fear+worth | 0.83 | 0.80 | 0.86 | **0.0000** | 6501 | 157 | 323 | 294 | 2.36 | 47.65 |
| dp | Joint Persist | fear+attend | fear+attend | 0.89 | 0.87 | 0.90 | **0.0000** | 3950 | 109 | 611 | 768 | 2.69 | 55.69 |
| dp | Joint Persist | fear+hyper | fear+hyper | 0.88 | 0.86 | 0.90 | **0.0000** | 5127 | 88 | 523 | 477 | 1.69 | 47.70 |
| dp | Joint Persist | fear+argues | fear+argues | 0.85 | 0.83 | 0.87 | **0.0000** | 4271 | 137 | 617 | 640 | 3.11 | 50.91 |
| dp | Joint Persist | fear+temper | fear+temper | 0.86 | 0.84 | 0.89 | **0.0000** | 5601 | 111 | 463 | 421 | 1.94 | 47.62 |
| dp | Joint Persist | fear+fights | fear+fights | 0.85 | 0.81 | 0.89 | **0.0000** | 6711 | 53 | 172 | 102 | 0.78 | 37.23 |
| dp | Joint Persist | fear+steals | fear+steals | 0.88 | 0.84 | 0.92 | **0.0000** | 6894 | 26 | 100 | 60 | 0.38 | 37.50 |
| dp | Joint Persist | worry+dysph | worry+dysph | 0.82 | 0.79 | 0.84 | **0.0000** | 5608 | 177 | 411 | 381 | 3.06 | 48.11 |
| dp | Joint Persist | worry+worth | worry+worth | 0.84 | 0.82 | 0.87 | **0.0000** | 5590 | 156 | 399 | 399 | 2.71 | 50.00 |
| dp | Joint Persist | worry+attend | worry+attend | 0.85 | 0.83 | 0.87 | **0.0000** | 3311 | 169 | 791 | 1102 | 4.86 | 58.21 |
| dp | Joint Persist | worry+hyper | worry+hyper | 0.87 | 0.85 | 0.89 | **0.0000** | 4329 | 101 | 657 | 651 | 2.28 | 49.77 |
| dp | Joint Persist | worry+argues | worry+argues | 0.84 | 0.82 | 0.86 | **0.0000** | 3693 | 173 | 802 | 1006 | 4.47 | 55.64 |
| dp | Joint Persist | worry+temper | worry+temper | 0.86 | 0.84 | 0.88 | **0.0000** | 4799 | 123 | 587 | 600 | 2.50 | 50.55 |
| dp | Joint Persist | worry+fights | worry+fights | 0.86 | 0.83 | 0.90 | **0.0000** | 5698 | 51 | 212 | 140 | 0.89 | 39.77 |
| dp | Joint Persist | worry+steals | worry+steals | 0.89 | 0.86 | 0.93 | **0.0000** | 5864 | 31 | 134 | 94 | 0.53 | 41.23 |
| dp | Joint Persist | dysph+worth | dysph+worth | 0.80 | 0.76 | 0.83 | **0.0000** | 7298 | 193 | 289 | 243 | 2.58 | 45.68 |
| dp | Joint Persist | dysph+attend | dysph+attend | 0.86 | 0.84 | 0.89 | **0.0000** | 4345 | 87 | 446 | 388 | 1.96 | 46.52 |
| dp | Joint Persist | dysph+hyper | dysph+hyper | 0.84 | 0.81 | 0.87 | **0.0000** | 5624 | 95 | 341 | 245 | 1.66 | 41.81 |
| dp | Joint Persist | dysph+argues | dysph+argues | 0.85 | 0.82 | 0.87 | **0.0000** | 4783 | 112 | 440 | 386 | 2.29 | 46.73 |
| dp | Joint Persist | dysph+temper | dysph+temper | 0.84 | 0.81 | 0.87 | **0.0000** | 6250 | 125 | 341 | 288 | 1.96 | 45.79 |
| dp | Joint Persist | dysph+fights | dysph+fights | 0.86 | 0.82 | 0.89 | **0.0000** | 7604 | 53 | 152 | 97 | 0.69 | 38.96 |
| dp | Joint Persist | dysph+steals | dysph+steals | 0.81 | 0.75 | 0.87 | **0.0000** | 7778 | 45 | 98 | 47 | 0.58 | 32.41 |
| dp | Joint Persist | worth+attend | worth+attend | 0.88 | 0.86 | 0.90 | **0.0000** | 4362 | 85 | 447 | 447 | 1.91 | 50.00 |
| dp | Joint Persist | worth+hyper | worth+hyper | 0.88 | 0.85 | 0.90 | **0.0000** | 5653 | 72 | 353 | 284 | 1.26 | 44.58 |
| dp | Joint Persist | worth+argues | worth+argues | 0.88 | 0.85 | 0.90 | **0.0000** | 4738 | 92 | 407 | 399 | 1.90 | 49.50 |
| dp | Joint Persist | worth+temper | worth+temper | 0.87 | 0.85 | 0.89 | **0.0000** | 6222 | 86 | 332 | 275 | 1.36 | 45.30 |
| dp | Joint Persist | worth+fights | worth+fights | 0.84 | 0.80 | 0.89 | **0.0000** | 7531 | 41 | 138 | 71 | 0.54 | 33.97 |
| dp | Joint Persist | worth+steals | worth+steals | 0.84 | 0.79 | 0.89 | **0.0000** | 7748 | 34 | 100 | 49 | 0.44 | 32.89 |
| dp | Joint Persist | attend+hyper | attend+hyper | 0.89 | 0.88 | 0.91 | **0.0000** | 4143 | 186 | 916 | 1686 | 4.30 | 64.80 |
| dp | Joint Persist | attend+argues | attend+argues | 0.88 | 0.86 | 0.89 | **0.0000** | 3153 | 167 | 949 | 1708 | 5.03 | 64.28 |
| dp | Joint Persist | attend+temper | attend+temper | 0.88 | 0.87 | 0.90 | **0.0000** | 3892 | 108 | 749 | 911 | 2.70 | 54.88 |
| dp | Joint Persist | attend+fights | attend+fights | 0.89 | 0.86 | 0.91 | **0.0000** | 4512 | 41 | 321 | 230 | 0.90 | 41.74 |
| dp | Joint Persist | attend+steals | attend+steals | 0.93 | 0.91 | 0.95 | **0.0000** | 4619 | 22 | 196 | 173 | 0.47 | 46.88 |
| dp | Joint Persist | hyper+argues | hyper+argues | 0.88 | 0.87 | 0.90 | **0.0000** | 3998 | 124 | 839 | 1085 | 3.01 | 56.39 |
| dp | Joint Persist | hyper+temper | hyper+temper | 0.89 | 0.87 | 0.91 | **0.0000** | 5020 | 93 | 602 | 636 | 1.82 | 51.37 |
| dp | Joint Persist | hyper+fights | hyper+fights | 0.90 | 0.88 | 0.93 | **0.0000** | 5952 | 34 | 268 | 181 | 0.57 | 40.31 |
| dp | Joint Persist | hyper+steals | hyper+steals | 0.91 | 0.88 | 0.93 | **0.0000** | 6073 | 30 | 172 | 124 | 0.49 | 41.89 |
| dp | Joint Persist | argues+temper | argues+temper | 0.86 | 0.84 | 0.88 | **0.0000** | 4523 | 178 | 830 | 1060 | 3.79 | 56.08 |
| dp | Joint Persist | argues+fights | argues+fights | 0.89 | 0.87 | 0.92 | **0.0000** | 5032 | 44 | 347 | 256 | 0.87 | 42.45 |
| dp | Joint Persist | argues+steals | argues+steals | 0.91 | 0.88 | 0.93 | **0.0000** | 5057 | 39 | 188 | 165 | 0.77 | 46.74 |
| dp | Joint Persist | temper+fights | temper+fights | 0.89 | 0.86 | 0.91 | **0.0000** | 6621 | 54 | 306 | 219 | 0.81 | 41.71 |
| dp | Joint Persist | temper+steals | temper+steals | 0.88 | 0.85 | 0.92 | **0.0000** | 6655 | 38 | 164 | 109 | 0.57 | 39.93 |
| dp | Joint Persist | fights+steals | fights+steals | 0.86 | 0.81 | 0.90 | **0.0000** | 8199 | 33 | 98 | 52 | 0.40 | 34.67 |
| cg | Shift | fear | worry | 0.03 | -.04 | 0.10 | 0.4010 | 4855 | 687 | 481 | 76 | 12.40 | 13.64 |
| bj | Shift | worry | fear | 0.03 | -.04 | 0.11 | 0.3919 | 5336 | 206 | 1581 | 69 | 3.72 | 4.18 |
| cg | Shift | fear | dysph | -.08 | -.16 | -.01 | **0.0254** | 6365 | 302 | 1396 | 47 | 4.53 | 3.26 |
| bj | Shift | dysph | fear | -.01 | -.09 | 0.07 | 0.8475 | 6092 | 575 | 481 | 44 | 8.62 | 8.38 |
| cg | Shift | fear | worth | -.05 | -.13 | 0.02 | 0.1559 | 6372 | 286 | 1358 | 49 | 4.30 | 3.48 |
| bj | Shift | worth | fear | -.01 | -.09 | 0.06 | 0.7231 | 6067 | 591 | 489 | 45 | 8.88 | 8.43 |
| cg | Shift | fear | attend | -.12 | -.19 | -.04 | **0.0018** | 3501 | 558 | 584 | 61 | 13.75 | 9.46 |
| bj | Shift | attend | fear | -.36 | -.42 | -.30 | **0.0000** | 3765 | 294 | 3073 | 60 | 7.24 | 1.92 |
| cg | Shift | fear | hyper | -.16 | -.23 | -.09 | **0.0000** | 4794 | 421 | 978 | 46 | 8.07 | 4.49 |
| bj | Shift | hyper | fear | -.22 | -.28 | -.16 | **0.0000** | 4747 | 468 | 1894 | 83 | 8.97 | 4.20 |
| cg | Shift | fear | argues | -.20 | -.27 | -.14 | **0.0000** | 3713 | 695 | 704 | 63 | 15.77 | 8.21 |
| bj | Shift | argues | fear | -.26 | -.32 | -.20 | **0.0000** | 4098 | 310 | 2705 | 79 | 7.03 | 2.84 |
| cg | Shift | fear | temper | -.16 | -.23 | -.09 | **0.0000** | 5256 | 456 | 1089 | 51 | 7.98 | 4.47 |
| bj | Shift | temper | fear | -.13 | -.19 | -.07 | **0.0000** | 5225 | 487 | 1399 | 81 | 8.53 | 5.47 |
| cg | Shift | fear | fights | -.18 | -.28 | -.08 | **0.0003** | 6619 | 145 | 1734 | 16 | 2.14 | 0.91 |
| bj | Shift | fights | fear | -.11 | -.19 | -.03 | **0.0085** | 6012 | 752 | 397 | 31 | 11.12 | 7.24 |
| cg | Shift | fear | steals | -.28 | -.39 | -.16 | **0.0000** | 6796 | 124 | 1856 | 8 | 1.79 | 0.43 |
| bj | Shift | steals | fear | -.11 | -.20 | -.01 | **0.0242** | 6121 | 799 | 252 | 20 | 11.55 | 7.35 |
| cg | Shift | worry | dysph | -.02 | -.09 | 0.06 | 0.6108 | 5624 | 161 | 2265 | 60 | 2.78 | 2.58 |
| bj | Shift | dysph | worry | 0.03 | -.05 | 0.11 | 0.4648 | 4898 | 887 | 261 | 53 | 15.33 | 16.88 |
| cg | Shift | worry | worth | -.03 | -.10 | 0.05 | 0.4696 | 5578 | 168 | 2258 | 61 | 2.92 | 2.63 |
| bj | Shift | worth | worry | -.00 | -.08 | 0.08 | 0.9809 | 4848 | 898 | 298 | 55 | 15.63 | 15.58 |
| cg | Shift | worry | attend | -.25 | -.32 | -.18 | **0.0000** | 3071 | 409 | 1161 | 63 | 11.75 | 5.15 |
| bj | Shift | attend | worry | -.40 | -.45 | -.35 | **0.0000** | 3022 | 458 | 2528 | 91 | 13.16 | 3.47 |
| cg | Shift | worry | hyper | -.27 | -.34 | -.21 | **0.0000** | 4098 | 332 | 1760 | 49 | 7.49 | 2.71 |
| bj | Shift | hyper | worry | -.28 | -.34 | -.23 | **0.0000** | 3721 | 709 | 1557 | 112 | 16.00 | 6.71 |
| cg | Shift | worry | argues | -.25 | -.31 | -.19 | **0.0000** | 3337 | 529 | 1227 | 82 | 13.68 | 6.26 |
| bj | Shift | argues | worry | -.31 | -.36 | -.26 | **0.0000** | 3373 | 493 | 2127 | 106 | 12.75 | 4.75 |
| cg | Shift | worry | temper | -.19 | -.25 | -.12 | **0.0000** | 4581 | 341 | 1862 | 68 | 6.93 | 3.52 |
| bj | Shift | temper | worry | -.16 | -.21 | -.10 | **0.0000** | 4173 | 749 | 1065 | 112 | 15.22 | 9.52 |
| cg | Shift | worry | fights | -.22 | -.31 | -.12 | **0.0000** | 5642 | 107 | 2746 | 19 | 1.86 | 0.69 |
| bj | Shift | fights | worry | -.12 | -.20 | -.04 | **0.0028** | 4697 | 1052 | 307 | 43 | 18.30 | 12.29 |
| cg | Shift | worry | steals | -.21 | -.31 | -.11 | **0.0000** | 5800 | 95 | 2871 | 18 | 1.61 | 0.62 |
| bj | Shift | steals | worry | -.20 | -.30 | -.11 | **0.0000** | 4795 | 1100 | 187 | 17 | 18.66 | 8.33 |
| cg | Shift | dysph | worth | -.02 | -.11 | 0.08 | 0.7316 | 7155 | 336 | 550 | 24 | 4.49 | 4.18 |
| bj | Shift | worth | dysph | 0.03 | -.05 | 0.12 | 0.4340 | 7133 | 358 | 585 | 34 | 4.78 | 5.49 |
| cg | Shift | dysph | attend | -.12 | -.21 | -.02 | **0.0142** | 3763 | 669 | 245 | 27 | 15.09 | 9.93 |
| bj | Shift | attend | dysph | -.35 | -.42 | -.27 | **0.0000** | 4248 | 184 | 3641 | 37 | 4.15 | 1.01 |
| cg | Shift | dysph | hyper | -.10 | -.18 | -.01 | **0.0226** | 5219 | 500 | 489 | 31 | 8.74 | 5.96 |
| bj | Shift | hyper | dysph | -.18 | -.24 | -.11 | **0.0000** | 5410 | 309 | 2324 | 67 | 5.40 | 2.80 |
| cg | Shift | dysph | argues | -.21 | -.30 | -.12 | **0.0000** | 4068 | 827 | 259 | 21 | 16.89 | 7.50 |
| bj | Shift | argues | dysph | -.18 | -.25 | -.11 | **0.0000** | 4718 | 177 | 3156 | 59 | 3.62 | 1.84 |
| cg | Shift | dysph | temper | -.06 | -.14 | 0.03 | 0.1936 | 5842 | 533 | 445 | 32 | 8.36 | 6.71 |
| bj | Shift | temper | dysph | -.12 | -.19 | -.05 | **0.0009** | 6058 | 317 | 1680 | 55 | 4.97 | 3.17 |
| cg | Shift | dysph | fights | -.10 | -.21 | 0.01 | 0.0883 | 7496 | 161 | 846 | 11 | 2.10 | 1.28 |
| bj | Shift | fights | dysph | -.02 | -.11 | 0.06 | 0.5636 | 7095 | 562 | 423 | 30 | 7.34 | 6.62 |
| cg | Shift | dysph | steals | -.11 | -.23 | 0.02 | 0.0925 | 7708 | 115 | 953 | 8 | 1.47 | 0.83 |
| bj | Shift | steals | dysph | -.04 | -.14 | 0.06 | 0.4162 | 7234 | 589 | 269 | 18 | 7.53 | 6.27 |
| cg | Shift | worth | attend | -.19 | -.29 | -.09 | **0.0002** | 3768 | 679 | 238 | 19 | 15.27 | 7.39 |
| bj | Shift | attend | worth | -.25 | -.32 | -.17 | **0.0000** | 4285 | 162 | 3568 | 50 | 3.64 | 1.38 |
| cg | Shift | worth | hyper | -.13 | -.21 | -.04 | **0.0035** | 5209 | 516 | 486 | 28 | 9.01 | 5.45 |
| bj | Shift | hyper | worth | -.17 | -.24 | -.11 | **0.0000** | 5421 | 304 | 2274 | 66 | 5.31 | 2.82 |
| cg | Shift | worth | argues | -.08 | -.16 | 0.00 | 0.0544 | 4013 | 817 | 300 | 45 | 16.92 | 13.04 |
| bj | Shift | argues | worth | -.18 | -.25 | -.10 | **0.0000** | 4665 | 165 | 3179 | 56 | 3.42 | 1.73 |
| cg | Shift | worth | temper | -.05 | -.13 | 0.03 | 0.2475 | 5767 | 541 | 505 | 39 | 8.58 | 7.17 |
| bj | Shift | temper | worth | -.09 | -.16 | -.02 | **0.0138** | 6013 | 295 | 1698 | 59 | 4.68 | 3.36 |
| cg | Shift | worth | fights | -.02 | -.12 | 0.08 | 0.7062 | 7405 | 167 | 923 | 19 | 2.21 | 2.02 |
| bj | Shift | fights | worth | 0.03 | -.05 | 0.11 | 0.4753 | 7053 | 519 | 455 | 38 | 6.85 | 7.71 |
| cg | Shift | worth | steals | -.07 | -.19 | 0.04 | 0.2118 | 7658 | 124 | 991 | 11 | 1.59 | 1.10 |
| bj | Shift | steals | worth | -.02 | -.12 | 0.08 | 0.7432 | 7220 | 562 | 264 | 19 | 7.22 | 6.71 |
| cg | Shift | attend | hyper | -.10 | -.19 | -.01 | **0.0304** | 4211 | 118 | 1875 | 35 | 2.73 | 1.83 |
| bj | Shift | hyper | attend | -.04 | -.12 | 0.05 | 0.3951 | 3816 | 513 | 336 | 39 | 11.85 | 10.40 |
| cg | Shift | attend | argues | -.33 | -.39 | -.27 | **0.0000** | 2934 | 386 | 1784 | 71 | 11.63 | 3.83 |
| bj | Shift | argues | attend | -.28 | -.35 | -.22 | **0.0000** | 2956 | 364 | 1326 | 58 | 10.96 | 4.19 |
| cg | Shift | attend | temper | -.27 | -.33 | -.20 | **0.0000** | 3775 | 225 | 2792 | 60 | 5.63 | 2.10 |
| bj | Shift | temper | attend | -.18 | -.25 | -.10 | **0.0000** | 3449 | 551 | 650 | 54 | 13.78 | 7.67 |
| cg | Shift | attend | fights | -.17 | -.27 | -.06 | **0.0023** | 4502 | 51 | 3940 | 21 | 1.12 | 0.53 |
| bj | Shift | fights | attend | -.03 | -.14 | 0.08 | 0.6002 | 3818 | 735 | 129 | 22 | 16.14 | 14.57 |
| cg | Shift | attend | steals | -.14 | -.27 | -.00 | 0.0493 | 4615 | 26 | 4131 | 12 | 0.56 | 0.29 |
| bj | Shift | steals | attend | -.13 | -.29 | 0.02 | 0.0998 | 3859 | 782 | 57 | 6 | 16.85 | 9.52 |
| cg | Shift | hyper | argues | -.23 | -.29 | -.17 | **0.0000** | 3519 | 603 | 979 | 74 | 14.63 | 7.03 |
| bj | Shift | argues | hyper | -.16 | -.24 | -.09 | **0.0000** | 3903 | 219 | 2055 | 62 | 5.31 | 2.93 |
| cg | Shift | hyper | temper | -.13 | -.19 | -.06 | **0.0001** | 4764 | 349 | 1663 | 76 | 6.83 | 4.37 |
| bj | Shift | temper | hyper | -.08 | -.15 | -.01 | 0.0318 | 4732 | 381 | 1062 | 64 | 7.45 | 5.68 |
| cg | Shift | hyper | fights | -.16 | -.26 | -.07 | **0.0012** | 5891 | 95 | 2509 | 19 | 1.59 | 0.75 |
| bj | Shift | fights | hyper | 0.03 | -.06 | 0.13 | 0.4820 | 5405 | 581 | 225 | 28 | 9.71 | 11.07 |
| cg | Shift | hyper | steals | 0.06 | -.05 | 0.16 | 0.3068 | 6055 | 48 | 2654 | 27 | 0.79 | 1.01 |
| bj | Shift | steals | hyper | -.11 | -.24 | 0.02 | 0.0927 | 5494 | 609 | 128 | 8 | 9.98 | 5.88 |
| cg | Shift | argues | temper | -.03 | -.11 | 0.05 | 0.4943 | 4558 | 143 | 2092 | 59 | 3.04 | 2.74 |
| bj | Shift | temper | argues | 0.01 | -.06 | 0.09 | 0.7683 | 4040 | 661 | 405 | 69 | 14.06 | 14.56 |
| cg | Shift | argues | fights | -.25 | -.39 | -.12 | **0.0002** | 5034 | 42 | 3430 | 8 | 0.83 | 0.23 |
| bj | Shift | fights | argues | 0.07 | -.04 | 0.18 | 0.2178 | 4149 | 927 | 76 | 23 | 18.26 | 23.23 |
| cg | Shift | argues | steals | -.16 | -.31 | -.02 | **0.0238** | 5068 | 28 | 3679 | 9 | 0.55 | 0.24 |
| bj | Shift | steals | argues | 0.04 | -.08 | 0.17 | 0.4982 | 4154 | 942 | 62 | 17 | 18.49 | 21.52 |
| cg | Shift | temper | fights | 0.07 | -.03 | 0.18 | 0.1628 | 6610 | 65 | 1814 | 25 | 0.97 | 1.36 |
| bj | Shift | Fights | temper | 0.03 | -.08 | 0.14 | 0.5720 | 6044 | 631 | 158 | 19 | 9.45 | 10.73 |
| cg | Shift | Temper | steals | -.02 | -.12 | 0.09 | 0.7653 | 6624 | 69 | 2071 | 20 | 1.03 | 0.96 |
| bj | Shift | steals | temper | -.01 | -.12 | 0.11 | 0.9017 | 6042 | 651 | 144 | 15 | 9.73 | 9.43 |
| cg | Shift | Fights | steals | -.05 | -.18 | 0.09 | 0.5032 | 8098 | 134 | 545 | 7 | 1.63 | 1.27 |
| bj | Shift | Steals | fights | 0.08 | -.04 | 0.21 | 0.1894 | 8021 | 211 | 271 | 11 | 2.56 | 3.90 |
| co | Subtract x | fear+worry | worry | 0.18 | 0.13 | 0.23 | **0.0000** | 4855 | 687 | 1174 | 293 | 12.40 | 19.97 |
| co | Subtract x | fear+dysph | dysph | 0.23 | 0.15 | 0.31 | **0.0000** | 6365 | 302 | 519 | 62 | 4.53 | 10.67 |
| co | Subtract x | fear+worth | worth | 0.28 | 0.20 | 0.35 | **0.0000** | 6372 | 286 | 544 | 73 | 4.30 | 11.83 |
| co | Subtract x | fear+attend | attend | 0.32 | 0.27 | 0.36 | **0.0000** | 3501 | 558 | 979 | 400 | 13.75 | 29.01 |
| co | Subtract x | fear+hyper | hyper | 0.37 | 0.32 | 0.42 | **0.0000** | 4794 | 421 | 769 | 231 | 8.07 | 23.10 |
| co | Subtract x | fear+argues | argues | 0.19 | 0.14 | 0.24 | **0.0000** | 3713 | 695 | 947 | 310 | 15.77 | 24.66 |
| co | Subtract x | fear+temper | temper | 0.26 | 0.20 | 0.32 | **0.0000** | 5256 | 456 | 728 | 156 | 7.98 | 17.65 |
| co | Subtract x | fear+fights | fights | 0.44 | 0.35 | 0.53 | **0.0000** | 6619 | 145 | 236 | 38 | 2.14 | 13.87 |
| co | Subtract x | fear+steals | Steals | 0.50 | 0.40 | 0.60 | **0.0000** | 6796 | 124 | 133 | 27 | 1.79 | 16.88 |
| co | Subtract x | worry+dysph | Dysph | 0.26 | 0.18 | 0.34 | **0.0000** | 5624 | 161 | 732 | 60 | 2.78 | 7.58 |
| co | Subtract x | worry+worth | Worth | 0.32 | 0.24 | 0.39 | **0.0000** | 5578 | 168 | 722 | 76 | 2.92 | 9.52 |
| co | Subtract x | worry+attend | attend | 0.28 | 0.23 | 0.32 | **0.0000** | 3071 | 409 | 1455 | 438 | 11.75 | 23.14 |
| co | Subtract x | worry+hyper | hyper | 0.32 | 0.27 | 0.38 | **0.0000** | 4098 | 332 | 1062 | 246 | 7.49 | 18.81 |
| co | Subtract x | worry+argues | argues | 0.16 | 0.11 | 0.21 | **0.0000** | 3337 | 529 | 1441 | 367 | 13.68 | 20.30 |
| co | Subtract x | worry+temper | temper | 0.20 | 0.14 | 0.26 | **0.0000** | 4581 | 341 | 1034 | 153 | 6.93 | 12.89 |
| co | Subtract x | worry+fights | fights | 0.36 | 0.26 | 0.46 | **0.0000** | 5642 | 107 | 321 | 31 | 1.86 | 8.81 |
| co | Subtract x | worry+steals | steals | 0.38 | 0.27 | 0.50 | **0.0000** | 5800 | 95 | 207 | 21 | 1.61 | 9.21 |
| co | Subtract x | dysph+worth | worth | 0.38 | 0.31 | 0.44 | **0.0000** | 7155 | 336 | 439 | 93 | 4.49 | 17.48 |
| co | Subtract x | dysph+attend | attend | 0.37 | 0.32 | 0.42 | **0.0000** | 3763 | 669 | 535 | 299 | 15.09 | 35.85 |
| co | Subtract x | dysph+hyper | hyper | 0.41 | 0.35 | 0.47 | **0.0000** | 5219 | 500 | 416 | 170 | 8.74 | 29.01 |
| co | Subtract x | dysph+argues | argues | 0.27 | 0.22 | 0.32 | **0.0000** | 4068 | 827 | 559 | 267 | 16.89 | 32.32 |
| co | Subtract x | dysph+temper | temper | 0.29 | 0.23 | 0.35 | **0.0000** | 5842 | 533 | 500 | 129 | 8.36 | 20.51 |
| co | Subtract x | dysph+fights | fights | 0.49 | 0.41 | 0.58 | **0.0000** | 7496 | 161 | 206 | 43 | 2.10 | 17.27 |
| co | Subtract x | dysph+steals | steals | 0.52 | 0.42 | 0.62 | **0.0000** | 7708 | 115 | 120 | 25 | 1.47 | 17.24 |
| co | Subtract x | worth+attend | attend | 0.35 | 0.30 | 0.40 | **0.0000** | 3768 | 679 | 584 | 310 | 15.27 | 34.68 |
| co | Subtract x | worth+hyper | hyper | 0.39 | 0.33 | 0.44 | **0.0000** | 5209 | 516 | 460 | 177 | 9.01 | 27.79 |
| co | Subtract x | worth+argues | argues | 0.26 | 0.21 | 0.32 | **0.0000** | 4013 | 817 | 550 | 256 | 16.92 | 31.76 |
| co | Subtract x | worth+temper | temper | 0.28 | 0.22 | 0.34 | **0.0000** | 5767 | 541 | 483 | 124 | 8.58 | 20.43 |
| co | Subtract x | worth+fights | fights | 0.43 | 0.34 | 0.53 | **0.0000** | 7405 | 167 | 178 | 31 | 2.21 | 14.83 |
| co | Subtract x | worth+steals | steals | 0.55 | 0.46 | 0.65 | **0.0000** | 7658 | 124 | 119 | 30 | 1.59 | 20.13 |
| co | Subtract x | attend+hyper | hyper | 0.05 | -.02 | 0.13 | 0.1694 | 4211 | 118 | 2516 | 86 | 2.73 | 3.31 |
| co | Subtract x | attend+argues | argues | -.00 | -.06 | 0.05 | 0.8951 | 2934 | 386 | 2351 | 306 | 11.63 | 11.52 |
| co | Subtract x | attend+temper | temper | 0.08 | 0.01 | 0.14 | **0.0234** | 3775 | 225 | 1540 | 120 | 5.63 | 7.23 |
| co | Subtract x | attend+fights | fights | 0.28 | 0.16 | 0.40 | **0.0000** | 4502 | 51 | 530 | 21 | 1.12 | 3.81 |
| co | Subtract x | attend+steals | steals | 0.46 | 0.33 | 0.59 | **0.0000** | 4615 | 26 | 351 | 18 | 0.56 | 4.88 |
| co | Subtract x | hyper+argues | argues | 0.14 | 0.09 | 0.19 | **0.0000** | 3519 | 603 | 1530 | 394 | 14.63 | 20.48 |
| co | Subtract x | hyper+temper | temper | 0.22 | 0.16 | 0.28 | **0.0000** | 4764 | 349 | 1074 | 164 | 6.83 | 13.25 |
| co | Subtract x | hyper+fights | fights | 0.36 | 0.26 | 0.46 | **0.0000** | 5891 | 95 | 416 | 33 | 1.59 | 7.35 |
| co | Subtract x | hyper+steals | steals | 0.55 | 0.45 | 0.65 | **0.0000** | 6055 | 48 | 266 | 30 | 0.79 | 10.14 |
| co | Subtract x | argues+temper | temper | 0.21 | 0.14 | 0.27 | **0.0000** | 4558 | 143 | 1772 | 118 | 3.04 | 6.24 |
| co | Subtract x | argues+fights | fights | 0.27 | 0.14 | 0.40 | **0.0001** | 5034 | 42 | 586 | 17 | 0.83 | 2.82 |
| co | Subtract x | argues+steals | steals | 0.44 | 0.31 | 0.57 | **0.0000** | 5068 | 28 | 337 | 16 | 0.55 | 4.53 |
| co | Subtract x | temper+fights | fights | 0.43 | 0.34 | 0.53 | **0.0000** | 6610 | 65 | 490 | 35 | 0.97 | 6.67 |
| co | Subtract x | temper+steals | steals | 0.52 | 0.42 | 0.61 | **0.0000** | 6624 | 69 | 243 | 30 | 1.03 | 10.99 |
| co | Subtract x | fights+steals | steals | 0.60 | 0.51 | 0.68 | **0.0000** | 8098 | 134 | 114 | 36 | 1.63 | 24.00 |
| bn | Subtract y | worry+fear | fear | 0.21 | 0.14 | 0.28 | **0.0000** | 5336 | 206 | 1354 | 113 | 3.72 | 7.70 |
| bn | Subtract y | dysph+fear | fear | 0.32 | 0.26 | 0.38 | **0.0000** | 6092 | 575 | 445 | 136 | 8.62 | 23.41 |
| bn | Subtract y | worth+fear | fear | 0.29 | 0.24 | 0.35 | **0.0000** | 6067 | 591 | 481 | 136 | 8.88 | 22.04 |
| bn | Subtract y | attend+fear | fear | -.04 | -.11 | 0.03 | 0.2743 | 3765 | 294 | 1291 | 88 | 7.24 | 6.38 |
| bn | Subtract y | hyper+fear | fear | 0.13 | 0.06 | 0.19 | **0.0001** | 4747 | 468 | 868 | 132 | 8.97 | 13.20 |
| bn | Subtract y | argues+fear | fear | 0.17 | 0.11 | 0.24 | **0.0000** | 4098 | 310 | 1107 | 150 | 7.03 | 11.93 |
| bn | Subtract y | temper+fear | fear | 0.25 | 0.19 | 0.31 | **0.0000** | 5225 | 487 | 724 | 160 | 8.53 | 18.10 |
| bn | Subtract y | fights+fear | fear | 0.23 | 0.16 | 0.31 | **0.0000** | 6012 | 752 | 208 | 66 | 11.12 | 24.09 |
| bn | Subtract y | steals+fear | Fear | 0.20 | 0.11 | 0.29 | **0.0000** | 6121 | 799 | 122 | 38 | 11.55 | 23.75 |
| bn | Subtract y | dysph+worry | Worry | 0.25 | 0.19 | 0.30 | **0.0000** | 4898 | 887 | 564 | 228 | 15.33 | 28.79 |
| bn | Subtract y | worth+worry | Worry | 0.21 | 0.15 | 0.26 | **0.0000** | 4848 | 898 | 586 | 212 | 15.63 | 26.57 |
| bn | Subtract y | attend+worry | worry | -.07 | -.13 | -.02 | **0.0101** | 3022 | 458 | 1689 | 204 | 13.16 | 10.78 |
| bn | Subtract y | hyper+worry | worry | 0.09 | 0.04 | 0.14 | **0.0008** | 3721 | 709 | 1046 | 262 | 16.00 | 20.03 |
| bn | Subtract y | argues+worry | worry | 0.03 | -.02 | 0.09 | 0.2198 | 3373 | 493 | 1556 | 252 | 12.75 | 13.94 |
| bn | Subtract y | temper+worry | worry | 0.15 | 0.10 | 0.20 | **0.0000** | 4173 | 749 | 925 | 262 | 15.22 | 22.07 |
| bn | Subtract y | fights+worry | worry | 0.22 | 0.15 | 0.29 | **0.0000** | 4697 | 1052 | 236 | 116 | 18.30 | 32.95 |
| bn | Subtract y | steals+worry | worry | 0.21 | 0.13 | 0.28 | **0.0000** | 4795 | 1100 | 152 | 76 | 18.66 | 33.33 |
| bn | Subtract y | worth+dysph | dysph | 0.28 | 0.21 | 0.35 | **0.0000** | 7133 | 358 | 460 | 72 | 4.78 | 13.53 |
| bn | Subtract y | attend+dysph | dysph | 0.14 | 0.06 | 0.23 | **0.0007** | 4248 | 184 | 776 | 58 | 4.15 | 6.95 |
| bn | Subtract y | hyper+dysph | dysph | 0.25 | 0.18 | 0.33 | **0.0000** | 5410 | 309 | 509 | 77 | 5.40 | 13.14 |
| bn | Subtract y | argues+dysph | dysph | 0.14 | 0.05 | 0.22 | **0.0016** | 4718 | 177 | 776 | 50 | 3.62 | 6.05 |
| bn | Subtract y | temper+dysph | dysph | 0.28 | 0.21 | 0.35 | **0.0000** | 6058 | 317 | 544 | 85 | 4.97 | 13.51 |
| bn | Subtract y | fights+dysph | dysph | 0.28 | 0.20 | 0.36 | **0.0000** | 7095 | 562 | 197 | 52 | 7.34 | 20.88 |
| bn | Subtract y | steals+dysph | dysph | 0.29 | 0.20 | 0.39 | **0.0000** | 7234 | 589 | 111 | 34 | 7.53 | 23.45 |
| bn | Subtract y | attend+worth | worth | 0.12 | 0.03 | 0.20 | **0.0087** | 4285 | 162 | 844 | 50 | 3.64 | 5.59 |
| bn | Subtract y | hyper+worth | worth | 0.24 | 0.17 | 0.31 | **0.0000** | 5421 | 304 | 559 | 78 | 5.31 | 12.24 |
| bn | Subtract y | argues+worth | worth | 0.23 | 0.15 | 0.31 | **0.0000** | 4665 | 165 | 742 | 64 | 3.42 | 7.94 |
| bn | Subtract y | temper+worth | worth | 0.34 | 0.27 | 0.40 | **0.0000** | 6013 | 295 | 515 | 92 | 4.68 | 15.16 |
| bn | Subtract y | fights+worth | worth | 0.40 | 0.32 | 0.48 | **0.0000** | 7053 | 519 | 149 | 60 | 6.85 | 28.71 |
| bn | Subtract y | steals+worth | worth | 0.32 | 0.23 | 0.41 | **0.0000** | 7220 | 562 | 112 | 37 | 7.22 | 24.83 |
| bn | Subtract y | hyper+attend | attend | 0.21 | 0.17 | 0.26 | **0.0000** | 3816 | 513 | 2079 | 523 | 11.85 | 20.10 |
| bn | Subtract y | argues+attend | attend | 0.15 | 0.10 | 0.20 | **0.0000** | 2956 | 364 | 2232 | 425 | 10.96 | 16.00 |
| bn | Subtract y | temper+attend | attend | 0.30 | 0.25 | 0.35 | **0.0000** | 3449 | 551 | 1197 | 463 | 13.78 | 27.89 |
| bn | Subtract y | fights+attend | attend | 0.44 | 0.38 | 0.49 | **0.0000** | 3818 | 735 | 304 | 247 | 16.14 | 44.83 |
| bn | Subtract y | steals+attend | attend | 0.33 | 0.26 | 0.39 | **0.0000** | 3859 | 782 | 227 | 142 | 16.85 | 38.48 |
| bn | Subtract y | argues+hyper | hyper | 0.26 | 0.20 | 0.32 | **0.0000** | 3903 | 219 | 1697 | 227 | 5.31 | 11.80 |
| bn | Subtract y | temper+hyper | hyper | 0.37 | 0.32 | 0.42 | **0.0000** | 4732 | 381 | 976 | 262 | 7.45 | 21.16 |
| bn | Subtract y | fights+hyper | hyper | 0.43 | 0.37 | 0.49 | **0.0000** | 5405 | 581 | 297 | 152 | 9.71 | 33.85 |
| bn | Subtract y | steals+hyper | hyper | 0.40 | 0.33 | 0.47 | **0.0000** | 5494 | 609 | 196 | 100 | 9.98 | 33.78 |
| bn | Subtract y | temper+argues | argues | 0.23 | 0.19 | 0.28 | **0.0000** | 4040 | 661 | 1425 | 465 | 14.06 | 24.60 |
| bn | Subtract y | fights+argues | argues | 0.35 | 0.30 | 0.41 | **0.0000** | 4149 | 927 | 354 | 249 | 18.26 | 41.29 |
| bn | Subtract y | steals+argues | argues | 0.24 | 0.17 | 0.31 | **0.0000** | 4154 | 942 | 232 | 121 | 18.49 | 34.28 |
| bn | Subtract y | fights+temper | temper | 0.41 | 0.35 | 0.46 | **0.0000** | 6044 | 631 | 360 | 165 | 9.45 | 31.43 |
| bn | Subtract y | steals+temper | temper | 0.28 | 0.20 | 0.35 | **0.0000** | 6042 | 651 | 206 | 67 | 9.73 | 24.54 |
| bn | Subtract y | steals+fights | fights | 0.37 | 0.26 | 0.47 | **0.0000** | 8021 | 211 | 129 | 21 | 2.56 | 14.00 |

| Supplemental Table S3H. Tetrachoric correlations from 4 x 4 pairwise analyses of all 90 combinations of the 10 selected psychological problems with every other problem to quantify the associations of each baseline problem with outcomes in the **first annual follow-up** that define four parsed paths traditionally conflated in estimates of change: ***add path*** (x_1_ at baseline and x_2_ + y_2_ at follow-up); ***joint persist path*** (x_1_ + y_1_ at baseline and x_2_ + y_2_ at follow-up); ***shift path*** (only x_1_ at baseline and only y_2_ at follow-up); ***subtract x path*** (x_1_ + y_1_ at baseline and only y_2_ at follow-up); and ***subtract y path*** (x_1_ + y_1_ at baseline and only x_2_ at follow-up). Results are for problems dichotomized at the **high rating cut (0 or 1 vs 2).** r_t_ = tetrachoric correlation. –CL and +CL = lower and upper 95% confidence intervals for the tetrachoric correlation. c00 = number without the predictor or the outcome; c01 = number without predictor with the outcome; c10 = number with the predictor without the outcome; c11 = number with the predictor and the outcome. Base% = percent of the outcome only x_2_ among children with neither x_1_ nor the other member of the pair, y_1_, at baseline. Obs% = Observed percent of outcomes on the indicated path. Raw P values significant after FDR adjustment in **bold**. | | | | | | | | | | | | | |
| --- | --- | --- | --- | --- | --- | --- | --- | --- | --- | --- | --- | --- | --- |
| prefix | Parsed paths | Predictor | Outcome | r_t_ | -CL | +CL | P | c00 | c01 | c10 | c11 | Base% | Obs% |
| dh | Add | fear | fear+worry | 0.38 | 0.22 | 0.54 | **0.0000** | 8644 | 75 | 87 | 7 | 0.86 | 7.45 |
| dl | Add | worry | worry+fear | 0.58 | 0.49 | 0.66 | **0.0000** | 8644 | 75 | 227 | 35 | 0.86 | 13.36 |
| dh | Add | fear | fear+dysph | 0.47 | 0.30 | 0.63 | **0.0000** | 8916 | 20 | 202 | 7 | 0.22 | 3.35 |
| dl | Add | dysph | dysph+fear | 0.44 | 0.18 | 0.70 | **0.0010** | 8916 | 20 | 43 | 2 | 0.22 | 4.44 |
| dh | Add | fear | fear+worth | 0.45 | 0.26 | 0.64 | **0.0000** | 8909 | 15 | 196 | 5 | 0.17 | 2.49 |
| dl | Add | worth | worth+fear | 0.52 | 0.30 | 0.75 | **0.0000** | 8909 | 15 | 54 | 3 | 0.17 | 5.26 |
| dh | Add | fear | fear+attend | 0.46 | 0.30 | 0.63 | **0.0000** | 7940 | 32 | 115 | 7 | 0.40 | 5.74 |
| dl | Add | attend | attend+fear | 0.56 | 0.48 | 0.64 | **0.0000** | 7940 | 32 | 955 | 54 | 0.40 | 5.35 |
| dh | Add | fear | fear+hyper | 0.49 | 0.30 | 0.68 | **0.0000** | 8391 | 14 | 146 | 5 | 0.17 | 3.31 |
| dl | Add | hyper | hyper+fear | 0.65 | 0.55 | 0.74 | **0.0000** | 8391 | 14 | 547 | 29 | 0.17 | 5.03 |
| dh | Add | fear | fear+argues | 0.36 | 0.18 | 0.55 | **0.0001** | 8212 | 32 | 147 | 5 | 0.39 | 3.29 |
| dl | Add | argues | argues+fear | 0.49 | 0.40 | 0.59 | **0.0000** | 8212 | 32 | 706 | 31 | 0.39 | 4.21 |
| dh | Add | fear | fear+temper | 0.35 | 0.16 | 0.53 | **0.0002** | 8643 | 32 | 168 | 5 | 0.37 | 2.89 |
| dl | Add | temper | temper+fear | 0.44 | 0.30 | 0.58 | **0.0000** | 8643 | 32 | 294 | 12 | 0.37 | 3.92 |
| dh | Add | fear | fear+fights | . | . | . | . | 8943 | 3 | 227 | 0 | 0.03 | 0.00 |
| dl | Add | fights | fights+fear | 0.70 | 0.45 | 0.95 | **0.0000** | 8943 | 3 | 33 | 2 | 0.03 | 5.71 |
| dh | Add | fear | fear+steals | 0.69 | 0.44 | 0.93 | **0.0000** | 8939 | 1 | 228 | 3 | 0.01 | 1.30 |
| dl | Add | steals | steals+fear | 0.79 | 0.56 | 1.00 | **0.0000** | 8939 | 1 | 39 | 2 | 0.01 | 4.88 |
| dh | Add | worry | worry+dysph | 0.60 | 0.48 | 0.71 | **0.0000** | 8761 | 17 | 349 | 18 | 0.19 | 4.90 |
| dl | Add | dysph | dysph+worry | 0.67 | 0.50 | 0.84 | **0.0000** | 8761 | 17 | 30 | 5 | 0.19 | 14.29 |
| dh | Add | worry | worry+worth | 0.51 | 0.38 | 0.64 | **0.0000** | 8750 | 20 | 342 | 13 | 0.23 | 3.66 |
| dl | Add | worth | worth+worry | 0.32 | -.01 | 0.65 | 0.0597 | 8750 | 20 | 42 | 1 | 0.23 | 2.33 |
| dh | Add | worry | worry+attend | 0.52 | 0.40 | 0.63 | **0.0000** | 7810 | 42 | 224 | 18 | 0.53 | 7.44 |
| dl | Add | attend | attend+worry | 0.51 | 0.42 | 0.59 | **0.0000** | 7810 | 42 | 910 | 51 | 0.53 | 5.31 |
| dh | Add | worry | worry+hyper | 0.40 | 0.24 | 0.56 | **0.0000** | 8230 | 24 | 294 | 8 | 0.29 | 2.65 |
| dl | Add | hyper | hyper+worry | 0.51 | 0.40 | 0.63 | **0.0000** | 8230 | 24 | 537 | 22 | 0.29 | 3.94 |
| dh | Add | worry | worry+argues | 0.47 | 0.35 | 0.60 | **0.0000** | 8079 | 37 | 265 | 15 | 0.46 | 5.36 |
| dl | Add | argues | argues+worry | 0.52 | 0.43 | 0.61 | **0.0000** | 8079 | 37 | 660 | 37 | 0.46 | 5.31 |
| dh | Add | worry | worry+temper | 0.42 | 0.28 | 0.56 | **0.0000** | 8495 | 30 | 312 | 11 | 0.35 | 3.41 |
| dl | Add | temper | temper+worry | 0.51 | 0.38 | 0.63 | **0.0000** | 8495 | 30 | 273 | 15 | 0.35 | 5.21 |
| dh | Add | worry | worry+fights | 0.57 | 0.25 | 0.88 | **0.0004** | 8780 | 1 | 390 | 2 | 0.01 | 0.51 |
| dl | Add | fights | fights+worry | . | . | . | . | 8780 | 1 | 32 | 0 | 0.01 | 0.00 |
| dh | Add | worry | worry+steals | 0.58 | 0.38 | 0.79 | **0.0000** | 8770 | 3 | 393 | 5 | 0.03 | 1.26 |
| dl | Add | steals | steals+worry | 0.57 | 0.22 | 0.93 | **0.0015** | 8770 | 3 | 39 | 1 | 0.03 | 2.50 |
| dh | Add | dysph | dysph+worth | 0.59 | 0.39 | 0.78 | **0.0000** | 9058 | 16 | 47 | 4 | 0.18 | 7.84 |
| dl | Add | worth | worth+dysph | 0.62 | 0.46 | 0.79 | **0.0000** | 9058 | 16 | 65 | 6 | 0.18 | 8.45 |
| dh | Add | dysph | dysph+attend | 0.68 | 0.49 | 0.87 | **0.0000** | 8050 | 11 | 29 | 4 | 0.14 | 12.12 |
| dl | Add | attend | attend+dysph | 0.55 | 0.43 | 0.66 | **0.0000** | 8050 | 11 | 1059 | 25 | 0.14 | 2.31 |
| dh | Add | dysph | dysph+hyper | 0.51 | 0.25 | 0.78 | **0.0001** | 8496 | 10 | 48 | 2 | 0.12 | 4.00 |
| dl | Add | hyper | hyper+dysph | 0.51 | 0.36 | 0.65 | **0.0000** | 8496 | 10 | 627 | 12 | 0.12 | 1.88 |
| dh | Add | dysph | dysph+argues | 0.38 | 0.04 | 0.72 | **0.0298** | 8348 | 18 | 29 | 1 | 0.22 | 3.33 |
| dl | Add | argues | argues+dysph | 0.47 | 0.35 | 0.60 | **0.0000** | 8348 | 18 | 760 | 19 | 0.22 | 2.44 |
| dh | Add | dysph | dysph+temper | 0.55 | 0.33 | 0.77 | **0.0000** | 8792 | 19 | 34 | 3 | 0.22 | 8.11 |
| dl | Add | temper | temper+dysph | 0.37 | 0.19 | 0.55 | **0.0001** | 8792 | 19 | 328 | 6 | 0.22 | 1.80 |
| dh | Add | dysph | dysph+fights | 0.57 | 0.31 | 0.84 | **0.0000** | 9101 | 5 | 65 | 2 | 0.05 | 2.99 |
| dl | Add | fights | fights+dysph | . | . | . | . | 9101 | 5 | 39 | 0 | 0.05 | 0.00 |
| dh | Add | dysph | dysph+steals | . | . | . | . | 9100 | 4 | 67 | 0 | 0.04 | 0.00 |
| dl | Add | steals | steals+dysph | 0.54 | 0.18 | 0.89 | **0.0028** | 9100 | 4 | 40 | 1 | 0.04 | 2.44 |
| dh | Add | worth | worth+attend | 0.38 | 0.04 | 0.73 | **0.0293** | 8040 | 12 | 41 | 1 | 0.15 | 2.38 |
| dl | Add | attend | attend+worth | 0.48 | 0.35 | 0.61 | **0.0000** | 8040 | 12 | 1054 | 19 | 0.15 | 1.77 |
| dh | Add | worth | worth+hyper | 0.37 | 0.02 | 0.71 | **0.0367** | 8481 | 9 | 65 | 1 | 0.11 | 1.52 |
| dl | Add | hyper | hyper+worth | 0.51 | 0.35 | 0.66 | **0.0000** | 8481 | 9 | 624 | 11 | 0.11 | 1.73 |
| dh | Add | worth | worth+argues | 0.58 | 0.36 | 0.81 | **0.0000** | 8338 | 11 | 44 | 3 | 0.13 | 6.38 |
| dl | Add | argues | argues+worth | 0.54 | 0.42 | 0.66 | **0.0000** | 8338 | 11 | 758 | 18 | 0.13 | 2.32 |
| dh | Add | worth | worth+temper | 0.56 | 0.34 | 0.79 | **0.0000** | 8780 | 11 | 54 | 3 | 0.13 | 5.26 |
| dl | Add | temper | temper+worth | 0.58 | 0.43 | 0.72 | **0.0000** | 8780 | 11 | 323 | 11 | 0.13 | 3.29 |
| dh | Add | worth | worth+fights | 0.65 | 0.39 | 0.92 | **0.0000** | 9084 | 2 | 85 | 2 | 0.02 | 2.30 |
| dl | Add | fights | fights+worth | . | . | . | . | 9084 | 2 | 39 | 0 | 0.02 | 0.00 |
| dh | Add | worth | worth+steals | 0.65 | 0.38 | 0.92 | **0.0000** | 9081 | 2 | 86 | 2 | 0.02 | 2.27 |
| dl | Add | steals | steals+worth | . | . | . | . | 9081 | 2 | 42 | 0 | 0.02 | 0.00 |
| dh | Add | attend | attend+hyper | 0.54 | 0.47 | 0.61 | **0.0000** | 7837 | 96 | 551 | 72 | 1.21 | 11.56 |
| dl | Add | hyper | hyper+attend | 0.46 | 0.35 | 0.58 | **0.0000** | 7837 | 96 | 142 | 19 | 1.21 | 11.80 |
| dh | Add | attend | attend+argues | 0.56 | 0.48 | 0.63 | **0.0000** | 7564 | 54 | 714 | 64 | 0.71 | 8.23 |
| dl | Add | argues | argues+attend | 0.60 | 0.52 | 0.67 | **0.0000** | 7564 | 54 | 425 | 51 | 0.71 | 10.71 |
| dh | Add | attend | attend+temper | 0.52 | 0.44 | 0.61 | **0.0000** | 7869 | 37 | 893 | 49 | 0.47 | 5.20 |
| dl | Add | temper | temper+attend | 0.54 | 0.42 | 0.66 | **0.0000** | 7869 | 37 | 173 | 15 | 0.47 | 7.98 |
| dh | Add | attend | attend+fights | 0.30 | 0.12 | 0.49 | **0.0013** | 8069 | 10 | 1087 | 7 | 0.12 | 0.64 |
| dl | Add | fights | fights+attend | 0.54 | 0.19 | 0.88 | **0.0025** | 8069 | 10 | 14 | 1 | 0.12 | 6.67 |
| dh | Add | attend | attend+steals | 0.55 | 0.39 | 0.70 | **0.0000** | 8073 | 4 | 1082 | 12 | 0.05 | 1.10 |
| dl | Add | steals | steals+attend | 0.74 | 0.51 | 0.98 | **0.0000** | 8073 | 4 | 15 | 2 | 0.05 | 11.76 |
| dh | Add | hyper | hyper+argues | 0.55 | 0.46 | 0.64 | **0.0000** | 7924 | 50 | 387 | 35 | 0.63 | 8.29 |
| dl | Add | argues | argues+hyper | 0.51 | 0.42 | 0.59 | **0.0000** | 7924 | 50 | 544 | 38 | 0.63 | 6.53 |
| dh | Add | hyper | hyper+temper | 0.50 | 0.40 | 0.60 | **0.0000** | 8282 | 35 | 505 | 26 | 0.42 | 4.90 |
| dl | Add | temper | temper+hyper | 0.51 | 0.39 | 0.64 | **0.0000** | 8282 | 35 | 224 | 15 | 0.42 | 6.28 |
| dh | Add | hyper | hyper+fights | 0.30 | 0.09 | 0.52 | **0.0063** | 8523 | 9 | 637 | 4 | 0.11 | 0.62 |
| dl | Add | fights | fights+hyper | 0.49 | 0.15 | 0.84 | **0.0052** | 8523 | 9 | 23 | 1 | 0.11 | 4.17 |
| dh | Add | hyper | hyper+steals | 0.39 | 0.18 | 0.60 | **0.0002** | 8523 | 7 | 636 | 5 | 0.08 | 0.78 |
| dl | Add | steals | steals+hyper | 0.64 | 0.38 | 0.89 | **0.0000** | 8523 | 7 | 24 | 2 | 0.08 | 7.69 |
| dh | Add | argues | argues+temper | 0.52 | 0.44 | 0.59 | **0.0000** | 8182 | 75 | 538 | 53 | 0.91 | 8.97 |
| dl | Add | temper | temper+argues | 0.56 | 0.46 | 0.67 | **0.0000** | 8182 | 75 | 118 | 21 | 0.91 | 15.11 |
| dh | Add | argues | argues+fights | 0.45 | 0.29 | 0.61 | **0.0000** | 8374 | 9 | 780 | 10 | 0.11 | 1.27 |
| dl | Add | fights | fights+argues | 0.57 | 0.22 | 0.91 | **0.0012** | 8374 | 9 | 12 | 1 | 0.11 | 7.69 |
| dh | Add | argues | argues+steals | 0.49 | 0.35 | 0.62 | **0.0000** | 8362 | 11 | 784 | 14 | 0.13 | 1.75 |
| dl | Add | steals | steals+argues | 0.73 | 0.55 | 0.91 | **0.0000** | 8362 | 11 | 19 | 4 | 0.13 | 17.39 |
| dh | Add | temper | temper+fights | 0.53 | 0.36 | 0.70 | **0.0000** | 8822 | 8 | 336 | 7 | 0.09 | 2.04 |
| dl | Add | fights | fights+temper | 0.74 | 0.54 | 0.94 | **0.0000** | 8822 | 8 | 15 | 3 | 0.09 | 16.67 |
| dh | Add | temper | temper+steals | 0.51 | 0.33 | 0.68 | **0.0000** | 8807 | 9 | 348 | 7 | 0.10 | 1.97 |
| dl | Add | steals | steals+temper | 0.71 | 0.53 | 0.90 | **0.0000** | 8807 | 9 | 28 | 4 | 0.10 | 12.50 |
| dh | Add | fights | fights+steals | . | . | . | . | 9128 | 3 | 40 | 0 | 0.03 | 0.00 |
| dl | Add | steals | steals+fights | . | . | . | . | 9128 | 3 | 42 | 0 | 0.03 | 0.00 |
| dp | Joint Persist | fear+worry | fear+worry | 0.76 | 0.70 | 0.82 | **0.0000** | 8644 | 75 | 95 | 46 | 0.86 | 32.62 |
| dp | Joint Persist | fear+dysph | fear+dysph | 0.65 | 0.46 | 0.84 | **0.0000** | 8916 | 20 | 22 | 4 | 0.22 | 15.38 |
| dp | Joint Persist | fear+worth | fear+worth | 0.76 | 0.62 | 0.89 | **0.0000** | 8909 | 15 | 27 | 7 | 0.17 | 20.59 |
| dp | Joint Persist | fear+attend | fear+attend | 0.75 | 0.67 | 0.84 | **0.0000** | 7940 | 32 | 88 | 25 | 0.40 | 22.12 |
| dp | Joint Persist | fear+hyper | fear+hyper | 0.82 | 0.73 | 0.90 | **0.0000** | 8391 | 14 | 66 | 18 | 0.17 | 21.43 |
| dp | Joint Persist | fear+argues | fear+argues | 0.73 | 0.64 | 0.83 | **0.0000** | 8212 | 32 | 65 | 18 | 0.39 | 21.69 |
| dp | Joint Persist | fear+temper | fear+temper | 0.61 | 0.46 | 0.75 | **0.0000** | 8643 | 32 | 54 | 8 | 0.37 | 12.90 |
| dp | Joint Persist | fear+fights | fear+fights | . | . | . | . | 8943 | 3 | 8 | 0 | 0.03 | 0.00 |
| dp | Joint Persist | fear+steals | fear+steals | 0.89 | 0.66 | 1.00 | **0.0000** | 8939 | 1 | 3 | 1 | 0.01 | 25.00 |
| dp | Joint Persist | worry+dysph | worry+dysph | 0.73 | 0.59 | 0.88 | **0.0000** | 8761 | 17 | 29 | 7 | 0.19 | 19.44 |
| dp | Joint Persist | worry+worth | worry+worth | 0.80 | 0.70 | 0.90 | **0.0000** | 8750 | 20 | 35 | 13 | 0.23 | 27.08 |
| dp | Joint Persist | worry+attend | worry+attend | 0.83 | 0.77 | 0.88 | **0.0000** | 7810 | 42 | 108 | 53 | 0.53 | 32.92 |
| dp | Joint Persist | worry+hyper | worry+hyper | 0.85 | 0.79 | 0.91 | **0.0000** | 8230 | 24 | 68 | 33 | 0.29 | 32.67 |
| dp | Joint Persist | worry+argues | worry+argues | 0.82 | 0.76 | 0.88 | **0.0000** | 8079 | 37 | 84 | 39 | 0.46 | 31.71 |
| dp | Joint Persist | worry+temper | worry+temper | 0.77 | 0.68 | 0.86 | **0.0000** | 8495 | 30 | 60 | 20 | 0.35 | 25.00 |
| dp | Joint Persist | worry+fights | worry+fights | . | . | . | . | 8780 | 1 | 11 | 0 | 0.01 | 0.00 |
| dp | Joint Persist | worry+steals | worry+steals | 0.79 | 0.49 | 1.00 | **0.0000** | 8770 | 3 | 4 | 1 | 0.03 | 20.00 |
| dp | Joint Persist | dysph+worth | dysph+worth | 0.76 | 0.60 | 0.91 | **0.0000** | 9058 | 16 | 15 | 5 | 0.18 | 25.00 |
| dp | Joint Persist | dysph+attend | dysph+attend | 0.74 | 0.59 | 0.89 | **0.0000** | 8050 | 11 | 32 | 6 | 0.14 | 15.79 |
| dp | Joint Persist | dysph+hyper | dysph+hyper | 0.82 | 0.69 | 0.95 | **0.0000** | 8496 | 10 | 15 | 6 | 0.12 | 28.57 |
| dp | Joint Persist | dysph+argues | dysph+argues | 0.84 | 0.75 | 0.93 | **0.0000** | 8348 | 18 | 27 | 14 | 0.22 | 34.15 |
| dp | Joint Persist | dysph+temper | dysph+temper | 0.76 | 0.63 | 0.89 | **0.0000** | 8792 | 19 | 26 | 8 | 0.22 | 23.53 |
| dp | Joint Persist | dysph+fights | dysph+fights | . | . | . | . | 9101 | 5 | 4 | 0 | 0.05 | 0.00 |
| dp | Joint Persist | dysph+steals | dysph+steals | 0.79 | 0.50 | 1.00 | **0.0000** | 9100 | 4 | 3 | 1 | 0.04 | 25.00 |
| dp | Joint Persist | worth+attend | worth+attend | 0.66 | 0.49 | 0.84 | **0.0000** | 8040 | 12 | 44 | 5 | 0.15 | 10.20 |
| dp | Joint Persist | worth+hyper | worth+hyper | 0.78 | 0.63 | 0.93 | **0.0000** | 8481 | 9 | 20 | 5 | 0.11 | 20.00 |
| dp | Joint Persist | worth+argues | worth+argues | 0.78 | 0.65 | 0.90 | **0.0000** | 8338 | 11 | 36 | 8 | 0.13 | 18.18 |
| dp | Joint Persist | worth+temper | worth+temper | 0.68 | 0.50 | 0.87 | **0.0000** | 8780 | 11 | 30 | 4 | 0.13 | 11.76 |
| dp | Joint Persist | worth+fights | worth+fights | . | . | . | . | 9084 | 2 | 4 | 0 | 0.02 | 0.00 |
| dp | Joint Persist | worth+steals | worth+steals | 0.87 | 0.63 | 1.00 | **0.0000** | 9081 | 2 | 2 | 1 | 0.02 | 33.33 |
| dp | Joint Persist | attend+hyper | attend+hyper | 0.87 | 0.84 | 0.89 | **0.0000** | 7837 | 96 | 270 | 229 | 1.21 | 45.89 |
| dp | Joint Persist | attend+argues | attend+argues | 0.87 | 0.84 | 0.90 | **0.0000** | 7564 | 54 | 206 | 138 | 0.71 | 40.12 |
| dp | Joint Persist | attend+temper | attend+temper | 0.83 | 0.78 | 0.88 | **0.0000** | 7869 | 37 | 125 | 55 | 0.47 | 30.56 |
| dp | Joint Persist | attend+fights | attend+fights | 0.66 | 0.45 | 0.87 | **0.0000** | 8069 | 10 | 25 | 3 | 0.12 | 10.71 |
| dp | Joint Persist | attend+steals | attend+steals | 0.86 | 0.74 | 0.98 | **0.0000** | 8073 | 4 | 22 | 6 | 0.05 | 21.43 |
| dp | Joint Persist | hyper+argues | hyper+argues | 0.85 | 0.81 | 0.89 | **0.0000** | 7924 | 50 | 149 | 89 | 0.63 | 37.39 |
| dp | Joint Persist | hyper+temper | hyper+temper | 0.82 | 0.76 | 0.88 | **0.0000** | 8282 | 35 | 90 | 39 | 0.42 | 30.23 |
| dp | Joint Persist | hyper+fights | hyper+fights | 0.52 | 0.18 | 0.87 | **0.0031** | 8523 | 9 | 18 | 1 | 0.11 | 5.26 |
| dp | Joint Persist | hyper+steals | hyper+steals | 0.75 | 0.55 | 0.95 | **0.0000** | 8523 | 7 | 16 | 3 | 0.08 | 15.79 |
| dp | Joint Persist | argues+temper | argues+temper | 0.80 | 0.76 | 0.85 | **0.0000** | 8182 | 75 | 148 | 81 | 0.91 | 35.37 |
| dp | Joint Persist | argues+fights | argues+fights | 0.76 | 0.60 | 0.92 | **0.0000** | 8374 | 9 | 25 | 5 | 0.11 | 16.67 |
| dp | Joint Persist | argues+steals | argues+steals | 0.74 | 0.56 | 0.91 | **0.0000** | 8362 | 11 | 18 | 4 | 0.13 | 18.18 |
| dp | Joint Persist | temper+fights | temper+fights | 0.63 | 0.37 | 0.89 | **0.0000** | 8822 | 8 | 23 | 2 | 0.09 | 8.00 |
| dp | Joint Persist | temper+steals | temper+steals | 0.77 | 0.58 | 0.96 | **0.0000** | 8807 | 9 | 10 | 3 | 0.10 | 23.08 |
| dp | Joint Persist | fights+steals | fights+steals | 0.84 | 0.58 | 1.00 | **0.0000** | 9128 | 3 | 2 | 1 | 0.03 | 33.33 |
| cg | Shift | fear | worry | 0.23 | 0.08 | 0.39 | **0.0031** | 8537 | 182 | 87 | 7 | 2.09 | 7.45 |
| bj | Shift | worry | fear | 0.16 | 0.00 | 0.32 | 0.0446 | 8640 | 79 | 256 | 6 | 0.91 | 2.29 |
| cg | Shift | fear | dysph | 0.20 | -.01 | 0.41 | 0.0640 | 8899 | 37 | 206 | 3 | 0.41 | 1.44 |
| bj | Shift | dysph | fear | 0.26 | 0.07 | 0.46 | **0.0074** | 8767 | 169 | 41 | 4 | 1.89 | 8.89 |
| cg | Shift | fear | worth | 0.19 | -.02 | 0.39 | 0.0823 | 8882 | 42 | 198 | 3 | 0.47 | 1.49 |
| bj | Shift | worth | fear | 0.27 | 0.09 | 0.44 | **0.0034** | 8752 | 172 | 52 | 5 | 1.93 | 8.77 |
| cg | Shift | fear | attend | 0.08 | -.06 | 0.22 | 0.2861 | 7618 | 354 | 114 | 8 | 4.44 | 6.56 |
| bj | Shift | attend | fear | 0.06 | -.05 | 0.18 | 0.2679 | 7879 | 93 | 993 | 16 | 1.17 | 1.59 |
| cg | Shift | fear | hyper | 0.12 | -.03 | 0.27 | 0.1121 | 8201 | 204 | 144 | 7 | 2.43 | 4.64 |
| bj | Shift | hyper | fear | 0.08 | -.04 | 0.19 | 0.1760 | 8267 | 138 | 562 | 14 | 1.64 | 2.43 |
| cg | Shift | fear | argues | -.04 | -.19 | 0.11 | 0.6230 | 7909 | 335 | 147 | 5 | 4.06 | 3.29 |
| bj | Shift | argues | fear | -.11 | -.25 | 0.02 | 0.0996 | 8118 | 126 | 731 | 6 | 1.53 | 0.81 |
| cg | Shift | fear | temper | 0.20 | 0.06 | 0.34 | **0.0046** | 8520 | 155 | 164 | 9 | 1.79 | 5.20 |
| bj | Shift | temper | fear | 0.11 | -.02 | 0.24 | 0.1063 | 8533 | 142 | 297 | 9 | 1.64 | 2.94 |
| cg | Shift | fear | fights | 0.08 | -.22 | 0.38 | 0.6170 | 8923 | 23 | 226 | 1 | 0.26 | 0.44 |
| bj | Shift | fights | fear | . | . | . | . | 8756 | 190 | 35 | 0 | 2.12 | 0.00 |
| cg | Shift | fear | steals | 0.23 | 0.01 | 0.44 | **0.0380** | 8912 | 28 | 228 | 3 | 0.31 | 1.30 |
| bj | Shift | steals | fear | . | . | . | . | 8748 | 192 | 41 | 0 | 2.15 | 0.00 |
| cg | Shift | worry | dysph | -.04 | -.32 | 0.24 | 0.7924 | 8747 | 31 | 366 | 1 | 0.35 | 0.27 |
| bj | Shift | dysph | worry | 0.19 | -.02 | 0.40 | 0.0807 | 8532 | 246 | 32 | 3 | 2.80 | 8.57 |
| cg | Shift | worry | worth | 0.08 | -.16 | 0.31 | 0.5153 | 8740 | 30 | 353 | 2 | 0.34 | 0.56 |
| bj | Shift | worth | worry | 0.26 | 0.08 | 0.44 | **0.0051** | 8525 | 245 | 38 | 5 | 2.79 | 11.63 |
| cg | Shift | worry | attend | 0.02 | -.10 | 0.14 | 0.7958 | 7522 | 330 | 231 | 11 | 4.20 | 4.55 |
| bj | Shift | attend | worry | -.02 | -.12 | 0.08 | 0.7296 | 7692 | 160 | 943 | 18 | 2.04 | 1.87 |
| cg | Shift | worry | hyper | 0.05 | -.08 | 0.18 | 0.4484 | 8065 | 189 | 293 | 9 | 2.29 | 2.98 |
| bj | Shift | hyper | worry | 0.03 | -.08 | 0.13 | 0.6370 | 8045 | 209 | 543 | 16 | 2.53 | 2.86 |
| cg | Shift | worry | argues | -.06 | -.19 | 0.07 | 0.3536 | 7799 | 317 | 272 | 8 | 3.91 | 2.86 |
| bj | Shift | argues | worry | 0.01 | -.10 | 0.11 | 0.9111 | 7935 | 181 | 681 | 16 | 2.23 | 2.30 |
| cg | Shift | worry | temper | -.17 | -.36 | 0.02 | 0.0731 | 8367 | 158 | 321 | 2 | 1.85 | 0.62 |
| bj | Shift | temper | worry | 0.06 | -.06 | 0.19 | 0.3412 | 8309 | 216 | 278 | 10 | 2.53 | 3.47 |
| cg | Shift | worry | fights | -.00 | -.29 | 0.29 | 0.9793 | 8758 | 23 | 391 | 1 | 0.26 | 0.26 |
| bj | Shift | fights | worry | 0.19 | -.02 | 0.41 | 0.0782 | 8514 | 267 | 29 | 3 | 3.04 | 9.38 |
| cg | Shift | worry | steals | 0.09 | -.15 | 0.33 | 0.4648 | 8748 | 25 | 396 | 2 | 0.28 | 0.50 |
| bj | Shift | steals | worry | 0.15 | -.05 | 0.36 | 0.1460 | 8509 | 264 | 37 | 3 | 3.01 | 7.50 |
| cg | Shift | dysph | worth | 0.32 | 0.06 | 0.57 | **0.0141** | 9031 | 43 | 49 | 2 | 0.47 | 3.92 |
| bj | Shift | worth | dysph | 0.15 | -.16 | 0.45 | 0.3399 | 9030 | 44 | 70 | 1 | 0.48 | 1.41 |
| cg | Shift | dysph | attend | 0.17 | -.02 | 0.37 | 0.0817 | 7679 | 382 | 29 | 4 | 4.74 | 12.12 |
| bj | Shift | attend | dysph | 0.11 | -.06 | 0.28 | 0.2227 | 8036 | 25 | 1078 | 6 | 0.31 | 0.55 |
| cg | Shift | dysph | hyper | 0.14 | -.06 | 0.35 | 0.1686 | 8291 | 215 | 47 | 3 | 2.53 | 6.00 |
| bj | Shift | hyper | dysph | -.01 | -.20 | 0.18 | 0.9322 | 8464 | 42 | 636 | 3 | 0.49 | 0.47 |
| cg | Shift | dysph | argues | 0.07 | -.16 | 0.31 | 0.5377 | 8010 | 356 | 28 | 2 | 4.26 | 6.67 |
| bj | Shift | argues | dysph | 0.09 | -.10 | 0.28 | 0.3712 | 8340 | 26 | 775 | 4 | 0.31 | 0.51 |
| cg | Shift | dysph | temper | 0.04 | -.25 | 0.33 | 0.7762 | 8633 | 178 | 36 | 1 | 2.02 | 2.70 |
| bj | Shift | temper | dysph | 0.22 | 0.04 | 0.40 | **0.0164** | 8774 | 37 | 329 | 5 | 0.42 | 1.50 |
| cg | Shift | dysph | fights | . | . | . | . | 9086 | 20 | 67 | 0 | 0.22 | 0.00 |
| bj | Shift | fights | dysph | 0.31 | 0.06 | 0.56 | **0.0162** | 9046 | 60 | 37 | 2 | 0.66 | 5.13 |
| cg | Shift | dysph | steals | . | . | . | . | 9073 | 31 | 67 | 0 | 0.34 | 0.00 |
| bj | Shift | steals | dysph | . | . | . | . | 9042 | 62 | 41 | 0 | 0.68 | 0.00 |
| cg | Shift | worth | attend | 0.13 | -.06 | 0.32 | 0.1901 | 7668 | 384 | 38 | 4 | 4.77 | 9.52 |
| bj | Shift | attend | worth | 0.19 | 0.04 | 0.35 | **0.0157** | 8027 | 25 | 1064 | 9 | 0.31 | 0.84 |
| cg | Shift | worth | hyper | 0.15 | -.03 | 0.34 | 0.1115 | 8274 | 216 | 62 | 4 | 2.54 | 6.06 |
| bj | Shift | hyper | worth | . | . | . | . | 8445 | 45 | 635 | 0 | 0.53 | 0.00 |
| cg | Shift | worth | argues | 0.07 | -.13 | 0.27 | 0.5079 | 7989 | 360 | 44 | 3 | 4.31 | 6.38 |
| bj | Shift | argues | worth | 0.18 | 0.01 | 0.34 | **0.0345** | 8320 | 29 | 769 | 7 | 0.35 | 0.90 |
| cg | Shift | worth | temper | 0.21 | 0.02 | 0.40 | **0.0311** | 8608 | 183 | 53 | 4 | 2.08 | 7.02 |
| bj | Shift | temper | worth | 0.17 | -.02 | 0.36 | 0.0838 | 8752 | 39 | 330 | 4 | 0.44 | 1.20 |
| cg | Shift | worth | fights | . | . | . | . | 9063 | 23 | 87 | 0 | 0.25 | 0.00 |
| bj | Shift | fights | worth | 0.31 | 0.05 | 0.56 | **0.0171** | 9025 | 61 | 37 | 2 | 0.67 | 5.13 |
| cg | Shift | worth | steals | 0.17 | -.14 | 0.48 | 0.2778 | 9053 | 30 | 87 | 1 | 0.33 | 1.14 |
| bj | Shift | steals | worth | . | . | . | . | 9020 | 63 | 42 | 0 | 0.69 | 0.00 |
| cg | Shift | attend | hyper | 0.24 | 0.11 | 0.37 | **0.0003** | 7884 | 49 | 610 | 13 | 0.62 | 2.09 |
| bj | Shift | hyper | attend | 0.26 | 0.15 | 0.37 | **0.0000** | 7665 | 268 | 143 | 18 | 3.38 | 11.18 |
| cg | Shift | attend | argues | 0.02 | -.07 | 0.11 | 0.6665 | 7385 | 233 | 752 | 26 | 3.06 | 3.34 |
| bj | Shift | argues | attend | 0.04 | -.05 | 0.14 | 0.3778 | 7343 | 275 | 455 | 21 | 3.61 | 4.41 |
| cg | Shift | attend | temper | -.02 | -.14 | 0.10 | 0.7015 | 7802 | 104 | 931 | 11 | 1.32 | 1.17 |
| bj | Shift | temper | attend | 0.14 | 0.02 | 0.25 | **0.0215** | 7572 | 334 | 173 | 15 | 4.22 | 7.98 |
| cg | Shift | attend | fights | -.03 | -.34 | 0.28 | 0.8481 | 8070 | 9 | 1093 | 1 | 0.11 | 0.09 |
| bj | Shift | fights | attend | 0.18 | -.08 | 0.44 | 0.1819 | 7691 | 388 | 13 | 2 | 4.80 | 13.33 |
| cg | Shift | attend | steals | 0.12 | -.09 | 0.33 | 0.2516 | 8062 | 15 | 1090 | 4 | 0.19 | 0.37 |
| bj | Shift | steals | attend | 0.03 | -.28 | 0.34 | 0.8510 | 7683 | 394 | 16 | 1 | 4.88 | 5.88 |
| cg | Shift | hyper | argues | 0.06 | -.04 | 0.16 | 0.2548 | 7701 | 273 | 403 | 19 | 3.42 | 4.50 |
| bj | Shift | argues | hyper | -.04 | -.17 | 0.09 | 0.5684 | 7840 | 134 | 574 | 8 | 1.68 | 1.37 |
| cg | Shift | hyper | temper | 0.11 | -.01 | 0.23 | 0.0609 | 8191 | 126 | 517 | 14 | 1.51 | 2.64 |
| bj | Shift | temper | hyper | 0.14 | 0.01 | 0.27 | **0.0391** | 8147 | 170 | 229 | 10 | 2.04 | 4.18 |
| cg | Shift | hyper | fights | 0.22 | -.02 | 0.45 | 0.0694 | 8521 | 11 | 638 | 3 | 0.13 | 0.47 |
| bj | Shift | fights | hyper | 0.27 | 0.05 | 0.50 | **0.0164** | 8315 | 217 | 21 | 3 | 2.54 | 12.50 |
| cg | Shift | hyper | steals | 0.23 | 0.04 | 0.42 | **0.0176** | 8512 | 18 | 636 | 5 | 0.21 | 0.78 |
| bj | Shift | steals | hyper | 0.18 | -.07 | 0.43 | 0.1606 | 8311 | 219 | 24 | 2 | 2.57 | 7.69 |
| cg | Shift | argues | temper | 0.10 | -.04 | 0.25 | 0.1684 | 8192 | 65 | 583 | 8 | 0.79 | 1.35 |
| bj | Shift | temper | argues | 0.16 | 0.02 | 0.29 | **0.0225** | 7986 | 271 | 129 | 10 | 3.28 | 7.19 |
| cg | Shift | argues | fights | 0.21 | -.08 | 0.49 | 0.1517 | 8377 | 6 | 788 | 2 | 0.07 | 0.25 |
| bj | Shift | fights | argues | 0.31 | 0.07 | 0.55 | **0.0105** | 8019 | 364 | 10 | 3 | 4.34 | 23.08 |
| cg | Shift | argues | steals | 0.25 | 0.01 | 0.50 | 0.0429 | 8366 | 7 | 795 | 3 | 0.08 | 0.38 |
| bj | Shift | steals | argues | 0.12 | -.13 | 0.36 | 0.3456 | 8013 | 360 | 21 | 2 | 4.30 | 8.70 |
| cg | Shift | temper | fights | 0.26 | -.01 | 0.52 | 0.0602 | 8820 | 10 | 341 | 2 | 0.11 | 0.58 |
| bj | Shift | fights | temper | 0.35 | 0.12 | 0.58 | **0.0025** | 8643 | 187 | 15 | 3 | 2.12 | 16.67 |
| cg | Shift | temper | steals | 0.32 | 0.11 | 0.52 | **0.0028** | 8801 | 15 | 351 | 4 | 0.17 | 1.13 |
| bj | Shift | steals | temper | 0.36 | 0.18 | 0.55 | **0.0001** | 8633 | 183 | 27 | 5 | 2.08 | 15.63 |
| cg | Shift | fights | steals | 0.27 | -.05 | 0.60 | 0.0968 | 9100 | 31 | 39 | 1 | 0.34 | 2.50 |
| bj | Shift | steals | fights | . | . | . | . | 9107 | 24 | 42 | 0 | 0.26 | 0.00 |
| co | Subtract x | fear+worry | worry | 0.50 | 0.41 | 0.60 | **0.0000** | 8537 | 182 | 112 | 29 | 2.09 | 20.57 |
| co | Subtract x | fear+dysph | dysph | 0.57 | 0.38 | 0.77 | **0.0000** | 8899 | 37 | 22 | 4 | 0.41 | 15.38 |
| co | Subtract x | fear+worth | worth | 0.56 | 0.38 | 0.74 | **0.0000** | 8882 | 42 | 29 | 5 | 0.47 | 14.71 |
| co | Subtract x | fear+attend | attend | 0.56 | 0.48 | 0.64 | **0.0000** | 7618 | 354 | 68 | 45 | 4.44 | 39.82 |
| co | Subtract x | fear+hyper | hyper | 0.55 | 0.45 | 0.65 | **0.0000** | 8201 | 204 | 59 | 25 | 2.43 | 29.76 |
| co | Subtract x | fear+argues | argues | 0.55 | 0.45 | 0.64 | **0.0000** | 7909 | 335 | 51 | 32 | 4.06 | 38.55 |
| co | Subtract x | fear+temper | temper | 0.51 | 0.38 | 0.63 | **0.0000** | 8520 | 155 | 48 | 14 | 1.79 | 22.58 |
| co | Subtract x | fear+fights | fights | . | . | . | . | 8923 | 23 | 8 | 0 | 0.26 | 0.00 |
| co | Subtract x | fear+steals | steals | . | . | . | . | 8912 | 28 | 4 | 0 | 0.31 | 0.00 |
| co | Subtract x | worry+dysph | dysph | 0.55 | 0.35 | 0.74 | **0.0000** | 8747 | 31 | 32 | 4 | 0.35 | 11.11 |
| co | Subtract x | worry+worth | worth | 0.51 | 0.31 | 0.71 | **0.0000** | 8740 | 30 | 44 | 4 | 0.34 | 8.33 |
| co | Subtract x | worry+attend | attend | 0.46 | 0.38 | 0.55 | **0.0000** | 7522 | 330 | 118 | 43 | 4.20 | 26.71 |
| co | Subtract x | worry+hyper | hyper | 0.51 | 0.41 | 0.61 | **0.0000** | 8065 | 189 | 77 | 24 | 2.29 | 23.76 |
| co | Subtract x | worry+argues | argues | 0.42 | 0.33 | 0.52 | **0.0000** | 7799 | 317 | 94 | 29 | 3.91 | 23.58 |
| co | Subtract x | worry+temper | temper | 0.45 | 0.32 | 0.57 | **0.0000** | 8367 | 158 | 66 | 14 | 1.85 | 17.50 |
| co | Subtract x | worry+fights | fights | 0.48 | 0.14 | 0.83 | **0.0061** | 8758 | 23 | 10 | 1 | 0.26 | 9.09 |
| co | Subtract x | worry+steals | steals | 0.58 | 0.24 | 0.93 | **0.0010** | 8748 | 25 | 4 | 1 | 0.28 | 20.00 |
| co | Subtract x | dysph+worth | worth | 0.33 | -.01 | 0.66 | 0.0544 | 9031 | 43 | 19 | 1 | 0.47 | 5.00 |
| co | Subtract x | dysph+attend | attend | 0.55 | 0.43 | 0.67 | 0.0000 | 7679 | 382 | 20 | 18 | 4.74 | 47.37 |
| co | Subtract x | dysph+hyper | hyper | 0.61 | 0.47 | 0.75 | 0.0000 | 8291 | 215 | 11 | 10 | 2.53 | 47.62 |
| co | Subtract x | dysph+argues | argues | 0.44 | 0.31 | 0.57 | **0.0000** | 8010 | 356 | 28 | 13 | 4.26 | 31.71 |
| co | Subtract x | dysph+temper | temper | 0.50 | 0.35 | 0.65 | **0.0000** | 8633 | 178 | 25 | 9 | 2.02 | 26.47 |
| co | Subtract x | dysph+fights | fights | . | . | . | . | 9086 | 20 | 4 | 0 | 0.22 | 0.00 |
| co | Subtract x | dysph+steals | steals | 0.60 | 0.25 | 0.95 | **0.0008** | 9073 | 31 | 3 | 1 | 0.34 | 25.00 |
| co | Subtract x | worth+attend | attend | 0.53 | 0.42 | 0.64 | **0.0000** | 7668 | 384 | 28 | 21 | 4.77 | 42.86 |
| co | Subtract x | worth+hyper | hyper | 0.53 | 0.38 | 0.68 | **0.0000** | 8274 | 216 | 16 | 9 | 2.54 | 36.00 |
| co | Subtract x | worth+argues | argues | 0.54 | 0.42 | 0.65 | **0.0000** | 7989 | 360 | 25 | 19 | 4.31 | 43.18 |
| co | Subtract x | worth+temper | temper | 0.46 | 0.31 | 0.62 | **0.0000** | 8608 | 183 | 26 | 8 | 2.08 | 23.53 |
| co | Subtract x | worth+fights | fights | . | . | . | . | 9063 | 23 | 4 | 0 | 0.25 | 0.00 |
| co | Subtract x | worth+steals | steals | . | . | . | . | 9053 | 30 | 3 | 0 | 0.33 | 0.00 |
| co | Subtract x | attend+hyper | hyper | 0.52 | 0.43 | 0.61 | **0.0000** | 7884 | 49 | 464 | 35 | 0.62 | 7.01 |
| co | Subtract x | attend+argues | argues | 0.42 | 0.35 | 0.50 | **0.0000** | 7385 | 233 | 287 | 57 | 3.06 | 16.57 |
| co | Subtract x | attend+temper | temper | 0.43 | 0.32 | 0.54 | **0.0000** | 7802 | 104 | 161 | 19 | 1.32 | 10.56 |
| co | Subtract x | attend+fights | fights | 0.47 | 0.12 | 0.82 | **0.0081** | 8070 | 9 | 27 | 1 | 0.11 | 3.57 |
| co | Subtract x | attend+steals | steals | 0.41 | 0.06 | 0.75 | **0.0205** | 8062 | 15 | 27 | 1 | 0.19 | 3.57 |
| co | Subtract x | hyper+argues | argues | 0.42 | 0.34 | 0.50 | **0.0000** | 7701 | 273 | 193 | 45 | 3.42 | 18.91 |
| co | Subtract x | hyper+temper | temper | 0.42 | 0.30 | 0.54 | **0.0000** | 8191 | 126 | 113 | 16 | 1.51 | 12.40 |
| co | Subtract x | hyper+fights | fights | 0.50 | 0.15 | 0.84 | **0.0047** | 8521 | 11 | 18 | 1 | 0.13 | 5.26 |
| co | Subtract x | hyper+steals | steals | 0.57 | 0.31 | 0.83 | **0.0000** | 8512 | 18 | 17 | 2 | 0.21 | 10.53 |
| co | Subtract x | argues+temper | temper | 0.44 | 0.33 | 0.56 | **0.0000** | 8192 | 65 | 212 | 17 | 0.79 | 7.42 |
| co | Subtract x | argues+fights | fights | . | . | . | . | 8377 | 6 | 30 | 0 | 0.07 | 0.00 |
| co | Subtract x | argues+steals | steals | 0.53 | 0.19 | 0.88 | **0.0026** | 8366 | 7 | 21 | 1 | 0.08 | 4.55 |
| co | Subtract x | temper+fights | fights | 0.48 | 0.13 | 0.82 | **0.0065** | 8820 | 10 | 24 | 1 | 0.11 | 4.00 |
| co | Subtract x | temper+steals | steals | 0.64 | 0.39 | 0.89 | **0.0000** | 8801 | 15 | 11 | 2 | 0.17 | 15.38 |
| co | Subtract x | fights+steals | steals | 0.64 | 0.30 | 0.99 | **0.0003** | 9100 | 31 | 2 | 1 | 0.34 | 33.33 |
| bn | Subtract y | worry+fear | fear | 0.44 | 0.31 | 0.57 | **0.0000** | 8640 | 79 | 128 | 13 | 0.91 | 9.22 |
| bn | Subtract y | dysph+fear | fear | 0.53 | 0.38 | 0.69 | **0.0000** | 8767 | 169 | 18 | 8 | 1.89 | 30.77 |
| bn | Subtract y | worth+fear | fear | 0.40 | 0.23 | 0.58 | **0.0000** | 8752 | 172 | 28 | 6 | 1.93 | 17.65 |
| bn | Subtract y | attend+fear | fear | 0.38 | 0.24 | 0.52 | **0.0000** | 7879 | 93 | 103 | 10 | 1.17 | 8.85 |
| bn | Subtract y | hyper+fear | fear | 0.35 | 0.21 | 0.50 | **0.0000** | 8267 | 138 | 75 | 9 | 1.64 | 10.71 |
| bn | Subtract y | argues+fear | fear | 0.31 | 0.15 | 0.47 | **0.0001** | 8118 | 126 | 76 | 7 | 1.53 | 8.43 |
| bn | Subtract y | temper+fear | fear | 0.59 | 0.48 | 0.70 | **0.0000** | 8533 | 142 | 44 | 18 | 1.64 | 29.03 |
| bn | Subtract y | fights+fear | fear | 0.51 | 0.27 | 0.75 | **0.0000** | 8756 | 190 | 5 | 3 | 2.12 | 37.50 |
| bn | Subtract y | steals+fear | fear | . | . | . | . | 8748 | 192 | 4 | 0 | 2.15 | 0.00 |
| bn | Subtract y | dysph+worry | worry | 0.51 | 0.38 | 0.65 | **0.0000** | 8532 | 246 | 24 | 12 | 2.80 | 33.33 |
| bn | Subtract y | worth+worry | worry | 0.49 | 0.36 | 0.62 | **0.0000** | 8525 | 245 | 34 | 14 | 2.79 | 29.17 |
| bn | Subtract y | attend+worry | worry | 0.42 | 0.32 | 0.53 | **0.0000** | 7692 | 160 | 138 | 23 | 2.04 | 14.29 |
| bn | Subtract y | hyper+worry | worry | 0.44 | 0.33 | 0.55 | **0.0000** | 8045 | 209 | 81 | 20 | 2.53 | 19.80 |
| bn | Subtract y | argues+worry | worry | 0.41 | 0.30 | 0.52 | **0.0000** | 7935 | 181 | 104 | 19 | 2.23 | 15.45 |
| bn | Subtract y | temper+worry | worry | 0.52 | 0.41 | 0.63 | **0.0000** | 8309 | 216 | 58 | 22 | 2.53 | 27.50 |
| bn | Subtract y | fights+worry | worry | 0.54 | 0.34 | 0.73 | **0.0000** | 8514 | 267 | 6 | 5 | 3.04 | 45.45 |
| bn | Subtract y | steals+worry | worry | . | . | . | . | 8509 | 264 | 5 | 0 | 3.01 | 0.00 |
| bn | Subtract y | worth+dysph | dysph | 0.53 | 0.31 | 0.76 | 0.0000 | 9030 | 44 | 17 | 3 | 0.48 | 15.00 |
| bn | Subtract y | attend+dysph | dysph | 0.50 | 0.28 | 0.73 | 0.0000 | 8036 | 25 | 35 | 3 | 0.31 | 7.89 |
| bn | Subtract y | hyper+dysph | dysph | 0.32 | -.02 | 0.65 | 0.0630 | 8464 | 42 | 20 | 1 | 0.49 | 4.76 |
| bn | Subtract y | argues+dysph | dysph | 0.29 | -.04 | 0.62 | 0.0886 | 8340 | 26 | 40 | 1 | 0.31 | 2.44 |
| bn | Subtract y | temper+dysph | dysph | 0.47 | 0.25 | 0.69 | **0.0000** | 8774 | 37 | 31 | 3 | 0.42 | 8.82 |
| bn | Subtract y | fights+dysph | dysph | 0.69 | 0.43 | 0.95 | **0.0000** | 9046 | 60 | 2 | 2 | 0.66 | 50.00 |
| bn | Subtract y | steals+dysph | dysph | . | . | . | . | 9042 | 62 | 4 | 0 | 0.68 | 0.00 |
| bn | Subtract y | attend+worth | worth | 0.64 | 0.49 | 0.79 | **0.0000** | 8027 | 25 | 42 | 7 | 0.31 | 14.29 |
| bn | Subtract y | hyper+worth | worth | 0.55 | 0.35 | 0.75 | **0.0000** | 8445 | 45 | 21 | 4 | 0.53 | 16.00 |
| bn | Subtract y | argues+worth | worth | 0.46 | 0.24 | 0.69 | **0.0001** | 8320 | 29 | 41 | 3 | 0.35 | 6.82 |
| bn | Subtract y | temper+worth | worth | 0.61 | 0.45 | 0.78 | **0.0000** | 8752 | 39 | 28 | 6 | 0.44 | 17.65 |
| bn | Subtract y | fights+worth | worth | 0.53 | 0.17 | 0.89 | **0.0042** | 9025 | 61 | 3 | 1 | 0.67 | 25.00 |
| bn | Subtract y | steals+worth | worth | . | . | . | . | 9020 | 63 | 3 | 0 | 0.69 | 0.00 |
| bn | Subtract y | hyper+attend | attend | 0.48 | 0.42 | 0.54 | **0.0000** | 7665 | 268 | 400 | 99 | 3.38 | 19.84 |
| bn | Subtract y | argues+attend | attend | 0.44 | 0.37 | 0.52 | **0.0000** | 7343 | 275 | 276 | 68 | 3.61 | 19.77 |
| bn | Subtract y | temper+attend | attend | 0.52 | 0.45 | 0.60 | **0.0000** | 7572 | 334 | 123 | 57 | 4.22 | 31.67 |
| bn | Subtract y | fights+attend | attend | 0.41 | 0.25 | 0.57 | **0.0000** | 7691 | 388 | 19 | 9 | 4.80 | 32.14 |
| bn | Subtract y | steals+attend | attend | 0.49 | 0.35 | 0.64 | **0.0000** | 7683 | 394 | 16 | 12 | 4.88 | 42.86 |
| bn | Subtract y | argues+hyper | hyper | 0.54 | 0.46 | 0.62 | **0.0000** | 7840 | 134 | 195 | 43 | 1.68 | 18.07 |
| bn | Subtract y | temper+hyper | hyper | 0.57 | 0.49 | 0.66 | **0.0000** | 8147 | 170 | 95 | 34 | 2.04 | 26.36 |
| bn | Subtract y | fights+hyper | hyper | 0.60 | 0.45 | 0.75 | **0.0000** | 8315 | 217 | 10 | 9 | 2.54 | 47.37 |
| bn | Subtract y | steals+hyper | hyper | 0.38 | 0.17 | 0.59 | **0.0003** | 8311 | 219 | 15 | 4 | 2.57 | 21.05 |
| bn | Subtract y | temper+argues | argues | 0.54 | 0.47 | 0.61 | **0.0000** | 7986 | 271 | 166 | 63 | 3.28 | 27.51 |
| bn | Subtract y | fights+argues | argues | 0.57 | 0.44 | 0.69 | **0.0000** | 8019 | 364 | 15 | 15 | 4.34 | 50.00 |
| bn | Subtract y | steals+argues | argues | 0.49 | 0.33 | 0.64 | **0.0000** | 8013 | 360 | 13 | 9 | 4.30 | 40.91 |
| bn | Subtract y | fights+temper | temper | 0.56 | 0.41 | 0.71 | **0.0000** | 8643 | 187 | 16 | 9 | 2.12 | 36.00 |
| bn | Subtract y | steals+temper | temper | 0.33 | 0.06 | 0.59 | **0.0164** | 8633 | 183 | 11 | 2 | 2.08 | 15.38 |
| bn | Subtract y | steals+fights | fights | . | . | . | . | 9107 | 24 | 3 | 0 | 0.26 | 0.00 |

| Supplemental Table S3I. Tetrachoric correlations from 4 x 4 pairwise analyses of all 90 combinations of the 10 selected psychological problems with every other problem to quantify the associations of each baseline problem with outcomes in the **second annual follow-up** that define four parsed paths traditionally conflated in estimates of change: ***add path*** (x_1_ at baseline and x_2_ + y_2_ at follow-up); ***joint persist path*** (x_1_ + y_1_ at baseline and x_2_ + y_2_ at follow-up); ***shift path*** (only x_1_ at baseline and only y_2_ at follow-up); ***subtract x path*** (x_1_ + y_1_ at baseline and only y_2_ at follow-up); and ***subtract y path*** (x_1_ + y_1_ at baseline and only x_2_ at follow-up). Results are for problems dichotomized at the **low rating cut (0 vs 1 or 2).** r_t_ = tetrachoric correlation. –CL and +CL = lower and upper 95% confidence intervals for the tetrachoric correlation. c00 = number without the predictor or the outcome; c01 = number without predictor with the outcome; c10 = number with the predictor without the outcome; c11 = number with the predictor and the outcome. Base% = percent of the outcome only x_2_ among children with neither x_1_ nor the other member of the pair, y_1_, at baseline. Obs% = Observed percent of outcomes on the indicated path. Raw P values significant after FDR adjustment in **bold**. | | | | | | | | | | | | | |
| --- | --- | --- | --- | --- | --- | --- | --- | --- | --- | --- | --- | --- | --- |
| prefix | Parsed paths | Predictor | Outcome | r_t_ | -CL | +CL | P | c00 | c01 | c10 | c11 | Base% | Obs% |
| dh | Add | fear | fear+worry | 0.46 | 0.40 | 0.52 | **0.0000** | 5112 | 280 | 414 | 127 | 5.19 | 23.48 |
| dl | Add | worry | worry+fear | 0.39 | 0.35 | 0.44 | **0.0000** | 5112 | 280 | 1341 | 279 | 5.19 | 17.22 |
| dh | Add | fear | fear+dysph | 0.42 | 0.37 | 0.48 | **0.0000** | 6297 | 205 | 1217 | 187 | 3.15 | 13.32 |
| dl | Add | dysph | dysph+fear | 0.43 | 0.36 | 0.50 | **0.0000** | 6297 | 205 | 431 | 79 | 3.15 | 15.49 |
| dh | Add | fear | fear+worth | 0.45 | 0.40 | 0.51 | **0.0000** | 6318 | 171 | 1189 | 176 | 2.64 | 12.89 |
| dl | Add | worth | worth+fear | 0.44 | 0.37 | 0.51 | **0.0000** | 6318 | 171 | 449 | 74 | 2.64 | 14.15 |
| dh | Add | fear | fear+attend | 0.42 | 0.35 | 0.50 | **0.0000** | 3824 | 134 | 535 | 91 | 3.39 | 14.54 |
| dl | Add | attend | attend+fear | 0.38 | 0.33 | 0.44 | **0.0000** | 3824 | 134 | 2692 | 362 | 3.39 | 11.85 |
| dh | Add | fear | fear+hyper | 0.40 | 0.33 | 0.48 | **0.0000** | 4978 | 98 | 907 | 87 | 1.93 | 8.75 |
| dl | Add | hyper | hyper+fear | 0.44 | 0.38 | 0.50 | **0.0000** | 4978 | 98 | 1752 | 184 | 1.93 | 9.50 |
| dh | Add | fear | fear+argues | 0.48 | 0.42 | 0.55 | **0.0000** | 4165 | 124 | 638 | 117 | 2.89 | 15.50 |
| dl | Add | argues | argues+fear | 0.43 | 0.38 | 0.48 | **0.0000** | 4165 | 124 | 2396 | 327 | 2.89 | 12.01 |
| dh | Add | fear | fear+temper | 0.36 | 0.28 | 0.44 | **0.0000** | 5491 | 95 | 1032 | 76 | 1.70 | 6.86 |
| dl | Add | temper | temper+fear | 0.51 | 0.45 | 0.56 | **0.0000** | 5491 | 95 | 1268 | 158 | 1.70 | 11.08 |
| dh | Add | fear | fear+fights | 0.36 | 0.27 | 0.44 | **0.0000** | 6550 | 47 | 1648 | 56 | 0.71 | 3.29 |
| dl | Add | fights | fights+fear | 0.45 | 0.35 | 0.56 | **0.0000** | 6550 | 47 | 390 | 25 | 0.71 | 6.02 |
| dh | Add | fear | fear+steals | 0.37 | 0.27 | 0.46 | **0.0000** | 6725 | 31 | 1764 | 43 | 0.46 | 2.38 |
| dl | Add | steals | steals+fear | 0.55 | 0.44 | 0.67 | **0.0000** | 6725 | 31 | 237 | 19 | 0.46 | 7.42 |
| dh | Add | worry | worry+dysph | 0.38 | 0.33 | 0.43 | **0.0000** | 5398 | 228 | 1973 | 307 | 4.05 | 13.46 |
| dl | Add | dysph | dysph+worry | 0.40 | 0.32 | 0.48 | **0.0000** | 5398 | 228 | 252 | 55 | 4.05 | 17.92 |
| dh | Add | worry | worry+worth | 0.42 | 0.37 | 0.47 | **0.0000** | 5416 | 172 | 1985 | 281 | 3.08 | 12.40 |
| dl | Add | worth | worth+worry | 0.48 | 0.40 | 0.56 | **0.0000** | 5416 | 172 | 280 | 65 | 3.08 | 18.84 |
| dh | Add | worry | worry+attend | 0.35 | 0.29 | 0.41 | **0.0000** | 3219 | 167 | 1025 | 173 | 4.93 | 14.44 |
| dl | Add | attend | attend+worry | 0.42 | 0.37 | 0.47 | **0.0000** | 3219 | 167 | 2107 | 440 | 4.93 | 17.28 |
| dh | Add | worry | worry+hyper | 0.32 | 0.26 | 0.39 | **0.0000** | 4197 | 110 | 1624 | 139 | 2.55 | 7.88 |
| dl | Add | hyper | hyper+worry | 0.49 | 0.44 | 0.55 | **0.0000** | 4197 | 110 | 1404 | 222 | 2.55 | 13.65 |
| dh | Add | worry | worry+argues | 0.37 | 0.31 | 0.43 | **0.0000** | 3592 | 167 | 1102 | 183 | 4.44 | 14.24 |
| dl | Add | argues | argues+worry | 0.43 | 0.38 | 0.48 | **0.0000** | 3592 | 167 | 1814 | 360 | 4.44 | 16.56 |
| dh | Add | worry | worry+temper | 0.32 | 0.26 | 0.39 | **0.0000** | 4687 | 108 | 1763 | 136 | 2.25 | 7.16 |
| dl | Add | temper | temper+worry | 0.55 | 0.49 | 0.60 | **0.0000** | 4687 | 108 | 962 | 176 | 2.25 | 15.47 |
| dh | Add | worry | worry+fights | 0.25 | 0.17 | 0.33 | **0.0000** | 5551 | 53 | 2626 | 71 | 0.95 | 2.63 |
| dl | Add | fights | fights+worry | 0.41 | 0.30 | 0.53 | **0.0000** | 5551 | 53 | 308 | 21 | 0.95 | 6.38 |
| dh | Add | worry | worry+steals | 0.26 | 0.17 | 0.36 | **0.0000** | 5712 | 35 | 2763 | 53 | 0.61 | 1.88 |
| dl | Add | steals | steals+worry | 0.60 | 0.49 | 0.71 | **0.0000** | 5712 | 35 | 165 | 21 | 0.61 | 11.29 |
| dh | Add | dysph | dysph+worth | 0.37 | 0.30 | 0.44 | **0.0000** | 7047 | 252 | 478 | 77 | 3.45 | 13.87 |
| dl | Add | worth | worth+dysph | 0.41 | 0.35 | 0.48 | **0.0000** | 7047 | 252 | 512 | 95 | 3.45 | 15.65 |
| dh | Add | dysph | dysph+attend | 0.36 | 0.25 | 0.46 | **0.0000** | 4192 | 126 | 234 | 32 | 2.92 | 12.03 |
| dl | Add | attend | attend+dysph | 0.43 | 0.38 | 0.48 | **0.0000** | 4192 | 126 | 3147 | 441 | 2.92 | 12.29 |
| dh | Add | dysph | dysph+hyper | 0.38 | 0.28 | 0.47 | **0.0000** | 5486 | 84 | 463 | 37 | 1.51 | 7.40 |
| dl | Add | hyper | hyper+dysph | 0.51 | 0.45 | 0.56 | **0.0000** | 5486 | 84 | 2104 | 232 | 1.51 | 9.93 |
| dh | Add | dysph | dysph+argues | 0.37 | 0.28 | 0.47 | **0.0000** | 4623 | 148 | 236 | 37 | 3.10 | 13.55 |
| dl | Add | argues | argues+dysph | 0.43 | 0.39 | 0.48 | **0.0000** | 4623 | 148 | 2728 | 407 | 3.10 | 12.98 |
| dh | Add | dysph | dysph+temper | 0.37 | 0.27 | 0.46 | **0.0000** | 6111 | 116 | 427 | 40 | 1.86 | 8.57 |
| dl | Add | temper | temper+dysph | 0.54 | 0.49 | 0.59 | **0.0000** | 6111 | 116 | 1460 | 219 | 1.86 | 13.04 |
| dh | Add | dysph | dysph+fights | 0.39 | 0.30 | 0.49 | **0.0000** | 7418 | 58 | 788 | 37 | 0.78 | 4.48 |
| dl | Add | fights | fights+dysph | 0.51 | 0.42 | 0.60 | **0.0000** | 7418 | 58 | 395 | 35 | 0.78 | 8.14 |
| dh | Add | dysph | dysph+steals | 0.31 | 0.20 | 0.41 | **0.0000** | 7578 | 56 | 901 | 28 | 0.73 | 3.01 |
| dl | Add | steals | steals+dysph | 0.59 | 0.50 | 0.68 | **0.0000** | 7578 | 56 | 239 | 33 | 0.73 | 12.13 |
| dh | Add | worth | worth+attend | 0.45 | 0.35 | 0.54 | **0.0000** | 4234 | 101 | 214 | 35 | 2.33 | 14.06 |
| dl | Add | attend | attend+worth | 0.43 | 0.38 | 0.48 | **0.0000** | 4234 | 101 | 3156 | 363 | 2.33 | 10.32 |
| dh | Add | worth | worth+hyper | 0.41 | 0.32 | 0.50 | **0.0000** | 5486 | 86 | 456 | 42 | 1.54 | 8.43 |
| dl | Add | hyper | hyper+worth | 0.45 | 0.39 | 0.50 | **0.0000** | 5486 | 86 | 2096 | 186 | 1.54 | 8.15 |
| dh | Add | worth | worth+argues | 0.53 | 0.45 | 0.61 | **0.0000** | 4593 | 108 | 282 | 61 | 2.30 | 17.78 |
| dl | Add | argues | argues+worth | 0.42 | 0.37 | 0.47 | **0.0000** | 4593 | 108 | 2835 | 318 | 2.30 | 10.09 |
| dh | Add | worth | worth+temper | 0.40 | 0.31 | 0.48 | **0.0000** | 6052 | 108 | 486 | 48 | 1.75 | 8.99 |
| dl | Add | temper | temper+worth | 0.47 | 0.42 | 0.53 | **0.0000** | 6052 | 108 | 1524 | 170 | 1.75 | 10.04 |
| dh | Add | worth | worth+fights | 0.46 | 0.37 | 0.55 | **0.0000** | 7339 | 44 | 875 | 43 | 0.60 | 4.68 |
| dl | Add | fights | fights+worth | 0.50 | 0.40 | 0.60 | **0.0000** | 7339 | 44 | 441 | 30 | 0.60 | 6.37 |
| dh | Add | worth | worth+steals | 0.41 | 0.30 | 0.51 | **0.0000** | 7552 | 35 | 946 | 30 | 0.46 | 3.07 |
| dl | Add | steals | steals+worth | 0.64 | 0.55 | 0.73 | **0.0000** | 7552 | 35 | 237 | 30 | 0.46 | 11.24 |
| dh | Add | attend | attend+hyper | 0.41 | 0.36 | 0.46 | **0.0000** | 4046 | 173 | 1577 | 275 | 4.10 | 14.85 |
| dl | Add | hyper | hyper+attend | 0.44 | 0.36 | 0.52 | **0.0000** | 4046 | 173 | 296 | 70 | 4.10 | 19.13 |
| dh | Add | attend | attend+argues | 0.47 | 0.42 | 0.51 | **0.0000** | 3065 | 172 | 1434 | 373 | 5.31 | 20.64 |
| dl | Add | argues | argues+attend | 0.42 | 0.36 | 0.47 | **0.0000** | 3065 | 172 | 1101 | 247 | 5.31 | 18.32 |
| dh | Add | attend | attend+temper | 0.42 | 0.37 | 0.48 | **0.0000** | 3804 | 99 | 2489 | 302 | 2.54 | 10.82 |
| dl | Add | temper | temper+attend | 0.51 | 0.44 | 0.58 | **0.0000** | 3804 | 99 | 576 | 105 | 2.54 | 15.42 |
| dh | Add | attend | attend+fights | 0.40 | 0.33 | 0.47 | **0.0000** | 4406 | 34 | 3700 | 161 | 0.77 | 4.17 |
| dl | Add | fights | fights+attend | 0.54 | 0.41 | 0.67 | **0.0000** | 4406 | 34 | 129 | 15 | 0.77 | 10.42 |
| dh | Add | attend | attend+steals | 0.35 | 0.28 | 0.43 | **0.0000** | 4493 | 32 | 3910 | 128 | 0.71 | 3.17 |
| dl | Add | steals | steals+attend | 0.59 | 0.43 | 0.74 | **0.0000** | 4493 | 32 | 50 | 9 | 0.71 | 15.25 |
| dh | Add | hyper | hyper+argues | 0.52 | 0.47 | 0.58 | **0.0000** | 3895 | 125 | 843 | 181 | 3.11 | 17.68 |
| dl | Add | argues | argues+hyper | 0.38 | 0.32 | 0.44 | **0.0000** | 3895 | 125 | 1826 | 225 | 3.11 | 10.97 |
| dh | Add | hyper | hyper+temper | 0.52 | 0.46 | 0.58 | **0.0000** | 4922 | 70 | 1530 | 172 | 1.40 | 10.11 |
| dl | Add | temper | temper+hyper | 0.52 | 0.46 | 0.59 | **0.0000** | 4922 | 70 | 966 | 112 | 1.40 | 10.39 |
| dh | Add | hyper | hyper+fights | 0.50 | 0.43 | 0.57 | **0.0000** | 5795 | 29 | 2366 | 111 | 0.50 | 4.48 |
| dl | Add | fights | fights+hyper | 0.53 | 0.41 | 0.65 | **0.0000** | 5795 | 29 | 229 | 17 | 0.50 | 6.91 |
| dh | Add | hyper | hyper+steals | 0.42 | 0.33 | 0.50 | **0.0000** | 5914 | 28 | 2544 | 77 | 0.47 | 2.94 |
| dl | Add | steals | steals+hyper | 0.62 | 0.50 | 0.74 | **0.0000** | 5914 | 28 | 113 | 15 | 0.47 | 11.72 |
| dh | Add | argues | argues+temper | 0.45 | 0.41 | 0.50 | **0.0000** | 4429 | 161 | 1787 | 317 | 3.51 | 15.07 |
| dl | Add | temper | temper+argues | 0.56 | 0.50 | 0.62 | **0.0000** | 4429 | 161 | 344 | 110 | 3.51 | 24.23 |
| dh | Add | argues | argues+fights | 0.42 | 0.36 | 0.49 | **0.0000** | 4900 | 47 | 3178 | 176 | 0.95 | 5.25 |
| dl | Add | fights | fights+argues | 0.57 | 0.45 | 0.70 | **0.0000** | 4900 | 47 | 82 | 15 | 0.95 | 15.46 |
| dh | Add | argues | argues+steals | 0.31 | 0.23 | 0.38 | **0.0000** | 4923 | 46 | 3478 | 116 | 0.93 | 3.23 |
| dl | Add | steals | steals+argues | 0.55 | 0.40 | 0.69 | **0.0000** | 4923 | 46 | 64 | 11 | 0.93 | 14.67 |
| dh | Add | temper | temper+fights | 0.46 | 0.39 | 0.53 | **0.0000** | 6456 | 64 | 1669 | 112 | 0.98 | 6.29 |
| dl | Add | fights | fights+temper | 0.42 | 0.29 | 0.55 | **0.0000** | 6456 | 64 | 160 | 14 | 0.98 | 8.05 |
| dh | Add | temper | temper+steals | 0.33 | 0.24 | 0.42 | **0.0000** | 6492 | 44 | 1971 | 56 | 0.67 | 2.76 |
| dl | Add | steals | steals+temper | 0.54 | 0.42 | 0.66 | **0.0000** | 6492 | 44 | 142 | 16 | 0.67 | 10.13 |
| dh | Add | fights | fights+steals | 0.44 | 0.32 | 0.56 | **0.0000** | 8003 | 29 | 513 | 18 | 0.36 | 3.39 |
| dl | Add | steals | steals+fights | 0.62 | 0.52 | 0.72 | **0.0000** | 8003 | 29 | 245 | 24 | 0.36 | 8.92 |
| dp | Joint Persist | fear+worry | fear+worry | 0.77 | 0.75 | 0.80 | **0.0000** | 5112 | 280 | 726 | 692 | 5.19 | 48.80 |
| dp | Joint Persist | fear+dysph | fear+dysph | 0.73 | 0.69 | 0.77 | **0.0000** | 6297 | 205 | 336 | 219 | 3.15 | 39.46 |
| dp | Joint Persist | fear+worth | fear+worth | 0.77 | 0.73 | 0.80 | **0.0000** | 6318 | 171 | 357 | 237 | 2.64 | 39.90 |
| dp | Joint Persist | fear+attend | fear+attend | 0.82 | 0.80 | 0.85 | **0.0000** | 3824 | 134 | 698 | 635 | 3.39 | 47.64 |
| dp | Joint Persist | fear+hyper | fear+hyper | 0.84 | 0.81 | 0.86 | **0.0000** | 4978 | 98 | 566 | 399 | 1.93 | 41.35 |
| dp | Joint Persist | fear+argues | fear+argues | 0.81 | 0.79 | 0.84 | **0.0000** | 4165 | 124 | 682 | 522 | 2.89 | 43.36 |
| dp | Joint Persist | fear+temper | fear+temper | 0.83 | 0.80 | 0.86 | **0.0000** | 5491 | 95 | 517 | 334 | 1.70 | 39.25 |
| dp | Joint Persist | fear+fights | fear+fights | 0.79 | 0.73 | 0.84 | **0.0000** | 6550 | 47 | 186 | 69 | 0.71 | 27.06 |
| dp | Joint Persist | fear+steals | fear+steals | 0.83 | 0.78 | 0.89 | **0.0000** | 6725 | 31 | 105 | 47 | 0.46 | 30.92 |
| dp | Joint Persist | worry+dysph | worry+dysph | 0.74 | 0.70 | 0.77 | **0.0000** | 5398 | 228 | 439 | 319 | 4.05 | 42.08 |
| dp | Joint Persist | worry+worth | worry+worth | 0.78 | 0.74 | 0.81 | **0.0000** | 5416 | 172 | 450 | 322 | 3.08 | 41.71 |
| dp | Joint Persist | worry+attend | worry+attend | 0.81 | 0.78 | 0.83 | **0.0000** | 3219 | 167 | 895 | 945 | 4.93 | 51.36 |
| dp | Joint Persist | worry+hyper | worry+hyper | 0.81 | 0.79 | 0.84 | **0.0000** | 4197 | 110 | 752 | 523 | 2.55 | 41.02 |
| dp | Joint Persist | worry+argues | worry+argues | 0.80 | 0.77 | 0.82 | **0.0000** | 3592 | 167 | 916 | 837 | 4.44 | 47.75 |
| dp | Joint Persist | worry+temper | worry+temper | 0.80 | 0.78 | 0.83 | **0.0000** | 4687 | 108 | 704 | 435 | 2.25 | 38.19 |
| dp | Joint Persist | worry+fights | worry+fights | 0.78 | 0.73 | 0.83 | **0.0000** | 5551 | 53 | 246 | 95 | 0.95 | 27.86 |
| dp | Joint Persist | worry+steals | worry+steals | 0.83 | 0.78 | 0.88 | **0.0000** | 5712 | 35 | 153 | 69 | 0.61 | 31.08 |
| dp | Joint Persist | dysph+worth | dysph+worth | 0.71 | 0.67 | 0.75 | **0.0000** | 7047 | 252 | 304 | 206 | 3.45 | 40.39 |
| dp | Joint Persist | dysph+attend | dysph+attend | 0.80 | 0.76 | 0.83 | **0.0000** | 4192 | 126 | 462 | 337 | 2.92 | 42.18 |
| dp | Joint Persist | dysph+hyper | dysph+hyper | 0.82 | 0.79 | 0.85 | **0.0000** | 5486 | 84 | 353 | 212 | 1.51 | 37.52 |
| dp | Joint Persist | dysph+argues | dysph+argues | 0.78 | 0.75 | 0.81 | **0.0000** | 4623 | 148 | 461 | 331 | 3.10 | 41.79 |
| dp | Joint Persist | dysph+temper | dysph+temper | 0.80 | 0.77 | 0.83 | **0.0000** | 6111 | 116 | 370 | 228 | 1.86 | 38.13 |
| dp | Joint Persist | dysph+fights | dysph+fights | 0.74 | 0.68 | 0.80 | **0.0000** | 7418 | 58 | 182 | 58 | 0.78 | 24.17 |
| dp | Joint Persist | dysph+steals | dysph+steals | 0.74 | 0.67 | 0.81 | **0.0000** | 7578 | 56 | 99 | 37 | 0.73 | 27.21 |
| dp | Joint Persist | worth+attend | worth+attend | 0.83 | 0.80 | 0.85 | **0.0000** | 4234 | 101 | 496 | 372 | 2.33 | 42.86 |
| dp | Joint Persist | worth+hyper | worth+hyper | 0.82 | 0.79 | 0.85 | **0.0000** | 5486 | 86 | 386 | 233 | 1.54 | 37.64 |
| dp | Joint Persist | worth+argues | worth+argues | 0.83 | 0.80 | 0.86 | **0.0000** | 4593 | 108 | 436 | 338 | 2.30 | 43.67 |
| dp | Joint Persist | worth+temper | worth+temper | 0.80 | 0.77 | 0.84 | **0.0000** | 6052 | 108 | 362 | 221 | 1.75 | 37.91 |
| dp | Joint Persist | worth+fights | worth+fights | 0.77 | 0.70 | 0.83 | **0.0000** | 7339 | 44 | 150 | 49 | 0.60 | 24.62 |
| dp | Joint Persist | worth+steals | worth+steals | 0.77 | 0.70 | 0.84 | **0.0000** | 7552 | 35 | 107 | 34 | 0.46 | 24.11 |
| dp | Joint Persist | attend+hyper | attend+hyper | 0.85 | 0.83 | 0.87 | **0.0000** | 4046 | 173 | 1126 | 1409 | 4.10 | 55.58 |
| dp | Joint Persist | attend+argues | attend+argues | 0.86 | 0.84 | 0.87 | **0.0000** | 3065 | 172 | 994 | 1586 | 5.31 | 61.47 |
| dp | Joint Persist | attend+temper | attend+temper | 0.85 | 0.83 | 0.87 | **0.0000** | 3804 | 99 | 840 | 756 | 2.54 | 47.37 |
| dp | Joint Persist | attend+fights | attend+fights | 0.85 | 0.81 | 0.88 | **0.0000** | 4406 | 34 | 363 | 163 | 0.77 | 30.99 |
| dp | Joint Persist | attend+steals | attend+steals | 0.88 | 0.84 | 0.91 | **0.0000** | 4493 | 32 | 217 | 132 | 0.71 | 37.82 |
| dp | Joint Persist | hyper+argues | hyper+argues | 0.85 | 0.83 | 0.87 | **0.0000** | 3895 | 125 | 931 | 946 | 3.11 | 50.40 |
| dp | Joint Persist | hyper+temper | hyper+temper | 0.87 | 0.85 | 0.89 | **0.0000** | 4922 | 70 | 692 | 507 | 1.40 | 42.29 |
| dp | Joint Persist | hyper+fights | hyper+fights | 0.85 | 0.81 | 0.89 | **0.0000** | 5795 | 29 | 304 | 120 | 0.50 | 28.30 |
| dp | Joint Persist | hyper+steals | hyper+steals | 0.87 | 0.83 | 0.91 | **0.0000** | 5914 | 28 | 187 | 93 | 0.47 | 33.21 |
| dp | Joint Persist | argues+temper | argues+temper | 0.84 | 0.82 | 0.86 | **0.0000** | 4429 | 161 | 907 | 916 | 3.51 | 50.25 |
| dp | Joint Persist | argues+fights | argues+fights | 0.83 | 0.80 | 0.87 | **0.0000** | 4900 | 47 | 390 | 183 | 0.95 | 31.94 |
| dp | Joint Persist | argues+steals | argues+steals | 0.86 | 0.82 | 0.89 | **0.0000** | 4923 | 46 | 205 | 128 | 0.93 | 38.44 |
| dp | Joint Persist | temper+fights | temper+fights | 0.81 | 0.78 | 0.85 | **0.0000** | 6456 | 64 | 341 | 155 | 0.98 | 31.25 |
| dp | Joint Persist | temper+steals | temper+steals | 0.85 | 0.80 | 0.89 | **0.0000** | 6492 | 44 | 162 | 88 | 0.67 | 35.20 |
| dp | Joint Persist | fights+steals | fights+steals | 0.82 | 0.76 | 0.88 | **0.0000** | 8003 | 29 | 101 | 38 | 0.36 | 27.34 |
| cg | Shift | fear | worry | 0.07 | -.00 | 0.14 | 0.0567 | 4786 | 606 | 465 | 76 | 11.24 | 14.05 |
| bj | Shift | worry | fear | 0.07 | -.00 | 0.14 | 0.0665 | 5212 | 180 | 1550 | 70 | 3.34 | 4.32 |
| cg | Shift | fear | dysph | -.04 | -.10 | 0.03 | 0.2790 | 6126 | 376 | 1333 | 71 | 5.78 | 5.06 |
| bj | Shift | dysph | fear | 0.03 | -.05 | 0.11 | 0.4075 | 6020 | 482 | 467 | 43 | 7.41 | 8.43 |
| cg | Shift | fear | worth | 0.02 | -.06 | 0.09 | 0.6462 | 6217 | 272 | 1304 | 61 | 4.19 | 4.47 |
| bj | Shift | worth | fear | -.03 | -.11 | 0.05 | 0.4909 | 5963 | 526 | 485 | 38 | 8.11 | 7.27 |
| cg | Shift | fear | attend | -.13 | -.21 | -.06 | **0.0003** | 3361 | 597 | 564 | 62 | 15.08 | 9.90 |
| bj | Shift | attend | fear | -.25 | -.32 | -.19 | **0.0000** | 3719 | 239 | 2980 | 74 | 6.04 | 2.42 |
| cg | Shift | fear | hyper | -.09 | -.16 | -.01 | **0.0223** | 4730 | 346 | 945 | 49 | 6.82 | 4.93 |
| bj | Shift | hyper | fear | -.17 | -.23 | -.12 | **0.0000** | 4641 | 435 | 1844 | 92 | 8.57 | 4.75 |
| cg | Shift | fear | argues | -.14 | -.20 | -.07 | **0.0000** | 3577 | 712 | 672 | 83 | 16.60 | 10.99 |
| bj | Shift | argues | fear | -.20 | -.26 | -.14 | **0.0000** | 4017 | 272 | 2637 | 86 | 6.34 | 3.16 |
| cg | Shift | fear | temper | -.06 | -.13 | 0.01 | 0.0760 | 5199 | 387 | 1047 | 61 | 6.93 | 5.51 |
| bj | Shift | temper | fear | -.08 | -.15 | -.02 | **0.0092** | 5120 | 466 | 1336 | 90 | 8.34 | 6.31 |
| cg | Shift | fear | fights | -.07 | -.17 | 0.02 | 0.1327 | 6469 | 128 | 1680 | 24 | 1.94 | 1.41 |
| bj | Shift | fights | fear | -.06 | -.14 | 0.02 | 0.1433 | 5895 | 702 | 380 | 35 | 10.64 | 8.43 |
| cg | Shift | fear | steals | -.10 | -.20 | 0.01 | 0.0676 | 6656 | 100 | 1790 | 17 | 1.48 | 0.94 |
| bj | Shift | steals | fear | -.04 | -.13 | 0.06 | 0.4387 | 6021 | 735 | 232 | 24 | 10.88 | 9.38 |
| cg | Shift | worry | dysph | -.03 | -.10 | 0.03 | 0.3176 | 5402 | 224 | 2200 | 80 | 3.98 | 3.51 |
| bj | Shift | dysph | worry | 0.04 | -.04 | 0.13 | 0.2900 | 4868 | 758 | 259 | 48 | 13.47 | 15.64 |
| cg | Shift | worry | worth | -.06 | -.14 | 0.01 | 0.1124 | 5416 | 172 | 2211 | 55 | 3.08 | 2.43 |
| bj | Shift | worth | worry | -.00 | -.08 | 0.08 | 0.9314 | 4785 | 803 | 296 | 49 | 14.37 | 14.20 |
| cg | Shift | worry | attend | -.23 | -.29 | -.16 | **0.0000** | 2923 | 463 | 1117 | 81 | 13.67 | 6.76 |
| bj | Shift | attend | worry | -.32 | -.38 | -.26 | **0.0000** | 3005 | 381 | 2446 | 101 | 11.25 | 3.97 |
| cg | Shift | worry | hyper | -.12 | -.19 | -.05 | **0.0012** | 4047 | 260 | 1692 | 71 | 6.04 | 4.03 |
| bj | Shift | hyper | worry | -.24 | -.29 | -.18 | **0.0000** | 3668 | 639 | 1508 | 118 | 14.84 | 7.26 |
| cg | Shift | worry | argues | -.13 | -.19 | -.07 | **0.0000** | 3203 | 556 | 1155 | 130 | 14.79 | 10.12 |
| bj | Shift | argues | worry | -.26 | -.31 | -.20 | **0.0000** | 3312 | 447 | 2059 | 115 | 11.89 | 5.29 |
| cg | Shift | worry | temper | -.13 | -.20 | -.06 | **0.0001** | 4495 | 300 | 1824 | 75 | 6.26 | 3.95 |
| bj | Shift | temper | worry | -.19 | -.25 | -.13 | **0.0000** | 4084 | 711 | 1044 | 94 | 14.83 | 8.26 |
| cg | Shift | worry | fights | -.10 | -.19 | -.01 | **0.0251** | 5504 | 100 | 2666 | 31 | 1.78 | 1.15 |
| bj | Shift | fights | worry | -.12 | -.20 | -.04 | **0.0038** | 4627 | 977 | 291 | 38 | 17.43 | 11.55 |
| cg | Shift | worry | steals | -.11 | -.21 | -.01 | **0.0316** | 5668 | 79 | 2792 | 24 | 1.37 | 0.85 |
| bj | Shift | steals | worry | -.17 | -.27 | -.07 | **0.0011** | 4731 | 1016 | 169 | 17 | 17.68 | 9.14 |
| cg | Shift | dysph | worth | 0.02 | -.07 | 0.11 | 0.6434 | 6975 | 324 | 528 | 27 | 4.44 | 4.86 |
| bj | Shift | worth | dysph | -.02 | -.10 | 0.06 | 0.6241 | 6842 | 457 | 572 | 35 | 6.26 | 5.77 |
| cg | Shift | dysph | attend | -.05 | -.14 | 0.04 | 0.2794 | 3628 | 690 | 230 | 36 | 15.98 | 13.53 |
| bj | Shift | attend | dysph | -.23 | -.30 | -.16 | **0.0000** | 4117 | 201 | 3517 | 71 | 4.65 | 1.98 |
| cg | Shift | dysph | hyper | 0.02 | -.07 | 0.10 | 0.7009 | 5151 | 419 | 460 | 40 | 7.52 | 8.00 |
| bj | Shift | hyper | dysph | -.18 | -.24 | -.13 | **0.0000** | 5142 | 428 | 2241 | 95 | 7.68 | 4.07 |
| cg | Shift | dysph | argues | -.01 | -.10 | 0.07 | 0.7308 | 3964 | 807 | 229 | 44 | 16.91 | 16.12 |
| bj | Shift | argues | dysph | -.22 | -.28 | -.15 | **0.0000** | 4551 | 220 | 3071 | 64 | 4.61 | 2.04 |
| cg | Shift | dysph | temper | -.05 | -.14 | 0.04 | 0.3049 | 5791 | 436 | 440 | 27 | 7.00 | 5.78 |
| bj | Shift | temper | dysph | -.11 | -.18 | -.05 | **0.0004** | 5800 | 427 | 1602 | 77 | 6.86 | 4.59 |
| cg | Shift | dysph | fights | 0.02 | -.09 | 0.12 | 0.7714 | 7333 | 143 | 808 | 17 | 1.91 | 2.06 |
| bj | Shift | fights | dysph | -.05 | -.14 | 0.03 | 0.1869 | 6763 | 713 | 397 | 33 | 9.54 | 7.67 |
| cg | Shift | dysph | steals | 0.04 | -.07 | 0.16 | 0.4649 | 7541 | 93 | 915 | 14 | 1.22 | 1.51 |
| bj | Shift | steals | dysph | 0.03 | -.06 | 0.12 | 0.5073 | 6913 | 721 | 243 | 29 | 9.44 | 10.66 |
| cg | Shift | worth | attend | -.12 | -.21 | -.03 | **0.0113** | 3614 | 721 | 222 | 27 | 16.63 | 10.84 |
| bj | Shift | attend | worth | -.28 | -.35 | -.21 | **0.0000** | 4165 | 170 | 3473 | 46 | 3.92 | 1.31 |
| cg | Shift | worth | hyper | -.08 | -.16 | 0.01 | 0.0932 | 5148 | 424 | 470 | 28 | 7.61 | 5.62 |
| bj | Shift | hyper | worth | -.14 | -.20 | -.07 | **0.0000** | 5246 | 326 | 2200 | 82 | 5.85 | 3.59 |
| cg | Shift | worth | argues | -.09 | -.17 | -.01 | **0.0322** | 3879 | 822 | 298 | 45 | 17.49 | 13.12 |
| bj | Shift | argues | worth | -.24 | -.31 | -.17 | **0.0000** | 4500 | 201 | 3100 | 53 | 4.28 | 1.68 |
| cg | Shift | worth | temper | -.02 | -.11 | 0.06 | 0.5979 | 5731 | 429 | 500 | 34 | 6.96 | 6.37 |
| bj | Shift | temper | worth | -.12 | -.19 | -.05 | **0.0005** | 5818 | 342 | 1634 | 60 | 5.55 | 3.54 |
| cg | Shift | worth | fights | 0.02 | -.08 | 0.12 | 0.7264 | 7235 | 148 | 898 | 20 | 2.00 | 2.18 |
| bj | Shift | fights | worth | -.03 | -.11 | 0.06 | 0.5467 | 6810 | 573 | 438 | 33 | 7.76 | 7.01 |
| cg | Shift | worth | steals | -.08 | -.20 | 0.04 | 0.1989 | 7471 | 116 | 966 | 10 | 1.53 | 1.02 |
| bj | Shift | steals | worth | 0.04 | -.05 | 0.14 | 0.3541 | 6997 | 590 | 242 | 25 | 7.78 | 9.36 |
| cg | Shift | attend | hyper | -.06 | -.16 | 0.03 | 0.2031 | 4120 | 99 | 1818 | 34 | 2.35 | 1.84 |
| bj | Shift | hyper | attend | -.05 | -.13 | 0.03 | 0.2508 | 3622 | 597 | 322 | 44 | 14.15 | 12.02 |
| cg | Shift | attend | argues | -.26 | -.32 | -.20 | **0.0000** | 2843 | 394 | 1710 | 97 | 12.17 | 5.37 |
| bj | Shift | argues | attend | -.28 | -.35 | -.22 | **0.0000** | 2838 | 399 | 1282 | 66 | 12.33 | 4.90 |
| cg | Shift | attend | temper | -.24 | -.32 | -.17 | **0.0000** | 3733 | 170 | 2743 | 48 | 4.36 | 1.72 |
| bj | Shift | temper | attend | -.12 | -.19 | -.05 | **0.0006** | 3296 | 607 | 608 | 73 | 15.55 | 10.72 |
| cg | Shift | attend | fights | -.24 | -.36 | -.13 | **0.0000** | 4393 | 47 | 3848 | 13 | 1.06 | 0.34 |
| bj | Shift | fights | attend | 0.03 | -.07 | 0.13 | 0.5481 | 3634 | 806 | 115 | 29 | 18.15 | 20.14 |
| cg | Shift | attend | steals | -.22 | -.37 | -.07 | **0.0042** | 4501 | 24 | 4031 | 7 | 0.53 | 0.17 |
| bj | Shift | steals | attend | 0.11 | -.02 | 0.25 | 0.0948 | 3698 | 827 | 43 | 16 | 18.28 | 27.12 |
| cg | Shift | hyper | argues | -.19 | -.25 | -.12 | **0.0000** | 3383 | 637 | 931 | 93 | 15.85 | 9.08 |
| bj | Shift | argues | hyper | -.16 | -.24 | -.09 | **0.0000** | 3839 | 181 | 2001 | 50 | 4.50 | 2.44 |
| cg | Shift | hyper | temper | -.06 | -.13 | 0.00 | 0.0653 | 4696 | 296 | 1621 | 81 | 5.93 | 4.76 |
| bj | Shift | temper | hyper | -.07 | -.14 | 0.00 | 0.0565 | 4651 | 341 | 1021 | 57 | 6.83 | 5.29 |
| cg | Shift | hyper | fights | -.04 | -.14 | 0.05 | 0.3699 | 5739 | 85 | 2447 | 30 | 1.46 | 1.21 |
| bj | Shift | fights | hyper | 0.00 | -.10 | 0.10 | 0.9345 | 5312 | 512 | 224 | 22 | 8.79 | 8.94 |
| cg | Shift | hyper | steals | 0.07 | -.03 | 0.17 | 0.1882 | 5888 | 54 | 2589 | 32 | 0.91 | 1.22 |
| bj | Shift | steals | hyper | 0.08 | -.03 | 0.20 | 0.1597 | 5421 | 521 | 112 | 16 | 8.77 | 12.50 |
| cg | Shift | argues | temper | -.04 | -.13 | 0.05 | 0.4243 | 4489 | 101 | 2064 | 40 | 2.20 | 1.90 |
| bj | Shift | temper | argues | -.02 | -.10 | 0.05 | 0.5942 | 3890 | 700 | 389 | 65 | 15.25 | 14.32 |
| cg | Shift | argues | fights | -.08 | -.23 | 0.07 | 0.2828 | 4925 | 22 | 3344 | 10 | 0.44 | 0.30 |
| bj | Shift | fights | argues | 0.10 | -.01 | 0.21 | 0.0682 | 3999 | 948 | 71 | 26 | 19.16 | 26.80 |
| cg | Shift | argues | steals | -.01 | -.16 | 0.15 | 0.9484 | 4952 | 17 | 3582 | 12 | 0.34 | 0.33 |
| bj | Shift | steals | argues | 0.10 | -.03 | 0.22 | 0.1216 | 4010 | 959 | 55 | 20 | 19.30 | 26.67 |
| cg | Shift | temper | fights | 0.10 | -.00 | 0.21 | 0.0607 | 6465 | 55 | 1757 | 24 | 0.84 | 1.35 |
| bj | Shift | fights | temper | 0.10 | -.01 | 0.21 | 0.0637 | 6000 | 520 | 153 | 21 | 7.98 | 12.07 |
| cg | Shift | Temper | steals | -.13 | -.24 | -.02 | **0.0256** | 6458 | 78 | 2014 | 13 | 1.19 | 0.64 |
| bj | Shift | Steals | temper | 0.13 | 0.02 | 0.24 | **0.0166** | 5999 | 537 | 136 | 22 | 8.22 | 13.92 |
| cg | Shift | Fights | steals | 0.02 | -.10 | 0.15 | 0.7131 | 7898 | 134 | 521 | 10 | 1.67 | 1.88 |
| bj | Shift | Steals | fights | 0.04 | -.10 | 0.17 | 0.5688 | 7838 | 194 | 261 | 8 | 2.42 | 2.97 |
| co | Subtract x | fear+worry | worry | 0.23 | 0.18 | 0.28 | **0.0000** | 4786 | 606 | 1127 | 291 | 11.24 | 20.52 |
| co | Subtract x | fear+dysph | dysph | 0.20 | 0.13 | 0.27 | **0.0000** | 6126 | 376 | 489 | 66 | 5.78 | 11.89 |
| co | Subtract x | fear+worth | worth | 0.30 | 0.23 | 0.37 | **0.0000** | 6217 | 272 | 519 | 75 | 4.19 | 12.63 |
| co | Subtract x | fear+attend | attend | 0.36 | 0.31 | 0.40 | **0.0000** | 3361 | 597 | 884 | 449 | 15.08 | 33.68 |
| co | Subtract x | fear+hyper | hyper | 0.40 | 0.35 | 0.45 | **0.0000** | 4730 | 346 | 752 | 213 | 6.82 | 22.07 |
| co | Subtract x | fear+argues | argues | 0.24 | 0.19 | 0.29 | **0.0000** | 3577 | 712 | 854 | 350 | 16.60 | 29.07 |
| co | Subtract x | fear+temper | temper | 0.28 | 0.22 | 0.34 | **0.0000** | 5199 | 387 | 711 | 140 | 6.93 | 16.45 |
| co | Subtract x | fear+fights | fights | 0.48 | 0.40 | 0.57 | **0.0000** | 6469 | 128 | 216 | 39 | 1.94 | 15.29 |
| co | Subtract x | fear+steals | steals | 0.52 | 0.42 | 0.62 | **0.0000** | 6656 | 100 | 127 | 25 | 1.48 | 16.45 |
| co | Subtract x | worry+dysph | dysph | 0.18 | 0.10 | 0.25 | **0.0000** | 5402 | 224 | 700 | 58 | 3.98 | 7.65 |
| co | Subtract x | worry+worth | worth | 0.32 | 0.24 | 0.39 | **0.0000** | 5416 | 172 | 696 | 76 | 3.08 | 9.84 |
| co | Subtract x | worry+attend | attend | 0.30 | 0.25 | 0.35 | **0.0000** | 2923 | 463 | 1335 | 505 | 13.67 | 27.45 |
| co | Subtract x | worry+hyper | hyper | 0.37 | 0.31 | 0.42 | **0.0000** | 4047 | 260 | 1048 | 227 | 6.04 | 17.80 |
| co | Subtract x | worry+argues | argues | 0.22 | 0.17 | 0.27 | **0.0000** | 3203 | 556 | 1319 | 434 | 14.79 | 24.76 |
| co | Subtract x | worry+temper | temper | 0.31 | 0.25 | 0.36 | **0.0000** | 4495 | 300 | 959 | 180 | 6.26 | 15.80 |
| co | Subtract x | worry+fights | fights | 0.48 | 0.39 | 0.57 | **0.0000** | 5504 | 100 | 297 | 44 | 1.78 | 12.90 |
| co | Subtract x | worry+steals | steals | 0.44 | 0.33 | 0.55 | **0.0000** | 5668 | 79 | 199 | 23 | 1.37 | 10.36 |
| co | Subtract x | dysph+worth | worth | 0.34 | 0.27 | 0.41 | **0.0000** | 6975 | 324 | 432 | 78 | 4.44 | 15.29 |
| co | Subtract x | dysph+attend | attend | 0.39 | 0.34 | 0.44 | **0.0000** | 3628 | 690 | 485 | 314 | 15.98 | 39.30 |
| co | Subtract x | dysph+hyper | hyper | 0.39 | 0.33 | 0.45 | **0.0000** | 5151 | 419 | 426 | 139 | 7.52 | 24.60 |
| co | Subtract x | dysph+argues | argues | 0.29 | 0.24 | 0.35 | **0.0000** | 3964 | 807 | 524 | 268 | 16.91 | 33.84 |
| co | Subtract x | dysph+temper | temper | 0.32 | 0.26 | 0.38 | **0.0000** | 5791 | 436 | 481 | 117 | 7.00 | 19.57 |
| co | Subtract x | dysph+fights | fights | 0.46 | 0.36 | 0.55 | **0.0000** | 7333 | 143 | 206 | 34 | 1.91 | 14.17 |
| co | Subtract x | dysph+steals | steals | 0.56 | 0.46 | 0.66 | **0.0000** | 7541 | 93 | 112 | 24 | 1.22 | 17.65 |
| co | Subtract x | worth+attend | attend | 0.38 | 0.33 | 0.43 | **0.0000** | 3614 | 721 | 528 | 340 | 16.63 | 39.17 |
| co | Subtract x | worth+hyper | hyper | 0.44 | 0.39 | 0.50 | **0.0000** | 5148 | 424 | 444 | 175 | 7.61 | 28.27 |
| co | Subtract x | worth+argues | argues | 0.28 | 0.22 | 0.33 | **0.0000** | 3879 | 822 | 515 | 259 | 17.49 | 33.46 |
| co | Subtract x | worth+temper | temper | 0.36 | 0.30 | 0.42 | **0.0000** | 5731 | 429 | 456 | 127 | 6.96 | 21.78 |
| co | Subtract x | worth+fights | fights | 0.43 | 0.34 | 0.53 | **0.0000** | 7235 | 148 | 171 | 28 | 2.00 | 14.07 |
| co | Subtract x | worth+steals | steals | 0.55 | 0.45 | 0.64 | **0.0000** | 7471 | 116 | 114 | 27 | 1.53 | 19.15 |
| co | Subtract x | attend+hyper | hyper | 0.11 | 0.04 | 0.19 | **0.0039** | 4120 | 99 | 2445 | 90 | 2.35 | 3.55 |
| co | Subtract x | attend+argues | argues | -.01 | -.07 | 0.04 | 0.6176 | 2843 | 394 | 2277 | 303 | 12.17 | 11.74 |
| co | Subtract x | attend+temper | temper | 0.17 | 0.10 | 0.23 | **0.0000** | 3733 | 170 | 1476 | 120 | 4.36 | 7.52 |
| co | Subtract x | attend+fights | fights | 0.25 | 0.12 | 0.38 | **0.0002** | 4393 | 47 | 509 | 17 | 1.06 | 3.23 |
| co | Subtract x | attend+steals | steals | 0.40 | 0.26 | 0.55 | **0.0000** | 4501 | 24 | 336 | 13 | 0.53 | 3.72 |
| co | Subtract x | hyper+argues | argues | 0.18 | 0.13 | 0.23 | **0.0000** | 3383 | 637 | 1426 | 451 | 15.85 | 24.03 |
| co | Subtract x | hyper+temper | temper | 0.29 | 0.24 | 0.35 | **0.0000** | 4696 | 296 | 1023 | 176 | 5.93 | 14.68 |
| co | Subtract x | hyper+fights | fights | 0.38 | 0.28 | 0.48 | **0.0000** | 5739 | 85 | 392 | 32 | 1.46 | 7.55 |
| co | Subtract x | hyper+steals | steals | 0.49 | 0.39 | 0.60 | **0.0000** | 5888 | 54 | 255 | 25 | 0.91 | 8.93 |
| co | Subtract x | argues+temper | temper | 0.22 | 0.15 | 0.30 | **0.0000** | 4489 | 101 | 1732 | 91 | 2.20 | 4.99 |
| co | Subtract x | argues+fights | fights | 0.39 | 0.25 | 0.52 | **0.0000** | 4925 | 22 | 557 | 16 | 0.44 | 2.79 |
| co | Subtract x | argues+steals | steals | 0.45 | 0.29 | 0.60 | **0.0000** | 4952 | 17 | 322 | 11 | 0.34 | 3.30 |
| co | Subtract x | temper+fights | fights | 0.40 | 0.29 | 0.50 | **0.0000** | 6465 | 55 | 470 | 26 | 0.84 | 5.24 |
| co | Subtract x | temper+steals | steals | 0.44 | 0.33 | 0.55 | **0.0000** | 6458 | 78 | 227 | 23 | 1.19 | 9.20 |
| bn | Subtract y | worry+fear | fear | 0.29 | 0.23 | 0.36 | **0.0000** | 5212 | 180 | 1289 | 129 | 3.34 | 9.10 |
| bn | Subtract y | dysph+fear | fear | 0.33 | 0.27 | 0.39 | **0.0000** | 6020 | 482 | 437 | 118 | 7.41 | 21.26 |
| bn | Subtract y | worth+fear | fear | 0.31 | 0.25 | 0.37 | **0.0000** | 5963 | 526 | 467 | 127 | 8.11 | 21.38 |
| bn | Subtract y | attend+fear | fear | 0.04 | -.03 | 0.11 | 0.2648 | 3719 | 239 | 1241 | 92 | 6.04 | 6.90 |
| bn | Subtract y | hyper+fear | fear | 0.15 | 0.09 | 0.21 | **0.0000** | 4641 | 435 | 835 | 130 | 8.57 | 13.47 |
| bn | Subtract y | argues+fear | fear | 0.21 | 0.15 | 0.27 | **0.0000** | 4017 | 272 | 1058 | 146 | 6.34 | 12.13 |
| bn | Subtract y | temper+fear | fear | 0.30 | 0.24 | 0.35 | **0.0000** | 5120 | 466 | 680 | 171 | 8.34 | 20.09 |
| bn | Subtract y | fights+fear | fear | 0.26 | 0.19 | 0.34 | **0.0000** | 5895 | 702 | 190 | 65 | 10.64 | 25.49 |
| bn | Subtract y | steals+fear | fear | 0.23 | 0.14 | 0.33 | **0.0000** | 6021 | 735 | 114 | 38 | 10.88 | 25.00 |
| bn | Subtract y | dysph+worry | worry | 0.25 | 0.20 | 0.31 | **0.0000** | 4868 | 758 | 557 | 201 | 13.47 | 26.52 |
| bn | Subtract y | worth+worry | worry | 0.26 | 0.21 | 0.32 | **0.0000** | 4785 | 803 | 553 | 219 | 14.37 | 28.37 |
| bn | Subtract y | attend+worry | worry | -.05 | -.11 | 0.01 | 0.0985 | 3005 | 381 | 1660 | 180 | 11.25 | 9.78 |
| bn | Subtract y | hyper+worry | worry | 0.14 | 0.09 | 0.20 | **0.0000** | 3668 | 639 | 1004 | 271 | 14.84 | 21.25 |
| bn | Subtract y | argues+worry | worry | 0.07 | 0.01 | 0.12 | **0.0168** | 3312 | 447 | 1504 | 249 | 11.89 | 14.20 |
| bn | Subtract y | temper+worry | worry | 0.23 | 0.17 | 0.28 | **0.0000** | 4084 | 711 | 845 | 294 | 14.83 | 25.81 |
| bn | Subtract y | fights+worry | worry | 0.24 | 0.17 | 0.31 | **0.0000** | 4627 | 977 | 227 | 114 | 17.43 | 33.43 |
| bn | Subtract y | steals+worry | worry | 0.21 | 0.13 | 0.28 | **0.0000** | 4731 | 1016 | 151 | 71 | 17.68 | 31.98 |
| bn | Subtract y | worth+dysph | dysph | 0.23 | 0.16 | 0.30 | **0.0000** | 6842 | 457 | 437 | 73 | 6.26 | 14.31 |
| bn | Subtract y | attend+dysph | dysph | 0.09 | 0.01 | 0.18 | **0.0315** | 4117 | 201 | 747 | 52 | 4.65 | 6.51 |
| bn | Subtract y | hyper+dysph | dysph | 0.16 | 0.09 | 0.23 | **0.0000** | 5142 | 428 | 490 | 75 | 7.68 | 13.27 |
| bn | Subtract y | argues+dysph | dysph | 0.15 | 0.07 | 0.23 | **0.0003** | 4551 | 220 | 730 | 62 | 4.61 | 7.83 |
| bn | Subtract y | temper+dysph | dysph | 0.21 | 0.14 | 0.28 | **0.0000** | 5800 | 427 | 514 | 84 | 6.86 | 14.05 |
| bn | Subtract y | fights+dysph | dysph | 0.26 | 0.18 | 0.34 | **0.0000** | 6763 | 713 | 183 | 57 | 9.54 | 23.75 |
| bn | Subtract y | steals+dysph | dysph | 0.23 | 0.13 | 0.33 | **0.0000** | 6913 | 721 | 105 | 31 | 9.44 | 22.79 |
| bn | Subtract y | attend+worth | worth | 0.06 | -.03 | 0.15 | 0.1706 | 4165 | 170 | 825 | 43 | 3.92 | 4.95 |
| bn | Subtract y | hyper+worth | worth | 0.18 | 0.11 | 0.26 | **0.0000** | 5246 | 326 | 550 | 69 | 5.85 | 11.15 |
| bn | Subtract y | argues+worth | worth | 0.08 | -.00 | 0.17 | 0.0635 | 4500 | 201 | 729 | 45 | 4.28 | 5.81 |
| bn | Subtract y | temper+worth | worth | 0.29 | 0.22 | 0.36 | **0.0000** | 5818 | 342 | 494 | 89 | 5.55 | 15.27 |
| bn | Subtract y | fights+worth | worth | 0.38 | 0.30 | 0.45 | **0.0000** | 6810 | 573 | 141 | 58 | 7.76 | 29.15 |
| bn | Subtract y | steals+worth | worth | 0.31 | 0.22 | 0.41 | **0.0000** | 6997 | 590 | 105 | 36 | 7.78 | 25.53 |
| bn | Subtract y | hyper+attend | attend | 0.26 | 0.22 | 0.30 | **0.0000** | 3622 | 597 | 1884 | 651 | 14.15 | 25.68 |
| bn | Subtract y | argues+attend | attend | 0.11 | 0.06 | 0.16 | **0.0000** | 2838 | 399 | 2163 | 417 | 12.33 | 16.16 |
| bn | Subtract y | temper+attend | attend | 0.33 | 0.28 | 0.37 | **0.0000** | 3296 | 607 | 1083 | 513 | 15.55 | 32.14 |
| bn | Subtract y | fights+attend | attend | 0.47 | 0.42 | 0.52 | **0.0000** | 3634 | 806 | 257 | 269 | 18.15 | 51.14 |
| bn | Subtract y | steals+attend | attend | 0.40 | 0.34 | 0.47 | **0.0000** | 3698 | 827 | 183 | 166 | 18.28 | 47.56 |
| bn | Subtract y | argues+hyper | hyper | 0.25 | 0.19 | 0.31 | **0.0000** | 3839 | 181 | 1691 | 186 | 4.50 | 9.91 |
| bn | Subtract y | temper+hyper | hyper | 0.40 | 0.35 | 0.45 | **0.0000** | 4651 | 341 | 937 | 262 | 6.83 | 21.85 |
| bn | Subtract y | fights+hyper | hyper | 0.48 | 0.42 | 0.54 | **0.0000** | 5312 | 512 | 272 | 152 | 8.79 | 35.85 |
| bn | Subtract y | steals+hyper | hyper | 0.45 | 0.39 | 0.52 | **0.0000** | 5421 | 521 | 180 | 100 | 8.77 | 35.71 |
| bn | Subtract y | temper+argues | argues | 0.25 | 0.21 | 0.30 | **0.0000** | 3890 | 700 | 1326 | 497 | 15.25 | 27.26 |
| bn | Subtract y | fights+argues | argues | 0.42 | 0.37 | 0.47 | **0.0000** | 3999 | 948 | 294 | 279 | 19.16 | 48.69 |
| bn | Subtract y | steals+argues | argues | 0.35 | 0.28 | 0.41 | **0.0000** | 4010 | 959 | 185 | 148 | 19.30 | 44.44 |
| bn | Subtract y | fights+temper | temper | 0.51 | 0.46 | 0.56 | **0.0000** | 6000 | 520 | 317 | 179 | 7.98 | 36.09 |
| bn | Subtract y | steals+temper | temper | 0.37 | 0.29 | 0.44 | **0.0000** | 5999 | 537 | 179 | 71 | 8.22 | 28.40 |
| bn | Subtract y | steals+fights | fights | 0.49 | 0.40 | 0.59 | **0.0000** | 7838 | 194 | 109 | 30 | 2.42 | 21.58 |

| Supplemental Table S3J. Tetrachoric correlations from 4 x 4 pairwise analyses of all 90 combinations of the 10 selected psychological problems with every other problem to quantify the associations of each baseline problem with outcomes in the **second annual follow-up** that define four parsed paths traditionally conflated in estimates of change: *add path* (x_1_ at baseline and x_2_ + y_2_ at follow-up); *joint persist path* parsed paths traditionally conflated in estimates of change: ***add path*** (x_1_ at baseline and x_2_ + y_2_ at follow-up); ***joint persist path*** (x_1_ + y_1_ at baseline and x_2_ + y_2_ at follow-up); ***shift path*** (only x_1_ at baseline and only y_2_ at follow-up);  ***subtract x path*** (x_1_ + y_1_ at baseline and only y_2_ at follow-up); and ***subtract y path*** (x_1_ + y_1_ at baseline and only x_2_ at follow-up). Results are for problems dichotomized at the **high rating cut (0 or 1 vs 2).** r_t_ = tetrachoric correlation. –CL and +CL = lower and upper 95% confidence intervals for the tetrachoric correlation. c00 = number without the predictor or the outcome; c01 = number without predictor with the outcome; c10 = number with the predictor without the outcome; c11 = number with the predictor and the outcome. Base% = percent of the outcome only x_2_ among children with neither x_1_ nor the other member of the pair, y_1_, at baseline. Obs% = Observed percent of outcomes on the indicated path. Raw P values significant after FDR adjustment in **bold**. | | | | | | | | | | | | | |
| --- | --- | --- | --- | --- | --- | --- | --- | --- | --- | --- | --- | --- | --- |
| prefix | Parsed paths | Predictor | Outcome | r_t_ | -CL | +CL | P | c00 | c01 | c10 | c11 | Base% | Obs% |
| dh | Add | fear | fear+worry | 0.39 | 0.23 | 0.55 | **0.0000** | 8416 | 74 | 80 | 7 | 0.87 | 8.05 |
| dl | Add | worry | worry+fear | 0.43 | 0.32 | 0.54 | **0.0000** | 8416 | 74 | 240 | 19 | 0.87 | 7.34 |
| dh | Add | fear | fear+dysph | 0.43 | 0.27 | 0.59 | **0.0000** | 8682 | 26 | 190 | 7 | 0.30 | 3.55 |
| dl | Add | dysph | dysph+fear | 0.41 | 0.15 | 0.67 | **0.0018** | 8682 | 26 | 39 | 2 | 0.30 | 4.88 |
| dh | Add | fear | fear+worth | 0.53 | 0.39 | 0.67 | **0.0000** | 8675 | 23 | 180 | 10 | 0.26 | 5.26 |
| dl | Add | worth | worth+fear | 0.28 | -.05 | 0.60 | 0.0971 | 8675 | 23 | 50 | 1 | 0.26 | 1.96 |
| dh | Add | fear | fear+attend | 0.48 | 0.30 | 0.65 | **0.0000** | 7738 | 24 | 111 | 6 | 0.31 | 5.13 |
| dl | Add | attend | attend+fear | 0.43 | 0.32 | 0.54 | **0.0000** | 7738 | 24 | 962 | 25 | 0.31 | 2.53 |
| dh | Add | fear | fear+hyper | 0.61 | 0.44 | 0.78 | **0.0000** | 8179 | 8 | 140 | 6 | 0.10 | 4.11 |
| dl | Add | hyper | hyper+fear | 0.62 | 0.50 | 0.74 | **0.0000** | 8179 | 8 | 545 | 17 | 0.10 | 3.02 |
| dh | Add | fear | fear+argues | 0.33 | 0.10 | 0.55 | **0.0042** | 8024 | 22 | 138 | 3 | 0.27 | 2.13 |
| dl | Add | argues | argues+fear | 0.43 | 0.30 | 0.56 | **0.0000** | 8024 | 22 | 686 | 17 | 0.27 | 2.42 |
| dh | Add | fear | fear+temper | 0.50 | 0.31 | 0.69 | **0.0000** | 8447 | 13 | 156 | 5 | 0.15 | 3.11 |
| dl | Add | temper | temper+fear | 0.37 | 0.16 | 0.58 | **0.0006** | 8447 | 13 | 285 | 4 | 0.15 | 1.38 |
| dh | Add | fear | fear+fights | . | . | . | . | 8716 | 0 | 213 | 2 | 0.00 | 0.93 |
| dl | Add | fights | fights+fear | . | . | . | . | 8716 | 0 | 33 | 0 | 0.00 | 0.00 |
| dh | Add | fear | fear+steals | 0.37 | -.00 | 0.75 | 0.0501 | 8707 | 3 | 217 | 1 | 0.03 | 0.46 |
| dl | Add | steals | steals+fear | . | . | . | . | 8707 | 3 | 39 | 0 | 0.03 | 0.00 |
| dh | Add | worry | worry+dysph | 0.46 | 0.32 | 0.59 | **0.0000** | 8518 | 27 | 347 | 13 | 0.32 | 3.61 |
| dl | Add | dysph | dysph+worry | 0.52 | 0.30 | 0.74 | **0.0000** | 8518 | 27 | 29 | 3 | 0.32 | 9.38 |
| dh | Add | worry | worry+worth | 0.44 | 0.30 | 0.58 | **0.0000** | 8513 | 25 | 339 | 11 | 0.29 | 3.14 |
| dl | Add | worth | worth+worry | 0.42 | 0.16 | 0.68 | **0.0014** | 8513 | 25 | 37 | 2 | 0.29 | 5.13 |
| dh | Add | worry | worry+attend | 0.45 | 0.30 | 0.59 | **0.0000** | 7610 | 28 | 231 | 10 | 0.37 | 4.15 |
| dl | Add | attend | attend+worry | 0.54 | 0.45 | 0.63 | **0.0000** | 7610 | 28 | 895 | 44 | 0.37 | 4.69 |
| dh | Add | worry | worry+hyper | 0.48 | 0.31 | 0.65 | **0.0000** | 8021 | 12 | 293 | 7 | 0.15 | 2.33 |
| dl | Add | hyper | hyper+worry | 0.64 | 0.54 | 0.74 | **0.0000** | 8021 | 12 | 519 | 25 | 0.15 | 4.60 |
| dh | Add | worry | worry+argues | 0.23 | 0.03 | 0.43 | **0.0216** | 7881 | 29 | 273 | 4 | 0.37 | 1.44 |
| dl | Add | argues | argues+worry | 0.56 | 0.47 | 0.65 | **0.0000** | 7881 | 29 | 630 | 37 | 0.37 | 5.55 |
| dh | Add | worry | worry+temper | 0.38 | 0.21 | 0.54 | **0.0000** | 8281 | 22 | 311 | 7 | 0.26 | 2.20 |
| dl | Add | temper | temper+worry | 0.56 | 0.44 | 0.69 | **0.0000** | 8281 | 22 | 259 | 15 | 0.26 | 5.47 |
| dh | Add | worry | worry+fights | 0.46 | 0.04 | 0.87 | **0.0315** | 8546 | 1 | 383 | 1 | 0.01 | 0.26 |
| dl | Add | fights | fights+worry | . | . | . | . | 8546 | 1 | 30 | 0 | 0.01 | 0.00 |
| dh | Add | worry | worry+steals | 0.57 | 0.25 | 0.88 | **0.0004** | 8539 | 1 | 386 | 2 | 0.01 | 0.52 |
| dl | Add | steals | steals+worry | 0.80 | 0.57 | 1.00 | **0.0000** | 8539 | 1 | 35 | 2 | 0.01 | 5.41 |
| dh | Add | dysph | dysph+worth | 0.40 | 0.14 | 0.66 | **0.0023** | 8816 | 24 | 46 | 2 | 0.27 | 4.17 |
| dl | Add | worth | worth+dysph | 0.36 | 0.10 | 0.62 | **0.0060** | 8816 | 24 | 63 | 2 | 0.27 | 3.08 |
| dh | Add | dysph | dysph+attend | 0.40 | 0.06 | 0.75 | **0.0218** | 7835 | 14 | 29 | 1 | 0.18 | 3.33 |
| dl | Add | attend | attend+dysph | 0.47 | 0.35 | 0.60 | **0.0000** | 7835 | 14 | 1035 | 21 | 0.18 | 1.99 |
| dh | Add | dysph | dysph+hyper | 0.64 | 0.37 | 0.90 | **0.0000** | 8282 | 4 | 45 | 2 | 0.05 | 4.26 |
| dl | Add | hyper | hyper+dysph | 0.65 | 0.52 | 0.79 | **0.0000** | 8282 | 4 | 605 | 14 | 0.05 | 2.26 |
| dh | Add | dysph | dysph+argues | 0.51 | 0.25 | 0.77 | **0.0001** | 8141 | 18 | 26 | 2 | 0.22 | 7.14 |
| dl | Add | argues | argues+dysph | 0.41 | 0.28 | 0.55 | **0.0000** | 8141 | 18 | 732 | 14 | 0.22 | 1.88 |
| dh | Add | dysph | dysph+temper | 0.64 | 0.42 | 0.86 | **0.0000** | 8577 | 10 | 31 | 3 | 0.12 | 8.82 |
| dl | Add | temper | temper+dysph | 0.51 | 0.33 | 0.68 | **0.0000** | 8577 | 10 | 311 | 7 | 0.12 | 2.20 |
| dh | Add | dysph | dysph+fights | 0.58 | 0.21 | 0.94 | **0.0019** | 8867 | 2 | 61 | 1 | 0.02 | 1.61 |
| dl | Add | fights | fights+dysph | 0.63 | 0.28 | 0.98 | **0.0004** | 8867 | 2 | 35 | 1 | 0.02 | 2.78 |
| dh | Add | dysph | dysph+steals | 0.58 | 0.21 | 0.94 | **0.0019** | 8864 | 2 | 61 | 1 | 0.02 | 1.61 |
| dl | Add | steals | steals+dysph | . | . | . | . | 8864 | 2 | 39 | 0 | 0.02 | 0.00 |
| dh | Add | worth | worth+attend | . | . | . | . | 7827 | 14 | 38 | 0 | 0.18 | 0.00 |
| dl | Add | attend | attend+worth | 0.51 | 0.40 | 0.62 | **0.0000** | 7827 | 14 | 1022 | 25 | 0.18 | 2.39 |
| dh | Add | worth | worth+hyper | 0.52 | 0.25 | 0.78 | **0.0002** | 8264 | 8 | 59 | 2 | 0.10 | 3.28 |
| dl | Add | hyper | hyper+worth | 0.46 | 0.29 | 0.64 | **0.0000** | 8264 | 8 | 608 | 8 | 0.10 | 1.30 |
| dh | Add | worth | worth+argues | . | . | . | . | 8127 | 16 | 44 | 0 | 0.20 | 0.00 |
| dl | Add | argues | argues+worth | 0.47 | 0.35 | 0.60 | **0.0000** | 8127 | 16 | 728 | 17 | 0.20 | 2.28 |
| dh | Add | worth | worth+temper | . | . | . | . | 8552 | 17 | 52 | 0 | 0.20 | 0.00 |
| dl | Add | temper | temper+worth | 0.42 | 0.25 | 0.59 | **0.0000** | 8552 | 17 | 312 | 7 | 0.20 | 2.19 |
| dh | Add | worth | worth+fights | . | . | . | . | 8851 | 0 | 79 | 1 | 0.00 | 1.25 |
| dl | Add | fights | fights+worth | . | . | . | . | 8851 | 0 | 37 | 0 | 0.00 | 0.00 |
| dh | Add | worth | worth+steals | 0.50 | 0.13 | 0.86 | **0.0077** | 8845 | 3 | 79 | 1 | 0.03 | 1.25 |
| dl | Add | steals | steals+worth | . | . | . | . | 8845 | 3 | 40 | 0 | 0.03 | 0.00 |
| dh | Add | attend | attend+hyper | 0.46 | 0.38 | 0.54 | **0.0000** | 7633 | 85 | 566 | 50 | 1.10 | 8.12 |
| dl | Add | hyper | hyper+attend | 0.56 | 0.46 | 0.66 | **0.0000** | 7633 | 85 | 136 | 26 | 1.10 | 16.05 |
| dh | Add | attend | attend+argues | 0.49 | 0.42 | 0.56 | **0.0000** | 7339 | 81 | 702 | 66 | 1.09 | 8.59 |
| dl | Add | argues | argues+attend | 0.50 | 0.42 | 0.59 | **0.0000** | 7339 | 81 | 414 | 46 | 1.09 | 10.00 |
| dh | Add | attend | attend+temper | 0.49 | 0.40 | 0.58 | **0.0000** | 7663 | 36 | 881 | 41 | 0.47 | 4.45 |
| dl | Add | temper | temper+attend | 0.50 | 0.36 | 0.63 | **0.0000** | 7663 | 36 | 168 | 12 | 0.47 | 6.67 |
| dh | Add | attend | attend+fights | 0.34 | 0.15 | 0.53 | **0.0004** | 7857 | 8 | 1059 | 7 | 0.10 | 0.66 |
| dl | Add | fights | fights+attend | 0.57 | 0.22 | 0.92 | **0.0013** | 7857 | 8 | 13 | 1 | 0.10 | 7.14 |
| dh | Add | attend | attend+steals | 0.41 | 0.27 | 0.56 | **0.0000** | 7853 | 11 | 1051 | 13 | 0.14 | 1.22 |
| dl | Add | steals | steals+attend | 0.65 | 0.40 | 0.91 | **0.0000** | 7853 | 11 | 13 | 2 | 0.14 | 13.33 |
| dh | Add | hyper | hyper+argues | 0.56 | 0.47 | 0.65 | **0.0000** | 7726 | 43 | 385 | 34 | 0.55 | 8.11 |
| dl | Add | argues | argues+hyper | 0.38 | 0.27 | 0.50 | **0.0000** | 7726 | 43 | 545 | 20 | 0.55 | 3.54 |
| dh | Add | hyper | hyper+temper | 0.57 | 0.47 | 0.68 | **0.0000** | 8085 | 20 | 492 | 24 | 0.25 | 4.65 |
| dl | Add | temper | temper+hyper | 0.47 | 0.31 | 0.63 | **0.0000** | 8085 | 20 | 220 | 8 | 0.25 | 3.51 |
| dh | Add | hyper | hyper+fights | 0.53 | 0.36 | 0.71 | **0.0000** | 8304 | 5 | 614 | 8 | 0.06 | 1.29 |
| dl | Add | fights | fights+hyper | 0.56 | 0.22 | 0.91 | **0.0015** | 8304 | 5 | 23 | 1 | 0.06 | 4.17 |
| dh | Add | hyper | hyper+steals | 0.48 | 0.28 | 0.68 | **0.0000** | 8304 | 5 | 613 | 6 | 0.06 | 0.97 |
| dl | Add | steals | steals+hyper | 0.56 | 0.22 | 0.91 | **0.0015** | 8304 | 5 | 23 | 1 | 0.06 | 4.17 |
| dh | Add | argues | argues+temper | 0.49 | 0.40 | 0.57 | **0.0000** | 7983 | 68 | 527 | 43 | 0.84 | 7.54 |
| dl | Add | temper | temper+argues | 0.43 | 0.29 | 0.56 | **0.0000** | 7983 | 68 | 125 | 11 | 0.84 | 8.09 |
| dh | Add | argues | argues+fights | 0.55 | 0.40 | 0.70 | **0.0000** | 8170 | 6 | 743 | 12 | 0.07 | 1.59 |
| dl | Add | fights | fights+argues | . | . | . | . | 8170 | 6 | 11 | 0 | 0.07 | 0.00 |
| dh | Add | argues | argues+steals | 0.44 | 0.28 | 0.60 | **0.0000** | 8155 | 10 | 753 | 10 | 0.12 | 1.31 |
| dl | Add | steals | steals+argues | 0.69 | 0.48 | 0.90 | **0.0000** | 8155 | 10 | 19 | 3 | 0.12 | 13.64 |
| dh | Add | temper | temper+fights | 0.49 | 0.29 | 0.69 | **0.0000** | 8597 | 7 | 322 | 5 | 0.08 | 1.53 |
| dl | Add | fights | fights+temper | 0.57 | 0.22 | 0.91 | **0.0013** | 8597 | 7 | 16 | 1 | 0.08 | 5.88 |
| dh | Add | temper | temper+steals | 0.51 | 0.31 | 0.71 | **0.0000** | 8583 | 6 | 334 | 5 | 0.07 | 1.47 |
| dl | Add | steals | steals+temper | 0.70 | 0.50 | 0.91 | **0.0000** | 8583 | 6 | 29 | 3 | 0.07 | 9.38 |
| dh | Add | fights | fights+steals | 0.63 | 0.28 | 0.98 | **0.0004** | 8889 | 2 | 36 | 1 | 0.02 | 2.70 |
| dl | Add | steals | steals+fights | 0.73 | 0.49 | 0.98 | **0.0000** | 8889 | 2 | 38 | 2 | 0.02 | 5.00 |
| dp | Joint Persist | fear+worry | fear+worry | 0.75 | 0.68 | 0.81 | **0.0000** | 8416 | 74 | 93 | 42 | 0.87 | 31.11 |
| dp | Joint Persist | fear+dysph | fear+dysph | 0.71 | 0.55 | 0.86 | **0.0000** | 8682 | 26 | 19 | 6 | 0.30 | 24.00 |
| dp | Joint Persist | fear+worth | fear+worth | 0.65 | 0.47 | 0.82 | **0.0000** | 8675 | 23 | 27 | 5 | 0.26 | 15.63 |
| dp | Joint Persist | fear+attend | fear+attend | 0.78 | 0.69 | 0.86 | **0.0000** | 7738 | 24 | 82 | 23 | 0.31 | 21.90 |
| dp | Joint Persist | fear+hyper | fear+hyper | 0.84 | 0.76 | 0.93 | **0.0000** | 8179 | 8 | 61 | 15 | 0.10 | 19.74 |
| dp | Joint Persist | fear+argues | fear+argues | 0.80 | 0.71 | 0.88 | **0.0000** | 8024 | 22 | 61 | 20 | 0.27 | 24.69 |
| dp | Joint Persist | fear+temper | fear+temper | 0.72 | 0.58 | 0.86 | **0.0000** | 8447 | 13 | 53 | 8 | 0.15 | 13.11 |
| dp | Joint Persist | fear+fights | fear+fights | . | . | . | . | 8716 | 0 | 6 | 1 | 0.00 | 14.29 |
| dp | Joint Persist | fear+steals | fear+steals | . | . | . | . | 8707 | 3 | 4 | 0 | 0.03 | 0.00 |
| dp | Joint Persist | worry+dysph | worry+dysph | 0.62 | 0.44 | 0.79 | **0.0000** | 8518 | 27 | 29 | 5 | 0.32 | 14.71 |
| dp | Joint Persist | worry+worth | worry+worth | 0.63 | 0.46 | 0.79 | **0.0000** | 8513 | 25 | 38 | 6 | 0.29 | 13.64 |
| dp | Joint Persist | worry+attend | worry+attend | 0.81 | 0.75 | 0.87 | **0.0000** | 7610 | 28 | 114 | 39 | 0.37 | 25.49 |
| dp | Joint Persist | worry+hyper | worry+hyper | 0.83 | 0.76 | 0.91 | **0.0000** | 8021 | 12 | 74 | 20 | 0.15 | 21.28 |
| dp | Joint Persist | worry+argues | worry+argues | 0.78 | 0.71 | 0.86 | **0.0000** | 7881 | 29 | 89 | 28 | 0.37 | 23.93 |
| dp | Joint Persist | worry+temper | worry+temper | 0.74 | 0.63 | 0.84 | **0.0000** | 8281 | 22 | 62 | 14 | 0.26 | 18.42 |
| dp | Joint Persist | worry+fights | worry+fights | 0.81 | 0.53 | 1.00 | **0.0000** | 8546 | 1 | 9 | 1 | 0.01 | 10.00 |
| dp | Joint Persist | worry+steals | worry+steals | . | . | . | . | 8539 | 1 | 6 | 0 | 0.01 | 0.00 |
| dp | Joint Persist | dysph+worth | dysph+worth | 0.80 | 0.67 | 0.92 | **0.0000** | 8816 | 24 | 11 | 7 | 0.27 | 38.89 |
| dp | Joint Persist | dysph+attend | dysph+attend | 0.72 | 0.57 | 0.87 | **0.0000** | 7835 | 14 | 30 | 6 | 0.18 | 16.67 |
| dp | Joint Persist | dysph+hyper | dysph+hyper | 0.84 | 0.70 | 0.99 | **0.0000** | 8282 | 4 | 15 | 4 | 0.05 | 21.05 |
| dp | Joint Persist | dysph+argues | dysph+argues | 0.72 | 0.57 | 0.86 | **0.0000** | 8141 | 18 | 31 | 7 | 0.22 | 18.42 |
| dp | Joint Persist | dysph+temper | dysph+temper | 0.82 | 0.71 | 0.94 | **0.0000** | 8577 | 10 | 24 | 8 | 0.12 | 25.00 |
| dp | Joint Persist | dysph+fights | dysph+fights | 0.84 | 0.58 | 1.00 | **0.0000** | 8867 | 2 | 3 | 1 | 0.02 | 25.00 |
| dp | Joint Persist | dysph+steals | dysph+steals | 0.84 | 0.58 | 1.00 | **0.0000** | 8864 | 2 | 3 | 1 | 0.02 | 25.00 |
| dp | Joint Persist | worth+attend | worth+attend | 0.65 | 0.48 | 0.83 | **0.0000** | 7827 | 14 | 40 | 5 | 0.18 | 11.11 |
| dp | Joint Persist | worth+hyper | worth+hyper | 0.64 | 0.39 | 0.90 | **0.0000** | 8264 | 8 | 20 | 2 | 0.10 | 9.09 |
| dp | Joint Persist | worth+argues | worth+argues | 0.66 | 0.49 | 0.83 | **0.0000** | 8127 | 16 | 34 | 5 | 0.20 | 12.82 |
| dp | Joint Persist | worth+temper | worth+temper | 0.75 | 0.62 | 0.89 | **0.0000** | 8552 | 17 | 24 | 7 | 0.20 | 22.58 |
| dp | Joint Persist | worth+fights | worth+fights | . | . | . | . | 8851 | 0 | 2 | 1 | 0.00 | 33.33 |
| dp | Joint Persist | worth+steals | worth+steals | . | . | . | . | 8845 | 3 | 3 | 0 | 0.03 | 0.00 |
| dp | Joint Persist | attend+hyper | attend+hyper | 0.84 | 0.81 | 0.87 | **0.0000** | 7633 | 85 | 293 | 183 | 1.10 | 38.45 |
| dp | Joint Persist | attend+argues | attend+argues | 0.80 | 0.76 | 0.85 | **0.0000** | 7339 | 81 | 211 | 113 | 1.09 | 34.88 |
| dp | Joint Persist | attend+temper | attend+temper | 0.69 | 0.60 | 0.78 | **0.0000** | 7663 | 36 | 143 | 27 | 0.47 | 15.88 |
| dp | Joint Persist | attend+fights | attend+fights | 0.78 | 0.63 | 0.94 | **0.0000** | 7857 | 8 | 21 | 5 | 0.10 | 19.23 |
| dp | Joint Persist | attend+steals | attend+steals | 0.78 | 0.63 | 0.92 | **0.0000** | 7853 | 11 | 22 | 6 | 0.14 | 21.43 |
| dp | Joint Persist | hyper+argues | hyper+argues | 0.83 | 0.79 | 0.88 | **0.0000** | 7726 | 43 | 148 | 71 | 0.55 | 32.42 |
| dp | Joint Persist | hyper+temper | hyper+temper | 0.73 | 0.63 | 0.83 | **0.0000** | 8085 | 20 | 104 | 18 | 0.25 | 14.75 |
| dp | Joint Persist | hyper+fights | hyper+fights | 0.84 | 0.70 | 0.99 | **0.0000** | 8304 | 5 | 12 | 4 | 0.06 | 25.00 |
| dp | Joint Persist | hyper+steals | hyper+steals | 0.78 | 0.59 | 0.97 | **0.0000** | 8304 | 5 | 16 | 3 | 0.06 | 15.79 |
| dp | Joint Persist | argues+temper | argues+temper | 0.72 | 0.65 | 0.78 | **0.0000** | 7983 | 68 | 163 | 51 | 0.84 | 23.83 |
| dp | Joint Persist | argues+fights | argues+fights | 0.76 | 0.59 | 0.94 | **0.0000** | 8170 | 6 | 25 | 4 | 0.07 | 13.79 |
| dp | Joint Persist | argues+steals | argues+steals | 0.62 | 0.36 | 0.88 | **0.0000** | 8155 | 10 | 19 | 2 | 0.12 | 9.52 |
| dp | Joint Persist | temper+fights | temper+fights | 0.78 | 0.61 | 0.94 | **0.0000** | 8597 | 7 | 19 | 4 | 0.08 | 17.39 |
| dp | Joint Persist | temper+steals | temper+steals | 0.63 | 0.30 | 0.97 | **0.0002** | 8583 | 6 | 10 | 1 | 0.07 | 9.09 |
| dp | Joint Persist | fights+steals | fights+steals | 0.87 | 0.63 | 1.00 | **0.0000** | 8889 | 2 | 2 | 1 | 0.02 | 33.33 |
| cg | Shift | fear | Worry | 0.23 | 0.06 | 0.39 | **0.0067** | 8324 | 166 | 81 | 6 | 1.96 | 6.90 |
| bj | Shift | worry | Fear | 0.15 | -.02 | 0.33 | 0.0761 | 8423 | 67 | 254 | 5 | 0.79 | 1.93 |
| cg | Shift | fear | Dysph | 0.08 | -.14 | 0.31 | 0.4697 | 8657 | 51 | 195 | 2 | 0.59 | 1.02 |
| bj | Shift | dysph | Fear | 0.18 | -.06 | 0.42 | 0.1476 | 8573 | 135 | 39 | 2 | 1.55 | 4.88 |
| cg | Shift | fear | Worth | 0.18 | -.03 | 0.39 | 0.0859 | 8654 | 44 | 187 | 3 | 0.51 | 1.58 |
| bj | Shift | worth | Fear | 0.21 | 0.00 | 0.43 | 0.0462 | 8560 | 138 | 48 | 3 | 1.59 | 5.88 |
| cg | Shift | fear | Attend | 0.11 | -.03 | 0.25 | 0.1108 | 7421 | 341 | 108 | 9 | 4.39 | 7.69 |
| bj | Shift | attend | Fear | 0.03 | -.08 | 0.15 | 0.5783 | 7661 | 101 | 972 | 15 | 1.30 | 1.52 |
| cg | Shift | fear | Hyper | 0.06 | -.12 | 0.23 | 0.5314 | 8025 | 162 | 142 | 4 | 1.98 | 2.74 |
| bj | Shift | hyper | Fear | -.05 | -.18 | 0.09 | 0.4756 | 8054 | 133 | 555 | 7 | 1.62 | 1.25 |
| cg | Shift | fear | argues | -.14 | -.31 | 0.03 | 0.1137 | 7663 | 383 | 138 | 3 | 4.76 | 2.13 |
| bj | Shift | argues | Fear | 0.06 | -.06 | 0.17 | 0.3580 | 7933 | 113 | 690 | 13 | 1.40 | 1.85 |
| cg | Shift | fear | temper | 0.11 | -.05 | 0.28 | 0.1832 | 8322 | 138 | 156 | 5 | 1.63 | 3.11 |
| bj | Shift | temper | Fear | 0.12 | -.01 | 0.26 | 0.0797 | 8321 | 139 | 280 | 9 | 1.64 | 3.11 |
| cg | Shift | fear | Fights | . | . | . | . | 8693 | 23 | 215 | 0 | 0.26 | 0.00 |
| bj | Shift | fights | Fear | 0.07 | -.23 | 0.37 | 0.6482 | 8552 | 164 | 32 | 1 | 1.88 | 3.03 |
| cg | Shift | fear | Steals | 0.14 | -.10 | 0.38 | 0.2496 | 8678 | 32 | 216 | 2 | 0.37 | 0.92 |
| bj | Shift | steals | Fear | 0.16 | -.08 | 0.40 | 0.1890 | 8550 | 160 | 37 | 2 | 1.84 | 5.13 |
| cg | Shift | worry | Dysph | 0.18 | 0.01 | 0.36 | **0.0409** | 8504 | 41 | 355 | 5 | 0.48 | 1.39 |
| bj | Shift | dysph | Worry | 0.03 | -.26 | 0.32 | 0.8548 | 8323 | 222 | 31 | 1 | 2.60 | 3.13 |
| cg | Shift | worry | Worth | 0.15 | -.04 | 0.34 | 0.1171 | 8498 | 40 | 346 | 4 | 0.47 | 1.14 |
| bj | Shift | worth | Worry | 0.29 | 0.11 | 0.47 | **0.0019** | 8317 | 221 | 34 | 5 | 2.59 | 12.82 |
| cg | Shift | worry | Attend | 0.08 | -.03 | 0.19 | 0.1651 | 7311 | 327 | 226 | 15 | 4.28 | 6.22 |
| bj | Shift | attend | Worry | -.11 | -.22 | -.00 | 0.0462 | 7469 | 169 | 927 | 12 | 2.21 | 1.28 |
| cg | Shift | worry | Hyper | 0.09 | -.05 | 0.22 | 0.1972 | 7881 | 152 | 291 | 9 | 1.89 | 3.00 |
| bj | Shift | hyper | Worry | -.05 | -.16 | 0.07 | 0.4329 | 7828 | 205 | 533 | 11 | 2.55 | 2.02 |
| cg | Shift | worry | argues | 0.00 | -.11 | 0.12 | 0.9512 | 7545 | 365 | 264 | 13 | 4.61 | 4.69 |
| bj | Shift | argues | Worry | 0.01 | -.10 | 0.12 | 0.8998 | 7738 | 172 | 652 | 15 | 2.17 | 2.25 |
| cg | Shift | worry | temper | 0.10 | -.04 | 0.24 | 0.1732 | 8179 | 124 | 310 | 8 | 1.49 | 2.52 |
| bj | Shift | temper | Worry | 0.15 | 0.03 | 0.27 | **0.0118** | 8101 | 202 | 260 | 14 | 2.43 | 5.11 |
| cg | Shift | worry | Fights | 0.00 | -.29 | 0.29 | 0.9909 | 8525 | 22 | 383 | 1 | 0.26 | 0.26 |
| bj | Shift | fights | Worry | 0.02 | -.27 | 0.31 | 0.8993 | 8296 | 251 | 29 | 1 | 2.94 | 3.33 |
| cg | Shift | worry | Steals | 0.18 | -.02 | 0.37 | 0.0727 | 8509 | 31 | 384 | 4 | 0.36 | 1.03 |
| bj | Shift | steals | Worry | 0.28 | 0.10 | 0.47 | **0.0025** | 8295 | 245 | 32 | 5 | 2.87 | 13.51 |
| cg | Shift | dysph | Worth | 0.37 | 0.15 | 0.59 | **0.0008** | 8789 | 51 | 45 | 3 | 0.58 | 6.25 |
| bj | Shift | worth | Dysph | 0.12 | -.18 | 0.42 | 0.4443 | 8781 | 59 | 64 | 1 | 0.67 | 1.54 |
| cg | Shift | dysph | Attend | 0.25 | 0.06 | 0.44 | **0.0092** | 7489 | 360 | 25 | 5 | 4.59 | 16.67 |
| bj | Shift | attend | Dysph | -.04 | -.20 | 0.12 | 0.6419 | 7803 | 46 | 1051 | 5 | 0.59 | 0.47 |
| cg | Shift | dysph | Hyper | 0.11 | -.12 | 0.35 | 0.3442 | 8114 | 172 | 45 | 2 | 2.08 | 4.26 |
| bj | Shift | hyper | Dysph | 0.05 | -.11 | 0.20 | 0.5543 | 8224 | 62 | 613 | 6 | 0.75 | 0.97 |
| cg | Shift | dysph | argues | -.04 | -.33 | 0.24 | 0.7577 | 7769 | 390 | 27 | 1 | 4.78 | 3.57 |
| bj | Shift | argues | Dysph | -.02 | -.20 | 0.15 | 0.7935 | 8109 | 50 | 742 | 4 | 0.61 | 0.54 |
| cg | Shift | dysph | temper | 0.20 | -.05 | 0.44 | 0.1193 | 8441 | 146 | 32 | 2 | 1.70 | 5.88 |
| bj | Shift | temper | Dysph | 0.17 | 0.01 | 0.33 | 0.0426 | 8524 | 63 | 312 | 6 | 0.73 | 1.89 |
| cg | Shift | dysph | Fights | . | . | . | . | 8847 | 22 | 62 | 0 | 0.25 | 0.00 |
| bj | Shift | fights | Dysph | . | . | . | . | 8786 | 83 | 36 | 0 | 0.94 | 0.00 |
| cg | Shift | dysph | Steals | . | . | . | . | 8831 | 35 | 62 | 0 | 0.39 | 0.00 |
| bj | Shift | steals | Dysph | . | . | . | . | 8782 | 84 | 39 | 0 | 0.95 | 0.00 |
| cg | Shift | worth | Attend | 0.20 | 0.02 | 0.38 | **0.0314** | 7480 | 361 | 33 | 5 | 4.60 | 13.16 |
| bj | Shift | attend | Worth | -.04 | -.21 | 0.14 | 0.6807 | 7804 | 37 | 1043 | 4 | 0.47 | 0.38 |
| cg | Shift | worth | Hyper | 0.25 | 0.07 | 0.43 | **0.0059** | 8107 | 165 | 56 | 5 | 1.99 | 8.20 |
| bj | Shift | hyper | Worth | 0.06 | -.10 | 0.22 | 0.4572 | 8214 | 58 | 610 | 6 | 0.70 | 0.97 |
| cg | Shift | worth | argues | 0.16 | -.01 | 0.34 | 0.0718 | 7753 | 390 | 39 | 5 | 4.79 | 11.36 |
| bj | Shift | argues | Worth | -.12 | -.32 | 0.09 | 0.2744 | 8098 | 45 | 743 | 2 | 0.55 | 0.27 |
| cg | Shift | worth | temper | 0.13 | -.10 | 0.37 | 0.2742 | 8427 | 142 | 50 | 2 | 1.66 | 3.85 |
| bj | Shift | temper | Worth | 0.21 | 0.04 | 0.37 | **0.0143** | 8519 | 50 | 313 | 6 | 0.58 | 1.88 |
| cg | Shift | worth | Fights | 0.22 | -.10 | 0.54 | 0.1805 | 8828 | 23 | 79 | 1 | 0.26 | 1.25 |
| bj | Shift | fights | Worth | 0.16 | -.15 | 0.46 | 0.3204 | 8772 | 79 | 36 | 1 | 0.89 | 2.70 |
| cg | Shift | worth | Steals | 0.17 | -.14 | 0.48 | 0.2865 | 8815 | 33 | 79 | 1 | 0.37 | 1.25 |
| bj | Shift | steals | Worth | 0.35 | 0.13 | 0.57 | **0.0018** | 8774 | 74 | 37 | 3 | 0.84 | 7.50 |
| cg | Shift | attend | Hyper | 0.25 | 0.10 | 0.39 | **0.0010** | 7683 | 35 | 606 | 10 | 0.45 | 1.62 |
| bj | Shift | hyper | Attend | -.06 | -.22 | 0.11 | 0.4876 | 7453 | 265 | 158 | 4 | 3.43 | 2.47 |
| cg | Shift | attend | argues | -.04 | -.13 | 0.05 | 0.4097 | 7177 | 243 | 747 | 21 | 3.27 | 2.73 |
| bj | Shift | argues | Attend | 0.08 | -.02 | 0.19 | 0.1053 | 7188 | 232 | 439 | 21 | 3.13 | 4.57 |
| cg | Shift | attend | temper | 0.10 | -.02 | 0.23 | 0.0924 | 7629 | 70 | 908 | 14 | 0.91 | 1.52 |
| bj | Shift | temper | Attend | 0.20 | 0.09 | 0.31 | **0.0005** | 7385 | 314 | 162 | 18 | 4.08 | 10.00 |
| cg | Shift | attend | Fights | 0.28 | 0.05 | 0.51 | **0.0179** | 7859 | 6 | 1062 | 4 | 0.08 | 0.38 |
| bj | Shift | fights | Attend | 0.07 | -.25 | 0.38 | 0.6828 | 7495 | 370 | 13 | 1 | 4.70 | 7.14 |
| cg | Shift | attend | Steals | 0.19 | -.03 | 0.41 | 0.0838 | 7854 | 10 | 1060 | 4 | 0.13 | 0.38 |
| bj | Shift | steals | Attend | 0.34 | 0.13 | 0.56 | **0.0019** | 7501 | 363 | 11 | 4 | 4.62 | 26.67 |
| cg | Shift | hyper | argues | 0.06 | -.04 | 0.16 | 0.2289 | 7457 | 312 | 397 | 22 | 4.02 | 5.25 |
| bj | Shift | argues | Hyper | 0.05 | -.08 | 0.18 | 0.4573 | 7662 | 107 | 555 | 10 | 1.38 | 1.77 |
| cg | Shift | hyper | temper | 0.10 | -.03 | 0.22 | 0.1398 | 7999 | 106 | 505 | 11 | 1.31 | 2.13 |
| bj | Shift | temper | Hyper | 0.26 | 0.14 | 0.38 | **0.0000** | 7967 | 138 | 214 | 14 | 1.70 | 6.14 |
| cg | Shift | hyper | Fights | 0.25 | 0.01 | 0.49 | **0.0401** | 8300 | 9 | 619 | 3 | 0.11 | 0.48 |
| bj | Shift | fights | Hyper | . | . | . | . | 8135 | 174 | 24 | 0 | 2.09 | 0.00 |
| cg | Shift | hyper | Steals | 0.15 | -.05 | 0.34 | 0.1447 | 8286 | 23 | 615 | 4 | 0.28 | 0.65 |
| bj | Shift | steals | Hyper | . | . | . | . | 8135 | 174 | 24 | 0 | 2.09 | 0.00 |
| cg | Shift | argues | temper | -.02 | -.21 | 0.17 | 0.8599 | 8004 | 47 | 567 | 3 | 0.58 | 0.53 |
| bj | Shift | temper | argues | 0.20 | 0.08 | 0.32 | **0.0011** | 7733 | 318 | 122 | 14 | 3.95 | 10.29 |
| cg | Shift | argues | Fights | 0.09 | -.25 | 0.43 | 0.6031 | 8170 | 6 | 754 | 1 | 0.07 | 0.13 |
| bj | Shift | fights | argues | 0.10 | -.23 | 0.42 | 0.5577 | 7772 | 404 | 10 | 1 | 4.94 | 9.09 |
| cg | Shift | argues | Steals | 0.05 | -.20 | 0.29 | 0.7057 | 8149 | 16 | 761 | 2 | 0.20 | 0.26 |
| bj | Shift | steals | argues | 0.19 | -.03 | 0.41 | 0.0979 | 7770 | 395 | 19 | 3 | 4.84 | 13.64 |
| cg | Shift | temper | Fights | 0.25 | -.02 | 0.51 | 0.0702 | 8593 | 11 | 325 | 2 | 0.13 | 0.61 |
| bj | Shift | fights | temper | 0.39 | 0.16 | 0.62 | **0.0008** | 8454 | 150 | 14 | 3 | 1.74 | 17.65 |
| cg | Shift | temper | Steals | 0.25 | 0.05 | 0.45 | **0.0145** | 8566 | 23 | 335 | 4 | 0.27 | 1.18 |
| bj | Shift | Steals | temper | 0.28 | 0.06 | 0.50 | **0.0123** | 8440 | 149 | 29 | 3 | 1.73 | 9.38 |
| cg | Shift | Fights | Steals | . | . | . | . | 8856 | 35 | 37 | 0 | 0.39 | 0.00 |
| bj | Shift | Steals | Fights | 0.33 | -.00 | 0.66 | 0.0519 | 8871 | 20 | 39 | 1 | 0.22 | 2.50 |
| co | Subtract x | fear+worry | Worry | 0.30 | 0.17 | 0.43 | **0.0000** | 8324 | 166 | 123 | 12 | 1.96 | 8.89 |
| co | Subtract x | fear+dysph | Dysph | 0.39 | 0.13 | 0.65 | **0.0029** | 8657 | 51 | 23 | 2 | 0.59 | 8.00 |
| co | Subtract x | fear+worth | Worth | 0.25 | -.07 | 0.58 | 0.1241 | 8654 | 44 | 31 | 1 | 0.51 | 3.13 |
| co | Subtract x | fear+attend | Attend | 0.56 | 0.47 | 0.64 | **0.0000** | 7421 | 341 | 64 | 41 | 4.39 | 39.05 |
| co | Subtract x | fear+hyper | Hyper | 0.60 | 0.50 | 0.70 | **0.0000** | 8025 | 162 | 52 | 24 | 1.98 | 31.58 |
| co | Subtract x | fear+argues | argues | 0.45 | 0.34 | 0.55 | **0.0000** | 7663 | 383 | 56 | 25 | 4.76 | 30.86 |
| co | Subtract x | fear+temper | temper | 0.42 | 0.27 | 0.56 | **0.0000** | 8322 | 138 | 52 | 9 | 1.63 | 14.75 |
| co | Subtract x | fear+fights | Fights | . | . | . | . | 8693 | 23 | 7 | 0 | 0.26 | 0.00 |
| co | Subtract x | fear+steals | Steals | 0.59 | 0.24 | 0.94 | **0.0009** | 8678 | 32 | 3 | 1 | 0.37 | 25.00 |
| co | Subtract x | worry+dysph | Dysph | 0.56 | 0.38 | 0.74 | **0.0000** | 8504 | 41 | 29 | 5 | 0.48 | 14.71 |
| co | Subtract x | worry+worth | Worth | 0.34 | 0.09 | 0.60 | **0.0089** | 8498 | 40 | 42 | 2 | 0.47 | 4.55 |
| co | Subtract x | worry+attend | Attend | 0.47 | 0.38 | 0.55 | **0.0000** | 7311 | 327 | 111 | 42 | 4.28 | 27.45 |
| co | Subtract x | worry+hyper | Hyper | 0.52 | 0.42 | 0.63 | **0.0000** | 7881 | 152 | 73 | 21 | 1.89 | 22.34 |
| co | Subtract x | worry+argues | argues | 0.41 | 0.32 | 0.51 | **0.0000** | 7545 | 365 | 87 | 30 | 4.61 | 25.64 |
| co | Subtract x | worry+temper | temper | 0.36 | 0.21 | 0.51 | **0.0000** | 8179 | 124 | 68 | 8 | 1.49 | 10.53 |
| co | Subtract x | worry+fights | Fights | 0.72 | 0.51 | 0.92 | **0.0000** | 8525 | 22 | 7 | 3 | 0.26 | 30.00 |
| co | Subtract x | worry+steals | Steals | 0.77 | 0.57 | 0.96 | **0.0000** | 8509 | 31 | 3 | 3 | 0.36 | 50.00 |
| co | Subtract x | dysph+worth | Worth | . | . | . | . | 8789 | 51 | 18 | 0 | 0.58 | 0.00 |
| co | Subtract x | dysph+attend | Attend | 0.49 | 0.35 | 0.62 | **0.0000** | 7489 | 360 | 22 | 14 | 4.59 | 38.89 |
| co | Subtract x | dysph+hyper | Hyper | 0.47 | 0.28 | 0.66 | **0.0000** | 8114 | 172 | 14 | 5 | 2.08 | 26.32 |
| co | Subtract x | dysph+argues | argues | 0.51 | 0.38 | 0.63 | **0.0000** | 7769 | 390 | 22 | 16 | 4.78 | 42.11 |
| co | Subtract x | dysph+temper | temper | 0.44 | 0.26 | 0.61 | **0.0000** | 8441 | 146 | 26 | 6 | 1.70 | 18.75 |
| co | Subtract x | dysph+fights | Fights | 0.63 | 0.29 | 0.98 | **0.0003** | 8847 | 22 | 3 | 1 | 0.25 | 25.00 |
| co | Subtract x | dysph+steals | Steals | 0.58 | 0.23 | 0.94 | **0.0012** | 8831 | 35 | 3 | 1 | 0.39 | 25.00 |
| co | Subtract x | worth+attend | Attend | 0.57 | 0.47 | 0.68 | **0.0000** | 7480 | 361 | 23 | 22 | 4.60 | 48.89 |
| co | Subtract x | worth+hyper | Hyper | 0.49 | 0.31 | 0.67 | **0.0000** | 8107 | 165 | 16 | 6 | 1.99 | 27.27 |
| co | Subtract x | worth+argues | argues | 0.36 | 0.21 | 0.51 | **0.0000** | 7753 | 390 | 29 | 10 | 4.79 | 25.64 |
| co | Subtract x | worth+temper | temper | 0.29 | 0.07 | 0.51 | **0.0092** | 8427 | 142 | 28 | 3 | 1.66 | 9.68 |
| co | Subtract x | worth+fights | Fights | . | . | . | . | 8828 | 23 | 3 | 0 | 0.26 | 0.00 |
| co | Subtract x | worth+steals | Steals | 0.64 | 0.29 | 0.99 | **0.0003** | 8815 | 33 | 2 | 1 | 0.37 | 33.33 |
| co | Subtract x | attend+hyper | Hyper | 0.41 | 0.29 | 0.53 | **0.0000** | 7683 | 35 | 459 | 17 | 0.45 | 3.57 |
| co | Subtract x | attend+argues | argues | 0.40 | 0.33 | 0.48 | **0.0000** | 7177 | 243 | 271 | 53 | 3.27 | 16.36 |
| co | Subtract x | attend+temper | temper | 0.33 | 0.18 | 0.48 | **0.0000** | 7629 | 70 | 161 | 9 | 0.91 | 5.29 |
| co | Subtract x | attend+fights | Fights | . | . | . | . | 7859 | 6 | 26 | 0 | 0.08 | 0.00 |
| co | Subtract x | attend+steals | Steals | 0.46 | 0.11 | 0.80 | **0.0104** | 7854 | 10 | 27 | 1 | 0.13 | 3.57 |
| co | Subtract x | hyper+argues | argues | 0.45 | 0.37 | 0.53 | **0.0000** | 7457 | 312 | 168 | 51 | 4.02 | 23.29 |
| co | Subtract x | hyper+temper | temper | 0.30 | 0.15 | 0.45 | **0.0001** | 7999 | 106 | 114 | 8 | 1.31 | 6.56 |
| co | Subtract x | hyper+fights | Fights | . | . | . | . | 8300 | 9 | 16 | 0 | 0.11 | 0.00 |
| co | Subtract x | hyper+steals | Steals | 0.53 | 0.27 | 0.80 | **0.0001** | 8286 | 23 | 17 | 2 | 0.28 | 10.53 |
| co | Subtract x | argues+temper | temper | 0.24 | 0.06 | 0.42 | **0.0088** | 8004 | 47 | 209 | 5 | 0.58 | 2.34 |
| co | Subtract x | argues+fights | Fights | 0.52 | 0.17 | 0.87 | **0.0038** | 8170 | 6 | 28 | 1 | 0.07 | 3.45 |
| co | Subtract x | argues+steals | Steals | 0.56 | 0.30 | 0.83 | **0.0000** | 8149 | 16 | 19 | 2 | 0.20 | 9.52 |
| co | Subtract x | temper+fights | Fights | 0.60 | 0.34 | 0.86 | **0.0000** | 8593 | 11 | 21 | 2 | 0.13 | 8.70 |
| co | Subtract x | temper+steals | Steals | 0.61 | 0.36 | 0.87 | **0.0000** | 8566 | 23 | 9 | 2 | 0.27 | 18.18 |
| co | Subtract x | fights+steals | Steals | . | . | . | . | 8856 | 35 | 3 | 0 | 0.39 | 0.00 |
| bn | Subtract y | worry+fear | Fear | 0.43 | 0.30 | 0.57 | **0.0000** | 8423 | 67 | 124 | 11 | 0.79 | 8.15 |
| bn | Subtract y | dysph+fear | Fear | 0.53 | 0.37 | 0.70 | **0.0000** | 8573 | 135 | 18 | 7 | 1.55 | 28.00 |
| bn | Subtract y | worth+fear | Fear | 0.45 | 0.27 | 0.62 | **0.0000** | 8560 | 138 | 26 | 6 | 1.59 | 18.75 |
| bn | Subtract y | attend+fear | Fear | 0.30 | 0.14 | 0.46 | **0.0003** | 7661 | 101 | 98 | 7 | 1.30 | 6.67 |
| bn | Subtract y | hyper+fear | Fear | 0.35 | 0.19 | 0.50 | **0.0000** | 8054 | 133 | 68 | 8 | 1.62 | 10.53 |
| bn | Subtract y | argues+fear | Fear | 0.33 | 0.17 | 0.49 | **0.0001** | 7933 | 113 | 74 | 7 | 1.40 | 8.64 |
| bn | Subtract y | temper+fear | Fear | 0.56 | 0.44 | 0.68 | **0.0000** | 8321 | 139 | 45 | 16 | 1.64 | 26.23 |
| bn | Subtract y | fights+fear | Fear | 0.31 | -.04 | 0.66 | 0.0866 | 8552 | 164 | 6 | 1 | 1.88 | 14.29 |
| bn | Subtract y | steals+fear | Fear | 0.41 | 0.04 | 0.78 | **0.0310** | 8550 | 160 | 3 | 1 | 1.84 | 25.00 |
| bn | Subtract y | dysph+worry | Worry | 0.49 | 0.34 | 0.63 | **0.0000** | 8323 | 222 | 24 | 10 | 2.60 | 29.41 |
| bn | Subtract y | worth+worry | Worry | 0.38 | 0.22 | 0.53 | **0.0000** | 8317 | 221 | 36 | 8 | 2.59 | 18.18 |
| bn | Subtract y | attend+worry | Worry | 0.25 | 0.12 | 0.38 | **0.0001** | 7469 | 169 | 141 | 12 | 2.21 | 7.84 |
| bn | Subtract y | hyper+worry | Worry | 0.44 | 0.33 | 0.56 | **0.0000** | 7828 | 205 | 75 | 19 | 2.55 | 20.21 |
| bn | Subtract y | argues+worry | Worry | 0.22 | 0.07 | 0.37 | **0.0042** | 7738 | 172 | 109 | 8 | 2.17 | 6.84 |
| bn | Subtract y | temper+worry | Worry | 0.53 | 0.42 | 0.63 | **0.0000** | 8101 | 202 | 55 | 21 | 2.43 | 27.63 |
| bn | Subtract y | fights+worry | Worry | 0.33 | 0.05 | 0.60 | **0.0196** | 8296 | 251 | 8 | 2 | 2.94 | 20.00 |
| bn | Subtract y | steals+worry | Worry | . | . | . | . | 8295 | 245 | 6 | 0 | 2.87 | 0.00 |
| bn | Subtract y | worth+dysph | Dysph | 0.30 | -.03 | 0.63 | 0.0785 | 8781 | 59 | 17 | 1 | 0.67 | 5.56 |
| bn | Subtract y | attend+dysph | Dysph | 0.22 | -.10 | 0.54 | 0.1805 | 7803 | 46 | 35 | 1 | 0.59 | 2.78 |
| bn | Subtract y | hyper+dysph | Dysph | 0.28 | -.05 | 0.61 | 0.1014 | 8224 | 62 | 18 | 1 | 0.75 | 5.26 |
| bn | Subtract y | argues+dysph | Dysph | 0.33 | 0.07 | 0.58 | **0.0124** | 8109 | 50 | 36 | 2 | 0.61 | 5.26 |
| bn | Subtract y | temper+dysph | Dysph | . | . | . | . | 8524 | 63 | 32 | 0 | 0.73 | 0.00 |
| bn | Subtract y | fights+dysph | Dysph | 0.49 | 0.12 | 0.85 | **0.0086** | 8786 | 83 | 3 | 1 | 0.94 | 25.00 |
| bn | Subtract y | steals+dysph | Dysph | . | . | . | . | 8782 | 84 | 4 | 0 | 0.95 | 0.00 |
| bn | Subtract y | attend+worth | Worth | 0.22 | -.10 | 0.54 | 0.1831 | 7804 | 37 | 44 | 1 | 0.47 | 2.22 |
| bn | Subtract y | hyper+worth | Worth | 0.39 | 0.13 | 0.65 | **0.0034** | 8214 | 58 | 20 | 2 | 0.70 | 9.09 |
| bn | Subtract y | argues+worth | Worth | 0.22 | -.10 | 0.54 | 0.1858 | 8098 | 45 | 38 | 1 | 0.55 | 2.56 |
| bn | Subtract y | temper+worth | Worth | 0.24 | -.08 | 0.56 | 0.1451 | 8519 | 50 | 30 | 1 | 0.58 | 3.23 |
| bn | Subtract y | fights+worth | Worth | . | . | . | . | 8772 | 79 | 3 | 0 | 0.89 | 0.00 |
| bn | Subtract y | steals+worth | Worth | . | . | . | . | 8774 | 74 | 3 | 0 | 0.84 | 0.00 |
| bn | Subtract y | hyper+attend | Attend | 0.46 | 0.40 | 0.53 | **0.0000** | 7453 | 265 | 385 | 91 | 3.43 | 19.12 |
| bn | Subtract y | argues+attend | Attend | 0.44 | 0.36 | 0.51 | **0.0000** | 7188 | 232 | 267 | 57 | 3.13 | 17.59 |
| bn | Subtract y | temper+attend | Attend | 0.59 | 0.52 | 0.66 | **0.0000** | 7385 | 314 | 105 | 65 | 4.08 | 38.24 |
| bn | Subtract y | fights+attend | Attend | 0.43 | 0.27 | 0.59 | **0.0000** | 7495 | 370 | 17 | 9 | 4.70 | 34.62 |
| bn | Subtract y | steals+attend | Attend | 0.50 | 0.36 | 0.65 | **0.0000** | 7501 | 363 | 16 | 12 | 4.62 | 42.86 |
| bn | Subtract y | argues+hyper | Hyper | 0.52 | 0.43 | 0.62 | **0.0000** | 7662 | 107 | 186 | 33 | 1.38 | 15.07 |
| bn | Subtract y | temper+hyper | Hyper | 0.66 | 0.58 | 0.73 | **0.0000** | 7967 | 138 | 83 | 39 | 1.70 | 31.97 |
| bn | Subtract y | fights+hyper | Hyper | 0.55 | 0.37 | 0.73 | **0.0000** | 8135 | 174 | 10 | 6 | 2.09 | 37.50 |
| bn | Subtract y | steals+hyper | Hyper | 0.63 | 0.48 | 0.77 | **0.0000** | 8135 | 174 | 10 | 9 | 2.09 | 47.37 |
| bn | Subtract y | temper+argues | argues | 0.57 | 0.50 | 0.64 | **0.0000** | 7733 | 318 | 141 | 73 | 3.95 | 34.11 |
| bn | Subtract y | fights+argues | argues | 0.48 | 0.34 | 0.62 | **0.0000** | 7772 | 404 | 17 | 12 | 4.94 | 41.38 |
| bn | Subtract y | steals+argues | argues | 0.55 | 0.40 | 0.70 | **0.0000** | 7770 | 395 | 10 | 11 | 4.84 | 52.38 |
| bn | Subtract y | fights+temper | temper | 0.40 | 0.20 | 0.60 | **0.0001** | 8454 | 150 | 19 | 4 | 1.74 | 17.39 |
| bn | Subtract y | steals+temper | temper | 0.25 | -.09 | 0.58 | 0.1523 | 8440 | 149 | 10 | 1 | 1.73 | 9.09 |
| bn | Subtract y | steals+fights | Fights | . | . | . | . | 8871 | 20 | 3 | 0 | 0.22 | 0.00 |

| Supplemental Table S3K. Tetrachoric correlations from 4 x 4 pairwise analyses of all 90 combinations of the 10 selected psychological problems with every other problem to quantify the associations of each baseline problem with outcomes in the **third annual follow-up** that define four parsed paths traditionally conflated in estimates of change: ***add path*** (x_1_ at baseline and x_2_ + y_2_ at follow-up); ***joint persist path*** (x_1_ + y_1_ at baseline and x_2_ + y_2_ at follow-up); ***shift path*** (only x_1_ at baseline and only y_2_ at follow-up ***subtract x path*** (x_1_ + y_1_ at baseline and only y_2_ at follow-up); and ***subtract y path*** (x_1_ + y_1_ at baseline and only x_2_ at follow-up). Results are for problems dichotomized at the **low rating cut (0 vs 1 or 2).** r_t_ = tetrachoric correlation. –CL and +CL = lower and upper 95% confidence intervals for the tetrachoric correlation. c00 = number without the predictor or the outcome; c01 = number without predictor with the outcome; c10 = number with the predictor without the outcome; c11 = number with the predictor and the outcome. Base% = percent of the outcome only x_2_ among children with neither x_1_ nor the other member of the pair, y_1_, at baseline. Obs% = Observed percent of outcomes on the indicated path. Raw P values significant after FDR adjustment in **bold**. | | | | | | | | | | | | | |
| --- | --- | --- | --- | --- | --- | --- | --- | --- | --- | --- | --- | --- | --- |
| prefix | Parsed paths | Predictor | Outcome | r_t_ | -CL | +CL | P | c00 | c01 | c10 | c11 | Base% | Obs% |
| dh | Add | fear | fear+worry | 0.41 | 0.35 | 0.48 | **0.0000** | 4665 | 311 | 386 | 117 | 6.25 | 23.26 |
| dl | Add | worry | worry+fear | 0.36 | 0.31 | 0.41 | **0.0000** | 4665 | 311 | 1234 | 272 | 6.25 | 18.06 |
| dh | Add | fear | fear+dysph | 0.38 | 0.33 | 0.43 | **0.0000** | 5747 | 276 | 1109 | 202 | 4.58 | 15.41 |
| dl | Add | dysph | dysph+fear | 0.29 | 0.22 | 0.37 | **0.0000** | 5747 | 276 | 397 | 62 | 4.58 | 13.51 |
| dh | Add | fear | fear+worth | 0.38 | 0.32 | 0.44 | **0.0000** | 5799 | 198 | 1125 | 154 | 3.30 | 12.04 |
| dl | Add | Worth | worth+fear | 0.36 | 0.28 | 0.44 | **0.0000** | 5799 | 198 | 422 | 62 | 3.30 | 12.81 |
| dh | Add | Fear | fear+attend | 0.41 | 0.33 | 0.48 | **0.0000** | 3540 | 138 | 506 | 89 | 3.75 | 14.96 |
| dl | Add | Attend | attend+fear | 0.38 | 0.33 | 0.43 | **0.0000** | 3540 | 138 | 2450 | 354 | 3.75 | 12.62 |
| dh | Add | fear | fear+hyper | 0.38 | 0.31 | 0.45 | **0.0000** | 4605 | 114 | 849 | 90 | 2.42 | 9.58 |
| dl | Add | hyper | hyper+fear | 0.39 | 0.33 | 0.45 | **0.0000** | 4605 | 114 | 1596 | 167 | 2.42 | 9.47 |
| dh | Add | fear | fear+argues | 0.45 | 0.39 | 0.52 | **0.0000** | 3791 | 167 | 578 | 129 | 4.22 | 18.25 |
| dl | Add | argues | argues+fear | 0.35 | 0.30 | 0.40 | **0.0000** | 3791 | 167 | 2206 | 318 | 4.22 | 12.60 |
| dh | Add | fear | fear+temper | 0.23 | 0.15 | 0.31 | **0.0000** | 5038 | 134 | 974 | 64 | 2.59 | 6.17 |
| dl | Add | temper | temper+fear | 0.38 | 0.31 | 0.44 | **0.0000** | 5038 | 134 | 1181 | 129 | 2.59 | 9.85 |
| dh | Add | fear | fear+fights | 0.28 | 0.18 | 0.39 | **0.0000** | 6071 | 40 | 1546 | 36 | 0.65 | 2.28 |
| dl | Add | fights | fights+fear | 0.42 | 0.29 | 0.54 | **0.0000** | 6071 | 40 | 353 | 18 | 0.65 | 4.85 |
| dh | Add | fear | fear+steals | 0.39 | 0.29 | 0.48 | **0.0000** | 6236 | 28 | 1653 | 43 | 0.45 | 2.54 |
| dl | Add | steals | steals+fear | 0.46 | 0.32 | 0.61 | **0.0000** | 6236 | 28 | 207 | 11 | 0.45 | 5.05 |
| dh | Add | worry | worry+dysph | 0.36 | 0.31 | 0.41 | **0.0000** | 4890 | 310 | 1770 | 364 | 5.96 | 17.06 |
| dl | Add | dysph | dysph+worry | 0.32 | 0.24 | 0.41 | **0.0000** | 4890 | 310 | 226 | 53 | 5.96 | 19.00 |
| dh | Add | worry | worry+worth | 0.31 | 0.25 | 0.36 | **0.0000** | 4954 | 210 | 1882 | 230 | 4.07 | 10.89 |
| dl | Add | worth | worth+worry | 0.41 | 0.33 | 0.49 | **0.0000** | 4954 | 210 | 257 | 58 | 4.07 | 18.41 |
| dh | Add | worry | worry+attend | 0.36 | 0.30 | 0.43 | **0.0000** | 2985 | 164 | 949 | 175 | 5.21 | 15.57 |
| dl | Add | attend | attend+worry | 0.41 | 0.36 | 0.46 | **0.0000** | 2985 | 164 | 1920 | 410 | 5.21 | 17.60 |
| dh | Add | worry | worry+hyper | 0.34 | 0.27 | 0.41 | **0.0000** | 3893 | 110 | 1509 | 146 | 2.75 | 8.82 |
| dl | Add | hyper | hyper+worry | 0.44 | 0.38 | 0.50 | **0.0000** | 3893 | 110 | 1297 | 179 | 2.75 | 12.13 |
| dh | Add | worry | worry+argues | 0.41 | 0.35 | 0.46 | **0.0000** | 3264 | 199 | 975 | 227 | 5.75 | 18.89 |
| dl | Add | argues | argues+worry | 0.39 | 0.33 | 0.44 | **0.0000** | 3264 | 199 | 1662 | 354 | 5.75 | 17.56 |
| dh | Add | worry | worry+temper | 0.31 | 0.24 | 0.37 | **0.0000** | 4303 | 128 | 1632 | 147 | 2.89 | 8.26 |
| dl | Add | temper | temper+worry | 0.47 | 0.41 | 0.53 | **0.0000** | 4303 | 128 | 899 | 149 | 2.89 | 14.22 |
| dh | Add | worry | worry+fights | 0.22 | 0.13 | 0.31 | **0.0000** | 5136 | 47 | 2454 | 56 | 0.91 | 2.23 |
| dl | Add | fights | fights+worry | 0.38 | 0.25 | 0.51 | **0.0000** | 5136 | 47 | 280 | 16 | 0.91 | 5.41 |
| dh | Add | worry | worry+steals | 0.34 | 0.25 | 0.42 | **0.0000** | 5296 | 34 | 2560 | 70 | 0.64 | 2.66 |
| dl | Add | steals | steals+worry | 0.60 | 0.48 | 0.71 | **0.0000** | 5296 | 34 | 131 | 18 | 0.64 | 12.08 |
| dh | Add | dysph | dysph+worth | 0.29 | 0.22 | 0.36 | **0.0000** | 6443 | 332 | 430 | 71 | 4.90 | 14.17 |
| dl | Add | worth | worth+dysph | 0.40 | 0.34 | 0.46 | **0.0000** | 6443 | 332 | 450 | 108 | 4.90 | 19.35 |
| dh | Add | dysph | dysph+attend | 0.29 | 0.19 | 0.39 | **0.0000** | 3822 | 205 | 209 | 37 | 5.09 | 15.04 |
| dl | Add | attend | attend+dysph | 0.34 | 0.30 | 0.39 | **0.0000** | 3822 | 205 | 2834 | 473 | 5.09 | 14.30 |
| dh | Add | dysph | dysph+hyper | 0.37 | 0.28 | 0.45 | **0.0000** | 5063 | 131 | 415 | 49 | 2.52 | 10.56 |
| dl | Add | hyper | hyper+dysph | 0.41 | 0.35 | 0.46 | **0.0000** | 5063 | 131 | 1920 | 220 | 2.52 | 10.28 |
| dh | Add | dysph | dysph+argues | 0.37 | 0.28 | 0.46 | **0.0000** | 4182 | 227 | 207 | 49 | 5.15 | 19.14 |
| dl | Add | argues | argues+dysph | 0.36 | 0.31 | 0.40 | **0.0000** | 4182 | 227 | 2488 | 437 | 5.15 | 14.94 |
| dh | Add | dysph | dysph+temper | 0.25 | 0.15 | 0.34 | **0.0000** | 5627 | 157 | 394 | 32 | 2.71 | 7.51 |
| dl | Add | temper | temper+dysph | 0.46 | 0.41 | 0.51 | **0.0000** | 5627 | 157 | 1346 | 204 | 2.71 | 13.16 |
| dh | Add | dysph | dysph+fights | 0.39 | 0.30 | 0.49 | **0.0000** | 6882 | 53 | 724 | 34 | 0.76 | 4.49 |
| dl | Add | fights | fights+dysph | 0.47 | 0.36 | 0.57 | **0.0000** | 6882 | 53 | 372 | 27 | 0.76 | 6.77 |
| dh | Add | dysph | dysph+steals | 0.34 | 0.25 | 0.44 | **0.0000** | 7046 | 59 | 822 | 33 | 0.83 | 3.86 |
| dl | Add | steals | steals+dysph | 0.54 | 0.44 | 0.64 | **0.0000** | 7046 | 59 | 204 | 25 | 0.83 | 10.92 |
| dh | Add | worth | worth+attend | 0.38 | 0.28 | 0.49 | **0.0000** | 3918 | 122 | 201 | 32 | 3.02 | 13.73 |
| dl | Add | attend | attend+worth | 0.40 | 0.35 | 0.45 | **0.0000** | 3918 | 122 | 2868 | 368 | 3.02 | 11.37 |
| dh | Add | worth | worth+hyper | 0.45 | 0.36 | 0.53 | **0.0000** | 5089 | 95 | 421 | 52 | 1.83 | 10.99 |
| dl | Add | hyper | hyper+worth | 0.43 | 0.37 | 0.49 | **0.0000** | 5089 | 95 | 1910 | 182 | 1.83 | 8.70 |
| dh | Add | worth | worth+argues | 0.45 | 0.36 | 0.53 | **0.0000** | 4220 | 130 | 264 | 51 | 2.99 | 16.19 |
| dl | Add | argues | argues+worth | 0.40 | 0.35 | 0.46 | **0.0000** | 4220 | 130 | 2590 | 336 | 2.99 | 11.48 |
| dh | Add | worth | worth+temper | 0.37 | 0.28 | 0.46 | **0.0000** | 5604 | 107 | 455 | 43 | 1.87 | 8.63 |
| dl | Add | temper | temper+worth | 0.49 | 0.44 | 0.55 | **0.0000** | 5604 | 107 | 1389 | 176 | 1.87 | 11.25 |
| dh | Add | worth | worth+fights | 0.34 | 0.23 | 0.44 | **0.0000** | 6789 | 53 | 820 | 30 | 0.77 | 3.53 |
| dl | Add | fights | fights+worth | 0.42 | 0.31 | 0.53 | **0.0000** | 6789 | 53 | 410 | 24 | 0.77 | 5.53 |
| dh | Add | worth | worth+steals | 0.32 | 0.21 | 0.42 | **0.0000** | 7004 | 49 | 879 | 27 | 0.69 | 2.98 |
| dl | Add | steals | steals+worth | 0.57 | 0.47 | 0.67 | **0.0000** | 7004 | 49 | 198 | 25 | 0.69 | 11.21 |
| dh | Add | attend | attend+hyper | 0.42 | 0.37 | 0.47 | **0.0000** | 3735 | 197 | 1419 | 307 | 5.01 | 17.79 |
| dl | Add | hyper | hyper+attend | 0.33 | 0.25 | 0.42 | **0.0000** | 3735 | 197 | 286 | 55 | 5.01 | 16.13 |
| dh | Add | attend | attend+argues | 0.47 | 0.42 | 0.52 | **0.0000** | 2791 | 209 | 1247 | 418 | 6.97 | 25.11 |
| dl | Add | argues | argues+attend | 0.35 | 0.29 | 0.41 | **0.0000** | 2791 | 209 | 1037 | 236 | 6.97 | 18.54 |
| dh | Add | attend | attend+temper | 0.36 | 0.31 | 0.42 | **0.0000** | 3524 | 118 | 2293 | 275 | 3.24 | 10.71 |
| dl | Add | temper | temper+attend | 0.43 | 0.36 | 0.51 | **0.0000** | 3524 | 118 | 540 | 91 | 3.24 | 14.42 |
| dh | Add | attend | attend+fights | 0.35 | 0.27 | 0.43 | **0.0000** | 4111 | 31 | 3435 | 116 | 0.75 | 3.27 |
| dl | Add | fights | fights+attend | 0.49 | 0.34 | 0.64 | **0.0000** | 4111 | 31 | 120 | 11 | 0.75 | 8.40 |
| dh | Add | attend | attend+steals | 0.36 | 0.29 | 0.43 | **0.0000** | 4187 | 37 | 3592 | 144 | 0.88 | 3.85 |
| dl | Add | steals | steals+attend | 0.53 | 0.36 | 0.71 | **0.0000** | 4187 | 37 | 42 | 7 | 0.88 | 14.29 |
| dh | Add | hyper | hyper+argues | 0.50 | 0.44 | 0.56 | **0.0000** | 3575 | 152 | 753 | 185 | 4.08 | 19.72 |
| dl | Add | argues | argues+hyper | 0.36 | 0.30 | 0.42 | **0.0000** | 3575 | 152 | 1686 | 245 | 4.08 | 12.69 |
| dh | Add | hyper | hyper+temper | 0.47 | 0.40 | 0.53 | **0.0000** | 4559 | 83 | 1414 | 154 | 1.79 | 9.82 |
| dl | Add | temper | temper+hyper | 0.48 | 0.42 | 0.55 | **0.0000** | 4559 | 83 | 906 | 110 | 1.79 | 10.83 |
| dh | Add | hyper | hyper+fights | 0.36 | 0.27 | 0.45 | **0.0000** | 5388 | 36 | 2200 | 69 | 0.66 | 3.04 |
| dl | Add | fights | fights+hyper | 0.48 | 0.35 | 0.61 | **0.0000** | 5388 | 36 | 218 | 16 | 0.66 | 6.84 |
| dh | Add | hyper | hyper+steals | 0.37 | 0.29 | 0.44 | **0.0000** | 5499 | 44 | 2330 | 87 | 0.79 | 3.60 |
| dl | Add | steals | steals+hyper | 0.56 | 0.44 | 0.69 | **0.0000** | 5499 | 44 | 100 | 15 | 0.79 | 13.04 |
| dh | Add | argues | argues+temper | 0.39 | 0.34 | 0.45 | **0.0000** | 4063 | 185 | 1672 | 290 | 4.35 | 14.78 |
| dl | Add | temper | temper+argues | 0.45 | 0.38 | 0.53 | **0.0000** | 4063 | 185 | 332 | 85 | 4.35 | 20.38 |
| dh | Add | argues | argues+fights | 0.39 | 0.32 | 0.46 | **0.0000** | 4541 | 40 | 2977 | 135 | 0.87 | 4.34 |
| dl | Add | fights | fights+argues | 0.52 | 0.36 | 0.67 | **0.0000** | 4541 | 40 | 74 | 10 | 0.87 | 11.90 |
| dh | Add | argues | argues+steals | 0.34 | 0.27 | 0.42 | **0.0000** | 4562 | 45 | 3223 | 130 | 0.98 | 3.88 |
| dl | Add | steals | steals+argues | 0.59 | 0.45 | 0.73 | **0.0000** | 4562 | 45 | 47 | 11 | 0.98 | 18.97 |
| dh | Add | temper | temper+fights | 0.47 | 0.39 | 0.54 | **0.0000** | 6011 | 46 | 1549 | 87 | 0.76 | 5.32 |
| dl | Add | fights | fights+temper | 0.41 | 0.27 | 0.56 | **0.0000** | 6011 | 46 | 143 | 10 | 0.76 | 6.54 |
| dh | Add | temper | temper+steals | 0.41 | 0.32 | 0.49 | **0.0000** | 6038 | 42 | 1808 | 72 | 0.69 | 3.83 |
| dl | Add | steals | steals+temper | 0.50 | 0.37 | 0.64 | **0.0000** | 6038 | 42 | 118 | 12 | 0.69 | 9.23 |
| dh | Add | fights | fights+steals | 0.48 | 0.37 | 0.60 | **0.0000** | 7437 | 29 | 473 | 21 | 0.39 | 4.25 |
| dl | Add | steals | steals+fights | 0.55 | 0.43 | 0.67 | **0.0000** | 7437 | 29 | 211 | 16 | 0.39 | 7.05 |
| dp | Joint Persist | fear+worry | fear+worry | 0.71 | 0.68 | 0.74 | **0.0000** | 4665 | 311 | 729 | 593 | 6.25 | 44.86 |
| dp | Joint Persist | fear+dysph | fear+dysph | 0.66 | 0.62 | 0.71 | **0.0000** | 5747 | 276 | 314 | 200 | 4.58 | 38.91 |
| dp | Joint Persist | fear+worth | fear+worth | 0.71 | 0.66 | 0.75 | **0.0000** | 5799 | 198 | 345 | 201 | 3.30 | 36.81 |
| dp | Joint Persist | fear+attend | fear+attend | 0.79 | 0.76 | 0.81 | **0.0000** | 3540 | 138 | 693 | 537 | 3.75 | 43.66 |
| dp | Joint Persist | fear+hyper | fear+hyper | 0.76 | 0.73 | 0.80 | **0.0000** | 4605 | 114 | 580 | 306 | 2.42 | 34.54 |
| dp | Joint Persist | fear+argues | fear+argues | 0.74 | 0.71 | 0.77 | **0.0000** | 3791 | 167 | 668 | 450 | 4.22 | 40.25 |
| dp | Joint Persist | fear+temper | fear+temper | 0.74 | 0.70 | 0.78 | **0.0000** | 5038 | 134 | 523 | 264 | 2.59 | 33.55 |
| dp | Joint Persist | fear+fights | fear+fights | 0.70 | 0.63 | 0.78 | **0.0000** | 6071 | 40 | 200 | 43 | 0.65 | 17.70 |
| dp | Joint Persist | fear+steals | fear+steals | 0.81 | 0.74 | 0.88 | **0.0000** | 6236 | 28 | 93 | 36 | 0.45 | 27.91 |
| dp | Joint Persist | worry+dysph | worry+dysph | 0.66 | 0.61 | 0.70 | **0.0000** | 4890 | 310 | 410 | 284 | 5.96 | 40.92 |
| dp | Joint Persist | worry+worth | worry+worth | 0.71 | 0.67 | 0.75 | **0.0000** | 4954 | 210 | 435 | 280 | 4.07 | 39.16 |
| dp | Joint Persist | worry+attend | worry+attend | 0.78 | 0.75 | 0.80 | **0.0000** | 2985 | 164 | 886 | 818 | 5.21 | 48.00 |
| dp | Joint Persist | worry+hyper | worry+hyper | 0.78 | 0.75 | 0.81 | **0.0000** | 3893 | 110 | 724 | 449 | 2.75 | 38.28 |
| dp | Joint Persist | worry+argues | worry+argues | 0.75 | 0.72 | 0.77 | **0.0000** | 3264 | 199 | 878 | 748 | 5.75 | 46.00 |
| dp | Joint Persist | worry+temper | worry+temper | 0.76 | 0.73 | 0.79 | **0.0000** | 4303 | 128 | 664 | 385 | 2.89 | 36.70 |
| dp | Joint Persist | worry+fights | worry+fights | 0.73 | 0.67 | 0.79 | **0.0000** | 5136 | 47 | 248 | 70 | 0.91 | 22.01 |
| dp | Joint Persist | worry+steals | worry+steals | 0.81 | 0.75 | 0.87 | **0.0000** | 5296 | 34 | 141 | 57 | 0.64 | 28.79 |
| dp | Joint Persist | dysph+worth | dysph+worth | 0.63 | 0.58 | 0.67 | **0.0000** | 6443 | 332 | 294 | 178 | 4.90 | 37.71 |
| dp | Joint Persist | dysph+attend | dysph+attend | 0.70 | 0.66 | 0.74 | **0.0000** | 3822 | 205 | 429 | 298 | 5.09 | 40.99 |
| dp | Joint Persist | dysph+hyper | dysph+hyper | 0.72 | 0.68 | 0.77 | **0.0000** | 5063 | 131 | 340 | 169 | 2.52 | 33.20 |
| dp | Joint Persist | dysph+argues | dysph+argues | 0.69 | 0.65 | 0.73 | **0.0000** | 4182 | 227 | 425 | 292 | 5.15 | 40.73 |
| dp | Joint Persist | dysph+temper | dysph+temper | 0.72 | 0.68 | 0.76 | **0.0000** | 5627 | 157 | 357 | 190 | 2.71 | 34.73 |
| dp | Joint Persist | dysph+fights | dysph+fights | 0.67 | 0.59 | 0.75 | **0.0000** | 6882 | 53 | 176 | 39 | 0.76 | 18.14 |
| dp | Joint Persist | dysph+steals | dysph+steals | 0.74 | 0.67 | 0.82 | **0.0000** | 7046 | 59 | 83 | 35 | 0.83 | 29.66 |
| dp | Joint Persist | worth+attend | worth+attend | 0.78 | 0.75 | 0.81 | **0.0000** | 3918 | 122 | 476 | 321 | 3.02 | 40.28 |
| dp | Joint Persist | worth+hyper | worth+hyper | 0.76 | 0.72 | 0.80 | **0.0000** | 5089 | 95 | 379 | 178 | 1.83 | 31.96 |
| dp | Joint Persist | worth+argues | worth+argues | 0.77 | 0.74 | 0.81 | **0.0000** | 4220 | 130 | 428 | 287 | 2.99 | 40.14 |
| dp | Joint Persist | worth+temper | worth+temper | 0.76 | 0.72 | 0.80 | **0.0000** | 5604 | 107 | 355 | 177 | 1.87 | 33.27 |
| dp | Joint Persist | worth+fights | worth+fights | 0.66 | 0.57 | 0.74 | **0.0000** | 6789 | 53 | 148 | 32 | 0.77 | 17.78 |
| dp | Joint Persist | worth+steals | worth+steals | 0.71 | 0.63 | 0.80 | **0.0000** | 7004 | 49 | 95 | 29 | 0.69 | 23.39 |
| dp | Joint Persist | attend+hyper | attend+hyper | 0.82 | 0.80 | 0.84 | **0.0000** | 3735 | 197 | 1068 | 1240 | 5.01 | 53.73 |
| dp | Joint Persist | attend+argues | attend+argues | 0.82 | 0.80 | 0.84 | **0.0000** | 2791 | 209 | 924 | 1445 | 6.97 | 61.00 |
| dp | Joint Persist | attend+temper | attend+temper | 0.81 | 0.79 | 0.84 | **0.0000** | 3524 | 118 | 809 | 657 | 3.24 | 44.82 |
| dp | Joint Persist | attend+fights | attend+fights | 0.81 | 0.76 | 0.85 | **0.0000** | 4111 | 31 | 360 | 123 | 0.75 | 25.47 |
| dp | Joint Persist | attend+steals | attend+steals | 0.83 | 0.78 | 0.87 | **0.0000** | 4187 | 37 | 203 | 95 | 0.88 | 31.88 |
| dp | Joint Persist | hyper+argues | hyper+argues | 0.80 | 0.78 | 0.82 | **0.0000** | 3575 | 152 | 907 | 804 | 4.08 | 46.99 |
| dp | Joint Persist | hyper+temper | hyper+temper | 0.82 | 0.79 | 0.85 | **0.0000** | 4559 | 83 | 679 | 402 | 1.79 | 37.19 |
| dp | Joint Persist | hyper+fights | hyper+fights | 0.78 | 0.73 | 0.84 | **0.0000** | 5388 | 36 | 293 | 87 | 0.66 | 22.89 |
| dp | Joint Persist | hyper+steals | hyper+steals | 0.77 | 0.71 | 0.83 | **0.0000** | 5499 | 44 | 171 | 61 | 0.79 | 26.29 |
| dp | Joint Persist | argues+temper | argues+temper | 0.78 | 0.75 | 0.80 | **0.0000** | 4063 | 185 | 928 | 752 | 4.35 | 44.76 |
| dp | Joint Persist | argues+fights | argues+fights | 0.80 | 0.76 | 0.85 | **0.0000** | 4541 | 40 | 389 | 141 | 0.87 | 26.60 |
| dp | Joint Persist | argues+steals | argues+steals | 0.81 | 0.76 | 0.86 | **0.0000** | 4562 | 45 | 196 | 93 | 0.98 | 32.18 |
| dp | Joint Persist | temper+fights | temper+fights | 0.78 | 0.74 | 0.83 | **0.0000** | 6011 | 46 | 350 | 111 | 0.76 | 24.08 |
| dp | Joint Persist | temper+steals | temper+steals | 0.80 | 0.75 | 0.86 | **0.0000** | 6038 | 42 | 153 | 64 | 0.69 | 29.49 |
| dp | Joint Persist | fights+steals | fights+steals | 0.78 | 0.70 | 0.85 | **0.0000** | 7437 | 29 | 92 | 28 | 0.39 | 23.33 |
| cg | Shift | fear | worry | 0.03 | -.05 | 0.10 | 0.5133 | 4402 | 574 | 440 | 63 | 11.54 | 12.52 |
| bj | Shift | worry | fear | 0.03 | -.05 | 0.11 | 0.4627 | 4795 | 181 | 1445 | 61 | 3.64 | 4.05 |
| cg | Shift | fear | dysph | -.06 | -.12 | 0.01 | 0.0803 | 5549 | 474 | 1226 | 85 | 7.87 | 6.48 |
| bj | Shift | dysph | fear | 0.08 | -.00 | 0.16 | 0.0621 | 5581 | 442 | 414 | 45 | 7.34 | 9.80 |
| cg | Shift | fear | worth | -.08 | -.16 | -.01 | **0.0360** | 5713 | 284 | 1235 | 44 | 4.74 | 3.44 |
| bj | Shift | worth | fear | -.02 | -.10 | 0.07 | 0.7113 | 5472 | 525 | 444 | 40 | 8.75 | 8.26 |
| cg | Shift | fear | attend | -.14 | -.22 | -.07 | **0.0001** | 3076 | 602 | 532 | 63 | 16.37 | 10.59 |
| bj | Shift | attend | fear | -.26 | -.33 | -.20 | **0.0000** | 3422 | 256 | 2727 | 77 | 6.96 | 2.75 |
| cg | Shift | fear | hyper | -.06 | -.14 | 0.01 | 0.0906 | 4371 | 348 | 884 | 55 | 7.37 | 5.86 |
| bj | Shift | hyper | fear | -.10 | -.16 | -.04 | **0.0006** | 4290 | 429 | 1648 | 115 | 9.09 | 6.52 |
| cg | Shift | fear | argues | -.08 | -.14 | -.02 | **0.0154** | 3205 | 753 | 599 | 108 | 19.02 | 15.28 |
| bj | Shift | argues | fear | -.20 | -.27 | -.14 | **0.0000** | 3699 | 259 | 2443 | 81 | 6.54 | 3.21 |
| cg | Shift | fear | temper | -.07 | -.15 | 0.00 | 0.0613 | 4841 | 331 | 987 | 51 | 6.40 | 4.91 |
| bj | Shift | temper | fear | -.01 | -.07 | 0.05 | 0.6929 | 4720 | 452 | 1200 | 110 | 8.74 | 8.40 |
| cg | Shift | fear | fights | -.11 | -.22 | -.01 | **0.0382** | 6007 | 104 | 1566 | 16 | 1.70 | 1.01 |
| bj | Shift | fights | fear | -.01 | -.09 | 0.07 | 0.8813 | 5387 | 724 | 328 | 43 | 11.85 | 11.59 |
| cg | Shift | fear | steals | -.06 | -.16 | 0.04 | 0.2136 | 6148 | 116 | 1672 | 24 | 1.85 | 1.42 |
| bj | Shift | steals | fear | -.10 | -.20 | 0.00 | 0.0619 | 5496 | 768 | 200 | 18 | 12.26 | 8.26 |
| cg | Shift | worry | dysph | -.06 | -.13 | 0.00 | 0.0597 | 4927 | 273 | 2044 | 90 | 5.25 | 4.22 |
| bj | Shift | dysph | worry | 0.02 | -.07 | 0.11 | 0.6810 | 4536 | 664 | 241 | 38 | 12.77 | 13.62 |
| cg | Shift | worry | worth | -.03 | -.11 | 0.04 | 0.4088 | 4988 | 176 | 2048 | 64 | 3.41 | 3.03 |
| bj | Shift | worth | worry | -.01 | -.10 | 0.07 | 0.7632 | 4411 | 753 | 271 | 44 | 14.58 | 13.97 |
| cg | Shift | worry | attend | -.17 | -.24 | -.11 | **0.0000** | 2694 | 455 | 1026 | 98 | 14.45 | 8.72 |
| bj | Shift | attend | worry | -.31 | -.37 | -.26 | **0.0000** | 2764 | 385 | 2224 | 106 | 12.23 | 4.55 |
| cg | Shift | worry | hyper | -.13 | -.20 | -.06 | **0.0003** | 3726 | 277 | 1581 | 74 | 6.92 | 4.47 |
| bj | Shift | hyper | worry | -.19 | -.25 | -.13 | **0.0000** | 3364 | 639 | 1339 | 137 | 15.96 | 9.28 |
| cg | Shift | worry | argues | -.12 | -.18 | -.06 | **0.0001** | 2880 | 583 | 1054 | 148 | 16.84 | 12.31 |
| bj | Shift | argues | worry | -.22 | -.28 | -.16 | **0.0000** | 3068 | 395 | 1899 | 117 | 11.41 | 5.80 |
| cg | Shift | worry | temper | -.13 | -.20 | -.06 | **0.0004** | 4187 | 244 | 1718 | 61 | 5.51 | 3.43 |
| bj | Shift | temper | worry | -.10 | -.17 | -.04 | **0.0006** | 3760 | 671 | 931 | 117 | 15.14 | 11.16 |
| cg | Shift | worry | fights | -.14 | -.25 | -.04 | **0.0084** | 5109 | 74 | 2491 | 19 | 1.43 | 0.76 |
| bj | Shift | fights | worry | -.07 | -.15 | 0.01 | 0.1080 | 4225 | 958 | 252 | 44 | 18.48 | 14.86 |
| cg | Shift | worry | steals | -.18 | -.28 | -.08 | **0.0003** | 5242 | 88 | 2611 | 19 | 1.65 | 0.72 |
| bj | Shift | steals | worry | -.12 | -.22 | -.01 | **0.0317** | 4335 | 995 | 131 | 18 | 18.67 | 12.08 |
| cg | Shift | dysph | worth | -.05 | -.16 | 0.05 | 0.3097 | 6513 | 262 | 486 | 15 | 3.87 | 2.99 |
| bj | Shift | worth | dysph | 0.05 | -.03 | 0.12 | 0.2320 | 6231 | 544 | 505 | 53 | 8.03 | 9.50 |
| cg | Shift | dysph | attend | -.02 | -.11 | 0.07 | 0.6553 | 3412 | 615 | 211 | 35 | 15.27 | 14.23 |
| bj | Shift | attend | dysph | -.31 | -.37 | -.25 | **0.0000** | 3744 | 283 | 3231 | 76 | 7.03 | 2.30 |
| cg | Shift | dysph | hyper | -.01 | -.10 | 0.08 | 0.8514 | 4801 | 393 | 430 | 34 | 7.57 | 7.33 |
| bj | Shift | hyper | dysph | -.17 | -.23 | -.12 | **0.0000** | 4640 | 554 | 2008 | 132 | 10.67 | 6.17 |
| cg | Shift | dysph | argues | -.01 | -.10 | 0.08 | 0.8244 | 3575 | 834 | 209 | 47 | 18.92 | 18.36 |
| bj | Shift | argues | dysph | -.17 | -.23 | -.11 | **0.0000** | 4137 | 272 | 2824 | 101 | 6.17 | 3.45 |
| cg | Shift | dysph | temper | -.03 | -.12 | 0.07 | 0.5539 | 5417 | 367 | 402 | 24 | 6.35 | 5.63 |
| bj | Shift | temper | dysph | -.09 | -.15 | -.03 | **0.0017** | 5220 | 564 | 1438 | 112 | 9.75 | 7.23 |
| cg | Shift | dysph | fights | -.09 | -.22 | 0.05 | 0.2037 | 6833 | 102 | 751 | 7 | 1.47 | 0.92 |
| bj | Shift | fights | dysph | -.02 | -.10 | 0.05 | 0.5302 | 6026 | 909 | 351 | 48 | 13.11 | 12.03 |
| cg | Shift | dysph | steals | -.01 | -.13 | 0.11 | 0.8141 | 6998 | 107 | 843 | 12 | 1.51 | 1.40 |
| bj | Shift | steals | dysph | 0.01 | -.08 | 0.10 | 0.8049 | 6183 | 922 | 198 | 31 | 12.98 | 13.54 |
| cg | Shift | worth | attend | -.06 | -.16 | 0.03 | 0.1858 | 3335 | 705 | 200 | 33 | 17.45 | 14.16 |
| bj | Shift | attend | worth | -.25 | -.32 | -.17 | **0.0000** | 3894 | 146 | 3192 | 44 | 3.61 | 1.36 |
| cg | Shift | worth | hyper | -.13 | -.22 | -.03 | **0.0069** | 4748 | 436 | 449 | 24 | 8.41 | 5.07 |
| bj | Shift | hyper | worth | -.10 | -.17 | -.04 | **0.0017** | 4870 | 314 | 2003 | 89 | 6.06 | 4.25 |
| cg | Shift | worth | argues | 0.01 | -.07 | 0.09 | 0.7644 | 3442 | 908 | 247 | 68 | 20.87 | 21.59 |
| bj | Shift | argues | worth | -.12 | -.20 | -.05 | **0.0014** | 4200 | 150 | 2862 | 64 | 3.45 | 2.19 |
| cg | Shift | worth | temper | 0.00 | -.08 | 0.09 | 0.9252 | 5316 | 395 | 463 | 35 | 6.92 | 7.03 |
| bj | Shift | temper | worth | -.06 | -.13 | 0.00 | 0.0646 | 5385 | 326 | 1494 | 71 | 5.71 | 4.54 |
| cg | Shift | worth | fights | -.07 | -.20 | 0.06 | 0.2734 | 6738 | 104 | 841 | 9 | 1.52 | 1.06 |
| bj | Shift | fights | worth | 0.00 | -.08 | 0.08 | 0.9954 | 6275 | 567 | 398 | 36 | 8.29 | 8.29 |
| cg | Shift | worth | steals | 0.05 | -.05 | 0.16 | 0.3337 | 6937 | 116 | 887 | 19 | 1.64 | 2.10 |
| bj | Shift | steals | worth | 0.05 | -.05 | 0.15 | 0.2893 | 6470 | 583 | 200 | 23 | 8.27 | 10.31 |
| cg | Shift | attend | hyper | -.03 | -.13 | 0.08 | 0.5985 | 3858 | 74 | 1697 | 29 | 1.88 | 1.68 |
| bj | Shift | hyper | attend | -.05 | -.14 | 0.04 | 0.2540 | 3336 | 596 | 297 | 44 | 15.16 | 12.90 |
| cg | Shift | attend | argues | -.28 | -.34 | -.22 | **0.0000** | 2572 | 428 | 1563 | 102 | 14.27 | 6.13 |
| bj | Shift | argues | attend | -.23 | -.30 | -.16 | **0.0000** | 2630 | 370 | 1196 | 77 | 12.33 | 6.05 |
| cg | Shift | attend | temper | -.23 | -.31 | -.15 | **0.0000** | 3497 | 145 | 2526 | 42 | 3.98 | 1.64 |
| bj | Shift | temper | attend | -.08 | -.15 | -.01 | **0.0227** | 3041 | 601 | 549 | 82 | 16.50 | 13.00 |
| cg | Shift | attend | fights | -.23 | -.36 | -.10 | **0.0005** | 4104 | 38 | 3540 | 11 | 0.92 | 0.31 |
| bj | Shift | fights | attend | -.01 | -.12 | 0.09 | 0.8130 | 3317 | 825 | 106 | 25 | 19.92 | 19.08 |
| cg | Shift | attend | steals | -.21 | -.37 | -.06 | **0.0069** | 4201 | 23 | 3729 | 7 | 0.54 | 0.19 |
| bj | Shift | steals | attend | 0.01 | -.14 | 0.16 | 0.9211 | 3386 | 838 | 39 | 10 | 19.84 | 20.41 |
| cg | Shift | hyper | argues | -.13 | -.19 | -.07 | **0.0000** | 3029 | 698 | 816 | 122 | 18.73 | 13.01 |
| bj | Shift | argues | hyper | -.19 | -.27 | -.11 | **0.0000** | 3559 | 168 | 1889 | 42 | 4.51 | 2.18 |
| cg | Shift | hyper | temper | -.06 | -.13 | 0.01 | 0.0761 | 4372 | 270 | 1495 | 73 | 5.82 | 4.66 |
| bj | Shift | temper | hyper | 0.03 | -.04 | 0.10 | 0.4555 | 4308 | 334 | 936 | 80 | 7.20 | 7.87 |
| cg | Shift | hyper | fights | 0.06 | -.04 | 0.16 | 0.2393 | 5365 | 59 | 2237 | 32 | 1.09 | 1.41 |
| bj | Shift | fights | hyper | 0.16 | 0.07 | 0.25 | **0.0008** | 4908 | 516 | 195 | 39 | 9.51 | 16.67 |
| cg | Shift | hyper | steals | 0.09 | -.02 | 0.19 | 0.1071 | 5494 | 49 | 2386 | 31 | 0.88 | 1.28 |
| bj | Shift | steals | hyper | 0.02 | -.11 | 0.15 | 0.7857 | 5007 | 536 | 103 | 12 | 9.67 | 10.43 |
| cg | Shift | argues | temper | -.03 | -.14 | 0.07 | 0.4978 | 4173 | 75 | 1932 | 30 | 1.77 | 1.53 |
| bj | Shift | temper | argues | 0.06 | -.01 | 0.13 | 0.1018 | 3453 | 795 | 325 | 92 | 18.71 | 22.06 |
| cg | Shift | argues | fights | -.26 | -.45 | -.07 | **0.0087** | 4563 | 18 | 3109 | 3 | 0.39 | 0.10 |
| bj | Shift | fights | argues | 0.09 | -.02 | 0.20 | 0.1257 | 3500 | 1081 | 58 | 26 | 23.60 | 30.95 |
| cg | Shift | argues | steals | -.05 | -.20 | 0.09 | 0.4605 | 4584 | 23 | 3340 | 13 | 0.50 | 0.39 |
| bj | Shift | steals | argues | 0.07 | -.06 | 0.20 | 0.3130 | 3523 | 1084 | 41 | 17 | 23.53 | 29.31 |
| cg | Shift | temper | fights | 0.05 | -.07 | 0.17 | 0.4296 | 6010 | 47 | 1620 | 16 | 0.78 | 0.98 |
| bj | Shift | fights | temper | 0.15 | 0.04 | 0.26 | **0.0059** | 5556 | 501 | 130 | 23 | 8.27 | 15.03 |
| cg | Shift | temper | steals | 0.07 | -.03 | 0.18 | 0.1521 | 6012 | 68 | 1851 | 29 | 1.12 | 1.54 |
| bj | Shift | steals | temper | 0.14 | 0.02 | 0.25 | **0.0179** | 5573 | 507 | 111 | 19 | 8.34 | 14.62 |
| cg | Shift | fights | steals | 0.15 | 0.04 | 0.26 | **0.0065** | 7324 | 142 | 475 | 19 | 1.90 | 3.85 |
| bj | Shift | steals | fights | 0.09 | -.06 | 0.24 | 0.2451 | 7322 | 144 | 220 | 7 | 1.93 | 3.08 |
| co | Subtract x | fear+worry | worry | 0.19 | 0.14 | 0.25 | **0.0000** | 4402 | 574 | 1066 | 256 | 11.54 | 19.36 |
| co | Subtract x | fear+dysph | dysph | 0.16 | 0.08 | 0.23 | **0.0000** | 5549 | 474 | 445 | 69 | 7.87 | 13.42 |
| co | Subtract x | fear+worth | worth | 0.28 | 0.21 | 0.36 | **0.0000** | 5713 | 284 | 475 | 71 | 4.74 | 13.00 |
| co | Subtract x | fear+attend | attend | 0.36 | 0.31 | 0.41 | **0.0000** | 3076 | 602 | 789 | 441 | 16.37 | 35.85 |
| co | Subtract x | fear+hyper | hyper | 0.42 | 0.37 | 0.47 | **0.0000** | 4371 | 348 | 668 | 218 | 7.37 | 24.60 |
| co | Subtract x | fear+argues | argues | 0.24 | 0.19 | 0.29 | **0.0000** | 3205 | 753 | 755 | 363 | 19.02 | 32.47 |
| co | Subtract x | fear+temper | temper | 0.39 | 0.33 | 0.45 | **0.0000** | 4841 | 331 | 622 | 165 | 6.40 | 20.97 |
| co | Subtract x | fear+fights | fights | 0.47 | 0.37 | 0.57 | **0.0000** | 6007 | 104 | 211 | 32 | 1.70 | 13.17 |
| co | Subtract x | fear+steals | steals | 0.40 | 0.28 | 0.53 | **0.0000** | 6148 | 116 | 113 | 16 | 1.85 | 12.40 |
| co | Subtract x | worry+dysph | dysph | 0.17 | 0.09 | 0.24 | **0.0000** | 4927 | 273 | 628 | 66 | 5.25 | 9.51 |
| co | Subtract x | worry+worth | worth | 0.21 | 0.12 | 0.29 | **0.0000** | 4988 | 176 | 662 | 53 | 3.41 | 7.41 |
| co | Subtract x | worry+attend | attend | 0.32 | 0.27 | 0.36 | **0.0000** | 2694 | 455 | 1200 | 504 | 14.45 | 29.58 |
| co | Subtract x | worry+hyper | hyper | 0.34 | 0.28 | 0.39 | **0.0000** | 3726 | 277 | 958 | 215 | 6.92 | 18.33 |
| co | Subtract x | worry+argues | argues | 0.20 | 0.15 | 0.25 | **0.0000** | 2880 | 583 | 1194 | 432 | 16.84 | 26.57 |
| co | Subtract x | worry+temper | temper | 0.29 | 0.22 | 0.35 | **0.0000** | 4187 | 244 | 906 | 143 | 5.51 | 13.63 |
| co | Subtract x | worry+fights | fights | 0.43 | 0.32 | 0.53 | **0.0000** | 5109 | 74 | 289 | 29 | 1.43 | 9.12 |
| co | Subtract x | worry+steals | steals | 0.29 | 0.15 | 0.43 | **0.0000** | 5242 | 88 | 185 | 13 | 1.65 | 6.57 |
| co | Subtract x | dysph+worth | worth | 0.29 | 0.21 | 0.37 | **0.0000** | 6513 | 262 | 416 | 56 | 3.87 | 11.86 |
| co | Subtract x | dysph+attend | attend | 0.39 | 0.33 | 0.44 | **0.0000** | 3412 | 615 | 453 | 274 | 15.27 | 37.69 |
| co | Subtract x | dysph+hyper | hyper | 0.41 | 0.35 | 0.48 | **0.0000** | 4801 | 393 | 373 | 136 | 7.57 | 26.72 |
| co | Subtract x | dysph+argues | argues | 0.24 | 0.18 | 0.29 | **0.0000** | 3575 | 834 | 482 | 235 | 18.92 | 32.78 |
| co | Subtract x | dysph+temper | temper | 0.35 | 0.28 | 0.41 | **0.0000** | 5417 | 367 | 439 | 108 | 6.35 | 19.74 |
| co | Subtract x | dysph+fights | fights | 0.49 | 0.39 | 0.59 | **0.0000** | 6833 | 102 | 186 | 29 | 1.47 | 13.49 |
| co | Subtract x | dysph+steals | steals | 0.40 | 0.27 | 0.53 | **0.0000** | 6998 | 107 | 105 | 13 | 1.51 | 11.02 |
| co | Subtract x | worth+attend | attend | 0.38 | 0.33 | 0.43 | **0.0000** | 3335 | 705 | 475 | 322 | 17.45 | 40.40 |
| co | Subtract x | worth+hyper | hyper | 0.44 | 0.38 | 0.50 | **0.0000** | 4748 | 436 | 389 | 168 | 8.41 | 30.16 |
| co | Subtract x | worth+argues | argues | 0.26 | 0.21 | 0.32 | **0.0000** | 3442 | 908 | 449 | 266 | 20.87 | 37.20 |
| co | Subtract x | worth+temper | temper | 0.36 | 0.30 | 0.42 | **0.0000** | 5316 | 395 | 416 | 116 | 6.92 | 21.80 |
| co | Subtract x | worth+fights | fights | 0.44 | 0.32 | 0.55 | **0.0000** | 6738 | 104 | 159 | 21 | 1.52 | 11.67 |
| co | Subtract x | worth+steals | steals | 0.48 | 0.37 | 0.59 | **0.0000** | 6937 | 116 | 104 | 20 | 1.64 | 16.13 |
| co | Subtract x | attend+hyper | hyper | 0.08 | -.01 | 0.16 | 0.0971 | 3858 | 74 | 2250 | 58 | 1.88 | 2.51 |
| co | Subtract x | attend+argues | argues | -.05 | -.10 | 0.00 | 0.0715 | 2572 | 428 | 2071 | 298 | 14.27 | 12.58 |
| co | Subtract x | attend+temper | temper | 0.08 | -.00 | 0.16 | 0.0595 | 3497 | 145 | 1390 | 76 | 3.98 | 5.18 |
| co | Subtract x | attend+fights | fights | 0.21 | 0.07 | 0.36 | **0.0045** | 4104 | 38 | 471 | 12 | 0.92 | 2.48 |
| co | Subtract x | attend+steals | steals | 0.21 | 0.01 | 0.41 | **0.0357** | 4201 | 23 | 293 | 5 | 0.54 | 1.68 |
| co | Subtract x | hyper+argues | argues | 0.18 | 0.14 | 0.23 | **0.0000** | 3029 | 698 | 1233 | 478 | 18.73 | 27.94 |
| co | Subtract x | hyper+temper | temper | 0.29 | 0.23 | 0.36 | **0.0000** | 4372 | 270 | 924 | 157 | 5.82 | 14.52 |
| co | Subtract x | hyper+fights | fights | 0.41 | 0.30 | 0.52 | **0.0000** | 5365 | 59 | 354 | 26 | 1.09 | 6.84 |
| co | Subtract x | hyper+steals | steals | 0.39 | 0.26 | 0.53 | **0.0000** | 5494 | 49 | 218 | 14 | 0.88 | 6.03 |
| co | Subtract x | argues+temper | temper | 0.27 | 0.19 | 0.35 | **0.0000** | 4173 | 75 | 1597 | 83 | 1.77 | 4.94 |
| co | Subtract x | argues+fights | fights | 0.27 | 0.09 | 0.44 | **0.0028** | 4563 | 18 | 522 | 8 | 0.39 | 1.51 |
| co | Subtract x | argues+steals | steals | 0.31 | 0.13 | 0.49 | **0.0008** | 4584 | 23 | 282 | 7 | 0.50 | 2.42 |
| co | Subtract x | temper+fights | fights | 0.44 | 0.34 | 0.55 | **0.0000** | 6010 | 47 | 434 | 27 | 0.78 | 5.86 |
| co | Subtract x | temper+steals | steals | 0.41 | 0.28 | 0.53 | **0.0000** | 6012 | 68 | 200 | 17 | 1.12 | 7.83 |
| co | Subtract x | fights+steals | steals | 0.49 | 0.38 | 0.59 | **0.0000** | 7324 | 142 | 98 | 22 | 1.90 | 18.33 |
| bn | Subtract y | worry+fear | fear | 0.27 | 0.20 | 0.33 | **0.0000** | 4795 | 181 | 1203 | 119 | 3.64 | 9.00 |
| bn | Subtract y | dysph+fear | fear | 0.25 | 0.18 | 0.32 | **0.0000** | 5581 | 442 | 426 | 88 | 7.34 | 17.12 |
| bn | Subtract y | worth+fear | fear | 0.27 | 0.20 | 0.33 | **0.0000** | 5472 | 525 | 435 | 111 | 8.75 | 20.33 |
| bn | Subtract y | attend+fear | fear | -.01 | -.08 | 0.06 | 0.7988 | 3422 | 256 | 1147 | 83 | 6.96 | 6.75 |
| bn | Subtract y | hyper+fear | fear | 0.19 | 0.13 | 0.25 | **0.0000** | 4290 | 429 | 744 | 142 | 9.09 | 16.03 |
| bn | Subtract y | argues+fear | fear | 0.15 | 0.08 | 0.22 | **0.0000** | 3699 | 259 | 1001 | 117 | 6.54 | 10.47 |
| bn | Subtract y | temper+fear | fear | 0.25 | 0.19 | 0.31 | **0.0000** | 4720 | 452 | 643 | 144 | 8.74 | 18.30 |
| bn | Subtract y | fights+fear | fear | 0.31 | 0.23 | 0.38 | **0.0000** | 5387 | 724 | 168 | 75 | 11.85 | 30.86 |
| bn | Subtract y | steals+fear | fear | 0.21 | 0.11 | 0.31 | **0.0000** | 5496 | 768 | 96 | 33 | 12.26 | 25.58 |
| bn | Subtract y | dysph+worry | worry | 0.22 | 0.16 | 0.28 | **0.0000** | 4536 | 664 | 532 | 162 | 12.77 | 23.34 |
| bn | Subtract y | worth+worry | worry | 0.22 | 0.17 | 0.28 | **0.0000** | 4411 | 753 | 528 | 187 | 14.58 | 26.15 |
| bn | Subtract y | attend+worry | worry | -.11 | -.18 | -.05 | **0.0002** | 2764 | 385 | 1554 | 150 | 12.23 | 8.80 |
| bn | Subtract y | hyper+worry | worry | 0.09 | 0.04 | 0.15 | **0.0008** | 3364 | 639 | 936 | 237 | 15.96 | 20.20 |
| bn | Subtract y | argues+worry | worry | 0.06 | 0.01 | 0.12 | **0.0314** | 3068 | 395 | 1406 | 220 | 11.41 | 13.53 |
| bn | Subtract y | temper+worry | worry | 0.20 | 0.15 | 0.26 | **0.0000** | 3760 | 671 | 787 | 262 | 15.14 | 24.98 |
| bn | Subtract y | fights+worry | worry | 0.27 | 0.20 | 0.34 | **0.0000** | 4225 | 958 | 200 | 118 | 18.48 | 37.11 |
| bn | Subtract y | steals+worry | worry | 0.17 | 0.08 | 0.25 | **0.0001** | 4335 | 995 | 138 | 60 | 18.67 | 30.30 |
| bn | Subtract y | worth+dysph | dysph | 0.19 | 0.12 | 0.27 | **0.0000** | 6231 | 544 | 398 | 74 | 8.03 | 15.68 |
| bn | Subtract y | attend+dysph | dysph | -.03 | -.11 | 0.05 | 0.4887 | 3744 | 283 | 681 | 46 | 7.03 | 6.33 |
| bn | Subtract y | hyper+dysph | dysph | 0.08 | 0.01 | 0.16 | **0.0280** | 4640 | 554 | 438 | 71 | 10.67 | 13.95 |
| bn | Subtract y | argues+dysph | dysph | 0.09 | 0.01 | 0.17 | **0.0225** | 4137 | 272 | 656 | 61 | 6.17 | 8.51 |
| bn | Subtract y | temper+dysph | dysph | 0.13 | 0.06 | 0.20 | **0.0004** | 5220 | 564 | 466 | 81 | 9.75 | 14.81 |
| bn | Subtract y | fights+dysph | dysph | 0.26 | 0.19 | 0.34 | **0.0000** | 6026 | 909 | 150 | 65 | 13.11 | 30.23 |
| bn | Subtract y | steals+dysph | dysph | 0.15 | 0.05 | 0.26 | **0.0031** | 6183 | 922 | 91 | 27 | 12.98 | 22.88 |
| bn | Subtract y | attend+worth | worth | 0.03 | -.07 | 0.12 | 0.5864 | 3894 | 146 | 765 | 32 | 3.61 | 4.02 |
| bn | Subtract y | hyper+worth | worth | 0.20 | 0.12 | 0.27 | **0.0000** | 4870 | 314 | 490 | 67 | 6.06 | 12.03 |
| bn | Subtract y | argues+worth | worth | 0.20 | 0.11 | 0.28 | **0.0000** | 4200 | 150 | 664 | 51 | 3.45 | 7.13 |
| bn | Subtract y | temper+worth | worth | 0.30 | 0.23 | 0.37 | **0.0000** | 5385 | 326 | 447 | 85 | 5.71 | 15.98 |
| bn | Subtract y | fights+worth | worth | 0.34 | 0.26 | 0.43 | **0.0000** | 6275 | 567 | 130 | 50 | 8.29 | 27.78 |
| bn | Subtract y | steals+worth | worth | 0.26 | 0.16 | 0.36 | **0.0000** | 6470 | 583 | 96 | 28 | 8.27 | 22.58 |
| bn | Subtract y | hyper+attend | attend | 0.25 | 0.21 | 0.29 | **0.0000** | 3336 | 596 | 1693 | 615 | 15.16 | 26.65 |
| bn | Subtract y | argues+attend | attend | 0.07 | 0.02 | 0.12 | **0.0104** | 2630 | 370 | 2020 | 349 | 12.33 | 14.73 |
| bn | Subtract y | temper+attend | attend | 0.34 | 0.30 | 0.39 | **0.0000** | 3041 | 601 | 955 | 511 | 16.50 | 34.86 |
| bn | Subtract y | fights+attend | attend | 0.47 | 0.42 | 0.52 | **0.0000** | 3317 | 825 | 223 | 260 | 19.92 | 53.83 |
| bn | Subtract y | steals+attend | attend | 0.41 | 0.34 | 0.47 | **0.0000** | 3386 | 838 | 147 | 151 | 19.84 | 50.67 |
| bn | Subtract y | argues+hyper | hyper | 0.24 | 0.17 | 0.30 | **0.0000** | 3559 | 168 | 1547 | 164 | 4.51 | 9.59 |
| bn | Subtract y | temper+hyper | hyper | 0.42 | 0.37 | 0.47 | **0.0000** | 4308 | 334 | 826 | 255 | 7.20 | 23.59 |
| bn | Subtract y | fights+hyper | hyper | 0.51 | 0.45 | 0.56 | **0.0000** | 4908 | 516 | 226 | 154 | 9.51 | 40.53 |
| bn | Subtract y | steals+hyper | hyper | 0.46 | 0.39 | 0.53 | **0.0000** | 5007 | 536 | 141 | 91 | 9.67 | 39.22 |
| bn | Subtract y | temper+argues | argues | 0.25 | 0.21 | 0.30 | **0.0000** | 3453 | 795 | 1140 | 540 | 18.71 | 32.14 |
| bn | Subtract y | fights+argues | argues | 0.38 | 0.32 | 0.43 | **0.0000** | 3500 | 1081 | 259 | 271 | 23.60 | 51.13 |
| bn | Subtract y | steals+argues | argues | 0.35 | 0.28 | 0.42 | **0.0000** | 3523 | 1084 | 141 | 148 | 23.53 | 51.21 |
| bn | Subtract y | fights+temper | temper | 0.45 | 0.40 | 0.51 | **0.0000** | 5556 | 501 | 312 | 149 | 8.27 | 32.32 |
| bn | Subtract y | steals+temper | temper | 0.39 | 0.32 | 0.47 | **0.0000** | 5573 | 507 | 150 | 67 | 8.34 | 30.88 |
| bn | Subtract y | steals+fights | fights | 0.40 | 0.28 | 0.52 | **0.0000** | 7322 | 144 | 104 | 16 | 1.93 | 13.33 |

| Supplemental Table S3L. Tetrachoric correlations from 4 x 4 pairwise analyses of all 90 combinations of the 10 selected psychological problems with every other problem to quantify the associations of each baseline problem with outcomes in the **third annual follow-up** that define four parsed paths traditionally conflated in estimates of change: ***add path*** (x_1_ at baseline and x_2_ + y_2_ at follow-up); ***joint persist path*** (x_1_ + y_1_ at baseline and x_2_ + y_2_ at follow-up); ***shift path*** (only x_1_ at baseline and only y_2_ at follow-up); ***subtract x path*** (x_1_ + y_1_ at baseline and only y_2_ at follow-up); and ***subtract y path*** (x_1_ + y_1_ at baseline and only x_2_ at follow-up). Results are for problems dichotomized at the **high rating cut (0 or 1 vs 2).** r_t_ = tetrachoric correlation. –CL and +CL = lower and upper 95% confidence intervals for the tetrachoric correlation. c00 = number without the predictor or the outcome; c01 = number without predictor with the outcome; c10 = number with the predictor without the outcome; c11 = number with the predictor and the outcome. Base% = percent of the outcome only x_2_ among children with neither x_1_ nor the other member of the pair, y_1_, at baseline. Obs% = Observed percent of outcomes on the indicated path. Raw P values significant after FDR adjustment in **bold**. | | | | | | | | | | | | | |
| --- | --- | --- | --- | --- | --- | --- | --- | --- | --- | --- | --- | --- | --- |
| prefix | Parsed paths | Predictor | Outcome | r_t_ | -CL | +CL | P | c00 | c01 | c10 | c11 | Base% | Obs% |
| dh | Add | fear | fear+worry | 0.42 | 0.28 | 0.57 | **0.0000** | 7785 | 88 | 73 | 9 | 1.12 | 10.98 |
| dl | Add | worry | worry+fear | 0.40 | 0.28 | 0.51 | **0.0000** | 7785 | 88 | 212 | 18 | 1.12 | 7.83 |
| dh | Add | fear | fear+dysph | 0.30 | 0.11 | 0.48 | **0.0015** | 8026 | 39 | 179 | 5 | 0.48 | 2.72 |
| dl | Add | dysph | dysph+fear | 0.44 | 0.22 | 0.66 | **0.0001** | 8026 | 39 | 35 | 3 | 0.48 | 7.89 |
| dh | Add | fear | fear+worth | 0.41 | 0.24 | 0.57 | **0.0000** | 8022 | 32 | 167 | 7 | 0.40 | 4.02 |
| dl | Add | worth | worth+fear | 0.54 | 0.35 | 0.72 | **0.0000** | 8022 | 32 | 43 | 5 | 0.40 | 10.42 |
| dh | Add | fear | fear+attend | 0.43 | 0.26 | 0.59 | **0.0000** | 7171 | 42 | 102 | 7 | 0.58 | 6.42 |
| dl | Add | attend | attend+fear | 0.40 | 0.31 | 0.50 | **0.0000** | 7171 | 42 | 857 | 33 | 0.58 | 3.71 |
| dh | Add | fear | fear+hyper | 0.35 | 0.13 | 0.58 | **0.0023** | 7591 | 19 | 129 | 3 | 0.25 | 2.27 |
| dl | Add | hyper | hyper+fear | 0.39 | 0.24 | 0.55 | **0.0000** | 7591 | 19 | 483 | 10 | 0.25 | 2.03 |
| dh | Add | fear | fear+argues | 0.36 | 0.17 | 0.54 | **0.0002** | 7418 | 36 | 124 | 5 | 0.48 | 3.88 |
| dl | Add | argues | argues+fear | 0.42 | 0.31 | 0.53 | **0.0000** | 7418 | 36 | 625 | 24 | 0.48 | 3.70 |
| dh | Add | fear | fear+temper | 0.13 | -.18 | 0.44 | **0.4138** | 7818 | 21 | 149 | 1 | 0.27 | 0.67 |
| dl | Add | temper | temper+fear | 0.44 | 0.27 | 0.60 | **0.0000** | 7818 | 21 | 256 | 8 | 0.27 | 3.03 |
| dh | Add | fear | fear+fights | . | . | . | . | 8069 | 4 | 200 | 0 | 0.05 | 0.00 |
| dl | Add | fights | fights+fear | . | . | . | . | 8069 | 4 | 30 | 0 | 0.05 | 0.00 |
| dh | Add | fear | fear+steals | 0.43 | 0.05 | 0.82 | **0.0282** | 8072 | 2 | 201 | 1 | 0.02 | 0.50 |
| dl | Add | steals | steals+fear | 0.76 | 0.52 | 0.99 | **0.0000** | 8072 | 2 | 27 | 2 | 0.02 | 6.90 |
| dh | Add | worry | worry+dysph | 0.47 | 0.35 | 0.59 | **0.0000** | 7886 | 41 | 304 | 18 | 0.52 | 5.59 |
| dl | Add | dysph | dysph+worry | 0.54 | 0.34 | 0.74 | **0.0000** | 7886 | 41 | 24 | 4 | 0.52 | 14.29 |
| dh | Add | worry | worry+worth | 0.51 | 0.39 | 0.64 | **0.0000** | 7889 | 28 | 295 | 16 | 0.35 | 5.14 |
| dl | Add | worth | worth+worry | 0.54 | 0.34 | 0.74 | **0.0000** | 7889 | 28 | 34 | 4 | 0.35 | 10.53 |
| dh | Add | worry | worry+attend | 0.49 | 0.37 | 0.61 | **0.0000** | 7051 | 50 | 203 | 18 | 0.70 | 8.14 |
| dl | Add | attend | attend+worry | 0.35 | 0.25 | 0.46 | **0.0000** | 7051 | 50 | 824 | 30 | 0.70 | 3.51 |
| dh | Add | worry | worry+hyper | 0.43 | 0.27 | 0.60 | **0.0000** | 7451 | 20 | 263 | 8 | 0.27 | 2.95 |
| dl | Add | hyper | hyper+worry | 0.41 | 0.26 | 0.55 | **0.0000** | 7451 | 20 | 473 | 11 | 0.27 | 2.27 |
| dh | Add | worry | worry+argues | 0.46 | 0.33 | 0.60 | **0.0000** | 7301 | 34 | 235 | 13 | 0.46 | 5.24 |
| dl | Add | argues | argues+worry | 0.49 | 0.39 | 0.59 | **0.0000** | 7301 | 34 | 590 | 30 | 0.46 | 4.84 |
| dh | Add | worry | worry+temper | 0.40 | 0.22 | 0.59 | **0.0000** | 7687 | 16 | 280 | 6 | 0.21 | 2.10 |
| dl | Add | temper | temper+worry | 0.48 | 0.32 | 0.65 | **0.0000** | 7687 | 16 | 244 | 8 | 0.21 | 3.17 |
| dh | Add | worry | worry+fights | 0.43 | 0.13 | 0.73 | **0.0050** | 7926 | 3 | 342 | 2 | 0.04 | 0.58 |
| dl | Add | fights | fights+worry | . | . | . | . | 7926 | 3 | 26 | 0 | 0.04 | 0.00 |
| dh | Add | worry | worry+steals | 0.46 | 0.04 | 0.88 | **0.0300** | 7927 | 1 | 347 | 1 | 0.01 | 0.29 |
| dl | Add | steals | steals+worry | 0.82 | 0.60 | 1.00 | **0.0000** | 7927 | 1 | 25 | 2 | 0.01 | 7.41 |
| dh | Add | dysph | dysph+worth | 0.36 | 0.10 | 0.62 | **0.0060** | 8151 | 36 | 39 | 2 | 0.44 | 4.88 |
| dl | Add | worth | worth+dysph | 0.38 | 0.16 | 0.60 | **0.0008** | 8151 | 36 | 58 | 3 | 0.44 | 4.92 |
| dh | Add | dysph | dysph+attend | 0.44 | 0.18 | 0.71 | **0.0012** | 7265 | 32 | 23 | 2 | 0.44 | 8.00 |
| dl | Add | attend | attend+dysph | 0.30 | 0.18 | 0.43 | **0.0000** | 7265 | 32 | 934 | 18 | 0.44 | 1.89 |
| dh | Add | dysph | dysph+hyper | 0.35 | 0.01 | 0.69 | 0.0463 | 7686 | 16 | 39 | 1 | 0.21 | 2.50 |
| dl | Add | hyper | hyper+dysph | 0.22 | 0.01 | 0.43 | **0.0392** | 7686 | 16 | 543 | 4 | 0.21 | 0.73 |
| dh | Add | dysph | dysph+argues | . | . | . | . | 7525 | 34 | 24 | 0 | 0.45 | 0.00 |
| dl | Add | argues | argues+dysph | 0.38 | 0.26 | 0.50 | **0.0000** | 7525 | 34 | 670 | 20 | 0.45 | 2.90 |
| dh | Add | dysph | dysph+temper | 0.39 | 0.05 | 0.73 | **0.0251** | 7944 | 16 | 28 | 1 | 0.20 | 3.45 |
| dl | Add | temper | temper+dysph | 0.33 | 0.12 | 0.53 | **0.0022** | 7944 | 16 | 285 | 4 | 0.20 | 1.38 |
| dh | Add | dysph | dysph+fights | . | . | . | . | 8218 | 1 | 54 | 0 | 0.01 | 0.00 |
| dl | Add | fights | fights+dysph | 0.72 | 0.38 | 1.00 | **0.0000** | 8218 | 1 | 29 | 1 | 0.01 | 3.33 |
| dh | Add | dysph | dysph+steals | 0.53 | 0.17 | 0.89 | **0.0039** | 8218 | 3 | 54 | 1 | 0.04 | 1.82 |
| dl | Add | steals | steals+dysph | . | . | . | . | 8218 | 3 | 28 | 0 | 0.04 | 0.00 |
| dh | Add | worth | worth+attend | 0.42 | 0.15 | 0.68 | **0.0020** | 7260 | 27 | 32 | 2 | 0.37 | 5.88 |
| dl | Add | attend | attend+worth | 0.32 | 0.20 | 0.45 | **0.0000** | 7260 | 27 | 924 | 17 | 0.37 | 1.81 |
| dh | Add | worth | worth+hyper | 0.46 | 0.19 | 0.72 | **0.0009** | 7672 | 13 | 54 | 2 | 0.17 | 3.57 |
| dl | Add | hyper | hyper+worth | 0.34 | 0.15 | 0.52 | **0.0004** | 7672 | 13 | 537 | 6 | 0.17 | 1.10 |
| dh | Add | worth | worth+argues | 0.34 | -.00 | 0.68 | **0.0527** | 7525 | 17 | 39 | 1 | 0.23 | 2.50 |
| dl | Add | argues | argues+worth | 0.49 | 0.37 | 0.61 | **0.0000** | 7525 | 17 | 667 | 19 | 0.23 | 2.77 |
| dh | Add | worth | worth+temper | 0.40 | 0.05 | 0.75 | **0.0236** | 7931 | 9 | 47 | 1 | 0.11 | 2.08 |
| dl | Add | temper | temper+worth | 0.53 | 0.35 | 0.70 | **0.0000** | 7931 | 9 | 281 | 7 | 0.11 | 2.43 |
| dh | Add | worth | worth+fights | . | . | . | . | 8194 | 3 | 75 | 0 | 0.04 | 0.00 |
| dl | Add | fights | fights+worth | . | . | . | . | 8194 | 3 | 31 | 0 | 0.04 | 0.00 |
| dh | Add | worth | worth+steals | . | . | . | . | 8197 | 3 | 75 | 0 | 0.04 | 0.00 |
| dl | Add | steals | steals+worth | . | . | . | . | 8197 | 3 | 28 | 0 | 0.04 | 0.00 |
| dh | Add | attend | attend+hyper | 0.44 | 0.35 | 0.52 | **0.0000** | 7083 | 98 | 512 | 49 | 1.36 | 8.73 |
| dl | Add | hyper | hyper+attend | 0.48 | 0.36 | 0.59 | **0.0000** | 7083 | 98 | 122 | 19 | 1.36 | 13.48 |
| dh | Add | attend | attend+argues | 0.49 | 0.41 | 0.56 | **0.0000** | 6805 | 86 | 627 | 65 | 1.25 | 9.39 |
| dl | Add | argues | argues+attend | 0.48 | 0.39 | 0.56 | **0.0000** | 6805 | 86 | 388 | 43 | 1.25 | 9.98 |
| dh | Add | attend | attend+temper | 0.40 | 0.30 | 0.51 | **0.0000** | 7114 | 36 | 811 | 28 | 0.50 | 3.34 |
| dl | Add | temper | temper+attend | 0.56 | 0.44 | 0.68 | **0.0000** | 7114 | 36 | 156 | 16 | 0.50 | 9.30 |
| dh | Add | attend | attend+fights | 0.30 | 0.09 | 0.52 | **0.0053** | 7302 | 7 | 959 | 5 | 0.10 | 0.52 |
| dl | Add | fights | fights+attend | 0.59 | 0.24 | 0.94 | **0.0009** | 7302 | 7 | 12 | 1 | 0.10 | 7.69 |
| dh | Add | attend | attend+steals | 0.51 | 0.35 | 0.67 | **0.0000** | 7305 | 5 | 955 | 11 | 0.07 | 1.14 |
| dl | Add | steals | steals+attend | 0.64 | 0.29 | 0.98 | **0.0003** | 7305 | 5 | 11 | 1 | 0.07 | 8.33 |
| dh | Add | hyper | hyper+argues | 0.47 | 0.36 | 0.57 | **0.0000** | 7172 | 56 | 330 | 25 | 0.77 | 7.04 |
| dl | Add | argues | argues+hyper | 0.38 | 0.27 | 0.49 | **0.0000** | 7172 | 56 | 490 | 24 | 0.77 | 4.67 |
| dh | Add | hyper | hyper+temper | 0.48 | 0.34 | 0.62 | **0.0000** | 7517 | 17 | 442 | 13 | 0.23 | 2.86 |
| dl | Add | temper | temper+hyper | 0.55 | 0.40 | 0.69 | **0.0000** | 7517 | 17 | 198 | 10 | 0.23 | 4.81 |
| dh | Add | hyper | hyper+fights | 0.28 | -.01 | 0.57 | 0.0593 | 7716 | 5 | 550 | 2 | 0.06 | 0.36 |
| dl | Add | fights | fights+hyper | . | . | . | . | 7716 | 5 | 21 | 0 | 0.06 | 0.00 |
| dh | Add | hyper | hyper+steals | 0.46 | 0.26 | 0.66 | **0.0000** | 7717 | 6 | 547 | 6 | 0.08 | 1.08 |
| dl | Add | steals | steals+hyper | 0.57 | 0.22 | 0.91 | **0.0015** | 7717 | 6 | 18 | 1 | 0.08 | 5.26 |
| dh | Add | argues | argues+temper | 0.45 | 0.36 | 0.55 | **0.0000** | 7396 | 65 | 492 | 36 | 0.87 | 6.82 |
| dl | Add | temper | temper+argues | 0.50 | 0.37 | 0.63 | **0.0000** | 7396 | 65 | 108 | 14 | 0.87 | 11.48 |
| dh | Add | argues | argues+fights | 0.52 | 0.36 | 0.68 | **0.0000** | 7568 | 6 | 689 | 10 | 0.08 | 1.43 |
| dl | Add | fights | fights+argues | 0.65 | 0.31 | 0.99 | **0.0002** | 7568 | 6 | 8 | 1 | 0.08 | 11.11 |
| dh | Add | argues | argues+steals | 0.54 | 0.40 | 0.68 | **0.0000** | 7562 | 8 | 692 | 14 | 0.11 | 1.98 |
| dl | Add | steals | steals+argues | 0.58 | 0.23 | 0.92 | **0.0012** | 7562 | 8 | 12 | 1 | 0.11 | 7.69 |
| dh | Add | temper | temper+fights | 0.48 | 0.26 | 0.70 | **0.0000** | 7969 | 6 | 294 | 4 | 0.08 | 1.34 |
| dl | Add | fights | fights+temper | . | . | . | . | 7969 | 6 | 14 | 0 | 0.08 | 0.00 |
| dh | Add | temper | temper+steals | 0.51 | 0.31 | 0.71 | **0.0000** | 7961 | 6 | 304 | 5 | 0.08 | 1.62 |
| dl | Add | steals | steals+temper | 0.55 | 0.20 | 0.90 | **0.0021** | 7961 | 6 | 21 | 1 | 0.08 | 4.55 |
| dh | Add | fights | fights+steals | 0.72 | 0.38 | 1.00 | **0.0000** | 8244 | 1 | 30 | 1 | 0.01 | 3.23 |
| dl | Add | steals | steals+fights | 0.72 | 0.39 | 1.00 | **0.0000** | 8244 | 1 | 27 | 1 | 0.01 | 3.57 |
| dp | Joint Persist | fear+worry | fear+worry | 0.66 | 0.57 | 0.74 | **0.0000** | 7785 | 88 | 91 | 31 | 1.12 | 25.41 |
| dp | Joint Persist | fear+dysph | fear+dysph | 0.65 | 0.47 | 0.83 | **0.0000** | 8026 | 39 | 15 | 5 | 0.48 | 25.00 |
| dp | Joint Persist | fear+worth | fear+worth | 0.42 | 0.16 | 0.69 | **0.0016** | 8022 | 32 | 28 | 2 | 0.40 | 6.67 |
| dp | Joint Persist | fear+attend | fear+attend | 0.74 | 0.66 | 0.83 | **0.0000** | 7171 | 42 | 70 | 25 | 0.58 | 26.32 |
| dp | Joint Persist | fear+hyper | fear+hyper | 0.67 | 0.53 | 0.81 | **0.0000** | 7591 | 19 | 63 | 9 | 0.25 | 12.50 |
| dp | Joint Persist | fear+argues | fear+argues | 0.67 | 0.56 | 0.79 | **0.0000** | 7418 | 36 | 61 | 14 | 0.48 | 18.67 |
| dp | Joint Persist | fear+temper | fear+temper | 0.67 | 0.53 | 0.82 | **0.0000** | 7818 | 21 | 46 | 8 | 0.27 | 14.81 |
| dp | Joint Persist | fear+fights | fear+fights | . | . | . | . | 8069 | 4 | 4 | 0 | 0.05 | 0.00 |
| dp | Joint Persist | fear+steals | fear+steals | 0.91 | 0.70 | 1.00 | **0.0000** | 8072 | 2 | 1 | 1 | 0.02 | 50.00 |
| dp | Joint Persist | worry+dysph | worry+dysph | 0.58 | 0.40 | 0.76 | **0.0000** | 7886 | 41 | 25 | 5 | 0.52 | 16.67 |
| dp | Joint Persist | worry+worth | worry+worth | 0.62 | 0.46 | 0.79 | **0.0000** | 7889 | 28 | 34 | 6 | 0.35 | 15.00 |
| dp | Joint Persist | worry+attend | worry+attend | 0.74 | 0.66 | 0.81 | **0.0000** | 7051 | 50 | 97 | 34 | 0.70 | 25.95 |
| dp | Joint Persist | worry+hyper | worry+hyper | 0.73 | 0.62 | 0.84 | **0.0000** | 7451 | 20 | 67 | 14 | 0.27 | 17.28 |
| dp | Joint Persist | worry+argues | worry+argues | 0.75 | 0.66 | 0.83 | **0.0000** | 7301 | 34 | 80 | 24 | 0.46 | 23.08 |
| dp | Joint Persist | worry+temper | worry+temper | 0.72 | 0.60 | 0.85 | **0.0000** | 7687 | 16 | 56 | 10 | 0.21 | 15.15 |
| dp | Joint Persist | worry+fights | worry+fights | . | . | . | . | 7926 | 3 | 8 | 0 | 0.04 | 0.00 |
| dp | Joint Persist | worry+steals | worry+steals | 0.95 | 0.85 | 1.00 | **0.0000** | 7927 | 1 | 2 | 2 | 0.01 | 50.00 |
| dp | Joint Persist | dysph+worth | dysph+worth | 0.58 | 0.35 | 0.80 | **0.0000** | 8151 | 36 | 14 | 3 | 0.44 | 17.65 |
| dp | Joint Persist | dysph+attend | dysph+attend | 0.63 | 0.46 | 0.80 | **0.0000** | 7265 | 32 | 27 | 6 | 0.44 | 18.18 |
| dp | Joint Persist | dysph+hyper | dysph+hyper | 0.58 | 0.32 | 0.84 | **0.0000** | 7686 | 16 | 16 | 2 | 0.21 | 11.11 |
| dp | Joint Persist | dysph+argues | dysph+argues | 0.71 | 0.58 | 0.84 | **0.0000** | 7525 | 34 | 25 | 9 | 0.45 | 26.47 |
| dp | Joint Persist | dysph+temper | dysph+temper | 0.60 | 0.38 | 0.82 | **0.0000** | 7944 | 16 | 26 | 3 | 0.20 | 10.34 |
| dp | Joint Persist | dysph+fights | dysph+fights | . | . | . | . | 8218 | 1 | 4 | 0 | 0.01 | 0.00 |
| dp | Joint Persist | dysph+steals | dysph+steals | 0.84 | 0.57 | 1.00 | **0.0000** | 8218 | 3 | 2 | 1 | 0.04 | 33.33 |
| dp | Joint Persist | worth+attend | worth+attend | 0.46 | 0.23 | 0.69 | **0.0001** | 7260 | 27 | 41 | 3 | 0.37 | 6.82 |
| dp | Joint Persist | worth+hyper | worth+hyper | . | . | . | . | 7672 | 13 | 22 | 0 | 0.17 | 0.00 |
| dp | Joint Persist | worth+argues | worth+argues | 0.61 | 0.41 | 0.80 | **0.0000** | 7525 | 17 | 34 | 4 | 0.23 | 10.53 |
| dp | Joint Persist | worth+temper | worth+temper | 0.46 | 0.11 | 0.81 | **0.0096** | 7931 | 9 | 29 | 1 | 0.11 | 3.33 |
| dp | Joint Persist | worth+fights | worth+fights | . | . | . | . | 8194 | 3 | 3 | 0 | 0.04 | 0.00 |
| dp | Joint Persist | worth+steals | worth+steals | . | . | . | . | 8197 | 3 | 3 | 0 | 0.04 | 0.00 |
| dp | Joint Persist | attend+hyper | attend+hyper | 0.77 | 0.72 | 0.81 | **0.0000** | 7083 | 98 | 290 | 134 | 1.36 | 31.60 |
| dp | Joint Persist | attend+argues | attend+argues | 0.78 | 0.73 | 0.83 | **0.0000** | 6805 | 86 | 194 | 99 | 1.25 | 33.79 |
| dp | Joint Persist | attend+temper | attend+temper | 0.68 | 0.59 | 0.78 | **0.0000** | 7114 | 36 | 122 | 24 | 0.50 | 16.44 |
| dp | Joint Persist | attend+fights | attend+fights | 0.73 | 0.53 | 0.93 | **0.0000** | 7302 | 7 | 18 | 3 | 0.10 | 14.29 |
| dp | Joint Persist | attend+steals | attend+steals | 0.82 | 0.66 | 0.98 | **0.0000** | 7305 | 5 | 15 | 4 | 0.07 | 21.05 |
| dp | Joint Persist | hyper+argues | hyper+argues | 0.75 | 0.68 | 0.81 | **0.0000** | 7172 | 56 | 157 | 53 | 0.77 | 25.24 |
| dp | Joint Persist | hyper+temper | hyper+temper | 0.77 | 0.68 | 0.86 | **0.0000** | 7517 | 17 | 91 | 19 | 0.23 | 17.27 |
| dp | Joint Persist | hyper+fights | hyper+fights | 0.63 | 0.29 | 0.97 | **0.0003** | 7716 | 5 | 12 | 1 | 0.06 | 7.69 |
| dp | Joint Persist | hyper+steals | hyper+steals | 0.74 | 0.50 | 0.98 | **0.0000** | 7717 | 6 | 10 | 2 | 0.08 | 16.67 |
| dp | Joint Persist | argues+temper | argues+temper | 0.70 | 0.63 | 0.77 | **0.0000** | 7396 | 65 | 151 | 45 | 0.87 | 22.96 |
| dp | Joint Persist | argues+fights | argues+fights | 0.73 | 0.52 | 0.93 | **0.0000** | 7568 | 6 | 22 | 3 | 0.08 | 12.00 |
| dp | Joint Persist | argues+steals | argues+steals | 0.74 | 0.53 | 0.94 | **0.0000** | 7562 | 8 | 15 | 3 | 0.11 | 16.67 |
| dp | Joint Persist | temper+fights | temper+fights | 0.80 | 0.64 | 0.96 | **0.0000** | 7969 | 6 | 16 | 4 | 0.08 | 20.00 |
| dp | Joint Persist | temper+steals | temper+steals | 0.65 | 0.32 | 0.99 | **0.0001** | 7961 | 6 | 8 | 1 | 0.08 | 11.11 |
| dp | Joint Persist | fights+steals | fights+steals | . | . | . | . | 8244 | 1 | 3 | 0 | 0.01 | 0.00 |
| cg | Shift | fear | worry | 0.22 | 0.04 | 0.40 | **0.0149** | 7734 | 139 | 77 | 5 | 1.77 | 6.10 |
| bj | Shift | worry | fear | 0.15 | -.02 | 0.32 | 0.0938 | 7799 | 74 | 225 | 5 | 0.94 | 2.17 |
| cg | Shift | fear | dysph | 0.20 | 0.02 | 0.37 | **0.0263** | 7995 | 70 | 179 | 5 | 0.87 | 2.72 |
| bj | Shift | dysph | fear | 0.31 | 0.11 | 0.51 | **0.0021** | 7926 | 139 | 34 | 4 | 1.72 | 10.53 |
| cg | Shift | fear | worth | 0.20 | -.01 | 0.41 | 0.0679 | 8013 | 41 | 171 | 3 | 0.51 | 1.72 |
| bj | Shift | worth | fear | 0.21 | -.01 | 0.42 | 0.0566 | 7909 | 145 | 45 | 3 | 1.80 | 6.25 |
| cg | Shift | fear | attend | 0.03 | -.12 | 0.19 | 0.6685 | 6880 | 333 | 103 | 6 | 4.62 | 5.50 |
| bj | Shift | attend | fear | -.06 | -.19 | 0.06 | 0.3274 | 7112 | 101 | 881 | 9 | 1.40 | 1.01 |
| cg | Shift | fear | hyper | 0.09 | -.08 | 0.25 | **0.3079** | 7432 | 178 | 127 | 5 | 2.34 | 3.79 |
| bj | Shift | hyper | fear | 0.13 | 0.02 | 0.25 | **0.0190** | 7471 | 139 | 476 | 17 | 1.83 | 3.45 |
| cg | Shift | fear | argues | 0.00 | -.14 | 0.15 | 0.9546 | 7058 | 396 | 122 | 7 | 5.31 | 5.43 |
| bj | Shift | argues | Fear | 0.08 | -.04 | 0.19 | 0.2049 | 7343 | 111 | 635 | 14 | 1.49 | 2.16 |
| cg | Shift | fear | Temper | 0.14 | -.03 | 0.31 | 0.0997 | 7723 | 116 | 145 | 5 | 1.48 | 3.33 |
| bj | Shift | temper | fear | 0.16 | 0.03 | 0.29 | **0.0158** | 7694 | 145 | 253 | 11 | 1.85 | 4.17 |
| cg | Shift | fear | fights | 0.12 | -.19 | 0.44 | 0.4385 | 8056 | 17 | 199 | 1 | 0.21 | 0.50 |
| bj | Shift | fights | fear | . | . | . | . | 7892 | 181 | 30 | 0 | 2.24 | 0.00 |
| cg | Shift | fear | steals | 0.17 | -.08 | 0.42 | 0.1801 | 8047 | 27 | 200 | 2 | 0.33 | 0.99 |
| bj | Shift | steals | fear | 0.06 | -.24 | 0.36 | 0.6744 | 7894 | 180 | 28 | 1 | 2.23 | 3.45 |
| cg | Shift | worry | dysph | 0.14 | -.03 | 0.31 | 0.1097 | 7872 | 55 | 317 | 5 | 0.69 | 1.55 |
| bj | Shift | dysph | worry | 0.06 | -.24 | 0.35 | 0.7175 | 7732 | 195 | 27 | 1 | 2.46 | 3.57 |
| cg | Shift | worry | worth | 0.18 | -.01 | 0.38 | 0.0656 | 7882 | 35 | 307 | 4 | 0.44 | 1.29 |
| bj | Shift | worth | worry | 0.19 | -.03 | 0.40 | 0.0835 | 7711 | 206 | 35 | 3 | 2.60 | 7.89 |
| cg | Shift | worry | attend | 0.03 | -.10 | 0.15 | 0.6595 | 6792 | 309 | 210 | 11 | 4.35 | 4.98 |
| bj | Shift | attend | worry | -.10 | -.21 | 0.01 | 0.0883 | 6951 | 150 | 843 | 11 | 2.11 | 1.29 |
| cg | Shift | worry | hyper | -.01 | -.15 | 0.14 | 0.9352 | 7300 | 171 | 265 | 6 | 2.29 | 2.21 |
| bj | Shift | hyper | worry | 0.04 | -.07 | 0.15 | 0.5242 | 7276 | 195 | 469 | 15 | 2.61 | 3.10 |
| cg | Shift | worry | argues | -.05 | -.17 | 0.07 | 0.3718 | 6948 | 387 | 238 | 10 | 5.28 | 4.03 |
| bj | Shift | argues | worry | 0.05 | -.06 | 0.16 | 0.3765 | 7175 | 160 | 603 | 17 | 2.18 | 2.74 |
| cg | Shift | worry | temper | 0.03 | -.13 | 0.19 | 0.7462 | 7587 | 116 | 281 | 5 | 1.51 | 1.75 |
| bj | Shift | temper | worry | 0.23 | 0.12 | 0.34 | **0.0000** | 7505 | 198 | 233 | 19 | 2.57 | 7.54 |
| cg | Shift | worry | fights | . | . | . | . | 7912 | 17 | 344 | 0 | 0.21 | 0.00 |
| bj | Shift | fights | worry | 0.16 | -.09 | 0.40 | 0.2178 | 7693 | 236 | 24 | 2 | 2.98 | 7.69 |
| cg | Shift | worry | steals | 0.21 | 0.02 | 0.41 | **0.0350** | 7902 | 26 | 344 | 4 | 0.33 | 1.15 |
| bj | Shift | steals | worry | 0.03 | -.26 | 0.33 | 0.8320 | 7691 | 237 | 26 | 1 | 2.99 | 3.70 |
| cg | Shift | dysph | worth | 0.21 | -.11 | 0.53 | 0.1916 | 8143 | 44 | 40 | 1 | 0.54 | 2.44 |
| bj | Shift | worth | dysph | 0.19 | -.05 | 0.43 | 0.1239 | 8109 | 78 | 59 | 2 | 0.95 | 3.28 |
| cg | Shift | dysph | attend | 0.09 | -.16 | 0.33 | 0.4887 | 6945 | 352 | 23 | 2 | 4.82 | 8.00 |
| bj | Shift | attend | dysph | 0.00 | -.14 | 0.14 | 0.9889 | 7236 | 61 | 944 | 8 | 0.84 | 0.84 |
| cg | Shift | dysph | hyper | 0.30 | 0.12 | 0.49 | **0.0014** | 7519 | 183 | 35 | 5 | 2.38 | 12.50 |
| bj | Shift | hyper | dysph | 0.04 | -.10 | 0.18 | 0.5696 | 7611 | 91 | 539 | 8 | 1.18 | 1.46 |
| cg | Shift | dysph | argues | 0.15 | -.07 | 0.38 | 0.1703 | 7152 | 407 | 21 | 3 | 5.38 | 12.50 |
| bj | Shift | argues | dysph | -.02 | -.18 | 0.15 | 0.8426 | 7499 | 60 | 685 | 5 | 0.79 | 0.72 |
| cg | Shift | dysph | temper | 0.32 | 0.09 | 0.54 | **0.0054** | 7837 | 123 | 26 | 3 | 1.55 | 10.34 |
| bj | Shift | temper | dysph | 0.10 | -.05 | 0.26 | 0.1963 | 7867 | 93 | 283 | 6 | 1.17 | 2.08 |
| cg | Shift | dysph | fights | . | . | . | . | 8198 | 21 | 54 | 0 | 0.26 | 0.00 |
| bj | Shift | fights | dysph | . | . | . | . | 8102 | 117 | 30 | 0 | 1.42 | 0.00 |
| cg | Shift | dysph | steals | . | . | . | . | 8193 | 28 | 55 | 0 | 0.34 | 0.00 |
| bj | Shift | steals | dysph | . | . | . | . | 8105 | 116 | 28 | 0 | 1.41 | 0.00 |
| cg | Shift | worth | attend | -.08 | -.36 | 0.20 | 0.5767 | 6929 | 358 | 33 | 1 | 4.91 | 2.94 |
| bj | Shift | attend | worth | -.08 | -.27 | 0.12 | 0.4424 | 7251 | 36 | 938 | 3 | 0.49 | 0.32 |
| cg | Shift | worth | hyper | 0.28 | 0.11 | 0.45 | **0.0014** | 7501 | 184 | 50 | 6 | 2.39 | 10.71 |
| bj | Shift | hyper | worth | 0.07 | -.09 | 0.23 | 0.3908 | 7627 | 58 | 537 | 6 | 0.75 | 1.10 |
| cg | Shift | worth | argues | 0.24 | 0.07 | 0.40 | **0.0054** | 7123 | 419 | 33 | 7 | 5.56 | 17.50 |
| bj | Shift | argues | worth | 0.05 | -.12 | 0.22 | 0.5788 | 7500 | 42 | 681 | 5 | 0.56 | 0.73 |
| cg | Shift | worth | temper | 0.03 | -.26 | 0.32 | 0.8249 | 7808 | 132 | 47 | 1 | 1.66 | 2.08 |
| bj | Shift | temper | worth | 0.24 | 0.09 | 0.40 | **0.0017** | 7881 | 59 | 280 | 8 | 0.74 | 2.78 |
| cg | Shift | worth | fights | . | . | . | . | 8178 | 19 | 75 | 0 | 0.23 | 0.00 |
| bj | Shift | fights | worth | 0.30 | 0.04 | 0.55 | **0.0232** | 8119 | 78 | 29 | 2 | 0.95 | 6.45 |
| cg | Shift | worth | steals | 0.32 | 0.06 | 0.57 | **0.0158** | 8173 | 27 | 73 | 2 | 0.33 | 2.67 |
| bj | Shift | steals | worth | . | . | . | . | 8120 | 80 | 28 | 0 | 0.98 | 0.00 |
| cg | Shift | attend | hyper | 0.11 | -.05 | 0.26 | 0.1836 | 7130 | 51 | 554 | 7 | 0.71 | 1.25 |
| bj | Shift | hyper | attend | 0.06 | -.09 | 0.20 | 0.4402 | 6917 | 264 | 134 | 7 | 3.68 | 4.96 |
| cg | Shift | attend | argues | -.04 | -.13 | 0.06 | 0.4304 | 6621 | 270 | 669 | 23 | 3.92 | 3.32 |
| bj | Shift | argues | attend | 0.04 | -.07 | 0.14 | 0.4689 | 6650 | 241 | 413 | 18 | 3.50 | 4.18 |
| cg | Shift | attend | temper | -.01 | -.15 | 0.13 | 0.9125 | 7079 | 71 | 831 | 8 | 0.99 | 0.95 |
| bj | Shift | temper | attend | 0.19 | 0.08 | 0.30 | **0.0009** | 6832 | 318 | 154 | 18 | 4.45 | 10.47 |
| cg | Shift | attend | fights | -.03 | -.34 | 0.29 | 0.8685 | 7300 | 9 | 963 | 1 | 0.12 | 0.10 |
| bj | Shift | fights | attend | 0.19 | -.07 | 0.46 | 0.1553 | 6931 | 378 | 11 | 2 | 5.17 | 15.38 |
| cg | Shift | attend | steals | 0.01 | -.24 | 0.26 | 0.9184 | 7296 | 14 | 964 | 2 | 0.19 | 0.21 |
| bj | Shift | steals | attend | 0.08 | -.25 | 0.40 | 0.6469 | 6929 | 381 | 11 | 1 | 5.21 | 8.33 |
| cg | Shift | hyper | argues | 0.10 | -.00 | 0.19 | 0.0530 | 6890 | 338 | 330 | 25 | 4.68 | 7.04 |
| bj | Shift | argues | hyper | 0.08 | -.04 | 0.21 | 0.2017 | 7115 | 113 | 502 | 12 | 1.56 | 2.33 |
| cg | Shift | hyper | temper | 0.14 | 0.01 | 0.26 | **0.0367** | 7433 | 101 | 443 | 12 | 1.34 | 2.64 |
| bj | Shift | temper | hyper | 0.27 | 0.15 | 0.38 | **0.0000** | 7372 | 162 | 192 | 16 | 2.15 | 7.69 |
| cg | Shift | hyper | fights | 0.12 | -.13 | 0.38 | 0.3449 | 7708 | 13 | 550 | 2 | 0.17 | 0.36 |
| bj | Shift | fights | hyper | . | . | . | . | 7521 | 200 | 21 | 0 | 2.59 | 0.00 |
| cg | Shift | hyper | steals | 0.15 | -.07 | 0.38 | 0.1811 | 7706 | 17 | 550 | 3 | 0.22 | 0.54 |
| bj | Shift | steals | hyper | 0.39 | 0.18 | 0.60 | **0.0003** | 7529 | 194 | 15 | 4 | 2.51 | 21.05 |
| cg | Shift | argues | temper | 0.07 | -.12 | 0.26 | 0.4669 | 7423 | 38 | 524 | 4 | 0.51 | 0.76 |
| bj | Shift | temper | argues | 0.21 | 0.09 | 0.33 | **0.0007** | 7111 | 350 | 107 | 15 | 4.69 | 12.30 |
| cg | Shift | argues | fights | . | . | . | . | 7568 | 6 | 699 | 0 | 0.08 | 0.00 |
| bj | Shift | fights | argues | 0.11 | -.23 | 0.45 | 0.5271 | 7138 | 436 | 8 | 1 | 5.76 | 11.11 |
| cg | Shift | argues | steals | 0.16 | -.11 | 0.44 | 0.2453 | 7562 | 8 | 704 | 2 | 0.11 | 0.28 |
| bj | Shift | steals | argues | 0.27 | 0.03 | 0.51 | **0.0279** | 7138 | 432 | 10 | 3 | 5.71 | 23.08 |
| cg | Shift | temper | fights | 0.26 | -.01 | 0.53 | 0.0549 | 7965 | 10 | 296 | 2 | 0.13 | 0.67 |
| bj | Shift | fights | temper | 0.21 | -.12 | 0.54 | 0.2085 | 7839 | 136 | 13 | 1 | 1.71 | 7.14 |
| cg | Shift | temper | steals | 0.41 | 0.22 | 0.59 | **0.0000** | 7952 | 15 | 303 | 6 | 0.19 | 1.94 |
| bj | Shift | steals | temper | 0.27 | 0.01 | 0.53 | 0.0405 | 7833 | 134 | 20 | 2 | 1.68 | 9.09 |
| cg | Shift | fights | steals | . | . | . | . | 8215 | 30 | 31 | 0 | 0.36 | 0.00 |
| bj | Shift | steals | fights | 0.38 | 0.04 | 0.72 | **0.0297** | 8226 | 19 | 27 | 1 | 0.23 | 3.57 |
| co | Subtract x | fear+worry | worry | 0.47 | 0.36 | 0.58 | **0.0000** | 7734 | 139 | 102 | 20 | 1.77 | 16.39 |
| co | Subtract x | fear+dysph | dysph | 0.38 | 0.12 | 0.64 | **0.0048** | 7995 | 70 | 18 | 2 | 0.87 | 10.00 |
| co | Subtract x | fear+worth | worth | 0.26 | -.06 | 0.59 | 0.1143 | 8013 | 41 | 29 | 1 | 0.51 | 3.33 |
| co | Subtract x | fear+attend | attend | 0.44 | 0.34 | 0.54 | **0.0000** | 6880 | 333 | 68 | 27 | 4.62 | 28.42 |
| co | Subtract x | fear+hyper | hyper | 0.51 | 0.39 | 0.62 | **0.0000** | 7432 | 178 | 54 | 18 | 2.34 | 25.00 |
| co | Subtract x | fear+argues | argues | 0.44 | 0.33 | 0.55 | **0.0000** | 7058 | 396 | 51 | 24 | 5.31 | 32.00 |
| co | Subtract x | fear+temper | temper | 0.43 | 0.27 | 0.59 | **0.0000** | 7723 | 116 | 46 | 8 | 1.48 | 14.81 |
| co | Subtract x | fear+fights | fights | . | . | . | . | 8056 | 17 | 4 | 0 | 0.21 | 0.00 |
| co | Subtract x | fear+steals | steals | . | . | . | . | 8047 | 27 | 2 | 0 | 0.33 | 0.00 |
| co | Subtract x | worry+dysph | dysph | 0.22 | -.10 | 0.55 | 0.1764 | 7872 | 55 | 29 | 1 | 0.69 | 3.33 |
| co | Subtract x | worry+worth | worth | 0.24 | -.08 | 0.57 | 0.1421 | 7882 | 35 | 39 | 1 | 0.44 | 2.50 |
| co | Subtract x | worry+attend | attend | 0.44 | 0.35 | 0.54 | **0.0000** | 6792 | 309 | 97 | 34 | 4.35 | 25.95 |
| co | Subtract x | worry+hyper | hyper | 0.44 | 0.31 | 0.56 | **0.0000** | 7300 | 171 | 66 | 15 | 2.29 | 18.52 |
| co | Subtract x | worry+argues | argues | 0.33 | 0.22 | 0.44 | **0.0000** | 6948 | 387 | 82 | 22 | 5.28 | 21.15 |
| co | Subtract x | worry+temper | temper | 0.32 | 0.15 | 0.50 | **0.0002** | 7587 | 116 | 60 | 6 | 1.51 | 9.09 |
| co | Subtract x | worry+fights | fights | 0.69 | 0.44 | 0.94 | **0.0000** | 7912 | 17 | 6 | 2 | 0.21 | 25.00 |
| co | Subtract x | worry+steals | steals | . | . | . | . | 7902 | 26 | 4 | 0 | 0.33 | 0.00 |
| co | Subtract x | dysph+worth | worth | 0.47 | 0.20 | 0.73 | **0.0005** | 8143 | 44 | 15 | 2 | 0.54 | 11.76 |
| co | Subtract x | dysph+attend | attend | 0.46 | 0.31 | 0.60 | **0.0000** | 6945 | 352 | 21 | 12 | 4.82 | 36.36 |
| co | Subtract x | dysph+hyper | hyper | 0.41 | 0.20 | 0.62 | **0.0002** | 7519 | 183 | 14 | 4 | 2.38 | 22.22 |
| co | Subtract x | dysph+argues | argues | 0.43 | 0.29 | 0.57 | **0.0000** | 7152 | 407 | 22 | 12 | 5.38 | 35.29 |
| co | Subtract x | dysph+temper | temper | 0.38 | 0.17 | 0.58 | **0.0003** | 7837 | 123 | 25 | 4 | 1.55 | 13.79 |
| co | Subtract x | dysph+fights | fights | 0.63 | 0.29 | 0.98 | **0.0003** | 8198 | 21 | 3 | 1 | 0.26 | 25.00 |
| co | Subtract x | dysph+steals | steals | . | . | . | . | 8193 | 28 | 3 | 0 | 0.34 | 0.00 |
| co | Subtract x | worth+attend | attend | 0.57 | 0.47 | 0.68 | **0.0000** | 6929 | 358 | 22 | 22 | 4.91 | 50.00 |
| co | Subtract x | worth+hyper | hyper | 0.51 | 0.34 | 0.68 | **0.0000** | 7501 | 184 | 15 | 7 | 2.39 | 31.82 |
| co | Subtract x | worth+argues | argues | 0.42 | 0.28 | 0.56 | **0.0000** | 7123 | 419 | 25 | 13 | 5.56 | 34.21 |
| co | Subtract x | worth+temper | temper | 0.49 | 0.33 | 0.66 | **0.0000** | 7808 | 132 | 23 | 7 | 1.66 | 23.33 |
| co | Subtract x | worth+fights | fights | . | . | . | . | 8178 | 19 | 3 | 0 | 0.23 | 0.00 |
| co | Subtract x | worth+steals | steals | 0.65 | 0.31 | 1.00 | **0.0002** | 8173 | 27 | 2 | 1 | 0.33 | 33.33 |
| co | Subtract x | attend+hyper | hyper | 0.25 | 0.11 | 0.39 | **0.0004** | 7130 | 51 | 413 | 11 | 0.71 | 2.59 |
| co | Subtract x | attend+argues | argues | 0.33 | 0.25 | 0.42 | **0.0000** | 6621 | 270 | 250 | 43 | 3.92 | 14.68 |
| co | Subtract x | attend+temper | temper | 0.34 | 0.19 | 0.49 | **0.0000** | 7079 | 71 | 137 | 9 | 0.99 | 6.16 |
| co | Subtract x | attend+fights | fights | . | . | . | . | 7300 | 9 | 21 | 0 | 0.12 | 0.00 |
| co | Subtract x | attend+steals | steals | 0.46 | 0.11 | 0.81 | **0.0103** | 7296 | 14 | 18 | 1 | 0.19 | 5.26 |
| co | Subtract x | hyper+argues | argues | 0.47 | 0.39 | 0.54 | **0.0000** | 6890 | 338 | 153 | 57 | 4.68 | 27.14 |
| co | Subtract x | hyper+temper | temper | 0.34 | 0.19 | 0.49 | **0.0000** | 7433 | 101 | 101 | 9 | 1.34 | 8.18 |
| co | Subtract x | hyper+fights | fights | 0.52 | 0.17 | 0.87 | **0.0035** | 7708 | 13 | 12 | 1 | 0.17 | 7.69 |
| co | Subtract x | hyper+steals | steals | 0.50 | 0.15 | 0.85 | **0.0051** | 7706 | 17 | 11 | 1 | 0.22 | 8.33 |
| co | Subtract x | argues+temper | temper | 0.41 | 0.25 | 0.56 | **0.0000** | 7423 | 38 | 187 | 9 | 0.51 | 4.59 |
| co | Subtract x | argues+fights | fights | . | . | . | . | 7568 | 6 | 25 | 0 | 0.08 | 0.00 |
| co | Subtract x | argues+steals | steals | 0.66 | 0.41 | 0.92 | **0.0000** | 7562 | 8 | 16 | 2 | 0.11 | 11.11 |
| co | Subtract x | temper+fights | fights | . | . | . | . | 7965 | 10 | 20 | 0 | 0.13 | 0.00 |
| co | Subtract x | temper+steals | steals | . | . | . | . | 7952 | 15 | 9 | 0 | 0.19 | 0.00 |
| co | Subtract x | fights+steals | steals | . | . | . | . | 8215 | 30 | 3 | 0 | 0.36 | 0.00 |
| bn | Subtract y | worry+fear | fear | 0.36 | 0.20 | 0.51 | **0.0000** | 7799 | 74 | 114 | 8 | 0.94 | 6.56 |
| bn | Subtract y | dysph+fear | fear | 0.43 | 0.22 | 0.64 | **0.0000** | 7926 | 139 | 16 | 4 | 1.72 | 20.00 |
| bn | Subtract y | worth+fear | fear | 0.51 | 0.36 | 0.67 | **0.0000** | 7909 | 145 | 22 | 8 | 1.80 | 26.67 |
| bn | Subtract y | attend+fear | fear | 0.14 | -.07 | 0.34 | 0.1990 | 7112 | 101 | 92 | 3 | 1.40 | 3.16 |
| bn | Subtract y | hyper+fear | fear | 0.28 | 0.11 | 0.45 | **0.0014** | 7471 | 139 | 66 | 6 | 1.83 | 8.33 |
| bn | Subtract y | argues+fear | fear | 0.22 | 0.03 | 0.41 | **0.0256** | 7343 | 111 | 71 | 4 | 1.49 | 5.33 |
| bn | Subtract y | temper+fear | fear | 0.42 | 0.27 | 0.57 | **0.0000** | 7694 | 145 | 45 | 9 | 1.85 | 16.67 |
| bn | Subtract y | fights+fear | fear | 0.56 | 0.27 | 0.85 | **0.0002** | 7892 | 181 | 2 | 2 | 2.24 | 50.00 |
| bn | Subtract y | steals+fear | fear | 0.54 | 0.14 | 0.94 | **0.0084** | 7894 | 180 | 1 | 1 | 2.23 | 50.00 |
| bn | Subtract y | dysph+worry | worry | 0.43 | 0.27 | 0.60 | **0.0000** | 7732 | 195 | 23 | 7 | 2.46 | 23.33 |
| bn | Subtract y | worth+worry | worry | 0.51 | 0.37 | 0.64 | **0.0000** | 7711 | 206 | 28 | 12 | 2.60 | 30.00 |
| bn | Subtract y | attend+worry | worry | 0.25 | 0.11 | 0.39 | **0.0004** | 6951 | 150 | 121 | 10 | 2.11 | 7.63 |
| bn | Subtract y | hyper+worry | worry | 0.45 | 0.33 | 0.57 | **0.0000** | 7276 | 195 | 64 | 17 | 2.61 | 20.99 |
| bn | Subtract y | argues+worry | worry | 0.31 | 0.18 | 0.45 | **0.0000** | 7175 | 160 | 93 | 11 | 2.18 | 10.58 |
| bn | Subtract y | temper+worry | worry | 0.48 | 0.36 | 0.60 | **0.0000** | 7505 | 198 | 50 | 16 | 2.57 | 24.24 |
| bn | Subtract y | fights+worry | worry | 0.37 | 0.09 | 0.66 | **0.0100** | 7693 | 236 | 6 | 2 | 2.98 | 25.00 |
| bn | Subtract y | steals+worry | worry | . | . | . | . | 7691 | 237 | 4 | 0 | 2.99 | 0.00 |
| bn | Subtract y | worth+dysph | dysph | 0.48 | 0.25 | 0.71 | **0.0000** | 8109 | 78 | 14 | 3 | 0.95 | 17.65 |
| bn | Subtract y | attend+dysph | dysph | . | . | . | . | 7236 | 61 | 33 | 0 | 0.84 | 0.00 |
| bn | Subtract y | hyper+dysph | dysph | 0.35 | 0.09 | 0.62 | **0.0088** | 7611 | 91 | 16 | 2 | 1.18 | 11.11 |
| bn | Subtract y | argues+dysph | dysph | 0.19 | -.13 | 0.51 | 0.2493 | 7499 | 60 | 33 | 1 | 0.79 | 2.94 |
| bn | Subtract y | temper+dysph | dysph | 0.42 | 0.22 | 0.62 | **0.0001** | 7867 | 93 | 25 | 4 | 1.17 | 13.79 |
| bn | Subtract y | fights+dysph | dysph | 0.44 | 0.07 | 0.82 | **0.0195** | 8102 | 117 | 3 | 1 | 1.42 | 25.00 |
| bn | Subtract y | steals+dysph | dysph | . | . | . | . | 8105 | 116 | 3 | 0 | 1.41 | 0.00 |
| bn | Subtract y | attend+worth | worth | 0.42 | 0.19 | 0.65 | **0.0003** | 7251 | 36 | 41 | 3 | 0.49 | 6.82 |
| bn | Subtract y | hyper+worth | worth | 0.47 | 0.24 | 0.70 | **0.0001** | 7627 | 58 | 19 | 3 | 0.75 | 13.64 |
| bn | Subtract y | argues+worth | worth | 0.34 | 0.08 | 0.60 | **0.0096** | 7500 | 42 | 36 | 2 | 0.56 | 5.26 |
| bn | Subtract y | temper+worth | worth | 0.48 | 0.28 | 0.68 | **0.0000** | 7881 | 59 | 26 | 4 | 0.74 | 13.33 |
| bn | Subtract y | fights+worth | worth | . | . | . | . | 8119 | 78 | 3 | 0 | 0.95 | 0.00 |
| bn | Subtract y | steals+worth | worth | . | . | . | . | 8120 | 80 | 3 | 0 | 0.98 | 0.00 |
| bn | Subtract y | hyper+attend | attend | 0.51 | 0.45 | 0.57 | **0.0000** | 6917 | 264 | 325 | 99 | 3.68 | 23.35 |
| bn | Subtract y | argues+attend | attend | 0.46 | 0.38 | 0.53 | **0.0000** | 6650 | 241 | 233 | 60 | 3.50 | 20.48 |
| bn | Subtract y | temper+attend | attend | 0.58 | 0.51 | 0.66 | **0.0000** | 6832 | 318 | 88 | 58 | 4.45 | 39.73 |
| bn | Subtract y | fights+attend | attend | 0.40 | 0.22 | 0.58 | **0.0000** | 6931 | 378 | 14 | 7 | 5.17 | 33.33 |
| bn | Subtract y | steals+attend | attend | 0.58 | 0.43 | 0.72 | **0.0000** | 6929 | 381 | 8 | 11 | 5.21 | 57.89 |
| bn | Subtract y | argues+hyper | hyper | 0.49 | 0.39 | 0.59 | **0.0000** | 7115 | 113 | 180 | 30 | 1.56 | 14.29 |
| bn | Subtract y | temper+hyper | hyper | 0.52 | 0.42 | 0.62 | **0.0000** | 7372 | 162 | 85 | 25 | 2.15 | 22.73 |
| bn | Subtract y | fights+hyper | hyper | 0.58 | 0.39 | 0.76 | **0.0000** | 7521 | 200 | 7 | 6 | 2.59 | 46.15 |
| bn | Subtract y | steals+hyper | hyper | 0.55 | 0.35 | 0.74 | **0.0000** | 7529 | 194 | 7 | 5 | 2.51 | 41.67 |
| bn | Subtract y | temper+argues | argues | 0.53 | 0.46 | 0.60 | **0.0000** | 7111 | 350 | 130 | 66 | 4.69 | 33.67 |
| bn | Subtract y | fights+argues | argues | 0.44 | 0.29 | 0.60 | **0.0000** | 7138 | 436 | 15 | 10 | 5.76 | 40.00 |
| bn | Subtract y | steals+argues | argues | 0.42 | 0.24 | 0.60 | **0.0000** | 7138 | 432 | 11 | 7 | 5.71 | 38.89 |
| bn | Subtract y | fights+temper | temper | 0.53 | 0.36 | 0.71 | **0.0000** | 7839 | 136 | 14 | 6 | 1.71 | 30.00 |
| bn | Subtract y | steals+temper | temper | 0.43 | 0.15 | 0.70 | **0.0025** | 7833 | 134 | 7 | 2 | 1.68 | 22.22 |
| bn | Subtract y | steals+fights | fights | 0.69 | 0.35 | 1.00 | **0.0001** | 8226 | 19 | 2 | 1 | 0.23 | 33.33 |

| Supplemental Table S3M. Tetrachoric correlations from 4 x 4 pairwise analyses of all 90 combinations of the 10 selected psychological problems with every other problem to **compare differences** between three pairs of paths to the **first annual follow-up.** Results are for problems dichotomized at the **low rating cut (0 vs 1 or 2).** r_t_ = tetrachoric correlation. –CL and +CL = lower and upper 95% confidence intervals for the tetrachoric correlation. c00 = number without the predictor who followed path 1; c01 = number without the predictor who followed path 2; c10 = number with the predictor who followed path 1; c11 = number with the predictor who followed path 2. Base path 1 % = percent without predictor who followed path 1; Obs path 1 % = percent with predictor who followed path 1. Base path 2 % = percent without predictor who followed path 2; Obs path 2 % = percent with predictor who followed path 2. Raw P values significant after FDR adjustment in **bold**. | | | | | | | | | | | | | | | |
| --- | --- | --- | --- | --- | --- | --- | --- | --- | --- | --- | --- | --- | --- | --- | --- |
| prefix | Compare Paths | Path 1 | Path 2 | r_t_ | -CL | +CL | P | c00 | c01 | c10 | c11 | Baserate path 1% | Observed path 1% | Baserate path 2% | Observed path 2% |
| gh | Shift VS Add | fear to worry | fear to fear+worry | 0.52 | 0.43 | 0.60 | **0.0000** | 687 | 270 | 76 | 145 | 71.79 | 34.39 | 28.21 | 65.61 |
| jl | Shift VS Add | worry to fear | worry to worry+fear | 0.45 | 0.36 | 0.54 | **0.0000** | 206 | 270 | 69 | 327 | 43.28 | 17.42 | 56.72 | 82.58 |
| gh | Shift VS Add | fear to dysph | fear to fear+dysph | 0.60 | 0.51 | 0.69 | **0.0000** | 302 | 170 | 47 | 162 | 63.98 | 22.49 | 36.02 | 77.51 |
| jl | Shift VS Add | dysph to fear | dysph to dysph+fear | 0.57 | 0.47 | 0.67 | **0.0000** | 575 | 170 | 44 | 83 | 77.18 | 34.65 | 22.82 | 65.35 |
| gh | Shift VS Add | fear to worth | fear to fear+worth | 0.57 | 0.48 | 0.67 | **0.0000** | 286 | 157 | 49 | 149 | 64.56 | 24.75 | 35.44 | 75.25 |
| jl | Shift VS Add | worth to fear | worth to worth+fear | 0.58 | 0.48 | 0.68 | **0.0000** | 591 | 157 | 45 | 79 | 79.01 | 36.29 | 20.99 | 63.71 |
| gh | Shift VS Add | fear to attend | fear to fear+attend | 0.63 | 0.54 | 0.72 | **0.0000** | 558 | 109 | 61 | 92 | 83.66 | 39.87 | 16.34 | 60.13 |
| jl | Shift VS Add | attend to fear | attend to attend+fear | 0.82 | 0.78 | 0.87 | **0.0000** | 294 | 109 | 60 | 409 | 72.95 | 12.79 | 27.05 | 87.21 |
| gh | Shift VS Add | fear to hyper | fear to fear+hyper | 0.73 | 0.65 | 0.81 | **0.0000** | 421 | 88 | 46 | 110 | 82.71 | 29.49 | 17.29 | 70.51 |
| jl | Shift VS Add | hyper to fear | hyper to hyper+fear | 0.79 | 0.74 | 0.85 | **0.0000** | 468 | 88 | 83 | 233 | 84.17 | 26.27 | 15.83 | 73.73 |
| gh | Shift VS Add | fear to argues | fear to fear+argues | 0.68 | 0.60 | 0.75 | **0.0000** | 695 | 137 | 63 | 119 | 83.53 | 34.62 | 16.47 | 65.38 |
| jl | Shift VS Add | argues to fear | argues to argues+fear | 0.72 | 0.66 | 0.79 | **0.0000** | 310 | 137 | 79 | 346 | 69.35 | 18.59 | 30.65 | 81.41 |
| gh | Shift VS Add | fear to temper | fear to fear+temper | 0.68 | 0.59 | 0.76 | **0.0000** | 456 | 111 | 51 | 112 | 80.42 | 31.29 | 19.58 | 68.71 |
| jl | Shift VS Add | temper to fear | temper to temper+fear | 0.73 | 0.66 | 0.79 | **0.0000** | 487 | 111 | 81 | 193 | 81.44 | 29.56 | 18.56 | 70.44 |
| gh | Shift VS Add | fear to fights | fear to fear+fights | 0.70 | 0.58 | 0.83 | **0.0000** | 145 | 53 | 16 | 58 | 73.23 | 21.62 | 26.77 | 78.38 |
| jl | Shift VS Add | fights to fear | fights to fights+fear | 0.72 | 0.62 | 0.82 | **0.0000** | 752 | 53 | 31 | 36 | 93.42 | 46.27 | 6.58 | 53.73 |
| gh | Shift VS Add | fear to steals | fear to fear+steals | 0.85 | 0.75 | 0.94 | **0.0000** | 124 | 26 | 8 | 42 | 82.67 | 16.00 | 17.33 | 84.00 |
| jl | Shift VS Add | steals to fear | steals to steals+fear | 0.83 | 0.74 | 0.92 | **0.0000** | 799 | 26 | 20 | 27 | 96.85 | 42.55 | 3.15 | 57.45 |
| gh | Shift VS Add | worry to dysph | worry to worry+dysph | 0.51 | 0.41 | 0.61 | **0.0000** | 161 | 177 | 60 | 283 | 47.63 | 17.49 | 52.37 | 82.51 |
| jl | Shift VS Add | dysph to worry | dysph to dysph+worry | 0.52 | 0.41 | 0.62 | **0.0000** | 887 | 177 | 53 | 61 | 83.36 | 46.49 | 16.64 | 53.51 |
| gh | Shift VS Add | worry to worth | worry to worry+worth | 0.53 | 0.43 | 0.62 | **0.0000** | 168 | 156 | 61 | 256 | 51.85 | 19.24 | 48.15 | 80.76 |
| jl | Shift VS Add | worth to worry | worth to worth+worry | 0.58 | 0.48 | 0.67 | **0.0000** | 898 | 156 | 55 | 69 | 85.20 | 44.35 | 14.80 | 55.65 |
| gh | Shift VS Add | worry to attend | worry to worry+attend | 0.63 | 0.55 | 0.71 | **0.0000** | 409 | 169 | 63 | 179 | 70.76 | 26.03 | 29.24 | 73.97 |
| jl | Shift VS Add | attend to worry | attend to attend+worry | 0.78 | 0.74 | 0.83 | **0.0000** | 458 | 169 | 91 | 460 | 73.05 | 16.52 | 26.95 | 83.48 |
| gh | Shift VS Add | worry to hyper | worry to worry+hyper | 0.76 | 0.69 | 0.82 | **0.0000** | 332 | 101 | 49 | 183 | 76.67 | 21.12 | 23.33 | 78.88 |
| jl | Shift VS Add | hyper to worry | hyper to hyper+worry | 0.80 | 0.75 | 0.84 | **0.0000** | 709 | 101 | 112 | 256 | 87.53 | 30.43 | 12.47 | 69.57 |
| gh | Shift VS Add | worry to argues | worry to worry+argues | 0.68 | 0.62 | 0.75 | **0.0000** | 529 | 173 | 82 | 230 | 75.36 | 26.28 | 24.64 | 73.72 |
| jl | Shift VS Add | argues to worry | argues to argues+worry | 0.74 | 0.69 | 0.79 | **0.0000** | 493 | 173 | 106 | 406 | 74.02 | 20.70 | 25.98 | 79.30 |
| gh | Shift VS Add | worry to temper | worry to worry+temper | 0.68 | 0.60 | 0.75 | **0.0000** | 341 | 123 | 68 | 198 | 73.49 | 25.56 | 26.51 | 74.44 |
| jl | Shift VS Add | temper to worry | temper to temper+worry | 0.72 | 0.66 | 0.78 | **0.0000** | 749 | 123 | 112 | 194 | 85.89 | 36.60 | 14.11 | 63.40 |
| gh | Shift VS Add | worry to fights | worry to worry+fights | 0.73 | 0.62 | 0.84 | **0.0000** | 107 | 51 | 19 | 95 | 67.72 | 16.67 | 32.28 | 83.33 |
| jl | Shift VS Add | fights to worry | fights to fights+worry | 0.68 | 0.57 | 0.79 | **0.0000** | 1052 | 51 | 43 | 32 | 95.38 | 57.33 | 4.62 | 42.67 |
| gh | Shift VS Add | worry to steals | worry to worry+steals | 0.71 | 0.58 | 0.85 | **0.0000** | 95 | 31 | 18 | 56 | 75.40 | 24.32 | 24.60 | 75.68 |
| jl | Shift VS Add | steals to worry | steals to steals+worry | 0.86 | 0.79 | 0.93 | **0.0000** | 1100 | 31 | 17 | 30 | 97.26 | 36.17 | 2.74 | 63.83 |
| gh | Shift VS Add | dysph to worth | dysph to dysph+worth | 0.57 | 0.46 | 0.68 | **0.0000** | 336 | 193 | 24 | 88 | 63.52 | 21.43 | 36.48 | 78.57 |
| jl | Shift VS Add | worth to dysph | worth to worth+dysph | 0.53 | 0.42 | 0.64 | **0.0000** | 358 | 193 | 34 | 96 | 64.97 | 26.15 | 35.03 | 73.85 |
| gh | Shift VS Add | dysph to attend | dysph to dysph+attend | 0.64 | 0.52 | 0.76 | **0.0000** | 669 | 87 | 27 | 37 | 88.49 | 42.19 | 11.51 | 57.81 |
| jl | Shift VS Add | attend to dysph | attend to attend+dysph | 0.84 | 0.78 | 0.89 | **0.0000** | 184 | 87 | 37 | 373 | 67.90 | 9.02 | 32.10 | 90.98 |
| gh | Shift VS Add | dysph to hyper | dysph to dysph+hyper | 0.56 | 0.43 | 0.69 | **0.0000** | 500 | 95 | 31 | 39 | 84.03 | 44.29 | 15.97 | 55.71 |
| jl | Shift VS Add | hyper to dysph | hyper to hyper+dysph | 0.73 | 0.66 | 0.80 | **0.0000** | 309 | 95 | 67 | 210 | 76.49 | 24.19 | 23.51 | 75.81 |
| gh | Shift VS Add | dysph to argues | dysph to dysph+argues | 0.74 | 0.66 | 0.83 | **0.0000** | 827 | 112 | 21 | 54 | 88.07 | 28.00 | 11.93 | 72.00 |
| jl | Shift VS Add | argues to dysph | argues to argues+dysph | 0.70 | 0.62 | 0.77 | **0.0000** | 177 | 112 | 59 | 333 | 61.25 | 15.05 | 38.75 | 84.95 |
| gh | Shift VS Add | dysph to temper | dysph to dysph+temper | 0.50 | 0.37 | 0.63 | **0.0000** | 533 | 125 | 32 | 40 | 81.00 | 44.44 | 19.00 | 55.56 |
| jl | Shift VS Add | temper to dysph | temper to temper+dysph | 0.68 | 0.61 | 0.76 | **0.0000** | 317 | 125 | 55 | 184 | 71.72 | 23.01 | 28.28 | 76.99 |
| gh | Shift VS Add | dysph to fights | dysph to dysph+fights | 0.74 | 0.62 | 0.86 | **0.0000** | 161 | 53 | 11 | 47 | 75.23 | 18.97 | 24.77 | 81.03 |
| jl | Shift VS Add | fights to dysph | fights to fights+dysph | 0.69 | 0.58 | 0.80 | **0.0000** | 562 | 53 | 30 | 36 | 91.38 | 45.45 | 8.62 | 54.55 |
| gh | Shift VS Add | dysph to steals | dysph to dysph+steals | 0.69 | 0.53 | 0.85 | **0.0000** | 115 | 45 | 8 | 32 | 71.88 | 20.00 | 28.13 | 80.00 |
| jl | Shift VS Add | steals to dysph | steals to steals+dysph | 0.76 | 0.65 | 0.86 | **0.0000** | 589 | 45 | 18 | 29 | 92.90 | 38.30 | 7.10 | 61.70 |
| gh | Shift VS Add | worth to attend | worth to worth+attend | 0.71 | 0.60 | 0.81 | **0.0000** | 679 | 85 | 19 | 37 | 88.87 | 33.93 | 11.13 | 66.07 |
| jl | Shift VS Add | attend to worth | attend to attend+worth | 0.77 | 0.70 | 0.84 | **0.0000** | 162 | 85 | 50 | 344 | 65.59 | 12.69 | 34.41 | 87.31 |
| gh | Shift VS Add | worth to hyper | worth to worth+hyper | 0.70 | 0.60 | 0.80 | **0.0000** | 516 | 72 | 28 | 49 | 87.76 | 36.36 | 12.24 | 63.64 |
| jl | Shift VS Add | hyper to worth | hyper to hyper+worth | 0.77 | 0.71 | 0.84 | **0.0000** | 304 | 72 | 66 | 199 | 80.85 | 24.91 | 19.15 | 75.09 |
| gh | Shift VS Add | worth to argues | worth to worth+argues | 0.68 | 0.59 | 0.77 | **0.0000** | 817 | 92 | 45 | 60 | 89.88 | 42.86 | 10.12 | 57.14 |
| jl | Shift VS Add | argues to worth | argues to argues+worth | 0.73 | 0.66 | 0.80 | **0.0000** | 165 | 92 | 56 | 328 | 64.20 | 14.58 | 35.80 | 85.42 |
| gh | Shift VS Add | worth to temper | worth to worth+temper | 0.68 | 0.58 | 0.77 | **0.0000** | 541 | 86 | 39 | 64 | 86.28 | 37.86 | 13.72 | 62.14 |
| jl | Shift VS Add | temper to worth | temper to temper+worth | 0.75 | 0.69 | 0.82 | **0.0000** | 295 | 86 | 59 | 201 | 77.43 | 22.69 | 22.57 | 77.31 |
| gh | Shift VS Add | worth to fights | worth to worth+fights | 0.70 | 0.56 | 0.83 | **0.0000** | 167 | 41 | 19 | 45 | 80.29 | 29.69 | 19.71 | 70.31 |
| jl | Shift VS Add | fights to worth | fights to fights+worth | 0.73 | 0.62 | 0.83 | **0.0000** | 519 | 41 | 38 | 43 | 92.68 | 46.91 | 7.32 | 53.09 |
| gh | Shift VS Add | worth to steals | worth to worth+steals | 0.70 | 0.54 | 0.85 | **0.0000** | 124 | 34 | 11 | 31 | 78.48 | 26.19 | 21.52 | 73.81 |
| jl | Shift VS Add | steals to worth | steals to steals+worth | 0.77 | 0.66 | 0.88 | **0.0000** | 562 | 34 | 19 | 26 | 94.30 | 42.22 | 5.70 | 57.78 |
| gh | Shift VS Add | attend to hyper | attend to attend+hyper | 0.57 | 0.47 | 0.67 | **0.0000** | 118 | 186 | 35 | 326 | 38.82 | 9.70 | 61.18 | 90.30 |
| jl | Shift VS Add | hyper to attend | hyper to hyper+attend | 0.55 | 0.44 | 0.65 | **0.0000** | 513 | 186 | 39 | 82 | 73.39 | 32.23 | 26.61 | 67.77 |
| gh | Shift VS Add | attend to argues | attend to attend+argues | 0.77 | 0.71 | 0.82 | **0.0000** | 386 | 167 | 71 | 390 | 69.80 | 15.40 | 30.20 | 84.60 |
| jl | Shift VS Add | argues to attend | argues to argues+attend | 0.69 | 0.61 | 0.76 | **0.0000** | 364 | 167 | 58 | 231 | 68.55 | 20.07 | 31.45 | 79.93 |
| gh | Shift VS Add | attend to temper | attend to attend+temper | 0.75 | 0.69 | 0.82 | **0.0000** | 225 | 108 | 60 | 337 | 67.57 | 15.11 | 32.43 | 84.89 |
| jl | Shift VS Add | temper to attend | temper to temper+attend | 0.69 | 0.62 | 0.77 | **0.0000** | 551 | 108 | 54 | 107 | 83.61 | 33.54 | 16.39 | 66.46 |
| gh | Shift VS Add | attend to fights | attend to attend+fights | 0.70 | 0.57 | 0.82 | **0.0000** | 51 | 41 | 21 | 159 | 55.43 | 11.67 | 44.57 | 88.33 |
| jl | Shift VS Add | fights to attend | fights to fights+attend | 0.71 | 0.58 | 0.83 | **0.0000** | 735 | 41 | 22 | 22 | 94.72 | 50.00 | 5.28 | 50.00 |
| gh | Shift VS Add | attend to steals | attend to attend+steals | 0.75 | 0.61 | 0.90 | **0.0000** | 26 | 22 | 12 | 140 | 54.17 | 7.89 | 45.83 | 92.11 |
| jl | Shift VS Add | steals to attend | steals to steals+attend | 0.81 | 0.68 | 0.95 | **0.0000** | 782 | 22 | 6 | 10 | 97.26 | 37.50 | 2.74 | 62.50 |
| gh | Shift VS Add | hyper to argues | hyper to hyper+argues | 0.77 | 0.72 | 0.83 | **0.0000** | 603 | 124 | 74 | 213 | 82.94 | 25.78 | 17.06 | 74.22 |
| jl | Shift VS Add | argues to hyper | argues to argues+hyper | 0.66 | 0.58 | 0.74 | **0.0000** | 219 | 124 | 62 | 260 | 63.85 | 19.25 | 36.15 | 80.75 |
| gh | Shift VS Add | hyper to temper | hyper to hyper+temper | 0.73 | 0.67 | 0.80 | **0.0000** | 349 | 93 | 76 | 212 | 78.96 | 26.39 | 21.04 | 73.61 |
| jl | Shift VS Add | temper to hyper | temper to temper+hyper | 0.67 | 0.58 | 0.75 | **0.0000** | 381 | 93 | 64 | 127 | 80.38 | 33.51 | 19.62 | 66.49 |
| gh | Shift VS Add | hyper to fights | hyper to hyper+fights | 0.82 | 0.74 | 0.91 | **0.0000** | 95 | 34 | 19 | 124 | 73.64 | 13.29 | 26.36 | 86.71 |
| jl | Shift VS Add | fights to hyper | fights to fights+hyper | 0.67 | 0.53 | 0.81 | **0.0000** | 581 | 34 | 28 | 22 | 94.47 | 56.00 | 5.53 | 44.00 |
| gh | Shift VS Add | hyper to steals | hyper to hyper+steals | 0.59 | 0.43 | 0.76 | **0.0000** | 48 | 30 | 27 | 95 | 61.54 | 22.13 | 38.46 | 77.87 |
| jl | Shift VS Add | steals to hyper | steals to steals+hyper | 0.83 | 0.72 | 0.93 | **0.0000** | 609 | 30 | 8 | 18 | 95.31 | 30.77 | 4.69 | 69.23 |
| gh | Shift VS Add | argues to temper | argues to argues+temper | 0.53 | 0.43 | 0.63 | **0.0000** | 143 | 178 | 59 | 350 | 44.55 | 14.43 | 55.45 | 85.57 |
| jl | Shift VS Add | temper to argues | temper to temper+argues | 0.55 | 0.46 | 0.64 | **0.0000** | 661 | 178 | 69 | 106 | 78.78 | 39.43 | 21.22 | 60.57 |
| gh | Shift VS Add | argues to fights | argues to argues+fights | 0.81 | 0.70 | 0.91 | **0.0000** | 42 | 44 | 8 | 178 | 48.84 | 4.30 | 51.16 | 95.70 |
| jl | Shift VS Add | fights to argues | fights to fights+argues | 0.69 | 0.56 | 0.82 | **0.0000** | 927 | 44 | 23 | 20 | 95.47 | 53.49 | 4.53 | 46.51 |
| gh | Shift VS Add | argues to steals | argues to argues+steals | 0.68 | 0.52 | 0.85 | **0.0000** | 28 | 39 | 9 | 124 | 41.79 | 6.77 | 58.21 | 93.23 |
| jl | Shift VS Add | steals to argues | steals to steals+argues | 0.71 | 0.57 | 0.84 | **0.0000** | 942 | 39 | 17 | 16 | 96.02 | 51.52 | 3.98 | 48.48 |
| gh | Shift VS Add | temper to fights | temper to temper+fights | 0.61 | 0.47 | 0.75 | **0.0000** | 65 | 54 | 25 | 128 | 54.62 | 16.34 | 45.38 | 83.66 |
| jl | Shift VS Add | fights to temper | fights to fights+temper | 0.70 | 0.58 | 0.82 | **0.0000** | 631 | 54 | 19 | 26 | 92.12 | 42.22 | 7.88 | 57.78 |
| gh | Shift VS Add | temper to steals | temper to temper+steals | 0.63 | 0.48 | 0.79 | **0.0000** | 69 | 38 | 20 | 73 | 64.49 | 21.51 | 35.51 | 78.49 |
| jl | Shift VS Add | steals to temper | steals to steals+temper | 0.79 | 0.69 | 0.90 | **0.0000** | 651 | 38 | 15 | 26 | 94.48 | 36.59 | 5.52 | 63.41 |
| gh | Shift VS Add | fights to steals | fights to fights+steals | 0.75 | 0.61 | 0.90 | **0.0000** | 134 | 33 | 7 | 26 | 80.24 | 21.21 | 19.76 | 78.79 |
| jl | Shift VS Add | steals to fights | steals to steals+fights | 0.65 | 0.47 | 0.83 | **0.0000** | 211 | 33 | 11 | 17 | 86.48 | 39.29 | 13.52 | 60.71 |
| fg | Shift VS Specific Persist | fear to worry | fear to fear | 0.53 | 0.44 | 0.62 | **0.0000** | 687 | 206 | 76 | 118 | 76.93 | 39.18 | 23.07 | 60.82 |
| jk | Shift VS Specific Persist | worry to fear | worry to worry | 0.33 | 0.24 | 0.42 | **0.0000** | 206 | 687 | 69 | 631 | 23.07 | 9.86 | 76.93 | 90.14 |
| fg | Shift VS Specific Persist | fear to dysph | fear to fear | 0.59 | 0.52 | 0.66 | **0.0000** | 302 | 575 | 47 | 646 | 34.44 | 6.78 | 65.56 | 93.22 |
| jk | Shift VS Specific Persist | dysph to fear | dysph to dysph | 0.59 | 0.50 | 0.67 | **0.0000** | 575 | 302 | 44 | 154 | 65.56 | 22.22 | 34.44 | 77.78 |
| fg | Shift VS Specific Persist | fear to worth | fear to fear | 0.56 | 0.49 | 0.63 | **0.0000** | 286 | 591 | 49 | 633 | 32.61 | 7.18 | 67.39 | 92.82 |
| jk | Shift VS Specific Persist | worth to fear | worth to worth | 0.64 | 0.56 | 0.71 | **0.0000** | 591 | 286 | 45 | 178 | 67.39 | 20.18 | 32.61 | 79.82 |
| fg | Shift VS Specific Persist | fear to attend | fear to fear | 0.66 | 0.59 | 0.72 | **0.0000** | 558 | 294 | 61 | 264 | 65.49 | 18.77 | 34.51 | 81.23 |
| jk | Shift VS Specific Persist | attend to fear | attend to attend | 0.74 | 0.70 | 0.79 | **0.0000** | 294 | 558 | 60 | 1894 | 34.51 | 3.07 | 65.49 | 96.93 |
| fg | Shift VS Specific Persist | fear to hyper | fear to fear | 0.67 | 0.61 | 0.73 | **0.0000** | 421 | 468 | 46 | 493 | 47.36 | 8.53 | 52.64 | 91.47 |
| jk | Shift VS Specific Persist | hyper to fear | hyper to hyper | 0.75 | 0.71 | 0.79 | **0.0000** | 468 | 421 | 83 | 996 | 52.64 | 7.69 | 47.36 | 92.31 |
| fg | Shift VS Specific Persist | fear to argues | fear to fear | 0.71 | 0.66 | 0.77 | **0.0000** | 695 | 310 | 63 | 303 | 69.15 | 17.21 | 30.85 | 82.79 |
| jk | Shift VS Specific Persist | argues to fear | argues to argues | 0.64 | 0.59 | 0.69 | **0.0000** | 310 | 695 | 79 | 1629 | 30.85 | 4.63 | 69.15 | 95.37 |
| fg | Shift VS Specific Persist | fear to temper | fear to fear | 0.67 | 0.61 | 0.73 | **0.0000** | 456 | 487 | 51 | 519 | 48.36 | 8.95 | 51.64 | 91.05 |
| jk | Shift VS Specific Persist | temper to fear | temper to temper | 0.65 | 0.60 | 0.71 | **0.0000** | 487 | 456 | 81 | 617 | 51.64 | 11.60 | 48.36 | 88.40 |
| fg | Shift VS Specific Persist | fear to fights | fear to fear | 0.62 | 0.53 | 0.70 | **0.0000** | 145 | 752 | 16 | 986 | 16.16 | 1.60 | 83.84 | 98.40 |
| jk | Shift VS Specific Persist | fights to fear | fights to fights | 0.80 | 0.75 | 0.86 | **0.0000** | 752 | 145 | 31 | 130 | 83.84 | 19.25 | 16.16 | 80.75 |
| fg | Shift VS Specific Persist | fear to steals | fear to fear | 0.67 | 0.59 | 0.75 | **0.0000** | 124 | 799 | 8 | 1072 | 13.43 | 0.74 | 86.57 | 99.26 |
| jk | Shift VS Specific Persist | steals to fear | steals to steals | 0.84 | 0.79 | 0.90 | **0.0000** | 799 | 124 | 20 | 103 | 86.57 | 16.26 | 13.43 | 83.74 |
| fg | Shift VS Specific Persist | worry to dysph | worry to worry | 0.38 | 0.30 | 0.47 | **0.0000** | 161 | 887 | 60 | 1195 | 15.36 | 4.78 | 84.64 | 95.22 |
| jk | Shift VS Specific Persist | dysph to worry | dysph to dysph | 0.56 | 0.46 | 0.66 | **0.0000** | 887 | 161 | 53 | 65 | 84.64 | 44.92 | 15.36 | 55.08 |
| fg | Shift VS Specific Persist | worry to worth | worry to worry | 0.39 | 0.32 | 0.47 | **0.0000** | 168 | 898 | 61 | 1220 | 15.76 | 4.76 | 84.24 | 95.24 |
| jk | Shift VS Specific Persist | worth to worry | worth to worth | 0.60 | 0.51 | 0.69 | **0.0000** | 898 | 168 | 55 | 80 | 84.24 | 40.74 | 15.76 | 59.26 |
| fg | Shift VS Specific Persist | worry to attend | worry to worry | 0.66 | 0.60 | 0.71 | **0.0000** | 409 | 458 | 63 | 602 | 47.17 | 9.47 | 52.83 | 90.53 |
| jk | Shift VS Specific Persist | attend to worry | attend to attend | 0.79 | 0.76 | 0.83 | **0.0000** | 458 | 409 | 91 | 1471 | 52.83 | 5.83 | 47.17 | 94.17 |
| fg | Shift VS Specific Persist | worry to hyper | worry to worry | 0.64 | 0.58 | 0.70 | **0.0000** | 332 | 709 | 49 | 991 | 31.89 | 4.71 | 68.11 | 95.29 |
| jk | Shift VS Specific Persist | hyper to worry | hyper to hyper | 0.79 | 0.75 | 0.82 | **0.0000** | 709 | 332 | 112 | 784 | 68.11 | 12.50 | 31.89 | 87.50 |
| fg | Shift VS Specific Persist | worry to argues | worry to worry | 0.64 | 0.59 | 0.70 | **0.0000** | 529 | 493 | 82 | 599 | 51.76 | 12.04 | 48.24 | 87.96 |
| jk | Shift VS Specific Persist | argues to worry | argues to argues | 0.70 | 0.65 | 0.74 | **0.0000** | 493 | 529 | 106 | 1146 | 48.24 | 8.47 | 51.76 | 91.53 |
| fg | Shift VS Specific Persist | worry to temper | worry to worry | 0.58 | 0.52 | 0.64 | **0.0000** | 341 | 749 | 68 | 1027 | 31.28 | 6.21 | 68.72 | 93.79 |
| jk | Shift VS Specific Persist | temper to worry | temper to temper | 0.68 | 0.63 | 0.73 | **0.0000** | 749 | 341 | 112 | 440 | 68.72 | 20.29 | 31.28 | 79.71 |
| fg | Shift VS Specific Persist | worry to fights | worry to worry | 0.54 | 0.46 | 0.63 | **0.0000** | 107 | 1052 | 19 | 1736 | 9.23 | 1.08 | 90.77 | 98.92 |
| jk | Shift VS Specific Persist | fights to worry | fights to fights | 0.80 | 0.75 | 0.86 | **0.0000** | 1052 | 107 | 43 | 103 | 90.77 | 29.45 | 9.23 | 70.55 |
| fg | Shift VS Specific Persist | worry to steals | worry to worry | 0.53 | 0.44 | 0.61 | **0.0000** | 95 | 1100 | 18 | 1861 | 7.95 | 0.96 | 92.05 | 99.04 |
| jk | Shift VS Specific Persist | steals to worry | steals to steals | 0.86 | 0.81 | 0.91 | **0.0000** | 1100 | 95 | 17 | 72 | 92.05 | 19.10 | 7.95 | 80.90 |
| fg | Shift VS Specific Persist | dysph to worth | dysph to dysph | 0.56 | 0.46 | 0.65 | **0.0000** | 336 | 358 | 24 | 164 | 48.41 | 12.77 | 51.59 | 87.23 |
| jk | Shift VS Specific Persist | worth to dysph | worth to worth | 0.56 | 0.47 | 0.65 | **0.0000** | 358 | 336 | 34 | 192 | 51.59 | 15.04 | 48.41 | 84.96 |
| fg | Shift VS Specific Persist | dysph to attend | dysph to dysph | 0.68 | 0.59 | 0.76 | **0.0000** | 669 | 184 | 27 | 84 | 78.43 | 24.32 | 21.57 | 75.68 |
| jk | Shift VS Specific Persist | attend to dysph | attend to attend | 0.71 | 0.66 | 0.76 | **0.0000** | 184 | 669 | 37 | 2411 | 21.57 | 1.51 | 78.43 | 98.49 |
| fg | Shift VS Specific Persist | dysph to hyper | dysph to dysph | 0.69 | 0.62 | 0.76 | **0.0000** | 500 | 309 | 31 | 206 | 61.80 | 13.08 | 38.20 | 86.92 |
| jk | Shift VS Specific Persist | hyper to dysph | hyper to hyper | 0.71 | 0.66 | 0.76 | **0.0000** | 309 | 500 | 67 | 1312 | 38.20 | 4.86 | 61.80 | 95.14 |
| fg | Shift VS Specific Persist | dysph to argues | dysph to dysph | 0.73 | 0.65 | 0.81 | **0.0000** | 827 | 177 | 21 | 77 | 82.37 | 21.43 | 17.63 | 78.57 |
| jk | Shift VS Specific Persist | argues to dysph | argues to argues | 0.54 | 0.48 | 0.61 | **0.0000** | 177 | 827 | 59 | 1939 | 17.63 | 2.95 | 82.37 | 97.05 |
| fg | Shift VS Specific Persist | dysph to temper | dysph to dysph | 0.62 | 0.54 | 0.70 | **0.0000** | 533 | 317 | 32 | 154 | 62.71 | 17.20 | 37.29 | 82.80 |
| jk | Shift VS Specific Persist | temper to dysph | temper to temper | 0.64 | 0.58 | 0.70 | **0.0000** | 317 | 533 | 55 | 786 | 37.29 | 6.54 | 62.71 | 93.46 |
| fg | Shift VS Specific Persist | dysph to fights | dysph to dysph | 0.58 | 0.48 | 0.68 | **0.0000** | 161 | 562 | 11 | 371 | 22.27 | 2.88 | 77.73 | 97.12 |
| jk | Shift VS Specific Persist | fights to dysph | fights to fights | 0.75 | 0.69 | 0.82 | **0.0000** | 562 | 161 | 30 | 130 | 77.73 | 18.75 | 22.27 | 81.25 |
| fg | Shift VS Specific Persist | dysph to steals | dysph to dysph | 0.59 | 0.48 | 0.69 | **0.0000** | 115 | 589 | 8 | 454 | 16.34 | 1.73 | 83.66 | 98.27 |
| jk | Shift VS Specific Persist | steals to dysph | steals to steals | 0.85 | 0.80 | 0.91 | **0.0000** | 589 | 115 | 18 | 116 | 83.66 | 13.43 | 16.34 | 86.57 |
| fg | Shift VS Specific Persist | worth to attend | worth to worth | 0.77 | 0.70 | 0.84 | **0.0000** | 679 | 162 | 19 | 90 | 80.74 | 17.43 | 19.26 | 82.57 |
| jk | Shift VS Specific Persist | attend to worth | attend to attend | 0.63 | 0.57 | 0.69 | **0.0000** | 162 | 679 | 50 | 2370 | 19.26 | 2.07 | 80.74 | 97.93 |
| fg | Shift VS Specific Persist | worth to hyper | worth to worth | 0.72 | 0.65 | 0.78 | **0.0000** | 516 | 304 | 28 | 213 | 62.93 | 11.62 | 37.07 | 88.38 |
| jk | Shift VS Specific Persist | hyper to worth | hyper to hyper | 0.69 | 0.64 | 0.74 | **0.0000** | 304 | 516 | 66 | 1277 | 37.07 | 4.91 | 62.93 | 95.09 |
| fg | Shift VS Specific Persist | worth to argues | worth to worth | 0.69 | 0.61 | 0.76 | **0.0000** | 817 | 165 | 45 | 101 | 83.20 | 30.82 | 16.80 | 69.18 |
| jk | Shift VS Specific Persist | argues to worth | argues to argues | 0.54 | 0.47 | 0.61 | **0.0000** | 165 | 817 | 56 | 1942 | 16.80 | 2.80 | 83.20 | 97.20 |
| fg | Shift VS Specific Persist | worth to temper | worth to worth | 0.66 | 0.59 | 0.73 | **0.0000** | 541 | 295 | 39 | 193 | 64.71 | 16.81 | 35.29 | 83.19 |
| jk | Shift VS Specific Persist | temper to worth | temper to temper | 0.60 | 0.54 | 0.67 | **0.0000** | 295 | 541 | 59 | 787 | 35.29 | 6.97 | 64.71 | 93.03 |
| fg | Shift VS Specific Persist | worth to fights | worth to worth | 0.56 | 0.47 | 0.65 | **0.0000** | 167 | 519 | 19 | 448 | 24.34 | 4.07 | 75.66 | 95.93 |
| jk | Shift VS Specific Persist | fights to worth | fights to fights | 0.74 | 0.68 | 0.81 | **0.0000** | 519 | 167 | 38 | 161 | 75.66 | 19.10 | 24.34 | 80.90 |
| fg | Shift VS Specific Persist | worth to steals | worth to worth | 0.59 | 0.49 | 0.69 | **0.0000** | 124 | 562 | 11 | 507 | 18.08 | 2.12 | 81.92 | 97.88 |
| jk | Shift VS Specific Persist | steals to worth | steals to steals | 0.83 | 0.77 | 0.89 | **0.0000** | 562 | 124 | 19 | 112 | 81.92 | 14.50 | 18.08 | 85.50 |
| fg | Shift VS Specific Persist | attend to hyper | attend to attend | 0.52 | 0.43 | 0.61 | **0.0000** | 118 | 513 | 35 | 936 | 18.70 | 3.60 | 81.30 | 96.40 |
| jk | Shift VS Specific Persist | hyper to attend | hyper to hyper | 0.66 | 0.57 | 0.76 | **0.0000** | 513 | 118 | 39 | 83 | 81.30 | 31.97 | 18.70 | 68.03 |
| fg | Shift VS Specific Persist | attend to argues | attend to attend | 0.76 | 0.71 | 0.80 | **0.0000** | 386 | 364 | 71 | 948 | 51.47 | 6.97 | 48.53 | 93.03 |
| jk | Shift VS Specific Persist | argues to attend | argues to argues | 0.71 | 0.66 | 0.76 | **0.0000** | 364 | 386 | 58 | 680 | 48.53 | 7.86 | 51.47 | 92.14 |
| fg | Shift VS Specific Persist | attend to temper | attend to attend | 0.68 | 0.63 | 0.73 | **0.0000** | 225 | 551 | 60 | 1760 | 28.99 | 3.30 | 71.01 | 96.70 |
| jk | Shift VS Specific Persist | temper to attend | temper to temper | 0.73 | 0.67 | 0.78 | **0.0000** | 551 | 225 | 54 | 249 | 71.01 | 17.82 | 28.99 | 82.18 |
| fg | Shift VS Specific Persist | attend to fights | attend to attend | 0.52 | 0.42 | 0.62 | **0.0000** | 51 | 735 | 21 | 2835 | 6.49 | 0.74 | 93.51 | 99.26 |
| jk | Shift VS Specific Persist | fights to attend | fights to fights | 0.76 | 0.66 | 0.86 | **0.0000** | 735 | 51 | 22 | 33 | 93.51 | 40.00 | 6.49 | 60.00 |
| fg | Shift VS Specific Persist | attend to steals | attend to attend | 0.46 | 0.33 | 0.59 | **0.0000** | 26 | 782 | 12 | 3016 | 3.22 | 0.40 | 96.78 | 99.60 |
| jk | Shift VS Specific Persist | steals to attend | steals to steals | 0.86 | 0.77 | 0.96 | **0.0000** | 782 | 26 | 6 | 16 | 96.78 | 27.27 | 3.22 | 72.73 |
| fg | Shift VS Specific Persist | hyper to argues | hyper to hyper | 0.79 | 0.75 | 0.83 | **0.0000** | 603 | 219 | 74 | 412 | 73.36 | 15.23 | 26.64 | 84.77 |
| jk | Shift VS Specific Persist | argues to hyper | argues to argues | 0.57 | 0.51 | 0.64 | **0.0000** | 219 | 603 | 62 | 1186 | 26.64 | 4.97 | 73.36 | 95.03 |
| fg | Shift VS Specific Persist | hyper to temper | hyper to hyper | 0.69 | 0.64 | 0.75 | **0.0000** | 349 | 381 | 76 | 827 | 47.81 | 8.42 | 52.19 | 91.58 |
| jk | Shift VS Specific Persist | temper to hyper | temper to temper | 0.65 | 0.59 | 0.71 | **0.0000** | 381 | 349 | 64 | 460 | 52.19 | 12.21 | 47.81 | 87.79 |
| fg | Shift VS Specific Persist | hyper to fights | hyper to hyper | 0.63 | 0.55 | 0.71 | **0.0000** | 95 | 581 | 19 | 1480 | 14.05 | 1.27 | 85.95 | 98.73 |
| jk | Shift VS Specific Persist | fights to hyper | fights to fights | 0.74 | 0.66 | 0.83 | **0.0000** | 581 | 95 | 28 | 70 | 85.95 | 28.57 | 14.05 | 71.43 |
| fg | Shift VS Specific Persist | hyper to steals | hyper to hyper | 0.40 | 0.29 | 0.51 | **0.0000** | 48 | 609 | 27 | 1618 | 7.31 | 1.64 | 92.69 | 98.36 |
| jk | Shift VS Specific Persist | steals to hyper | steals to steals | 0.90 | 0.84 | 0.95 | **0.0000** | 609 | 48 | 8 | 45 | 92.69 | 15.09 | 7.31 | 84.91 |
| fg | Shift VS Specific Persist | argues to temper | argues to argues | 0.41 | 0.33 | 0.49 | **0.0000** | 143 | 661 | 59 | 1050 | 17.79 | 5.32 | 82.21 | 94.68 |
| jk | Shift VS Specific Persist | temper to argues | temper to temper | 0.60 | 0.52 | 0.69 | **0.0000** | 661 | 143 | 69 | 103 | 82.21 | 40.12 | 17.79 | 59.88 |
| fg | Shift VS Specific Persist | argues to fights | argues to argues | 0.55 | 0.43 | 0.66 | **0.0000** | 42 | 927 | 8 | 2242 | 4.33 | 0.36 | 95.67 | 99.64 |
| jk | Shift VS Specific Persist | fights to argues | fights to fights | 0.60 | 0.43 | 0.76 | **0.0000** | 927 | 42 | 23 | 13 | 95.67 | 63.89 | 4.33 | 36.11 |
| fg | Shift VS Specific Persist | argues to steals | argues to argues | 0.46 | 0.33 | 0.59 | **0.0000** | 28 | 942 | 9 | 2515 | 2.89 | 0.36 | 97.11 | 99.64 |
| jk | Shift VS Specific Persist | steals to argues | steals to steals | 0.80 | 0.69 | 0.90 | **0.0000** | 942 | 28 | 17 | 20 | 97.11 | 45.95 | 2.89 | 54.05 |
| fg | Shift VS Specific Persist | temper to fights | temper to temper | 0.36 | 0.24 | 0.47 | **0.0000** | 65 | 631 | 25 | 875 | 9.34 | 2.78 | 90.66 | 97.22 |
| jk | Shift VS Specific Persist | fights to temper | fights to fights | 0.67 | 0.54 | 0.80 | **0.0000** | 631 | 65 | 19 | 26 | 90.66 | 42.22 | 9.34 | 57.78 |
| fg | Shift VS Specific Persist | temper to steals | temper to temper | 0.47 | 0.36 | 0.57 | **0.0000** | 69 | 651 | 20 | 1138 | 9.58 | 1.73 | 90.42 | 98.27 |
| jk | Shift VS Specific Persist | steals to temper | steals to steals | 0.83 | 0.76 | 0.90 | **0.0000** | 651 | 69 | 15 | 52 | 90.42 | 22.39 | 9.58 | 77.61 |
| fg | Shift VS Specific Persist | fights to steals | fights to fights | 0.74 | 0.64 | 0.83 | **0.0000** | 134 | 211 | 7 | 207 | 38.84 | 3.27 | 61.16 | 96.73 |
| jk | Shift VS Specific Persist | steals to fights | steals to steals | 0.75 | 0.66 | 0.84 | **0.0000** | 211 | 134 | 11 | 112 | 61.16 | 8.94 | 38.84 | 91.06 |
| op | Subtract x VS Joint Persist | fear+worry to worry | fear+worry to fear+worry | 0.66 | 0.61 | 0.70 | **0.0000** | 687 | 270 | 293 | 836 | 71.79 | 25.95 | 28.21 | 74.05 |
| op | Subtract x VS Joint Persist | fear+dysph to dysph | fear+dysph to fear+dysph | 0.66 | 0.59 | 0.74 | **0.0000** | 302 | 170 | 62 | 268 | 63.98 | 18.79 | 36.02 | 81.21 |
| op | Subtract x VS Joint Persist | fear+worth to worth | fear+worth to fear+worth | 0.66 | 0.58 | 0.73 | **0.0000** | 286 | 157 | 73 | 294 | 64.56 | 19.89 | 35.44 | 80.11 |
| op | Subtract x VS Joint Persist | fear+attend to attend | fear+attend to fear+attend | 0.71 | 0.66 | 0.75 | **0.0000** | 558 | 109 | 400 | 768 | 83.66 | 34.25 | 16.34 | 65.75 |
| op | Subtract x VS Joint Persist | fear+hyper to hyper | fear+hyper to fear+hyper | 0.72 | 0.66 | 0.77 | **0.0000** | 421 | 88 | 231 | 477 | 82.71 | 32.63 | 17.29 | 67.37 |
| op | Subtract x VS Joint Persist | fear+argues to argues | fear+argues to fear+argues | 0.73 | 0.69 | 0.77 | **0.0000** | 695 | 137 | 310 | 640 | 83.53 | 32.63 | 16.47 | 67.37 |
| op | Subtract x VS Joint Persist | fear+temper to temper | fear+temper to fear+temper | 0.75 | 0.70 | 0.80 | **0.0000** | 456 | 111 | 156 | 421 | 80.42 | 27.04 | 19.58 | 72.96 |
| op | Subtract x VS Joint Persist | fear+fights to fights | fear+fights to fear+fights | 0.66 | 0.55 | 0.77 | **0.0000** | 145 | 53 | 38 | 102 | 73.23 | 27.14 | 26.77 | 72.86 |
| op | Subtract x VS Joint Persist | fear+steals to steals | fear+steals to fear+steals | 0.73 | 0.61 | 0.85 | **0.0000** | 124 | 26 | 27 | 60 | 82.67 | 31.03 | 17.33 | 68.97 |
| op | Subtract x VS Joint Persist | worry+dysph to dysph | worry+dysph to worry+dysph | 0.58 | 0.49 | 0.67 | **0.0000** | 161 | 177 | 60 | 381 | 47.63 | 13.61 | 52.37 | 86.39 |
| op | Subtract x VS Joint Persist | worry+worth to worth | worry+worth to worry+worth | 0.58 | 0.50 | 0.67 | **0.0000** | 168 | 156 | 76 | 399 | 51.85 | 16.00 | 48.15 | 84.00 |
| op | Subtract x VS Joint Persist | worry+attend to attend | worry+attend to worry+attend | 0.60 | 0.54 | 0.65 | **0.0000** | 409 | 169 | 438 | 1102 | 70.76 | 28.44 | 29.24 | 71.56 |
| op | Subtract x VS Joint Persist | worry+hyper to hyper | worry+hyper to worry+hyper | 0.69 | 0.63 | 0.74 | **0.0000** | 332 | 101 | 246 | 651 | 76.67 | 27.42 | 23.33 | 72.58 |
| op | Subtract x VS Joint Persist | worry+argues to argues | worry+argues to worry+argues | 0.68 | 0.64 | 0.73 | **0.0000** | 529 | 173 | 367 | 1006 | 75.36 | 26.73 | 24.64 | 73.27 |
| op | Subtract x VS Joint Persist | worry+temper to temper | worry+temper to worry+temper | 0.74 | 0.69 | 0.79 | **0.0000** | 341 | 123 | 153 | 600 | 73.49 | 20.32 | 26.51 | 79.68 |
| op | Subtract x VS Joint Persist | worry+fights to fights | worry+fights to worry+fights | 0.71 | 0.61 | 0.82 | **0.0000** | 107 | 51 | 31 | 140 | 67.72 | 18.13 | 32.28 | 81.87 |
| op | Subtract x VS Joint Persist | worry+steals to steals | worry+steals to worry+steals | 0.78 | 0.68 | 0.88 | **0.0000** | 95 | 31 | 21 | 94 | 75.40 | 18.26 | 24.60 | 81.74 |
| op | Subtract x VS Joint Persist | dysph+worth to worth | dysph+worth to dysph+worth | 0.53 | 0.45 | 0.62 | **0.0000** | 336 | 193 | 93 | 243 | 63.52 | 27.68 | 36.48 | 72.32 |
| op | Subtract x VS Joint Persist | dysph+attend to attend | dysph+attend to dysph+attend | 0.71 | 0.66 | 0.76 | **0.0000** | 669 | 87 | 299 | 388 | 88.49 | 43.52 | 11.51 | 56.48 |
| op | Subtract x VS Joint Persist | dysph+hyper to hyper | dysph+hyper to dysph+hyper | 0.66 | 0.59 | 0.73 | **0.0000** | 500 | 95 | 170 | 245 | 84.03 | 40.96 | 15.97 | 59.04 |
| op | Subtract x VS Joint Persist | dysph+argues to argues | dysph+argues to dysph+argues | 0.73 | 0.68 | 0.77 | **0.0000** | 827 | 112 | 267 | 386 | 88.07 | 40.89 | 11.93 | 59.11 |
| op | Subtract x VS Joint Persist | dysph+temper to temper | dysph+temper to dysph+temper | 0.71 | 0.66 | 0.77 | **0.0000** | 533 | 125 | 129 | 288 | 81.00 | 30.94 | 19.00 | 69.06 |
| op | Subtract x VS Joint Persist | dysph+fights to fights | dysph+fights to dysph+fights | 0.64 | 0.53 | 0.76 | **0.0000** | 161 | 53 | 43 | 97 | 75.23 | 30.71 | 24.77 | 69.29 |
| op | Subtract x VS Joint Persist | dysph+steals to steals | dysph+steals to dysph+steals | 0.54 | 0.37 | 0.71 | **0.0000** | 115 | 45 | 25 | 47 | 71.88 | 34.72 | 28.13 | 65.28 |
| op | Subtract x VS Joint Persist | worth+attend to attend | worth+attend to worth+attend | 0.74 | 0.69 | 0.78 | **0.0000** | 679 | 85 | 310 | 447 | 88.87 | 40.95 | 11.13 | 59.05 |
| op | Subtract x VS Joint Persist | worth+hyper to hyper | worth+hyper to worth+hyper | 0.74 | 0.69 | 0.80 | **0.0000** | 516 | 72 | 177 | 284 | 87.76 | 38.39 | 12.24 | 61.61 |
| op | Subtract x VS Joint Persist | worth+argues to argues | worth+argues to worth+argues | 0.77 | 0.73 | 0.81 | **0.0000** | 817 | 92 | 256 | 399 | 89.88 | 39.08 | 10.12 | 60.92 |
| op | Subtract x VS Joint Persist | worth+temper to temper | worth+temper to worth+temper | 0.78 | 0.73 | 0.83 | **0.0000** | 541 | 86 | 124 | 275 | 86.28 | 31.08 | 13.72 | 68.92 |
| op | Subtract x VS Joint Persist | worth+fights to fights | worth+fights to worth+fights | 0.70 | 0.59 | 0.82 | **0.0000** | 167 | 41 | 31 | 71 | 80.29 | 30.39 | 19.71 | 69.61 |
| op | Subtract x VS Joint Persist | worth+steals to steals | worth+steals to worth+steals | 0.60 | 0.45 | 0.75 | **0.0000** | 124 | 34 | 30 | 49 | 78.48 | 37.97 | 21.52 | 62.03 |
| op | Subtract x VS Joint Persist | attend+hyper to hyper | attend+hyper to attend+hyper | 0.69 | 0.62 | 0.75 | **0.0000** | 118 | 186 | 86 | 1686 | 38.82 | 4.85 | 61.18 | 95.15 |
| op | Subtract x VS Joint Persist | attend+argues to argues | attend+argues to attend+argues | 0.75 | 0.71 | 0.79 | **0.0000** | 386 | 167 | 306 | 1708 | 69.80 | 15.19 | 30.20 | 84.81 |
| op | Subtract x VS Joint Persist | attend+temper to temper | attend+temper to attend+temper | 0.79 | 0.74 | 0.83 | **0.0000** | 225 | 108 | 120 | 911 | 67.57 | 11.64 | 32.43 | 88.36 |
| op | Subtract x VS Joint Persist | attend+fights to fights | attend+fights to attend+fights | 0.75 | 0.65 | 0.86 | **0.0000** | 51 | 41 | 21 | 230 | 55.43 | 8.37 | 44.57 | 91.63 |
| op | Subtract x VS Joint Persist | attend+steals to steals | attend+steals to attend+steals | 0.71 | 0.56 | 0.86 | **0.0000** | 26 | 22 | 18 | 173 | 54.17 | 9.42 | 45.83 | 90.58 |
| op | Subtract x VS Joint Persist | hyper+argues to argues | hyper+argues to hyper+argues | 0.77 | 0.73 | 0.80 | **0.0000** | 603 | 124 | 394 | 1085 | 82.94 | 26.64 | 17.06 | 73.36 |
| op | Subtract x VS Joint Persist | hyper+temper to temper | hyper+temper to hyper+temper | 0.79 | 0.74 | 0.83 | **0.0000** | 349 | 93 | 164 | 636 | 78.96 | 20.50 | 21.04 | 79.50 |
| op | Subtract x VS Joint Persist | hyper+fights to fights | hyper+fights to hyper+fights | 0.80 | 0.71 | 0.88 | **0.0000** | 95 | 34 | 33 | 181 | 73.64 | 15.42 | 26.36 | 84.58 |
| op | Subtract x VS Joint Persist | hyper+steals to steals | hyper+steals to hyper+steals | 0.63 | 0.48 | 0.78 | **0.0000** | 48 | 30 | 30 | 124 | 61.54 | 19.48 | 38.46 | 80.52 |
| op | Subtract x VS Joint Persist | argues+temper to temper | argues+temper to argues+temper | 0.61 | 0.54 | 0.68 | **0.0000** | 143 | 178 | 118 | 1060 | 44.55 | 10.02 | 55.45 | 89.98 |
| Op | Subtract x VS Joint Persist | argues+fights to fights | argues+fights to argues+fights | 0.75 | 0.64 | 0.86 | **0.0000** | 42 | 44 | 17 | 256 | 48.84 | 6.23 | 51.16 | 93.77 |
| Op | Subtract x VS Joint Persist | argues+steals to steals | argues+steals to argues+steals | 0.62 | 0.45 | 0.79 | **0.0000** | 28 | 39 | 16 | 165 | 41.79 | 8.84 | 58.21 | 91.16 |
| Op | Subtract x VS Joint Persist | temper+fights to fights | temper+fights to temper+fights | 0.65 | 0.53 | 0.77 | **0.0000** | 65 | 54 | 35 | 219 | 54.62 | 13.78 | 45.38 | 86.22 |
| Op | Subtract x VS Joint Persist | temper+steals to steals | temper+steals to temper+steals | 0.63 | 0.50 | 0.77 | **0.0000** | 69 | 38 | 30 | 109 | 64.49 | 21.58 | 35.51 | 78.42 |
| Op | Subtract x VS Joint Persist | fights+steals to steals | fights+steals to fights+steals | 0.60 | 0.45 | 0.74 | **0.0000** | 134 | 33 | 36 | 52 | 80.24 | 40.91 | 19.76 | 59.09 |
| np | Subtract y VS Joint Persist | worry+fear to fear | worry+fear to worry+fear | 0.57 | 0.50 | 0.64 | **0.0000** | 206 | 270 | 113 | 836 | 43.28 | 11.91 | 56.72 | 88.09 |
| np | Subtract y VS Joint Persist | dysph+fear to fear | dysph+fear to dysph+fear | 0.63 | 0.57 | 0.70 | **0.0000** | 575 | 170 | 136 | 268 | 77.18 | 33.66 | 22.82 | 66.34 |
| np | Subtract y VS Joint Persist | worth+fear to fear | worth+fear to worth+fear | 0.68 | 0.62 | 0.74 | **0.0000** | 591 | 157 | 136 | 294 | 79.01 | 31.63 | 20.99 | 68.37 |
| np | Subtract y VS Joint Persist | attend+fear to fear | attend+fear to attend+fear | 0.85 | 0.81 | 0.89 | **0.0000** | 294 | 109 | 88 | 768 | 72.95 | 10.28 | 27.05 | 89.72 |
| np | Subtract y VS Joint Persist | hyper+fear to fear | hyper+fear to hyper+fear | 0.83 | 0.79 | 0.87 | **0.0000** | 468 | 88 | 132 | 477 | 84.17 | 21.67 | 15.83 | 78.33 |
| np | Subtract y VS Joint Persist | argues+fear to fear | argues+fear to argues+fear | 0.71 | 0.66 | 0.77 | **0.0000** | 310 | 137 | 150 | 640 | 69.35 | 18.99 | 30.65 | 81.01 |
| np | Subtract y VS Joint Persist | temper+fear to fear | temper+fear to temper+fear | 0.75 | 0.70 | 0.80 | **0.0000** | 487 | 111 | 160 | 421 | 81.44 | 27.54 | 18.56 | 72.46 |
| np | Subtract y VS Joint Persist | fights+fear to fear | fights+fear to fights+fear | 0.81 | 0.75 | 0.87 | **0.0000** | 752 | 53 | 66 | 102 | 93.42 | 39.29 | 6.58 | 60.71 |
| np | Subtract y VS Joint Persist | steals+fear to fear | steals+fear to steals+fear | 0.88 | 0.82 | 0.93 | **0.0000** | 799 | 26 | 38 | 60 | 96.85 | 38.78 | 3.15 | 61.22 |
| np | Subtract y VS Joint Persist | dysph+worry to worry | dysph+worry to dysph+worry | 0.68 | 0.63 | 0.73 | **0.0000** | 887 | 177 | 228 | 381 | 83.36 | 37.44 | 16.64 | 62.56 |
| np | Subtract y VS Joint Persist | worth+worry to worry | worth+worry to worth+worry | 0.73 | 0.69 | 0.78 | **0.0000** | 898 | 156 | 212 | 399 | 85.20 | 34.70 | 14.80 | 65.30 |
| np | Subtract y VS Joint Persist | attend+worry to worry | attend+worry to attend+worry | 0.79 | 0.75 | 0.82 | **0.0000** | 458 | 169 | 204 | 1102 | 73.05 | 15.62 | 26.95 | 84.38 |
| np | Subtract y VS Joint Persist | hyper+worry to worry | hyper+worry to hyper+worry | 0.81 | 0.78 | 0.85 | **0.0000** | 709 | 101 | 262 | 651 | 87.53 | 28.70 | 12.47 | 71.30 |
| np | Subtract y VS Joint Persist | argues+worry to worry | argues+worry to argues+worry | 0.75 | 0.70 | 0.79 | **0.0000** | 493 | 173 | 252 | 1006 | 74.02 | 20.03 | 25.98 | 79.97 |
| np | Subtract y VS Joint Persist | temper+worry to worry | temper+worry to temper+worry | 0.78 | 0.74 | 0.82 | **0.0000** | 749 | 123 | 262 | 600 | 85.89 | 30.39 | 14.11 | 69.61 |
| np | Subtract y VS Joint Persist | fights+worry to worry | fights+worry to fights+worry | 0.82 | 0.78 | 0.87 | **0.0000** | 1052 | 51 | 116 | 140 | 95.38 | 45.31 | 4.62 | 54.69 |
| np | Subtract y VS Joint Persist | steals+worry to worry | steals+worry to steals+worry | 0.87 | 0.82 | 0.92 | **0.0000** | 1100 | 31 | 76 | 94 | 97.26 | 44.71 | 2.74 | 55.29 |
| np | Subtract y VS Joint Persist | worth+dysph to dysph | worth+dysph to worth+dysph | 0.61 | 0.53 | 0.69 | **0.0000** | 358 | 193 | 72 | 243 | 64.97 | 22.86 | 35.03 | 77.14 |
| np | Subtract y VS Joint Persist | attend+dysph to dysph | attend+dysph to attend+dysph | 0.78 | 0.72 | 0.84 | **0.0000** | 184 | 87 | 58 | 388 | 67.90 | 13.00 | 32.10 | 87.00 |
| np | Subtract y VS Joint Persist | hyper+dysph to dysph | hyper+dysph to hyper+dysph | 0.73 | 0.67 | 0.80 | **0.0000** | 309 | 95 | 77 | 245 | 76.49 | 23.91 | 23.51 | 76.09 |
| np | Subtract y VS Joint Persist | argues+dysph to dysph | argues+dysph to argues+dysph | 0.75 | 0.69 | 0.82 | **0.0000** | 177 | 112 | 50 | 386 | 61.25 | 11.47 | 38.75 | 88.53 |
| np | Subtract y VS Joint Persist | temper+dysph to dysph | temper+dysph to temper+dysph | 0.70 | 0.63 | 0.76 | **0.0000** | 317 | 125 | 85 | 288 | 71.72 | 22.79 | 28.28 | 77.21 |
| np | Subtract y VS Joint Persist | fights+dysph to dysph | fights+dysph to fights+dysph | 0.81 | 0.74 | 0.87 | **0.0000** | 562 | 53 | 52 | 97 | 91.38 | 34.90 | 8.62 | 65.10 |
| np | Subtract y VS Joint Persist | steals+dysph to dysph | steals+dysph to steals+dysph | 0.76 | 0.67 | 0.85 | **0.0000** | 589 | 45 | 34 | 47 | 92.90 | 41.98 | 7.10 | 58.02 |
| np | Subtract y VS Joint Persist | attend+worth to worth | attend+worth to attend+worth | 0.80 | 0.75 | 0.86 | **0.0000** | 162 | 85 | 50 | 447 | 65.59 | 10.06 | 34.41 | 89.94 |
| np | Subtract y VS Joint Persist | hyper+worth to worth | hyper+worth to hyper+worth | 0.80 | 0.75 | 0.86 | **0.0000** | 304 | 72 | 78 | 284 | 80.85 | 21.55 | 19.15 | 78.45 |
| np | Subtract y VS Joint Persist | argues+worth to worth | argues+worth to argues+worth | 0.74 | 0.67 | 0.81 | **0.0000** | 165 | 92 | 64 | 399 | 64.20 | 13.82 | 35.80 | 86.18 |
| np | Subtract y VS Joint Persist | temper+worth to worth | temper+worth to temper+worth | 0.73 | 0.67 | 0.80 | **0.0000** | 295 | 86 | 92 | 275 | 77.43 | 25.07 | 22.57 | 74.93 |
| np | Subtract y VS Joint Persist | fights+worth to worth | fights+worth to fights+worth | 0.76 | 0.67 | 0.84 | **0.0000** | 519 | 41 | 60 | 71 | 92.68 | 45.80 | 7.32 | 54.20 |
| np | Subtract y VS Joint Persist | steals+worth to worth | steals+worth to steals+worth | 0.79 | 0.71 | 0.88 | **0.0000** | 562 | 34 | 37 | 49 | 94.30 | 43.02 | 5.70 | 56.98 |
| np | Subtract y VS Joint Persist | hyper+attend to attend | hyper+attend to hyper+attend | 0.68 | 0.64 | 0.72 | **0.0000** | 513 | 186 | 523 | 1686 | 73.39 | 23.68 | 26.61 | 76.32 |
| np | Subtract y VS Joint Persist | argues+attend to attend | argues+attend to argues+attend | 0.66 | 0.62 | 0.71 | **0.0000** | 364 | 167 | 425 | 1708 | 68.55 | 19.92 | 31.45 | 80.08 |
| np | Subtract y VS Joint Persist | temper+attend to attend | temper+attend to temper+attend | 0.71 | 0.66 | 0.75 | **0.0000** | 551 | 108 | 463 | 911 | 83.61 | 33.70 | 16.39 | 66.30 |
| np | Subtract y VS Joint Persist | fights+attend to attend | fights+attend to fights+attend | 0.78 | 0.72 | 0.83 | **0.0000** | 735 | 41 | 247 | 230 | 94.72 | 51.78 | 5.28 | 48.22 |
| np | Subtract y VS Joint Persist | steals+attend to attend | steals+attend to steals+attend | 0.88 | 0.84 | 0.92 | **0.0000** | 782 | 22 | 142 | 173 | 97.26 | 45.08 | 2.74 | 54.92 |
| np | Subtract y VS Joint Persist | argues+hyper to hyper | argues+hyper to argues+hyper | 0.66 | 0.60 | 0.72 | **0.0000** | 219 | 124 | 227 | 1085 | 63.85 | 17.30 | 36.15 | 82.70 |
| np | Subtract y VS Joint Persist | temper+hyper to hyper | temper+hyper to temper+hyper | 0.71 | 0.66 | 0.77 | **0.0000** | 381 | 93 | 262 | 636 | 80.38 | 29.18 | 19.62 | 70.82 |
| np | Subtract y VS Joint Persist | fights+hyper to hyper | fights+hyper to fights+hyper | 0.81 | 0.76 | 0.86 | **0.0000** | 581 | 34 | 152 | 181 | 94.47 | 45.65 | 5.53 | 54.35 |
| np | Subtract y VS Joint Persist | steals+hyper to hyper | steals+hyper to steals+hyper | 0.83 | 0.78 | 0.89 | **0.0000** | 609 | 30 | 100 | 124 | 95.31 | 44.64 | 4.69 | 55.36 |
| np | Subtract y VS Joint Persist | temper+argues to argues | temper+argues to temper+argues | 0.68 | 0.64 | 0.72 | **0.0000** | 661 | 178 | 465 | 1060 | 78.78 | 30.49 | 21.22 | 69.51 |
| Np | Subtract y VS Joint Persist | fights+argues to argues | fights+argues to fights+argues | 0.81 | 0.77 | 0.86 | **0.0000** | 927 | 44 | 249 | 256 | 95.47 | 49.31 | 4.53 | 50.69 |
| Np | Subtract y VS Joint Persist | steals+argues to argues | steals+argues to steals+argues | 0.86 | 0.82 | 0.90 | **0.0000** | 942 | 39 | 121 | 165 | 96.02 | 42.31 | 3.98 | 57.69 |
| Np | Subtract y VS Joint Persist | fights+temper to temper | fights+temper to fights+temper | 0.78 | 0.73 | 0.83 | **0.0000** | 631 | 54 | 165 | 219 | 92.12 | 42.97 | 7.88 | 57.03 |
| Np | Subtract y VS Joint Persist | steals+temper to temper | steals+temper to steals+temper | 0.85 | 0.79 | 0.90 | **0.0000** | 651 | 38 | 67 | 109 | 94.48 | 38.07 | 5.52 | 61.93 |
| Np | Subtract y VS Joint Persist | steals+fights to fights | steals+fights to steals+fights | 0.78 | 0.68 | 0.88 | **0.0000** | 211 | 33 | 21 | 52 | 86.48 | 28.77 | 13.52 | 71.23 |

| Supplemental Table S3N. Tetrachoric correlations from 4 x 4 pairwise analyses of all 90 combinations of the 10 selected psychological problems with every other problem to **compare differences** between three pairs of paths to the **first annual follow-up.** Results are for problems dichotomized at the **high rating cut (0 or 1 vs 2).** r_t_ = tetrachoric correlation. –CL and +CL = lower and upper 95% confidence intervals for the tetrachoric correlation. c00 = number without the predictor who followed path 1; c01 = number without the predictor who followed path 2; c10 = number with the predictor who followed path 1; c11 = number with the predictor who followed path 2. Base path 1 % = percent without predictor who followed path 1; Obs path 1 % = percent with predictor who followed path 1. Base path 2 % = percent without predictor who followed path 2; Obs path 2 % = percent with predictor who followed path 2. Raw P values significant after FDR adjustment in **bold**. | | | | | | | | | | | | | | | |
| --- | --- | --- | --- | --- | --- | --- | --- | --- | --- | --- | --- | --- | --- | --- | --- |
| prefix | Compare Paths | Path 1 | Path 2 | r_t_ | -CL | +CL | P | c00 | c01 | c10 | c11 | Baserate path 1% | Observed path 1% | Baserate path 2% | Observed path 2% |
| gh | Shift VS Add | fear to worry | fear to fear+worry | 0.25 | -.05 | 0.56 | 0.1022 | 182 | 75 | 7 | 7 | 70.82 | 50.00 | 29.18 | 50.00 |
| jl | Shift VS Add | worry to fear | worry to worry+fear | 0.55 | 0.35 | 0.75 | **0.0000** | 79 | 75 | 6 | 35 | 51.30 | 14.63 | 48.70 | 85.37 |
| gh | Shift VS Add | fear to dysph | fear to fear+dysph | 0.46 | 0.07 | 0.86 | **0.0199** | 37 | 20 | 3 | 7 | 64.91 | 30.00 | 35.09 | 70.00 |
| jl | Shift VS Add | dysph to fear | dysph to dysph+fear | 0.36 | -.09 | 0.81 | 0.1190 | 169 | 20 | 4 | 2 | 89.42 | 66.67 | 10.58 | 33.33 |
| gh | Shift VS Add | fear to worth | fear to fear+worth | 0.48 | 0.06 | 0.89 | **0.0240** | 42 | 15 | 3 | 5 | 73.68 | 37.50 | 26.32 | 62.50 |
| jl | Shift VS Add | worth to fear | worth to worth+fear | 0.49 | 0.11 | 0.86 | **0.0112** | 172 | 15 | 5 | 3 | 91.98 | 62.50 | 8.02 | 37.50 |
| gh | Shift VS Add | fear to attend | fear to fear+attend | 0.56 | 0.32 | 0.81 | **0.0000** | 354 | 32 | 8 | 7 | 91.71 | 53.33 | 8.29 | 46.67 |
| jl | Shift VS Add | attend to fear | attend to attend+fear | 0.72 | 0.58 | 0.85 | **0.0000** | 93 | 32 | 16 | 54 | 74.40 | 22.86 | 25.60 | 77.14 |
| gh | Shift VS Add | fear to hyper | fear to fear+hyper | 0.59 | 0.31 | 0.88 | **0.0001** | 204 | 14 | 7 | 5 | 93.58 | 58.33 | 6.42 | 41.67 |
| jl | Shift VS Add | hyper to fear | hyper to hyper+fear | 0.82 | 0.70 | 0.94 | **0.0000** | 138 | 14 | 14 | 29 | 90.79 | 32.56 | 9.21 | 67.44 |
| gh | Shift VS Add | fear to argues | fear to fear+argues | 0.56 | 0.28 | 0.84 | **0.0001** | 335 | 32 | 5 | 5 | 91.28 | 50.00 | 8.72 | 50.00 |
| jl | Shift VS Add | argues to fear | argues to argues+fear | 0.80 | 0.68 | 0.93 | **0.0000** | 126 | 32 | 6 | 31 | 79.75 | 16.22 | 20.25 | 83.78 |
| gh | Shift VS Add | fear to temper | fear to fear+temper | 0.28 | -.05 | 0.62 | 0.0974 | 155 | 32 | 9 | 5 | 82.89 | 64.29 | 17.11 | 35.71 |
| jl | Shift VS Add | temper to fear | temper to temper+fear | 0.53 | 0.29 | 0.77 | **0.0000** | 142 | 32 | 9 | 12 | 81.61 | 42.86 | 18.39 | 57.14 |
| gh | Shift VS Add | fear to fights | fear to fear+fights | . | . | . | . | 23 | 3 | 1 | 0 | 88.46 | 100.0 | 11.54 | 0.00 |
| jl | Shift VS Add | fights to fear | fights to fights+fear | . | . | . | . | 190 | 3 | 0 | 2 | 98.45 | 0.00 | 1.55 | 100.0 |
| gh | Shift VS Add | fear to steals | fear to fear+steals | 0.83 | 0.51 | 1.00 | **0.0000** | 28 | 1 | 3 | 3 | 96.55 | 50.00 | 3.45 | 50.00 |
| jl | Shift VS Add | steals to fear | steals to steals+fear | . | . | . | . | 192 | 1 | 0 | 2 | 99.48 | 0.00 | 0.52 | 100.0 |
| gh | Shift VS Add | worry to dysph | worry to worry+dysph | 0.84 | 0.66 | 1.00 | **0.0000** | 31 | 17 | 1 | 18 | 64.58 | 5.26 | 35.42 | 94.74 |
| jl | Shift VS Add | dysph to worry | dysph to dysph+worry | 0.72 | 0.47 | 0.97 | **0.0000** | 246 | 17 | 3 | 5 | 93.54 | 37.50 | 6.46 | 62.50 |
| gh | Shift VS Add | worry to worth | worry to worry+worth | 0.67 | 0.38 | 0.96 | **0.0000** | 30 | 20 | 2 | 13 | 60.00 | 13.33 | 40.00 | 86.67 |
| jl | Shift VS Add | worth to worry | worth to worth+worry | 0.20 | -.32 | 0.72 | 0.4553 | 245 | 20 | 5 | 1 | 92.45 | 83.33 | 7.55 | 16.67 |
| gh | Shift VS Add | worry to attend | worry to worry+attend | 0.67 | 0.51 | 0.83 | **0.0000** | 330 | 42 | 11 | 18 | 88.71 | 37.93 | 11.29 | 62.07 |
| jl | Shift VS Add | attend to worry | attend to attend+worry | 0.72 | 0.60 | 0.84 | **0.0000** | 160 | 42 | 18 | 51 | 79.21 | 26.09 | 20.79 | 73.91 |
| gh | Shift VS Add | worry to hyper | worry to worry+hyper | 0.54 | 0.28 | 0.80 | **0.0000** | 189 | 24 | 9 | 8 | 88.73 | 52.94 | 11.27 | 47.06 |
| jl | Shift VS Add | hyper to worry | hyper to hyper+worry | 0.70 | 0.55 | 0.86 | **0.0000** | 209 | 24 | 16 | 22 | 89.70 | 42.11 | 10.30 | 57.89 |
| gh | Shift VS Add | worry to argues | worry to worry+argues | 0.70 | 0.54 | 0.87 | **0.0000** | 317 | 37 | 8 | 15 | 89.55 | 34.78 | 10.45 | 65.22 |
| jl | Shift VS Add | argues to worry | argues to argues+worry | 0.72 | 0.58 | 0.85 | **0.0000** | 181 | 37 | 16 | 37 | 83.03 | 30.19 | 16.97 | 69.81 |
| gh | Shift VS Add | worry to temper | worry to worry+temper | 0.78 | 0.60 | 0.97 | **0.0000** | 158 | 30 | 2 | 11 | 84.04 | 15.38 | 15.96 | 84.62 |
| jl | Shift VS Add | temper to worry | temper to temper+worry | 0.66 | 0.47 | 0.84 | **0.0000** | 216 | 30 | 10 | 15 | 87.80 | 40.00 | 12.20 | 60.00 |
| gh | Shift VS Add | worry to fights | worry to worry+fights | 0.88 | 0.57 | 1.00 | **0.0000** | 23 | 1 | 1 | 2 | 95.83 | 33.33 | 4.17 | 66.67 |
| jl | Shift VS Add | fights to worry | fights to fights+worry | . | . | . | . | 267 | 1 | 3 | 0 | 99.63 | 100.0 | 0.37 | 0.00 |
| gh | Shift VS Add | worry to steals | worry to worry+steals | 0.82 | 0.54 | 1.00 | **0.0000** | 25 | 3 | 2 | 5 | 89.29 | 28.57 | 10.71 | 71.43 |
| jl | Shift VS Add | steals to worry | steals to steals+worry | 0.65 | 0.17 | 1.00 | **0.0074** | 264 | 3 | 3 | 1 | 98.88 | 75.00 | 1.12 | 25.00 |
| gh | Shift VS Add | dysph to worth | dysph to dysph+worth | 0.50 | 0.05 | 0.94 | **0.0287** | 43 | 16 | 2 | 4 | 72.88 | 33.33 | 27.12 | 66.67 |
| jl | Shift VS Add | worth to dysph | worth to worth+dysph | 0.72 | 0.40 | 1.00 | **0.0000** | 44 | 16 | 1 | 6 | 73.33 | 14.29 | 26.67 | 85.71 |
| gh | Shift VS Add | dysph to attend | dysph to dysph+attend | 0.74 | 0.49 | 0.98 | **0.0000** | 382 | 11 | 4 | 4 | 97.20 | 50.00 | 2.80 | 50.00 |
| jl | Shift VS Add | attend to dysph | attend to attend+dysph | 0.71 | 0.49 | 0.94 | **0.0000** | 25 | 11 | 6 | 25 | 69.44 | 19.35 | 30.56 | 80.65 |
| gh | Shift VS Add | dysph to hyper | dysph to dysph+hyper | 0.59 | 0.19 | 0.99 | **0.0035** | 215 | 10 | 3 | 2 | 95.56 | 60.00 | 4.44 | 40.00 |
| jl | Shift VS Add | hyper to dysph | hyper to hyper+dysph | 0.79 | 0.57 | 1.00 | **0.0000** | 42 | 10 | 3 | 12 | 80.77 | 20.00 | 19.23 | 80.00 |
| gh | Shift VS Add | dysph to argues | dysph to dysph+argues | 0.46 | -.06 | 0.97 | 0.0814 | 356 | 18 | 2 | 1 | 95.19 | 66.67 | 4.81 | 33.33 |
| jl | Shift VS Add | argues to dysph | argues to argues+dysph | 0.62 | 0.34 | 0.90 | **0.0000** | 26 | 18 | 4 | 19 | 59.09 | 17.39 | 40.91 | 82.61 |
| gh | Shift VS Add | dysph to temper | dysph to dysph+temper | 0.71 | 0.39 | 1.00 | **0.0000** | 178 | 19 | 1 | 3 | 90.36 | 25.00 | 9.64 | 75.00 |
| jl | Shift VS Add | temper to dysph | temper to temper+dysph | 0.29 | -.14 | 0.71 | 0.1846 | 37 | 19 | 5 | 6 | 66.07 | 45.45 | 33.93 | 54.55 |
| gh | Shift VS Add | dysph to fights | dysph to dysph+fights | . | . | . | . | 20 | 5 | 0 | 2 | 80.00 | 0.00 | 20.00 | 100.0 |
| jl | Shift VS Add | fights to dysph | fights to fights+dysph | . | . | . | . | 60 | 5 | 2 | 0 | 92.31 | 100.0 | 7.69 | 0.00 |
| gh | Shift VS Add | dysph to steals | dysph to dysph+steals | . | . | . | . | 31 | 4 | 0 | 0 | 88.57 | . | 11.43 | . |
| jl | Shift VS Add | steals to dysph | steals to steals+dysph | . | . | . | . | 62 | 4 | 0 | 1 | 93.94 | 0.00 | 6.06 | 100.0 |
| gh | Shift VS Add | worth to attend | worth to worth+attend | 0.41 | -.08 | 0.91 | 0.1038 | 384 | 12 | 4 | 1 | 96.97 | 80.00 | 3.03 | 20.00 |
| jl | Shift VS Add | attend to worth | attend to attend+worth | 0.53 | 0.22 | 0.83 | **0.0007** | 25 | 12 | 9 | 19 | 67.57 | 32.14 | 32.43 | 67.86 |
| gh | Shift VS Add | worth to hyper | worth to worth+hyper | 0.39 | -.15 | 0.93 | 0.1586 | 216 | 9 | 4 | 1 | 96.00 | 80.00 | 4.00 | 20.00 |
| jl | Shift VS Add | hyper to worth | hyper to hyper+worth | . | . | . | . | 45 | 9 | 0 | 11 | 83.33 | 0.00 | 16.67 | 100.0 |
| gh | Shift VS Add | worth to argues | worth to worth+argues | 0.72 | 0.43 | 1.00 | **0.0000** | 360 | 11 | 3 | 3 | 97.04 | 50.00 | 2.96 | 50.00 |
| jl | Shift VS Add | argues to worth | argues to argues+worth | 0.64 | 0.37 | 0.91 | **0.0000** | 29 | 11 | 7 | 18 | 72.50 | 28.00 | 27.50 | 72.00 |
| gh | Shift VS Add | worth to temper | worth to worth+temper | 0.60 | 0.25 | 0.95 | **0.0007** | 183 | 11 | 4 | 3 | 94.33 | 57.14 | 5.67 | 42.86 |
| jl | Shift VS Add | temper to worth | temper to temper+worth | 0.69 | 0.43 | 0.96 | **0.0000** | 39 | 11 | 4 | 11 | 78.00 | 26.67 | 22.00 | 73.33 |
| gh | Shift VS Add | worth to fights | worth to worth+fights | . | . | . | . | 23 | 2 | 0 | 2 | 92.00 | 0.00 | 8.00 | 100.0 |
| jl | Shift VS Add | fights to worth | fights to fights+worth | . | . | . | . | 61 | 2 | 2 | 0 | 96.83 | 100.0 | 3.17 | 0.00 |
| gh | Shift VS Add | worth to steals | worth to worth+steals | 0.82 | 0.45 | 1.00 | **0.0000** | 30 | 2 | 1 | 2 | 93.75 | 33.33 | 6.25 | 66.67 |
| jl | Shift VS Add | steals to worth | steals to steals+worth | . | . | . | . | 63 | 2 | 0 | 0 | 96.92 | . | 3.08 | . |
| gh | Shift VS Add | attend to hyper | attend to attend+hyper | 0.36 | 0.15 | 0.56 | **0.0007** | 49 | 96 | 13 | 72 | 33.79 | 15.29 | 66.21 | 84.71 |
| jl | Shift VS Add | hyper to attend | hyper to hyper+attend | 0.33 | 0.13 | 0.53 | **0.0013** | 268 | 96 | 18 | 19 | 73.63 | 48.65 | 26.37 | 51.35 |
| gh | Shift VS Add | attend to argues | attend to attend+argues | 0.72 | 0.61 | 0.82 | **0.0000** | 233 | 54 | 26 | 64 | 81.18 | 28.89 | 18.82 | 71.11 |
| jl | Shift VS Add | argues to attend | argues to argues+attend | 0.73 | 0.62 | 0.84 | **0.0000** | 275 | 54 | 21 | 51 | 83.59 | 29.17 | 16.41 | 70.83 |
| gh | Shift VS Add | attend to temper | attend to attend+temper | 0.75 | 0.62 | 0.88 | **0.0000** | 104 | 37 | 11 | 49 | 73.76 | 18.33 | 26.24 | 81.67 |
| jl | Shift VS Add | temper to attend | temper to temper+attend | 0.60 | 0.42 | 0.78 | **0.0000** | 334 | 37 | 15 | 15 | 90.03 | 50.00 | 9.97 | 50.00 |
| gh | Shift VS Add | attend to fights | attend to attend+fights | 0.57 | 0.07 | 1.00 | **0.0264** | 9 | 10 | 1 | 7 | 47.37 | 12.50 | 52.63 | 87.50 |
| jl | Shift VS Add | fights to attend | fights to fights+attend | 0.56 | 0.08 | 1.00 | **0.0231** | 388 | 10 | 2 | 1 | 97.49 | 66.67 | 2.51 | 33.33 |
| gh | Shift VS Add | attend to steals | attend to attend+steals | 0.75 | 0.46 | 1.00 | **0.0000** | 15 | 4 | 4 | 12 | 78.95 | 25.00 | 21.05 | 75.00 |
| jl | Shift VS Add | steals to attend | steals to steals+attend | 0.88 | 0.65 | 1.00 | **0.0000** | 394 | 4 | 1 | 2 | 98.99 | 33.33 | 1.01 | 66.67 |
| gh | Shift VS Add | hyper to argues | hyper to hyper+argues | 0.67 | 0.54 | 0.81 | **0.0000** | 273 | 50 | 19 | 35 | 84.52 | 35.19 | 15.48 | 64.81 |
| jl | Shift VS Add | argues to hyper | argues to argues+hyper | 0.73 | 0.59 | 0.87 | **0.0000** | 134 | 50 | 8 | 38 | 72.83 | 17.39 | 27.17 | 82.61 |
| gh | Shift VS Add | hyper to temper | hyper to hyper+temper | 0.60 | 0.42 | 0.79 | **0.0000** | 126 | 35 | 14 | 26 | 78.26 | 35.00 | 21.74 | 65.00 |
| jl | Shift VS Add | temper to hyper | temper to temper+hyper | 0.58 | 0.37 | 0.79 | **0.0000** | 170 | 35 | 10 | 15 | 82.93 | 40.00 | 17.07 | 60.00 |
| gh | Shift VS Add | hyper to fights | hyper to hyper+fights | 0.18 | -.45 | 0.80 | 0.5735 | 11 | 9 | 3 | 4 | 55.00 | 42.86 | 45.00 | 57.14 |
| jl | Shift VS Add | fights to hyper | fights to fights+hyper | 0.45 | -.09 | 0.98 | 0.1057 | 217 | 9 | 3 | 1 | 96.02 | 75.00 | 3.98 | 25.00 |
| gh | Shift VS Add | hyper to steals | hyper to hyper+steals | 0.34 | -.17 | 0.86 | 0.1943 | 18 | 7 | 5 | 5 | 72.00 | 50.00 | 28.00 | 50.00 |
| jl | Shift VS Add | steals to hyper | steals to steals+hyper | 0.71 | 0.36 | 1.00 | **0.0001** | 219 | 7 | 2 | 2 | 96.90 | 50.00 | 3.10 | 50.00 |
| gh | Shift VS Add | argues to temper | argues to argues+temper | 0.55 | 0.36 | 0.74 | **0.0000** | 65 | 75 | 8 | 53 | 46.43 | 13.11 | 53.57 | 86.89 |
| jl | Shift VS Add | temper to argues | temper to temper+argues | 0.57 | 0.39 | 0.75 | **0.0000** | 271 | 75 | 10 | 21 | 78.32 | 32.26 | 21.68 | 67.74 |
| gh | Shift VS Add | argues to fights | argues to argues+fights | 0.42 | -.13 | 0.97 | 0.1369 | 6 | 9 | 2 | 10 | 40.00 | 16.67 | 60.00 | 83.33 |
| jl | Shift VS Add | fights to argues | fights to fights+argues | 0.51 | 0.01 | 1.00 | 0.0447 | 364 | 9 | 3 | 1 | 97.59 | 75.00 | 2.41 | 25.00 |
| gh | Shift VS Add | argues to steals | argues to argues+steals | 0.39 | -.11 | 0.88 | 0.1266 | 7 | 11 | 3 | 14 | 38.89 | 17.65 | 61.11 | 82.35 |
| jl | Shift VS Add | steals to argues | steals to steals+argues | 0.81 | 0.60 | 1.00 | **0.0000** | 360 | 11 | 2 | 4 | 97.04 | 33.33 | 2.96 | 66.67 |
| gh | Shift VS Add | temper to fights | temper to temper+fights | 0.51 | -.00 | 1.00 | 0.0500 | 10 | 8 | 2 | 7 | 55.56 | 22.22 | 44.44 | 77.78 |
| jl | Shift VS Add | fights to temper | fights to fights+temper | 0.71 | 0.40 | 1.00 | **0.0000** | 187 | 8 | 3 | 3 | 95.90 | 50.00 | 4.10 | 50.00 |
| gh | Shift VS Add | temper to steals | temper to temper+steals | 0.39 | -.10 | 0.87 | 0.1167 | 15 | 9 | 4 | 7 | 62.50 | 36.36 | 37.50 | 63.64 |
| jl | Shift VS Add | steals to temper | steals to steals+temper | 0.67 | 0.38 | 0.96 | **0.0000** | 183 | 9 | 5 | 4 | 95.31 | 55.56 | 4.69 | 44.44 |
| gh | Shift VS Add | fights to steals | fights to fights+steals | . | . | . | . | 31 | 3 | 1 | 0 | 91.18 | 100.0 | 8.82 | 0.00 |
| jl | Shift VS Add | steals to fights | steals to steals+fights | . | . | . | . | 24 | 3 | 0 | 0 | 88.89 | . | 11.11 | . |
| fg | Shift VS Specific Persist | fear to worry | fear to fear | 0.42 | 0.18 | 0.67 | **0.0008** | 182 | 79 | 7 | 13 | 69.73 | 35.00 | 30.27 | 65.00 |
| jk | Shift VS Specific Persist | worry to fear | worry to worry | 0.41 | 0.21 | 0.61 | **0.0001** | 79 | 182 | 6 | 57 | 30.27 | 9.52 | 69.73 | 90.48 |
| fg | Shift VS Specific Persist | fear to dysph | fear to fear | 0.40 | 0.14 | 0.65 | **0.0021** | 37 | 169 | 3 | 60 | 17.96 | 4.76 | 82.04 | 95.24 |
| jk | Shift VS Specific Persist | dysph to fear | dysph to dysph | 0.73 | 0.55 | 0.91 | **0.0000** | 169 | 37 | 4 | 15 | 82.04 | 21.05 | 17.96 | 78.95 |
| fg | Shift VS Specific Persist | fear to worth | fear to fear | 0.43 | 0.19 | 0.67 | **0.0005** | 42 | 172 | 3 | 61 | 19.63 | 4.69 | 80.37 | 95.31 |
| jk | Shift VS Specific Persist | worth to fear | worth to worth | 0.51 | 0.24 | 0.79 | **0.0002** | 172 | 42 | 5 | 8 | 80.37 | 38.46 | 19.63 | 61.54 |
| fg | Shift VS Specific Persist | fear to attend | fear to fear | 0.73 | 0.61 | 0.85 | **0.0000** | 354 | 93 | 8 | 37 | 79.19 | 17.78 | 20.81 | 82.22 |
| jk | Shift VS Specific Persist | attend to fear | attend to attend | 0.58 | 0.47 | 0.68 | **0.0000** | 93 | 354 | 16 | 491 | 20.81 | 3.16 | 79.19 | 96.84 |
| fg | Shift VS Specific Persist | fear to hyper | fear to fear | 0.63 | 0.48 | 0.77 | **0.0000** | 204 | 138 | 7 | 47 | 59.65 | 12.96 | 40.35 | 87.04 |
| jk | Shift VS Specific Persist | hyper to fear | hyper to hyper | 0.69 | 0.59 | 0.78 | **0.0000** | 138 | 204 | 14 | 238 | 40.35 | 5.56 | 59.65 | 94.44 |
| fg | Shift VS Specific Persist | fear to argues | fear to fear | 0.77 | 0.67 | 0.88 | **0.0000** | 335 | 126 | 5 | 49 | 72.67 | 9.26 | 27.33 | 90.74 |
| jk | Shift VS Specific Persist | argues to fear | argues to argues | 0.71 | 0.62 | 0.81 | **0.0000** | 126 | 335 | 6 | 323 | 27.33 | 1.82 | 72.67 | 98.18 |
| fg | Shift VS Specific Persist | fear to temper | fear to fear | 0.52 | 0.36 | 0.69 | **0.0000** | 155 | 142 | 9 | 48 | 52.19 | 15.79 | 47.81 | 84.21 |
| jk | Shift VS Specific Persist | temper to fear | temper to temper | 0.67 | 0.55 | 0.78 | **0.0000** | 142 | 155 | 9 | 105 | 47.81 | 7.89 | 52.19 | 92.11 |
| fg | Shift VS Specific Persist | fear to fights | fear to fear | 0.49 | 0.20 | 0.79 | **0.0010** | 23 | 190 | 1 | 76 | 10.80 | 1.30 | 89.20 | 98.70 |
| jk | Shift VS Specific Persist | fights to fear | fights to fights | . | . | . | . | 190 | 23 | 0 | 4 | 89.20 | 0.00 | 10.80 | 100.0 |
| fg | Shift VS Specific Persist | fear to steals | fear to fear | 0.34 | 0.08 | 0.61 | **0.0109** | 28 | 192 | 3 | 75 | 12.73 | 3.85 | 87.27 | 96.15 |
| jk | Shift VS Specific Persist | steals to fear | steals to steals | . | . | . | . | 192 | 28 | 0 | 11 | 87.27 | 0.00 | 12.73 | 100.0 |
| fg | Shift VS Specific Persist | worry to dysph | worry to worry | 0.59 | 0.35 | 0.82 | **0.0000** | 31 | 246 | 1 | 130 | 11.19 | 0.76 | 88.81 | 99.24 |
| jk | Shift VS Specific Persist | dysph to worry | dysph to dysph | 0.74 | 0.54 | 0.94 | **0.0000** | 246 | 31 | 3 | 9 | 88.81 | 25.00 | 11.19 | 75.00 |
| fg | Shift VS Specific Persist | worry to worth | worry to worry | 0.48 | 0.25 | 0.72 | **0.0000** | 30 | 245 | 2 | 127 | 10.91 | 1.55 | 89.09 | 98.45 |
| jk | Shift VS Specific Persist | worth to worry | worth to worth | 0.53 | 0.23 | 0.82 | **0.0005** | 245 | 30 | 5 | 5 | 89.09 | 50.00 | 10.91 | 50.00 |
| fg | Shift VS Specific Persist | worry to attend | worry to worry | 0.70 | 0.60 | 0.81 | **0.0000** | 330 | 160 | 11 | 73 | 67.35 | 13.10 | 32.65 | 86.90 |
| jk | Shift VS Specific Persist | attend to worry | attend to attend | 0.69 | 0.61 | 0.77 | **0.0000** | 160 | 330 | 18 | 468 | 32.65 | 3.70 | 67.35 | 96.30 |
| fg | Shift VS Specific Persist | worry to hyper | worry to worry | 0.65 | 0.53 | 0.76 | **0.0000** | 189 | 209 | 9 | 106 | 47.49 | 7.83 | 52.51 | 92.17 |
| jk | Shift VS Specific Persist | hyper to worry | hyper to hyper | 0.76 | 0.68 | 0.83 | **0.0000** | 209 | 189 | 16 | 231 | 52.51 | 6.48 | 47.49 | 93.52 |
| fg | Shift VS Specific Persist | worry to argues | worry to worry | 0.76 | 0.67 | 0.85 | **0.0000** | 317 | 181 | 8 | 94 | 63.65 | 7.84 | 36.35 | 92.16 |
| jk | Shift VS Specific Persist | argues to worry | argues to argues | 0.66 | 0.57 | 0.75 | **0.0000** | 181 | 317 | 16 | 299 | 36.35 | 5.08 | 63.65 | 94.92 |
| fg | Shift VS Specific Persist | worry to temper | worry to worry | 0.78 | 0.68 | 0.89 | **0.0000** | 158 | 216 | 2 | 114 | 42.25 | 1.72 | 57.75 | 98.28 |
| jk | Shift VS Specific Persist | temper to worry | temper to temper | 0.69 | 0.58 | 0.80 | **0.0000** | 216 | 158 | 10 | 90 | 57.75 | 10.00 | 42.25 | 90.00 |
| fg | Shift VS Specific Persist | worry to fights | worry to worry | 0.55 | 0.30 | 0.80 | **0.0000** | 23 | 267 | 1 | 160 | 7.93 | 0.62 | 92.07 | 99.38 |
| jk | Shift VS Specific Persist | fights to worry | fights to fights | 0.68 | 0.42 | 0.94 | **0.0000** | 267 | 23 | 3 | 5 | 92.07 | 37.50 | 7.93 | 62.50 |
| fg | Shift VS Specific Persist | worry to steals | worry to worry | 0.47 | 0.24 | 0.71 | **0.0001** | 25 | 264 | 2 | 161 | 8.65 | 1.23 | 91.35 | 98.77 |
| jk | Shift VS Specific Persist | steals to worry | steals to steals | 0.81 | 0.66 | 0.97 | **0.0000** | 264 | 25 | 3 | 11 | 91.35 | 21.43 | 8.65 | 78.57 |
| fg | Shift VS Specific Persist | dysph to worth | dysph to dysph | 0.53 | 0.21 | 0.85 | **0.0012** | 43 | 44 | 2 | 13 | 49.43 | 13.33 | 50.57 | 86.67 |
| jk | Shift VS Specific Persist | worth to dysph | worth to worth | 0.62 | 0.29 | 0.94 | **0.0002** | 44 | 43 | 1 | 11 | 50.57 | 8.33 | 49.43 | 91.67 |
| fg | Shift VS Specific Persist | dysph to attend | dysph to dysph | 0.82 | 0.69 | 0.96 | **0.0000** | 382 | 25 | 4 | 12 | 93.86 | 25.00 | 6.14 | 75.00 |
| jk | Shift VS Specific Persist | attend to dysph | attend to attend | 0.44 | 0.27 | 0.62 | **0.0000** | 25 | 382 | 6 | 566 | 6.14 | 1.05 | 93.86 | 98.95 |
| fg | Shift VS Specific Persist | dysph to hyper | dysph to dysph | 0.78 | 0.62 | 0.93 | **0.0000** | 215 | 42 | 3 | 16 | 83.66 | 15.79 | 16.34 | 84.21 |
| jk | Shift VS Specific Persist | hyper to dysph | hyper to hyper | 0.67 | 0.53 | 0.82 | **0.0000** | 42 | 215 | 3 | 282 | 16.34 | 1.05 | 83.66 | 98.95 |
| fg | Shift VS Specific Persist | dysph to argues | dysph to dysph | 0.83 | 0.68 | 0.98 | **0.0000** | 356 | 26 | 2 | 9 | 93.19 | 18.18 | 6.81 | 81.82 |
| jk | Shift VS Specific Persist | argues to dysph | argues to argues | 0.45 | 0.26 | 0.65 | **0.0000** | 26 | 356 | 4 | 358 | 6.81 | 1.10 | 93.19 | 98.90 |
| fg | Shift VS Specific Persist | dysph to temper | dysph to dysph | 0.82 | 0.65 | 0.99 | **0.0000** | 178 | 37 | 1 | 11 | 82.79 | 8.33 | 17.21 | 91.67 |
| jk | Shift VS Specific Persist | temper to dysph | temper to temper | 0.44 | 0.23 | 0.65 | **0.0000** | 37 | 178 | 5 | 116 | 17.21 | 4.13 | 82.79 | 95.87 |
| fg | Shift VS Specific Persist | dysph to fights | dysph to dysph | . | . | . | . | 20 | 60 | 0 | 21 | 25.00 | 0.00 | 75.00 | 100.0 |
| jk | Shift VS Specific Persist | fights to dysph | fights to fights | 0.61 | 0.27 | 0.95 | **0.0004** | 60 | 20 | 2 | 6 | 75.00 | 25.00 | 25.00 | 75.00 |
| fg | Shift VS Specific Persist | dysph to steals | dysph to dysph | . | . | . | . | 31 | 62 | 0 | 24 | 33.33 | 0.00 | 66.67 | 100.0 |
| jk | Shift VS Specific Persist | steals to dysph | steals to steals | . | . | . | . | 62 | 31 | 0 | 11 | 66.67 | 0.00 | 33.33 | 100.0 |
| fg | Shift VS Specific Persist | worth to attend | worth to worth | 0.79 | 0.64 | 0.95 | **0.0000** | 384 | 25 | 4 | 10 | 93.89 | 28.57 | 6.11 | 71.43 |
| jk | Shift VS Specific Persist | attend to worth | attend to attend | 0.37 | 0.19 | 0.54 | **0.0000** | 25 | 384 | 9 | 570 | 6.11 | 1.55 | 93.89 | 98.45 |
| fg | Shift VS Specific Persist | worth to hyper | worth to worth | 0.69 | 0.50 | 0.88 | **0.0000** | 216 | 45 | 4 | 13 | 82.76 | 23.53 | 17.24 | 76.47 |
| jk | Shift VS Specific Persist | hyper to worth | hyper to hyper | . | . | . | . | 45 | 216 | 0 | 285 | 17.24 | 0.00 | 82.76 | 100.0 |
| fg | Shift VS Specific Persist | worth to argues | worth to worth | 0.78 | 0.61 | 0.95 | **0.0000** | 360 | 29 | 3 | 9 | 92.54 | 25.00 | 7.46 | 75.00 |
| jk | Shift VS Specific Persist | argues to worth | argues to argues | 0.37 | 0.19 | 0.55 | **0.0001** | 29 | 360 | 7 | 359 | 7.46 | 1.91 | 92.54 | 98.09 |
| fg | Shift VS Specific Persist | worth to temper | worth to worth | 0.64 | 0.41 | 0.87 | **0.0000** | 183 | 39 | 4 | 10 | 82.43 | 28.57 | 17.57 | 71.43 |
| jk | Shift VS Specific Persist | temper to worth | temper to temper | 0.49 | 0.28 | 0.69 | **0.0000** | 39 | 183 | 4 | 116 | 17.57 | 3.33 | 82.43 | 96.67 |
| fg | Shift VS Specific Persist | worth to fights | worth to worth | . | . | . | . | 23 | 61 | 0 | 20 | 27.38 | 0.00 | 72.62 | 100.0 |
| jk | Shift VS Specific Persist | fights to worth | fights to fights | 0.58 | 0.23 | 0.93 | **0.0012** | 61 | 23 | 2 | 6 | 72.62 | 25.00 | 27.38 | 75.00 |
| fg | Shift VS Specific Persist | worth to steals | worth to worth | 0.57 | 0.25 | 0.89 | **0.0006** | 30 | 63 | 1 | 20 | 32.26 | 4.76 | 67.74 | 95.24 |
| jk | Shift VS Specific Persist | steals to worth | steals to steals | . | . | . | . | 63 | 30 | 0 | 13 | 67.74 | 0.00 | 32.26 | 100.0 |
| fg | Shift VS Specific Persist | attend to hyper | attend to attend | 0.33 | 0.17 | 0.50 | **0.0001** | 49 | 268 | 13 | 215 | 15.46 | 5.70 | 84.54 | 94.30 |
| jk | Shift VS Specific Persist | hyper to attend | hyper to hyper | 0.62 | 0.46 | 0.77 | **0.0000** | 268 | 49 | 18 | 27 | 84.54 | 40.00 | 15.46 | 60.00 |
| fg | Shift VS Specific Persist | attend to argues | attend to attend | 0.70 | 0.62 | 0.77 | **0.0000** | 233 | 275 | 26 | 345 | 45.87 | 7.01 | 54.13 | 92.99 |
| jk | Shift VS Specific Persist | argues to attend | argues to argues | 0.65 | 0.55 | 0.74 | **0.0000** | 275 | 233 | 21 | 158 | 54.13 | 11.73 | 45.87 | 88.27 |
| fg | Shift VS Specific Persist | attend to temper | attend to attend | 0.66 | 0.56 | 0.76 | **0.0000** | 104 | 334 | 11 | 454 | 23.74 | 2.37 | 76.26 | 97.63 |
| jk | Shift VS Specific Persist | temper to attend | temper to temper | 0.67 | 0.55 | 0.79 | **0.0000** | 334 | 104 | 15 | 50 | 76.26 | 23.08 | 23.74 | 76.92 |
| fg | Shift VS Specific Persist | attend to fights | attend to attend | 0.51 | 0.24 | 0.79 | **0.0002** | 9 | 388 | 1 | 596 | 2.27 | 0.17 | 97.73 | 99.83 |
| jk | Shift VS Specific Persist | fights to attend | fights to fights | 0.58 | 0.10 | 1.00 | **0.0183** | 388 | 9 | 2 | 1 | 97.73 | 66.67 | 2.27 | 33.33 |
| fg | Shift VS Specific Persist | attend to steals | attend to attend | 0.40 | 0.19 | 0.61 | **0.0002** | 15 | 394 | 4 | 585 | 3.67 | 0.68 | 96.33 | 99.32 |
| jk | Shift VS Specific Persist | steals to attend | steals to steals | 0.87 | 0.71 | 1.00 | **0.0000** | 394 | 15 | 1 | 5 | 96.33 | 16.67 | 3.67 | 83.33 |
| fg | Shift VS Specific Persist | hyper to argues | hyper to hyper | 0.77 | 0.69 | 0.84 | **0.0000** | 273 | 134 | 19 | 143 | 67.08 | 11.73 | 32.92 | 88.27 |
| jk | Shift VS Specific Persist | argues to hyper | argues to argues | 0.68 | 0.58 | 0.78 | **0.0000** | 134 | 273 | 8 | 232 | 32.92 | 3.33 | 67.08 | 96.67 |
| fg | Shift VS Specific Persist | hyper to temper | hyper to hyper | 0.69 | 0.59 | 0.79 | **0.0000** | 126 | 170 | 14 | 211 | 42.57 | 6.22 | 57.43 | 93.78 |
| jk | Shift VS Specific Persist | temper to hyper | temper to temper | 0.65 | 0.52 | 0.77 | **0.0000** | 170 | 126 | 10 | 69 | 57.43 | 12.66 | 42.57 | 87.34 |
| fg | Shift VS Specific Persist | hyper to fights | hyper to hyper | 0.39 | 0.13 | 0.65 | **0.0033** | 11 | 217 | 3 | 296 | 4.82 | 1.00 | 95.18 | 99.00 |
| jk | Shift VS Specific Persist | fights to hyper | fights to fights | 0.67 | 0.35 | 0.99 | **0.0000** | 217 | 11 | 3 | 3 | 95.18 | 50.00 | 4.82 | 50.00 |
| fg | Shift VS Specific Persist | hyper to steals | hyper to hyper | 0.41 | 0.20 | 0.63 | **0.0002** | 18 | 219 | 5 | 298 | 7.59 | 1.65 | 92.41 | 98.35 |
| jk | Shift VS Specific Persist | steals to hyper | steals to steals | 0.81 | 0.62 | 1.00 | **0.0000** | 219 | 18 | 2 | 7 | 92.41 | 22.22 | 7.59 | 77.78 |
| fg | Shift VS Specific Persist | argues to temper | argues to argues | 0.50 | 0.35 | 0.65 | **0.0000** | 65 | 271 | 8 | 207 | 19.35 | 3.72 | 80.65 | 96.28 |
| jk | Shift VS Specific Persist | temper to argues | temper to temper | 0.59 | 0.42 | 0.77 | **0.0000** | 271 | 65 | 10 | 20 | 80.65 | 33.33 | 19.35 | 66.67 |
| fg | Shift VS Specific Persist | argues to fights | argues to argues | 0.25 | -.08 | 0.58 | 0.1311 | 6 | 364 | 2 | 374 | 1.62 | 0.53 | 98.38 | 99.47 |
| jk | Shift VS Specific Persist | fights to argues | fights to fights | . | . | . | . | 364 | 6 | 3 | 0 | 98.38 | 100.0 | 1.62 | 0.00 |
| fg | Shift VS Specific Persist | argues to steals | argues to argues | 0.21 | -.09 | 0.51 | 0.1711 | 7 | 360 | 3 | 377 | 1.91 | 0.79 | 98.09 | 99.21 |
| jk | Shift VS Specific Persist | steals to argues | steals to steals | 0.89 | 0.74 | 1.00 | **0.0000** | 360 | 7 | 2 | 5 | 98.09 | 28.57 | 1.91 | 71.43 |
| fg | Shift VS Specific Persist | temper to fights | temper to temper | 0.29 | -.04 | 0.62 | 0.0829 | 10 | 187 | 2 | 121 | 5.08 | 1.63 | 94.92 | 98.37 |
| jk | Shift VS Specific Persist | fights to temper | fights to fights | . | . | . | . | 187 | 10 | 3 | 0 | 94.92 | 100.0 | 5.08 | 0.00 |
| fg | Shift VS Specific Persist | temper to steals | temper to temper | 0.26 | -.02 | 0.54 | 0.0642 | 15 | 183 | 4 | 127 | 7.58 | 3.05 | 92.42 | 96.95 |
| jk | Shift VS Specific Persist | steals to temper | steals to steals | 0.63 | 0.34 | 0.91 | **0.0000** | 183 | 15 | 5 | 5 | 92.42 | 50.00 | 7.58 | 50.00 |
| fg | Shift VS Specific Persist | fights to steals | fights to fights | 0.52 | 0.06 | 0.98 | **0.0261** | 31 | 24 | 1 | 5 | 56.36 | 16.67 | 43.64 | 83.33 |
| jk | Shift VS Specific Persist | steals to fights | steals to steals | . | . | . | . | 24 | 31 | 0 | 12 | 43.64 | 0.00 | 56.36 | 100.0 |
| op | Subtract x VS Joint Persist | fear+worry to worry | fear+worry to fear+worry | 0.46 | 0.30 | 0.62 | **0.0000** | 182 | 75 | 29 | 46 | 70.82 | 38.67 | 29.18 | 61.33 |
| op | Subtract x VS Joint Persist | fear+dysph to dysph | fear+dysph to fear+dysph | 0.20 | -.28 | 0.68 | 0.4112 | 37 | 20 | 4 | 4 | 64.91 | 50.00 | 35.09 | 50.00 |
| op | Subtract x VS Joint Persist | fear+worth to worth | fear+worth to fear+worth | 0.45 | 0.07 | 0.83 | **0.0193** | 42 | 15 | 5 | 7 | 73.68 | 41.67 | 26.32 | 58.33 |
| op | Subtract x VS Joint Persist | fear+attend to attend | fear+attend to fear+attend | 0.54 | 0.38 | 0.70 | **0.0000** | 354 | 32 | 45 | 25 | 91.71 | 64.29 | 8.29 | 35.71 |
| op | Subtract x VS Joint Persist | fear+hyper to hyper | fear+hyper to fear+hyper | 0.67 | 0.50 | 0.84 | **0.0000** | 204 | 14 | 25 | 18 | 93.58 | 58.14 | 6.42 | 41.86 |
| op | Subtract x VS Joint Persist | fear+argues to argues | fear+argues to fear+argues | 0.52 | 0.33 | 0.70 | **0.0000** | 335 | 32 | 32 | 18 | 91.28 | 64.00 | 8.72 | 36.00 |
| op | Subtract x VS Joint Persist | fear+temper to temper | fear+temper to fear+temper | 0.31 | 0.02 | 0.60 | **0.0347** | 155 | 32 | 14 | 8 | 82.89 | 63.64 | 17.11 | 36.36 |
| op | Subtract x VS Joint Persist | fear+fights to fights | fear+fights to fear+fights | . | . | . | . | 23 | 3 | 0 | 0 | 88.46 | . | 11.54 | . |
| op | Subtract x VS Joint Persist | fear+steals to steals | fear+steals to fear+steals | . | . | . | . | 28 | 1 | 0 | 1 | 96.55 | 0.00 | 3.45 | 100.0 |
| op | Subtract x VS Joint Persist | worry+dysph to dysph | worry+dysph to worry+dysph | 0.39 | -.02 | 0.81 | 0.0639 | 31 | 17 | 4 | 7 | 64.58 | 36.36 | 35.42 | 63.64 |
| op | Subtract x VS Joint Persist | worry+worth to worth | worry+worth to worry+worth | 0.52 | 0.20 | 0.85 | **0.0018** | 30 | 20 | 4 | 13 | 60.00 | 23.53 | 40.00 | 76.47 |
| op | Subtract x VS Joint Persist | worry+attend to attend | worry+attend to worry+attend | 0.68 | 0.57 | 0.79 | **0.0000** | 330 | 42 | 43 | 53 | 88.71 | 44.79 | 11.29 | 55.21 |
| op | Subtract x VS Joint Persist | worry+hyper to hyper | worry+hyper to worry+hyper | 0.71 | 0.57 | 0.84 | **0.0000** | 189 | 24 | 24 | 33 | 88.73 | 42.11 | 11.27 | 57.89 |
| op | Subtract x VS Joint Persist | worry+argues to argues | worry+argues to worry+argues | 0.70 | 0.59 | 0.82 | **0.0000** | 317 | 37 | 29 | 39 | 89.55 | 42.65 | 10.45 | 57.35 |
| op | Subtract x VS Joint Persist | worry+temper to temper | worry+temper to worry+temper | 0.61 | 0.43 | 0.80 | **0.0000** | 158 | 30 | 14 | 20 | 84.04 | 41.18 | 15.96 | 58.82 |
| op | Subtract x VS Joint Persist | worry+fights to fights | worry+fights to worry+fights | . | . | . | . | 23 | 1 | 1 | 0 | 95.83 | 100.0 | 4.17 | 0.00 |
| op | Subtract x VS Joint Persist | worry+steals to steals | worry+steals to worry+steals | 0.57 | -.14 | 1.00 | 0.1132 | 25 | 3 | 1 | 1 | 89.29 | 50.00 | 10.71 | 50.00 |
| op | Subtract x VS Joint Persist | dysph+worth to worth | dysph+worth to dysph+worth | 0.68 | 0.32 | 1.00 | **0.0002** | 43 | 16 | 1 | 5 | 72.88 | 16.67 | 27.12 | 83.33 |
| op | Subtract x VS Joint Persist | dysph+attend to attend | dysph+attend to dysph+attend | 0.59 | 0.34 | 0.84 | **0.0000** | 382 | 11 | 18 | 6 | 97.20 | 75.00 | 2.80 | 25.00 |
| op | Subtract x VS Joint Persist | dysph+hyper to hyper | dysph+hyper to dysph+hyper | 0.65 | 0.39 | 0.90 | **0.0000** | 215 | 10 | 10 | 6 | 95.56 | 62.50 | 4.44 | 37.50 |
| op | Subtract x VS Joint Persist | dysph+argues to argues | dysph+argues to dysph+argues | 0.75 | 0.60 | 0.90 | **0.0000** | 356 | 18 | 13 | 14 | 95.19 | 48.15 | 4.81 | 51.85 |
| op | Subtract x VS Joint Persist | dysph+temper to temper | dysph+temper to dysph+temper | 0.58 | 0.33 | 0.84 | **0.0000** | 178 | 19 | 9 | 8 | 90.36 | 52.94 | 9.64 | 47.06 |
| op | Subtract x VS Joint Persist | dysph+fights to fights | dysph+fights to dysph+fights | . | . | . | . | 20 | 5 | 0 | 0 | 80.00 | . | 20.00 | . |
| op | Subtract x VS Joint Persist | dysph+steals to steals | dysph+steals to dysph+steals | 0.54 | -.16 | 1.00 | 0.1292 | 31 | 4 | 1 | 1 | 88.57 | 50.00 | 11.43 | 50.00 |
| op | Subtract x VS Joint Persist | worth+attend to attend | worth+attend to worth+attend | 0.49 | 0.22 | 0.77 | **0.0005** | 384 | 12 | 21 | 5 | 96.97 | 80.77 | 3.03 | 19.23 |
| op | Subtract x VS Joint Persist | worth+hyper to hyper | worth+hyper to worth+hyper | 0.64 | 0.37 | 0.91 | **0.0000** | 216 | 9 | 9 | 5 | 96.00 | 64.29 | 4.00 | 35.71 |
| op | Subtract x VS Joint Persist | worth+argues to argues | worth+argues to worth+argues | 0.64 | 0.43 | 0.86 | **0.0000** | 360 | 11 | 19 | 8 | 97.04 | 70.37 | 2.96 | 29.63 |
| op | Subtract x VS Joint Persist | worth+temper to temper | worth+temper to worth+temper | 0.54 | 0.22 | 0.87 | **0.0011** | 183 | 11 | 8 | 4 | 94.33 | 66.67 | 5.67 | 33.33 |
| op | Subtract x VS Joint Persist | worth+fights to fights | worth+fights to worth+fights | . | . | . | . | 23 | 2 | 0 | 0 | 92.00 | . | 8.00 | . |
| op | Subtract x VS Joint Persist | worth+steals to steals | worth+steals to worth+steals | . | . | . | . | 30 | 2 | 0 | 1 | 93.75 | 0.00 | 6.25 | 100.0 |
| op | Subtract x VS Joint Persist | attend+hyper to hyper | attend+hyper to attend+hyper | 0.41 | 0.26 | 0.56 | **0.0000** | 49 | 96 | 35 | 229 | 33.79 | 13.26 | 66.21 | 86.74 |
| op | Subtract x VS Joint Persist | attend+argues to argues | attend+argues to attend+argues | 0.73 | 0.65 | 0.82 | **0.0000** | 233 | 54 | 57 | 138 | 81.18 | 29.23 | 18.82 | 70.77 |
| op | Subtract x VS Joint Persist | attend+temper to temper | attend+temper to attend+temper | 0.68 | 0.54 | 0.82 | **0.0000** | 104 | 37 | 19 | 55 | 73.76 | 25.68 | 26.24 | 74.32 |
| op | Subtract x VS Joint Persist | attend+fights to fights | attend+fights to attend+fights | 0.33 | -.39 | 1.00 | 0.3732 | 9 | 10 | 1 | 3 | 47.37 | 25.00 | 52.63 | 75.00 |
| op | Subtract x VS Joint Persist | attend+steals to steals | attend+steals to attend+steals | 0.83 | 0.55 | 1.00 | **0.0000** | 15 | 4 | 1 | 6 | 78.95 | 14.29 | 21.05 | 85.71 |
| op | Subtract x VS Joint Persist | hyper+argues to argues | hyper+argues to hyper+argues | 0.73 | 0.64 | 0.82 | **0.0000** | 273 | 50 | 45 | 89 | 84.52 | 33.58 | 15.48 | 66.42 |
| op | Subtract x VS Joint Persist | hyper+temper to temper | hyper+temper to hyper+temper | 0.68 | 0.53 | 0.83 | **0.0000** | 126 | 35 | 16 | 39 | 78.26 | 29.09 | 21.74 | 70.91 |
| op | Subtract x VS Joint Persist | hyper+fights to fights | hyper+fights to hyper+fights | 0.06 | -.86 | 0.98 | 0.8923 | 11 | 9 | 1 | 1 | 55.00 | 50.00 | 45.00 | 50.00 |
| op | Subtract x VS Joint Persist | hyper+steals to steals | hyper+steals to hyper+steals | 0.44 | -.14 | 1.00 | 0.1339 | 18 | 7 | 2 | 3 | 72.00 | 40.00 | 28.00 | 60.00 |
| op | Subtract x VS Joint Persist | argues+temper to temper | argues+temper to argues+temper | 0.49 | 0.31 | 0.66 | **0.0000** | 65 | 75 | 17 | 81 | 46.43 | 17.35 | 53.57 | 82.65 |
| op | Subtract x VS Joint Persist | argues+fights to fights | argues+fights to argues+fights | . | . | . | . | 6 | 9 | 0 | 5 | 40.00 | 0.00 | 60.00 | 100.0 |
| op | Subtract x VS Joint Persist | argues+steals to steals | argues+steals to argues+steals | 0.31 | -.40 | 1.00 | 0.3921 | 7 | 11 | 1 | 4 | 38.89 | 20.00 | 61.11 | 80.00 |
| op | Subtract x VS Joint Persist | temper+fights to fights | temper+fights to temper+fights | 0.30 | -.48 | 1.00 | 0.4519 | 10 | 8 | 1 | 2 | 55.56 | 33.33 | 44.44 | 66.67 |
| op | Subtract x VS Joint Persist | temper+steals to steals | temper+steals to temper+steals | 0.31 | -.32 | 0.94 | 0.3320 | 15 | 9 | 2 | 3 | 62.50 | 40.00 | 37.50 | 60.00 |
| op | Subtract x VS Joint Persist | fights+steals to steals | fights+steals to fights+steals | 0.61 | -.06 | 1.00 | 0.0754 | 31 | 3 | 1 | 1 | 91.18 | 50.00 | 8.82 | 50.00 |
| np | Subtract y VS Joint Persist | worry+fear to fear | worry+fear to worry+fear | 0.45 | 0.25 | 0.64 | **0.0000** | 79 | 75 | 13 | 46 | 51.30 | 22.03 | 48.70 | 77.97 |
| np | Subtract y VS Joint Persist | dysph+fear to fear | dysph+fear to dysph+fear | 0.39 | 0.04 | 0.74 | **0.0291** | 169 | 20 | 8 | 4 | 89.42 | 66.67 | 10.58 | 33.33 |
| np | Subtract y VS Joint Persist | worth+fear to fear | worth+fear to worth+fear | 0.67 | 0.43 | 0.92 | **0.0000** | 172 | 15 | 6 | 7 | 91.98 | 46.15 | 8.02 | 53.85 |
| np | Subtract y VS Joint Persist | attend+fear to fear | attend+fear to attend+fear | 0.63 | 0.43 | 0.82 | **0.0000** | 93 | 32 | 10 | 25 | 74.40 | 28.57 | 25.60 | 71.43 |
| np | Subtract y VS Joint Persist | hyper+fear to fear | hyper+fear to hyper+fear | 0.80 | 0.65 | 0.94 | **0.0000** | 138 | 14 | 9 | 18 | 90.79 | 33.33 | 9.21 | 66.67 |
| np | Subtract y VS Joint Persist | argues+fear to fear | argues+fear to argues+fear | 0.67 | 0.48 | 0.86 | **0.0000** | 126 | 32 | 7 | 18 | 79.75 | 28.00 | 20.25 | 72.00 |
| np | Subtract y VS Joint Persist | temper+fear to fear | temper+fear to temper+fear | 0.21 | -.08 | 0.51 | 0.1517 | 142 | 32 | 18 | 8 | 81.61 | 69.23 | 18.39 | 30.77 |
| np | Subtract y VS Joint Persist | fights+fear to fear | fights+fear to fights+fear | . | . | . | . | 190 | 3 | 3 | 0 | 98.45 | 100.0 | 1.55 | 0.00 |
| np | Subtract y VS Joint Persist | steals+fear to fear | steals+fear to steals+fear | . | . | . | . | 192 | 1 | 0 | 1 | 99.48 | 0.00 | 0.52 | 100.0 |
| np | Subtract y VS Joint Persist | dysph+worry to worry | dysph+worry to dysph+worry | 0.56 | 0.31 | 0.82 | **0.0000** | 246 | 17 | 12 | 7 | 93.54 | 63.16 | 6.46 | 36.84 |
| np | Subtract y VS Joint Persist | worth+worry to worry | worth+worry to worth+worry | 0.66 | 0.47 | 0.85 | **0.0000** | 245 | 20 | 14 | 13 | 92.45 | 51.85 | 7.55 | 48.15 |
| np | Subtract y VS Joint Persist | attend+worry to worry | attend+worry to attend+worry | 0.68 | 0.56 | 0.81 | **0.0000** | 160 | 42 | 23 | 53 | 79.21 | 30.26 | 20.79 | 69.74 |
| np | Subtract y VS Joint Persist | hyper+worry to worry | hyper+worry to hyper+worry | 0.75 | 0.63 | 0.88 | **0.0000** | 209 | 24 | 20 | 33 | 89.70 | 37.74 | 10.30 | 62.26 |
| np | Subtract y VS Joint Persist | argues+worry to worry | argues+worry to argues+worry | 0.70 | 0.56 | 0.83 | **0.0000** | 181 | 37 | 19 | 39 | 83.03 | 32.76 | 16.97 | 67.24 |
| np | Subtract y VS Joint Persist | temper+worry to worry | temper+worry to temper+worry | 0.57 | 0.39 | 0.75 | **0.0000** | 216 | 30 | 22 | 20 | 87.80 | 52.38 | 12.20 | 47.62 |
| np | Subtract y VS Joint Persist | fights+worry to worry | fights+worry to fights+worry | . | . | . | . | 267 | 1 | 5 | 0 | 99.63 | 100.0 | 0.37 | 0.00 |
| np | Subtract y VS Joint Persist | steals+worry to worry | steals+worry to steals+worry | . | . | . | . | 264 | 3 | 0 | 1 | 98.88 | 0.00 | 1.12 | 100.0 |
| np | Subtract y VS Joint Persist | worth+dysph to dysph | worth+dysph to worth+dysph | 0.47 | 0.06 | 0.88 | **0.0258** | 44 | 16 | 3 | 5 | 73.33 | 37.50 | 26.67 | 62.50 |
| np | Subtract y VS Joint Persist | attend+dysph to dysph | attend+dysph to attend+dysph | 0.50 | 0.07 | 0.93 | **0.0227** | 25 | 11 | 3 | 6 | 69.44 | 33.33 | 30.56 | 66.67 |
| np | Subtract y VS Joint Persist | hyper+dysph to dysph | hyper+dysph to hyper+dysph | 0.80 | 0.53 | 1.00 | **0.0000** | 42 | 10 | 1 | 6 | 80.77 | 14.29 | 19.23 | 85.71 |
| np | Subtract y VS Joint Persist | argues+dysph to dysph | argues+dysph to argues+dysph | 0.77 | 0.52 | 1.00 | **0.0000** | 26 | 18 | 1 | 14 | 59.09 | 6.67 | 40.91 | 93.33 |
| np | Subtract y VS Joint Persist | temper+dysph to dysph | temper+dysph to temper+dysph | 0.52 | 0.16 | 0.88 | **0.0051** | 37 | 19 | 3 | 8 | 66.07 | 27.27 | 33.93 | 72.73 |
| np | Subtract y VS Joint Persist | fights+dysph to dysph | fights+dysph to fights+dysph | . | . | . | . | 60 | 5 | 2 | 0 | 92.31 | 100.0 | 7.69 | 0.00 |
| np | Subtract y VS Joint Persist | steals+dysph to dysph | steals+dysph to steals+dysph | . | . | . | . | 62 | 4 | 0 | 1 | 93.94 | 0.00 | 6.06 | 100.0 |
| np | Subtract y VS Joint Persist | attend+worth to worth | attend+worth to attend+worth | 0.14 | -.34 | 0.63 | 0.5590 | 25 | 12 | 7 | 5 | 67.57 | 58.33 | 32.43 | 41.67 |
| np | Subtract y VS Joint Persist | hyper+worth to worth | hyper+worth to hyper+worth | 0.56 | 0.18 | 0.95 | **0.0040** | 45 | 9 | 4 | 5 | 83.33 | 44.44 | 16.67 | 55.56 |
| np | Subtract y VS Joint Persist | argues+worth to worth | argues+worth to argues+worth | 0.62 | 0.27 | 0.96 | **0.0005** | 29 | 11 | 3 | 8 | 72.50 | 27.27 | 27.50 | 72.73 |
| np | Subtract y VS Joint Persist | temper+worth to worth | temper+worth to temper+worth | 0.29 | -.18 | 0.76 | 0.2307 | 39 | 11 | 6 | 4 | 78.00 | 60.00 | 22.00 | 40.00 |
| np | Subtract y VS Joint Persist | fights+worth to worth | fights+worth to fights+worth | . | . | . | . | 61 | 2 | 1 | 0 | 96.83 | 100.0 | 3.17 | 0.00 |
| np | Subtract y VS Joint Persist | steals+worth to worth | steals+worth to steals+worth | . | . | . | . | 63 | 2 | 0 | 1 | 96.92 | 0.00 | 3.08 | 100.0 |
| np | Subtract y VS Joint Persist | hyper+attend to attend | hyper+attend to hyper+attend | 0.63 | 0.55 | 0.71 | **0.0000** | 268 | 96 | 99 | 229 | 73.63 | 30.18 | 26.37 | 69.82 |
| np | Subtract y VS Joint Persist | argues+attend to attend | argues+attend to argues+attend | 0.73 | 0.65 | 0.81 | **0.0000** | 275 | 54 | 68 | 138 | 83.59 | 33.01 | 16.41 | 66.99 |
| np | Subtract y VS Joint Persist | temper+attend to attend | temper+attend to temper+attend | 0.66 | 0.55 | 0.77 | **0.0000** | 334 | 37 | 57 | 55 | 90.03 | 50.89 | 9.97 | 49.11 |
| np | Subtract y VS Joint Persist | fights+attend to attend | fights+attend to fights+attend | 0.56 | 0.24 | 0.88 | **0.0005** | 388 | 10 | 9 | 3 | 97.49 | 75.00 | 2.51 | 25.00 |
| np | Subtract y VS Joint Persist | steals+attend to attend | steals+attend to steals+attend | 0.80 | 0.62 | 0.99 | **0.0000** | 394 | 4 | 12 | 6 | 98.99 | 66.67 | 1.01 | 33.33 |
| np | Subtract y VS Joint Persist | argues+hyper to hyper | argues+hyper to argues+hyper | 0.59 | 0.46 | 0.72 | **0.0000** | 134 | 50 | 43 | 89 | 72.83 | 32.58 | 27.17 | 67.42 |
| np | Subtract y VS Joint Persist | temper+hyper to hyper | temper+hyper to temper+hyper | 0.57 | 0.41 | 0.73 | **0.0000** | 170 | 35 | 34 | 39 | 82.93 | 46.58 | 17.07 | 53.42 |
| np | Subtract y VS Joint Persist | fights+hyper to hyper | fights+hyper to fights+hyper | 0.22 | -.31 | 0.75 | 0.4111 | 217 | 9 | 9 | 1 | 96.02 | 90.00 | 3.98 | 10.00 |
| np | Subtract y VS Joint Persist | steals+hyper to hyper | steals+hyper to steals+hyper | 0.70 | 0.39 | 1.00 | **0.0000** | 219 | 7 | 4 | 3 | 96.90 | 57.14 | 3.10 | 42.86 |
| np | Subtract y VS Joint Persist | temper+argues to argues | temper+argues to temper+argues | 0.53 | 0.41 | 0.65 | **0.0000** | 271 | 75 | 63 | 81 | 78.32 | 43.75 | 21.68 | 56.25 |
| np | Subtract y VS Joint Persist | fights+argues to argues | fights+argues to fights+argues | 0.61 | 0.35 | 0.87 | **0.0000** | 364 | 9 | 15 | 5 | 97.59 | 75.00 | 2.41 | 25.00 |
| np | Subtract y VS Joint Persist | steals+argues to argues | steals+argues to steals+argues | 0.61 | 0.33 | 0.89 | **0.0000** | 360 | 11 | 9 | 4 | 97.04 | 69.23 | 2.96 | 30.77 |
| np | Subtract y VS Joint Persist | fights+temper to temper | fights+temper to fights+temper | 0.40 | -.03 | 0.83 | 0.0689 | 187 | 8 | 9 | 2 | 95.90 | 81.82 | 4.10 | 18.18 |
| np | Subtract y VS Joint Persist | steals+temper to temper | steals+temper to steals+temper | 0.74 | 0.45 | 1.00 | **0.0000** | 183 | 9 | 2 | 3 | 95.31 | 40.00 | 4.69 | 60.00 |
| np | Subtract y VS Joint Persist | steals+fights to fights | steals+fights to steals+fights | . | . | . | . | 24 | 3 | 0 | 1 | 88.89 | 0.00 | 11.11 | 100.0 |

| Supplemental Table S3O. Tetrachoric correlations from 4 x 4 pairwise analyses of all 90 combinations of the 10 selected psychological problems with every other problem to **compare differences** between three pairs of paths to the **second annual follow-up.** Results are for problems dichotomized at the **low rating cut (0 vs 1 or 2).** r_t_ = tetrachoric correlation. –CL and +CL = lower and upper 95% confidence intervals for the tetrachoric correlation. c00 = number without the predictor who followed path 1; c01 = number without the predictor who followed path 2; c10 = number with the predictor who followed path 1; c11 = number with the predictor who followed path 2. Base path 1 % = percent without predictor who followed path 1; Obs path 1 % = percent with predictor who followed path 1. Base path 2 % = percent without predictor who followed path 2; Obs path 2 % = percent with predictor who followed path 2. Raw P values significant after FDR adjustment in **bold**. | | | | | | | | | | | | | | | |
| --- | --- | --- | --- | --- | --- | --- | --- | --- | --- | --- | --- | --- | --- | --- | --- |
| prefix | Compare Paths | Path 1 | Path 2 | r_t_ | -CL | +CL | P | c00 | c01 | c10 | c11 | Baserate path 1% | Observed path 1% | Baserate path 2% | Observed path 2% |
| gh | Shift VS Add | fear to worry | fear to fear+worry | 0.43 | 0.34 | 0.52 | **0.0000** | 606 | 280 | 76 | 127 | 68.40 | 37.44 | 31.60 | 62.56 |
| jl | Shift VS Add | worry to fear | worry to worry+fear | 0.34 | 0.23 | 0.44 | **0.0000** | 180 | 280 | 70 | 279 | 39.13 | 20.06 | 60.87 | 79.94 |
| gh | Shift VS Add | fear to dysph | fear to fear+dysph | 0.54 | 0.45 | 0.63 | **0.0000** | 376 | 205 | 71 | 187 | 64.72 | 27.52 | 35.28 | 72.48 |
| jl | Shift VS Add | dysph to fear | dysph to dysph+fear | 0.47 | 0.36 | 0.58 | **0.0000** | 482 | 205 | 43 | 79 | 70.16 | 35.25 | 29.84 | 64.75 |
| gh | Shift VS Add | fear to worth | fear to fear+worth | 0.53 | 0.43 | 0.62 | **0.0000** | 272 | 171 | 61 | 176 | 61.40 | 25.74 | 38.60 | 74.26 |
| jl | Shift VS Add | worth to fear | worth to worth+fear | 0.55 | 0.44 | 0.66 | **0.0000** | 526 | 171 | 38 | 74 | 75.47 | 33.93 | 24.53 | 66.07 |
| gh | Shift VS Add | fear to attend | fear to fear+attend | 0.59 | 0.49 | 0.68 | **0.0000** | 597 | 134 | 62 | 91 | 81.67 | 40.52 | 18.33 | 59.48 |
| jl | Shift VS Add | attend to fear | attend to attend+fear | 0.69 | 0.63 | 0.76 | **0.0000** | 239 | 134 | 74 | 362 | 64.08 | 16.97 | 35.92 | 83.03 |
| gh | Shift VS Add | fear to hyper | fear to fear+hyper | 0.60 | 0.49 | 0.70 | **0.0000** | 346 | 98 | 49 | 87 | 77.93 | 36.03 | 22.07 | 63.97 |
| jl | Shift VS Add | hyper to fear | hyper to hyper+fear | 0.69 | 0.62 | 0.77 | **0.0000** | 435 | 98 | 92 | 184 | 81.61 | 33.33 | 18.39 | 66.67 |
| gh | Shift VS Add | fear to argues | fear to fear+argues | 0.64 | 0.57 | 0.72 | **0.0000** | 712 | 124 | 83 | 117 | 85.17 | 41.50 | 14.83 | 58.50 |
| jl | Shift VS Add | argues to fear | argues to argues+fear | 0.69 | 0.62 | 0.76 | **0.0000** | 272 | 124 | 86 | 327 | 68.69 | 20.82 | 31.31 | 79.18 |
| gh | Shift VS Add | fear to temper | fear to fear+temper | 0.54 | 0.42 | 0.65 | **0.0000** | 387 | 95 | 61 | 76 | 80.29 | 44.53 | 19.71 | 55.47 |
| jl | Shift VS Add | temper to fear | temper to temper+fear | 0.68 | 0.61 | 0.76 | **0.0000** | 466 | 95 | 90 | 158 | 83.07 | 36.29 | 16.93 | 63.71 |
| gh | Shift VS Add | fear to fights | fear to fear+fights | 0.61 | 0.47 | 0.76 | **0.0000** | 128 | 47 | 24 | 56 | 73.14 | 30.00 | 26.86 | 70.00 |
| jl | Shift VS Add | fights to fear | fights to fights+fear | 0.62 | 0.49 | 0.76 | **0.0000** | 702 | 47 | 35 | 25 | 93.72 | 58.33 | 6.28 | 41.67 |
| gh | Shift VS Add | fear to steals | fear to fear+steals | 0.67 | 0.52 | 0.83 | **0.0000** | 100 | 31 | 17 | 43 | 76.34 | 28.33 | 23.66 | 71.67 |
| jl | Shift VS Add | steals to fear | steals to steals+fear | 0.71 | 0.57 | 0.84 | **0.0000** | 735 | 31 | 24 | 19 | 95.95 | 55.81 | 4.05 | 44.19 |
| gh | Shift VS Add | worry to dysph | worry to worry+dysph | 0.47 | 0.38 | 0.56 | **0.0000** | 224 | 228 | 80 | 307 | 49.56 | 20.67 | 50.44 | 79.33 |
| jl | Shift VS Add | dysph to worry | dysph to dysph+worry | 0.41 | 0.29 | 0.52 | **0.0000** | 758 | 228 | 48 | 55 | 76.88 | 46.60 | 23.12 | 53.40 |
| gh | Shift VS Add | worry to worth | worry to worry+worth | 0.55 | 0.46 | 0.65 | **0.0000** | 172 | 172 | 55 | 281 | 50.00 | 16.37 | 50.00 | 83.63 |
| jl | Shift VS Add | worth to worry | worth to worth+worry | 0.54 | 0.44 | 0.64 | **0.0000** | 803 | 172 | 49 | 65 | 82.36 | 42.98 | 17.64 | 57.02 |
| gh | Shift VS Add | worry to attend | worry to worry+attend | 0.59 | 0.51 | 0.67 | **0.0000** | 463 | 167 | 81 | 173 | 73.49 | 31.89 | 26.51 | 68.11 |
| jl | Shift VS Add | attend to worry | attend to attend+worry | 0.72 | 0.67 | 0.78 | **0.0000** | 381 | 167 | 101 | 440 | 69.53 | 18.67 | 30.47 | 81.33 |
| gh | Shift VS Add | worry to hyper | worry to worry+hyper | 0.54 | 0.43 | 0.64 | **0.0000** | 260 | 110 | 71 | 139 | 70.27 | 33.81 | 29.73 | 66.19 |
| jl | Shift VS Add | hyper to worry | hyper to hyper+worry | 0.73 | 0.67 | 0.79 | **0.0000** | 639 | 110 | 118 | 222 | 85.31 | 34.71 | 14.69 | 65.29 |
| gh | Shift VS Add | worry to argues | worry to worry+argues | 0.53 | 0.45 | 0.61 | **0.0000** | 556 | 167 | 130 | 183 | 76.90 | 41.53 | 23.10 | 58.47 |
| jl | Shift VS Add | argues to worry | argues to argues+worry | 0.69 | 0.63 | 0.75 | **0.0000** | 447 | 167 | 115 | 360 | 72.80 | 24.21 | 27.20 | 75.79 |
| gh | Shift VS Add | worry to temper | worry to worry+temper | 0.56 | 0.46 | 0.66 | **0.0000** | 300 | 108 | 75 | 136 | 73.53 | 35.55 | 26.47 | 64.45 |
| jl | Shift VS Add | temper to worry | temper to temper+worry | 0.74 | 0.68 | 0.80 | **0.0000** | 711 | 108 | 94 | 176 | 86.81 | 34.81 | 13.19 | 65.19 |
| gh | Shift VS Add | worry to fights | worry to worry+fights | 0.52 | 0.36 | 0.68 | **0.0000** | 100 | 53 | 31 | 71 | 65.36 | 30.39 | 34.64 | 69.61 |
| jl | Shift VS Add | fights to worry | fights to fights+worry | 0.58 | 0.44 | 0.72 | **0.0000** | 977 | 53 | 38 | 21 | 94.85 | 64.41 | 5.15 | 35.59 |
| gh | Shift VS Add | worry to steals | worry to worry+steals | 0.56 | 0.39 | 0.73 | **0.0000** | 79 | 35 | 24 | 53 | 69.30 | 31.17 | 30.70 | 68.83 |
| jl | Shift VS Add | steals to worry | steals to steals+worry | 0.78 | 0.68 | 0.89 | **0.0000** | 1016 | 35 | 17 | 21 | 96.67 | 44.74 | 3.33 | 55.26 |
| gh | Shift VS Add | dysph to worth | dysph to dysph+worth | 0.41 | 0.29 | 0.54 | **0.0000** | 324 | 252 | 27 | 77 | 56.25 | 25.96 | 43.75 | 74.04 |
| jl | Shift VS Add | worth to dysph | worth to worth+dysph | 0.50 | 0.39 | 0.61 | **0.0000** | 457 | 252 | 35 | 95 | 64.46 | 26.92 | 35.54 | 73.08 |
| gh | Shift VS Add | dysph to attend | dysph to dysph+attend | 0.46 | 0.32 | 0.59 | **0.0000** | 690 | 126 | 36 | 32 | 84.56 | 52.94 | 15.44 | 47.06 |
| jl | Shift VS Add | attend to dysph | attend to attend+dysph | 0.72 | 0.65 | 0.78 | **0.0000** | 201 | 126 | 71 | 441 | 61.47 | 13.87 | 38.53 | 86.13 |
| gh | Shift VS Add | dysph to hyper | dysph to dysph+hyper | 0.48 | 0.33 | 0.62 | **0.0000** | 419 | 84 | 40 | 37 | 83.30 | 51.95 | 16.70 | 48.05 |
| jl | Shift VS Add | hyper to dysph | hyper to hyper+dysph | 0.76 | 0.71 | 0.82 | **0.0000** | 428 | 84 | 95 | 232 | 83.59 | 29.05 | 16.41 | 70.95 |
| gh | Shift VS Add | dysph to argues | dysph to dysph+argues | 0.44 | 0.31 | 0.57 | **0.0000** | 807 | 148 | 44 | 37 | 84.50 | 54.32 | 15.50 | 45.68 |
| jl | Shift VS Add | argues to dysph | argues to argues+dysph | 0.71 | 0.64 | 0.77 | **0.0000** | 220 | 148 | 64 | 407 | 59.78 | 13.59 | 40.22 | 86.41 |
| gh | Shift VS Add | dysph to temper | dysph to dysph+temper | 0.52 | 0.38 | 0.65 | **0.0000** | 436 | 116 | 27 | 40 | 78.99 | 40.30 | 21.01 | 59.70 |
| jl | Shift VS Add | temper to dysph | temper to temper+dysph | 0.73 | 0.67 | 0.79 | **0.0000** | 427 | 116 | 77 | 219 | 78.64 | 26.01 | 21.36 | 73.99 |
| gh | Shift VS Add | dysph to fights | dysph to dysph+fights | 0.55 | 0.38 | 0.72 | **0.0000** | 143 | 58 | 17 | 37 | 71.14 | 31.48 | 28.86 | 68.52 |
| jl | Shift VS Add | fights to dysph | fights to fights+dysph | 0.68 | 0.57 | 0.79 | **0.0000** | 713 | 58 | 33 | 35 | 92.48 | 48.53 | 7.52 | 51.47 |
| gh | Shift VS Add | dysph to steals | dysph to dysph+steals | 0.41 | 0.19 | 0.63 | **0.0002** | 93 | 56 | 14 | 28 | 62.42 | 33.33 | 37.58 | 66.67 |
| jl | Shift VS Add | steals to dysph | steals to steals+dysph | 0.70 | 0.58 | 0.81 | **0.0000** | 721 | 56 | 29 | 33 | 92.79 | 46.77 | 7.21 | 53.23 |
| gh | Shift VS Add | worth to attend | worth to worth+attend | 0.60 | 0.48 | 0.73 | **0.0000** | 721 | 101 | 27 | 35 | 87.71 | 43.55 | 12.29 | 56.45 |
| jl | Shift VS Add | attend to worth | attend to attend+worth | 0.77 | 0.70 | 0.83 | **0.0000** | 170 | 101 | 46 | 363 | 62.73 | 11.25 | 37.27 | 88.75 |
| gh | Shift VS Add | worth to hyper | worth to worth+hyper | 0.59 | 0.47 | 0.72 | **0.0000** | 424 | 86 | 28 | 42 | 83.14 | 40.00 | 16.86 | 60.00 |
| jl | Shift VS Add | hyper to worth | hyper to hyper+worth | 0.69 | 0.62 | 0.77 | **0.0000** | 326 | 86 | 82 | 186 | 79.13 | 30.60 | 20.87 | 69.40 |
| gh | Shift VS Add | worth to argues | worth to worth+argues | 0.65 | 0.56 | 0.75 | **0.0000** | 822 | 108 | 45 | 61 | 88.39 | 42.45 | 11.61 | 57.55 |
| jl | Shift VS Add | argues to worth | argues to argues+worth | 0.74 | 0.67 | 0.81 | **0.0000** | 201 | 108 | 53 | 318 | 65.05 | 14.29 | 34.95 | 85.71 |
| gh | Shift VS Add | worth to temper | worth to worth+temper | 0.53 | 0.40 | 0.66 | **0.0000** | 429 | 108 | 34 | 48 | 79.89 | 41.46 | 20.11 | 58.54 |
| jl | Shift VS Add | temper to worth | temper to temper+worth | 0.70 | 0.62 | 0.77 | **0.0000** | 342 | 108 | 60 | 170 | 76.00 | 26.09 | 24.00 | 73.91 |
| gh | Shift VS Add | worth to fights | worth to worth+fights | 0.63 | 0.49 | 0.78 | **0.0000** | 148 | 44 | 20 | 43 | 77.08 | 31.75 | 22.92 | 68.25 |
| jl | Shift VS Add | fights to worth | fights to fights+worth | 0.67 | 0.54 | 0.79 | **0.0000** | 573 | 44 | 33 | 30 | 92.87 | 52.38 | 7.13 | 47.62 |
| gh | Shift VS Add | worth to steals | worth to worth+steals | 0.69 | 0.53 | 0.85 | **0.0000** | 116 | 35 | 10 | 30 | 76.82 | 25.00 | 23.18 | 75.00 |
| jl | Shift VS Add | steals to worth | steals to steals+worth | 0.76 | 0.65 | 0.86 | **0.0000** | 590 | 35 | 25 | 30 | 94.40 | 45.45 | 5.60 | 54.55 |
| gh | Shift VS Add | attend to hyper | attend to attend+hyper | 0.51 | 0.39 | 0.62 | **0.0000** | 99 | 173 | 34 | 275 | 36.40 | 11.00 | 63.60 | 89.00 |
| jl | Shift VS Add | hyper to attend | hyper to hyper+attend | 0.52 | 0.42 | 0.63 | **0.0000** | 597 | 173 | 44 | 70 | 77.53 | 38.60 | 22.47 | 61.40 |
| gh | Shift VS Add | attend to argues | attend to attend+argues | 0.70 | 0.64 | 0.76 | **0.0000** | 394 | 172 | 97 | 373 | 69.61 | 20.64 | 30.39 | 79.36 |
| jl | Shift VS Add | argues to attend | argues to argues+attend | 0.69 | 0.62 | 0.76 | **0.0000** | 399 | 172 | 66 | 247 | 69.88 | 21.09 | 30.12 | 78.91 |
| gh | Shift VS Add | attend to temper | attend to attend+temper | 0.73 | 0.66 | 0.81 | **0.0000** | 170 | 99 | 48 | 302 | 63.20 | 13.71 | 36.80 | 86.29 |
| jl | Shift VS Add | temper to attend | temper to temper+attend | 0.67 | 0.58 | 0.75 | **0.0000** | 607 | 99 | 73 | 105 | 85.98 | 41.01 | 14.02 | 58.99 |
| gh | Shift VS Add | attend to fights | attend to attend+fights | 0.80 | 0.69 | 0.90 | **0.0000** | 47 | 34 | 13 | 161 | 58.02 | 7.47 | 41.98 | 92.53 |
| jl | Shift VS Add | fights to attend | fights to fights+attend | 0.61 | 0.45 | 0.77 | **0.0000** | 806 | 34 | 29 | 15 | 95.95 | 65.91 | 4.05 | 34.09 |
| gh | Shift VS Add | attend to steals | attend to attend+steals | 0.74 | 0.58 | 0.89 | **0.0000** | 24 | 32 | 7 | 128 | 42.86 | 5.19 | 57.14 | 94.81 |
| jl | Shift VS Add | steals to attend | steals to steals+attend | 0.61 | 0.42 | 0.80 | **0.0000** | 827 | 32 | 16 | 9 | 96.27 | 64.00 | 3.73 | 36.00 |
| gh | Shift VS Add | hyper to argues | hyper to hyper+argues | 0.71 | 0.64 | 0.77 | **0.0000** | 637 | 125 | 93 | 181 | 83.60 | 33.94 | 16.40 | 66.06 |
| jl | Shift VS Add | argues to hyper | argues to argues+hyper | 0.62 | 0.53 | 0.72 | **0.0000** | 181 | 125 | 50 | 225 | 59.15 | 18.18 | 40.85 | 81.82 |
| gh | Shift VS Add | hyper to temper | hyper to hyper+temper | 0.70 | 0.62 | 0.78 | **0.0000** | 296 | 70 | 81 | 172 | 80.87 | 32.02 | 19.13 | 67.98 |
| jl | Shift VS Add | temper to hyper | temper to temper+hyper | 0.70 | 0.62 | 0.79 | **0.0000** | 341 | 70 | 57 | 112 | 82.97 | 33.73 | 17.03 | 66.27 |
| gh | Shift VS Add | hyper to fights | hyper to hyper+fights | 0.74 | 0.63 | 0.85 | **0.0000** | 85 | 29 | 30 | 111 | 74.56 | 21.28 | 25.44 | 78.72 |
| jl | Shift VS Add | fights to hyper | fights to fights+hyper | 0.67 | 0.51 | 0.82 | **0.0000** | 512 | 29 | 22 | 17 | 94.64 | 56.41 | 5.36 | 43.59 |
| gh | Shift VS Add | hyper to steals | hyper to hyper+steals | 0.54 | 0.37 | 0.72 | **0.0000** | 54 | 28 | 32 | 77 | 65.85 | 29.36 | 34.15 | 70.64 |
| jl | Shift VS Add | steals to hyper | steals to steals+hyper | 0.70 | 0.55 | 0.85 | **0.0000** | 521 | 28 | 16 | 15 | 94.90 | 51.61 | 5.10 | 48.39 |
| gh | Shift VS Add | argues to temper | argues to argues+temper | 0.53 | 0.42 | 0.64 | **0.0000** | 101 | 161 | 40 | 317 | 38.55 | 11.20 | 61.45 | 88.80 |
| jl | Shift VS Add | temper to argues | temper to temper+argues | 0.62 | 0.53 | 0.70 | **0.0000** | 700 | 161 | 65 | 110 | 81.30 | 37.14 | 18.70 | 62.86 |
| gh | Shift VS Add | argues to fights | argues to argues+fights | 0.62 | 0.44 | 0.80 | **0.0000** | 22 | 47 | 10 | 176 | 31.88 | 5.38 | 68.12 | 94.62 |
| jl | Shift VS Add | fights to argues | fights to fights+argues | 0.59 | 0.43 | 0.75 | **0.0000** | 948 | 47 | 26 | 15 | 95.28 | 63.41 | 4.72 | 36.59 |
| gh | Shift VS Add | argues to steals | argues to argues+steals | 0.42 | 0.18 | 0.66 | **0.0007** | 17 | 46 | 12 | 116 | 26.98 | 9.38 | 73.02 | 90.63 |
| jl | Shift VS Add | steals to argues | steals to steals+argues | 0.57 | 0.39 | 0.74 | **0.0000** | 959 | 46 | 20 | 11 | 95.42 | 64.52 | 4.58 | 35.48 |
| gh | Shift VS Add | temper to fights | temper to temper+fights | 0.49 | 0.32 | 0.65 | **0.0000** | 55 | 64 | 24 | 112 | 46.22 | 17.65 | 53.78 | 82.35 |
| jl | Shift VS Add | fights to temper | fights to fights+temper | 0.46 | 0.27 | 0.65 | **0.0000** | 520 | 64 | 21 | 14 | 89.04 | 60.00 | 10.96 | 40.00 |
| gh | Shift VS Add | temper to steals | temper to temper+steals | 0.65 | 0.50 | 0.81 | **0.0000** | 78 | 44 | 13 | 56 | 63.93 | 18.84 | 36.07 | 81.16 |
[truncated: 184,046 more chars]
